# Genome-wide identification and transcriptional profiling analysis of auxin response-related gene families in cucumber

**DOI:** 10.1186/1756-0500-7-218

**Published:** 2014-04-08

**Authors:** Jian Wu, Songyu Liu, Xiaoyan Guan, Lifei Chen, Yanjun He, Jie Wang, Gang Lu

**Affiliations:** 1 Key Laboratory of Horticultural Plant Growth, Development and Biotechnology, Agricultural Ministry of China, Department of Horticulture, Zhejiang University, Zijingang Campus, A535 Agriculture building, Hangzhou 310058, Zhejiang, China

## Abstract

**Background:**

Auxin signaling has a vital function in the regulation of plant growth and development, both which are known to be mediated by auxin-responsive genes. So far, significant progress has been made toward the identification and characterization of auxin-response genes in several model plants, while no systematic analysis for these families was reported in cucumber (*Cucumis sativus* L.), a reference species for Cucurbitaceae crops. The comprehensive analyses will help design experiments for functional validation of their precise roles in plant development and stress responses.

**Results:**

A genome-wide search for auxin-response gene homologues identified 16 auxin-response factors (ARFs), 27 auxin/indole acetic acids (Aux/IAAs), 10 Gretchen Hagen 3 (GH3s), 61 small auxin-up mRNAs (SAURs), and 39 lateral organ boundaries (LBDs) in cucumber. Sequence analysis together with the organization of putative motifs indicated the potential diverse functions of these five auxin-related family members. The distribution and density of auxin response-related genes on chromosomes were not uniform. Evolutionary analysis showed that the chromosomal segment duplications mainly contributed to the expansion of the CsARF, CsIAA, CsGH3, and CsLBD gene families. Quantitative real-time RT-PCR analysis demonstrated that many ARFs, AUX/IAAs, GH3s, SAURs, and LBD genes were expressed in diverse patterns within different organs/tissues and during different development stages. They were also implicated in IAA, methyl jasmonic acid, or salicylic acid response, which is consistent with the finding that a great number of diverse cis-elements are present in their promoter regions involving a variety of signaling transduction pathways.

**Conclusion:**

Genome-wide comparative analysis of auxin response-related family genes and their expression analysis provide new evidence for the potential role of auxin in development and hormone response of plants. Our data imply that the auxin response genes may be involved in various vegetative and reproductive developmental processes. Furthermore, they will be involved in different signal pathways and may mediate the crosstalk between various hormone responses.

## Background

Auxin, which is widely distributed in higher plants, has long been recognized as an essential plant hormone involved in diverse processes of plant growth and development, including plant root formation, apical dominance, senescence, fruit development, and abiotic and biotic stress responses. Auxin often rapidly induces the expression alteration of many auxin response-related genes, referred to as primary or early auxin response genes, including auxin/indoleacetic acid (Aux/IAA), Gretchen Hagen 3 (GH3), small auxin up mRNA (SAUR), and lateral organ boundaries (LBD) [1-4]. Molecular genetic and biochemical findings have suggested that the interaction of Aux/IAAs and auxin response factor (ARF) has a central function in the auxin signaling transduction pathway. Under low auxin concentration, ARF protein activities are inhibited by dimerization with Aux/IAAs [1,5,6]. Elevated auxin concentration causes ARFs to be released from a repressor heterodimer by promoting the degradation of Aux/IAA proteins through the ubiquitin-proteasome protein (TIR1) pathway [7-9]. The released ARFs can activate or repress the expression of other primary/early auxin response genes by binding to auxin response elements (AuxREs) on the promoters of these genes [7].

A typical ARF protein contains a conserved N-terminal B3-like DNA-binding domain (DBD) that regulates the expression of auxin response genes, a conserved C-terminal dimerization domain (CTD) that resembles domains III and IV in Aux/IAA proteins, and a variable middle region (MR) [10,11]. Aux/IAA proteins generally have four characteristic domains: I, II, III, and IV [11,12]. Except for domain II that is known to be involved in protein stability, domains I, III, and IV are repression domains (RDs) with additional functions in different processes. Domain I is an N-terminal RD represented by an LxLxL motif [13]. This domain can interact with the TOPLESS co-repressor [14]. Domains III and IV, the C-terminal domains, can repress the function of ARFs and subsequently repress auxin signaling transduction through the dimerization of ARFs with the CTD [6,15-17]. However, no conserved motif or domain has ever been found in the GH3 and SAUR proteins [1,18,19]. Although SAUR proteins are not highly homologous to any other published domains, the central regions of these proteins are quite conservative [19]. The N-terminal lateral organ boundaries (LOB) domain is approximately 100 amino acids in length [20] and typically contains three highly conservative regions, including C-domain, Gly-Ala-Ser (GAS) block, and predicted coiled-coil motif [20,21]. The C-domain contains four highly conserved cysteine (C) residues arranged in a CX_2_CX_6_CX_3_C motif, which is required for DNA binding. Similarly, the predicted coiled-coil motif contains four perfectly conserved leucine residues in a LX6LX3LX6L spacing that is reminiscent of a leucine zipper and may provide protein interaction [22].

Genome-wide analysis indicated that ARFs and AUX/IAAs are encoded by relatively large gene families in *Arabidopsis*, rice, maize, sorghum, *Populus trichocarpa*, tomato, soybean etc. [1,10,18,19,23-27]. Functional identification revealed that these genes have important functions in many aspects of plant development. In *Arabidopsis*, AtARF1 and AtARF2 can regulate leaf senescence and floral organ abscission in a partially redundant manner [28]. AtARF2 also functions as a transcriptional repressor involved in the auxin-mediated control of *Arabidopsis* leaf longevity [29]. IAA28 can promote lateral root initiation in response to auxin signals as a transcription repressor [30]. In rice, OsARF12 is implicated in regulating root elongation as a transcription activator [31]. In tomato, three ARF genes (SlARF4, SlARF7, and SlARF10) and one Aux/IAA gene (SlIAA9) exhibit different functions during fruit development [32-35]. Although many GH3 and SAUR genes from different species have been published, the precise functions of these genes remain unclear. Some GH3 genes in *Arabidopsis* are involved in maintaining auxin homeostasis by conjugating excess IAA to amino acids [36]. JAR1 (GH3.11) can conjugate jasmonic acid (JA) to amino acids [37]. SAUR genes may have important functions in the regulation of cell elongation in soybean and maize [38-40] and cell expansion in *Arabidopsis*[41]. In rice, OsAUR39 acts as a repressor of auxin synthesis and transport [42]. A few reports on the biological roles of LBD genes are available. AtAS2 (AtLBD6) has an important role in flat leaf formation and flower development [43]. LBD16 and LBD18 can influence lateral root formation in *Arabidopsis*[44]. LBD18 is also involved in regulating tracheary element differentiation [45]. The ortholog of AtLBD16 in rice can regulate leaf formation [46].

Cucumber (*Cucumis sativus* L*.*), an economically important crop of the botanical family Cucurbitaceae, is considered as one of the model dicot plants for molecular and genetic studies. As a fresh-fruit plant, cucumber has a few traits that may have been consequences of various auxin gene networks. However, only five CsARFs and three CsIAAs have previously been identified in cucumber [47,48]. To the best of our knowledge, no systematic investigations on auxin response gene families have been reported in cucumber. Taking advantage of the available cucumber genome database [49], a genome-wide search was carried out in the present study to find the homologues of auxin response gene families in cucumber. A total of 16 ARFs, 27 IAAs, 10 GH3s, 61 SAURs, and 39 LBDs were identified from the cucumber genome. Detailed information on the genomic structures, chromosomal locations, and sequence homology of these genes was presented. In addition, the phylogenetic relationship between the auxin response genes in cucumber and those in *Arabidopsis*, rice, and maize were also compared. Subsequently, the different temporal and spatial expression patterns of this family of genes during fruiting and under IAA, JA and SA treatment in cucumber plants were also compared through quantitative real-time PCR (qRT-PCR).

## Results and discussion

### Identification of auxin response genes in cucumber

To identify all auxin response genes in cucumber, BLAST searches on the cucumber genome database were performed using the published peptide sequences of ARF, AUX/IAA, GH3, SAUR, and LBD genes from *Arabidopsis*, rice, tomato, and maize as query sequences. A total of 181 candidates for auxin-related genes were predicted from the cucumber genome database using the TBLASTN program. These predicted peptide sequences of the candidates were analyzed through BLASTP of NCBI to check their corresponding conserved domains. The ones without anticipant domains were removed. After these analyses, the cucumber genomes appeared to have 16 ARF, 27 AUX/IAA, 10 GH3, 61 SAUR, and 39 LBD genes, referred to as *CsARF, CsIAA, CsGH3, CsSAUR,* and *CsLBD*, respectively (Additional file 1: Table S1). The number of CsARF, CsIAA, CsGH3, CsSAUR, and CsLBD members of cucumber is comparable with that of *Arabidopsis* (*23 AtARFs*, *29 AtIAAs*, *20 AtGH3s*, *72 AtSAURs*, and *42 AtLBDs*) and rice (*25 OsARFs*, *31 OsIAAs*, *13 OsGH3s*, *58 OsSAURs*, and *35 OsLBDs*). However, cucumber, *Arabidopsis*, and rice have different genome sizes (cucumber, ~243.5 Mb; *Arabidopsis*, ~125 Mb; and rice, ~420 Mb) [49]. The observed similarity partially accounts for the conservation of auxin response genes in these three species.

It is worth mentioning the nomenclature system for auxin response genes used in the present study. Distinctive names for the CsARF, CsIAA, and CsGH3 families were given according to their homologous genes in *Arabidopsis*. Some ARF, IAA, and GH3 family genes identified in cucumber without apparent homologous genes in *Arabidopsis* were named according to the order of identification. Conversely, given that sequence analysis indicated that the similarity in SAUR and LBD amino acid sequences between *Arabidopsis* and cucumber was low, distinctive names for each of the SAUR and LBD family members identified in this study were given according to their position from the top to the bottom on the cucumber chromosomes 1 to 7.

### Classification and gene structure analysis

**Figure 1:**
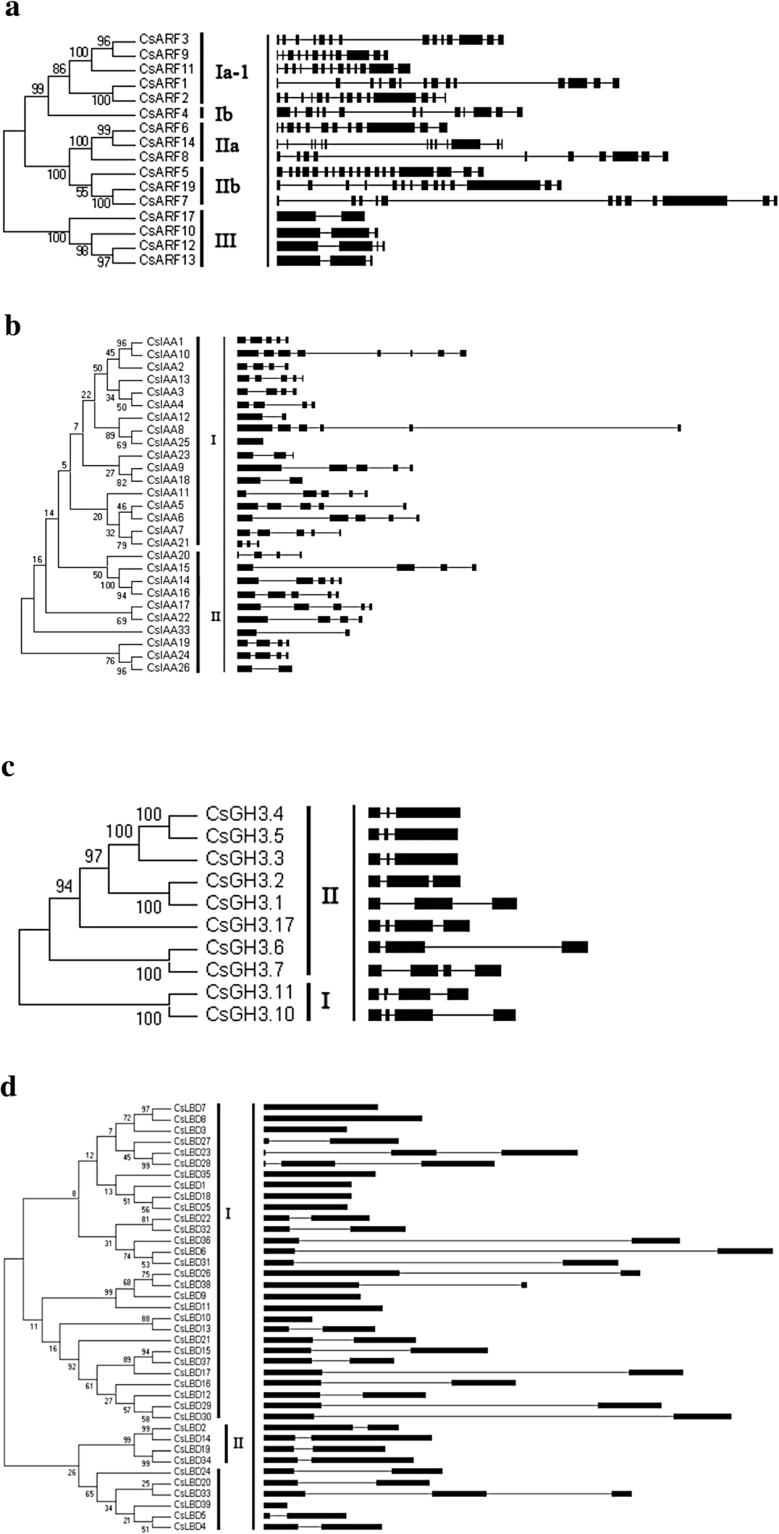
**The phylogenetic relationships and gene structure of cucumber auxin response-related genes.** The left part in different panel illustrates the relationships among the cucumber ARF **(a)**, AUX/IAA **(b)** GH3 **(c)**, LBD **(d)** proteins. The unrooted phylogenetic tree was generated using MEGA4.1 through the neighbor joining method. Bootstrap values (above 50%) from 1,000 replicates are indicated at each branch. The right part illustrates the exon–intron organization of ARF **(a)**, AUX/IAA **(b)** GH3 **(c),** LBD **(d)** family genes. The exons and introns are represented by black boxes and lines, respectively.

Phylogenetic analysis showed that 16 CsARF proteins can be divided into three major groups (groups I to III), wherein groups I and II can be further divided two subgroups (Figure 1a). A similar scenario was reported in *Arabidopsis* and tomato [26]. The CsARF genes in the same group display similar exon and intron structures, especially those in group III, with much fewer intron numbers (Figure 1a; Additional file 1: Table S1-1). Interestingly, all the Q*-*rich SlARFs clustered in group II. Moreover, they formed one triplet and one sister pair (*CsARF6*/*CsARF14*/*CsARF8* and *CsARF7*/*CsARF19*) with very strong bootstrap support (>99%).

Similar to tomato and *Arabidopsis*, 27 *CsIAA* genes formed two groups (groups I and II) (Figure 1b). However, the gene structure was different within the same group, although all *CsIAA* genes, except for *CsIAA25*, were interrupted by introns. The number of introns ranged from one (*CsIAA12*, *CsIAA18*, *CsIAA26*, and *CsIAA33*) to seven (*CsIAA10*) (Figure 1b; Additional file 1: Table S1-2). Similarly, all 61 *CsSAURs* can be divided into two distinct groups with 9 and 52 members (Additional file 2: Figure S1-4). However, no intron was identified from most *CsSAUR* genes, and only seven *CsSAUR* genes, including *CsSAUR14*, *CsSAUR15*, *CsSAUR19*, *CsSAUR32*, *CsSAUR42*, *CsSAUR50*, and *CsSAUR56*, contained one intron (Additional file 1: Table S1-5).

The GH3 gene family is highly conserved in both dicots and monocots [18]. Alignment of deduced amino acid sequences of cucumber *CsGH3* revealed that 10 *CsGH3* genes can be divided into two groups with two and eight members, respectively. The GH3 domains among these proteins were highly conserved. The multiple sequence alignment of the full-length OsGH3 proteins using ClustalX revealed high similarity, ranging from 94% to 100%. Furthermore, a comparison of the full-length cDNA sequences with corresponding genomic DNA sequences showed that the gene structure was also similar among the *CsGH3* genes. The coding sequences of all 10 *CsGH3s* were disrupted by two or three introns (Figure 1c; Additional file 1: Table S1-3). This finding is consistent with a previous report in rice [18].

According to the sequence homologs, all 39 *CsLBD* proteins were divided into two groups (groups I and II) (Figure 1d). A similar classification was reported in *Arabidopsis*, rice, and sorghum [26]. Group I consisted of 35 members, whereas group II contained the remaining four members. Most *CsLBDs* contain one or two introns (Figure 1d; Additional file 1: Table S1-4).

### Protein sequences analysis in auxin-related genes

**Figure 2:**
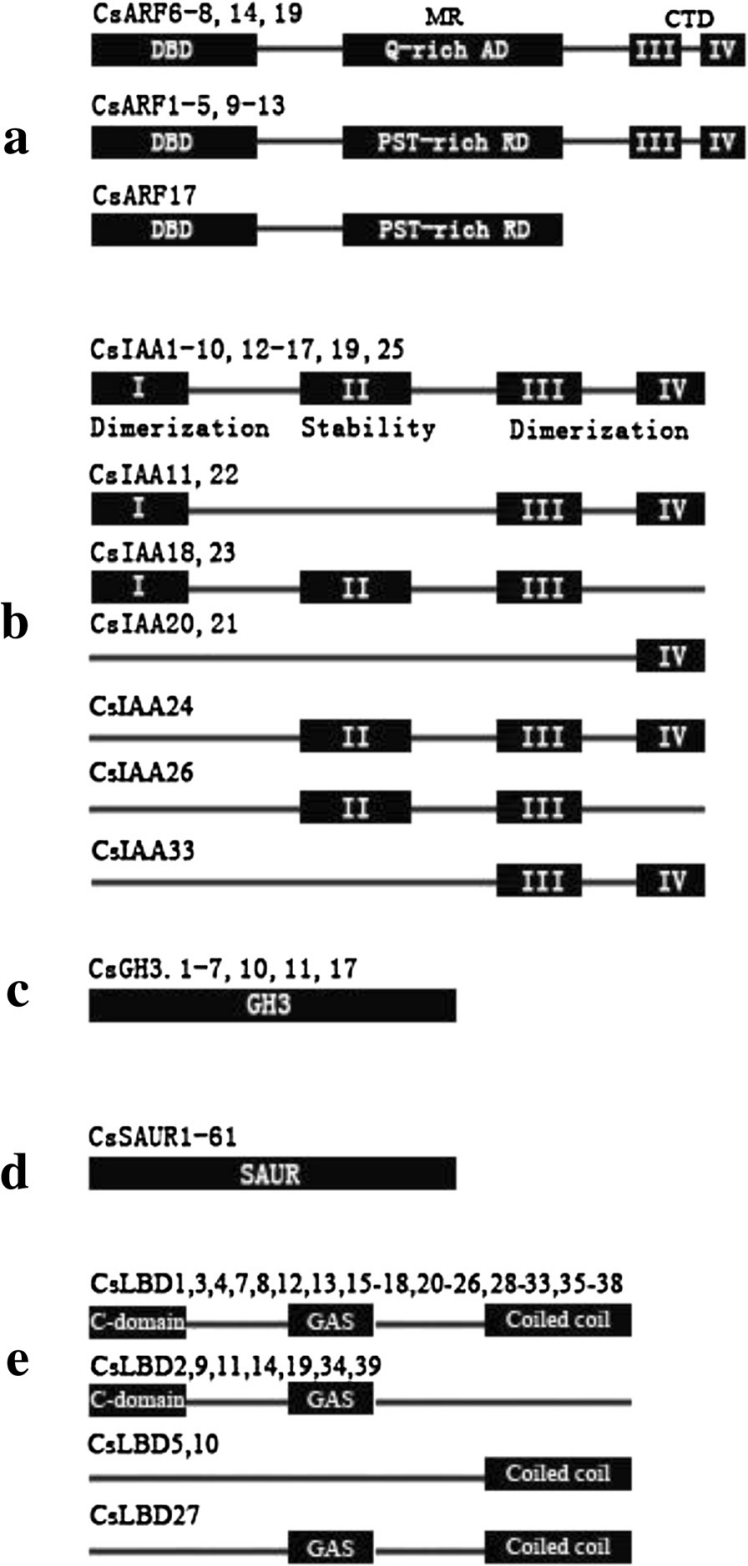
**Domain distribution of CsARF, CsIAA, CsGH3, CsSAUR, and CsLBD gene families on their peptide sequences. (a)** CsARF contains a DNA-binding domain (DBD), a middle region (MR), and a C-terminus domain (CTD). MRs rich in glutamine (Q-rich) are activator domains (ADs), whereas those rich in proline, serine, and threonine (PST-rich) are repressor domains (RDs). CsARF17 lacks CTD. **(b)** CsIAA proteins consist of four domains, I, II, III, and IV, but several members lack one or more of the four domains. **(c)** CsGH3 proteins contain a highly conservative GH3 domain. **(d)** CsSAUR proteins contain a highly conservative SAUR domain. **(e)** The LOB domain of LBD genes consists of three highly conservative regions: C-domain, GAS block, and coiled coil.

All cucumber CsARF protein sequences were found to contain DBDs, MRs, and CTDs (domains III and IV), except for *CsARF17* that lacked the CTD domain (Figure 2; Additional file 1: Table S1-1; Additional file 3: Figure S2-1). A previous study proved that the ARF MRs function either as activation domains (ADs) or RDs [50]. In *Arabidopsis*, protoplast transfection assays indicated that *AtARF1*, *AtARF2*, *AtARF4*, and *AtARF9* containing MRs rich in proline (P), serine (S), and threonine (T) act as repressors and that *AtARF5*, *AtARF6*, *AtARF7*, and *AtARF8* containing MRs rich in glutamine (Q) act as transcriptional activators [7,51]. In the present study, *CsARF1-5*, *CsARF9-13*, and *CsARF17* in cucumber possessed MRs rich in proline (P), serine (S), or threonine (T), indicating their role as repressors. By contrast, *CsARF6-8*, *CsARF14*, and *CsARF19* had MRs rich in glutamine (Q), implying their role as transcriptional activators (Figure 2). Only *CsARF17* lacked the CTD, indicating that it may regulate the expression of other auxin response genes in an auxin-independent manner [52].

Eighteen CsIAA proteins, including CsIAA1-CsIAA10, CsIAA12-CsIAA17, CsIAA19, and CsIAA25, contained all four highly conserved domains (domains I, II, III, and IV). However, some CsIAA genes lacked domain I, II, or IV, whereas others contained only one or two of these conserved domains (Figure 2; Additional file 1: Table S1-2; Additional file 3: Figure S2-2). *CsIAA24, CsIAA26, and CsIAA33* did not contain domain I, an active repression domain that was transferable and dominant over activation domains. Five CsIAA proteins, including CsIAA11, CsIAA12, CsIAA20, CsIAA21, and CsIAA33 lacked domain II, which plays important functions in protein stability [8,17-20]. Furthermore, domain II is responsible for the degradation of AUX/IAA proteins by physically interacting with TIR1 under a high level of auxin [53]. Previous studies demonstrated that the half-lives of proteins without domain II were much longer than those of canonical Aux/IAA proteins [54]. Similarly, two and six non-canonical AUX/IAA genes were found in tomato and *Arabidopsis*, respectively [55]. The expression levels of these non-canonical Aux/IAA genes reported so far were low in *Arabidopsis* and tomato. These genes might be relatively insensitive to IAA treatment, indicating that they might have a specific function in mediating auxin signaling during well-defined plant developmental events [53-56]. However, no consistent roles can be assigned to these Aux/IAA proteins that lack domain II until now [54]. The deduced CsIAA20 and CsIAA21 might be pseudogenes because they only contain domain IV. No information about their expression is available.

No previously-known conserved motifs or domains were found in cucumber GH3 and SAUR proteins (Figure 2; Additional file 1: Table S1-3, 4; Additional file 3: Figure S2, 3, 4). A similar finding was reported in previous studies [18]. However, the central regions of GH3 and SAUR proteins in rice and *Arabidopsis* are highly conserved [18,54]. Our data also showed that CsGH3 proteins contained the conserved central regions in cucumber (Additional file 3: Figure S2-3), implying that they might be essential and might be performing similar functions. Similarly, five putative motifs were identified from 61 CsSAURs*.* Interestingly, all deduced SAUR proteins contained motifs 1 and 2 (Additional file 3: Figure S2-4), indicating that these two motifs were extremely conserved during the evolutionary history of different species and that they are essential for SAUR functions. Motifs 3 to 5 can only be found in about half of the members, indicating their distinct origin and function (Additional file 3: Figure S2-4).

The LBD gene family encodes proteins harboring a conserved plant-specific LOB domain [20]. In cucumber, 29 CsLBD proteins all contained highly conserved regions within the LOB domain, including a conserved four-Cys motif (C-domain), a GAS block, and a leucine zipper-like coiled-coil motif. Previous studies showed that the LBD genes classified to class II all lack the coiled-coil motif in their LOB domain [20-22]. In the present study, four CsLBD genes in class II were found to lack the coiled-coil region: CsLBD2, CsLBD14, CsLBD19, and CsLBD34 (Figure 2; Additional file 3: Figure 2-5). Unexpectively, CsLBD9, CsLBD11, and CsLBD39 belonging to class I also lacked the coiled-coil region (Figure 2; Additional file 3: Figure S2-5). Unfortunately, limited functional information is available for these non-canonical genes in the LBD family.

### Chromosomal distribution and tandem duplication

**Figure 3:**
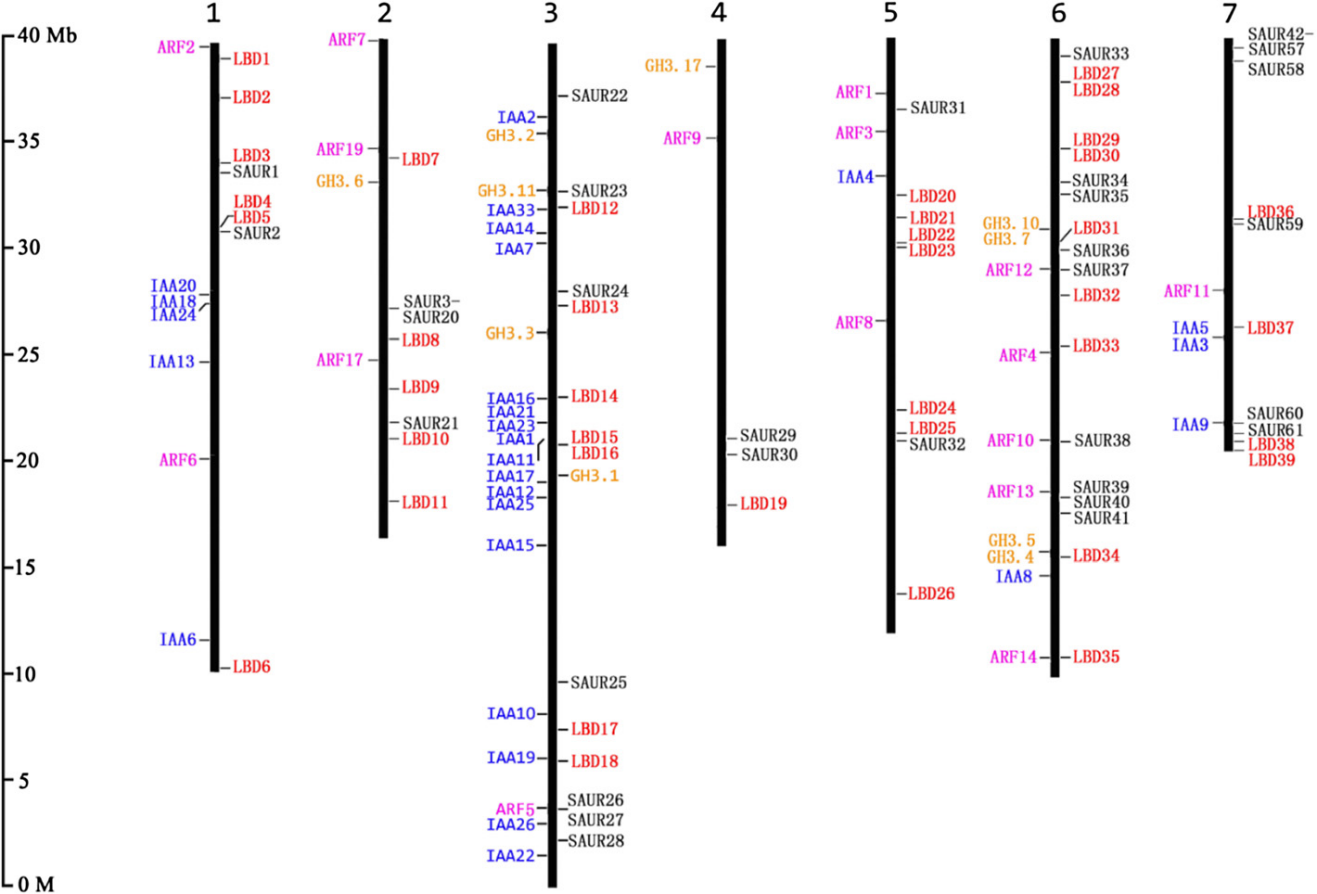
**Genomic distribution of *CsARF, CsIAA, CsGH3, CsSAUR, *and *CsLBD *genes on cucumber chromosomes.** The chromosome number is indicated at the top of each chromosome.

The chromosomal locations of all auxin-related genes were determined and demonstrated using BLASTN analysis on the cucumber genome database. The distribution and density of the auxin-responsive genes on chromosomes were not uniform. Cucumber *ARF*, *SAUR*, and *LBD* genes were present on all seven chromosomes (Figure 3; Additional file 1: Tables S1-1, 4, and 5), and the *CsIAA* genes were present on all chromosomes, except on chromosomes 2 and 4. Conversely, the *CsGH3* genes were only localized on chromosomes 2, 3, 4, and 6. A total of 19 *CsSAUR* genes were localized on chromosome 2, 18 of which clustered on the same region. Similarly, 16 out of the 20 *CsSAUR* genes localized on chromosome 7 formed a cluster (Figure 3). Moreover, all members of these two clusters were also gathered into three clades on the phylogenetic tree (Additional file 2; Figure S1-4, colored in blue). Hence, tandem duplications might have had a crucial function in the evolution of the CsSAUR gene family. In tomato, our previous study found that eight gene clusters were located physically near each other in four chromosomes because many tandem-duplications were present in the tomato genome.

Large-scale or whole-genome duplication and tandem duplications of the cucumber genome have been reported [49]. Phylogenetic analysis revealed one triplet (CsARF6/CsARF8/CsARF14) and two sister pairs (CsARF1/CsARF2 and CsARF7/CsARF19) of CsARFs, three sister pairs (CsGH3.1/CsGH3.2, CsGH3.6/CsGH3.7, and CsGH3.10/CsGH3.11) and one triplet (CsGH3.3/CsGH3.4/CsGH3.5) of CsGH3s, and three CsLBD sister pairs (CsLBD23/CsLBD28, CsLBD2/CsLBD14, and CsLBD19/CsLBD34) in the phylogenetic tree. However, when all sister pairs and triplets were compared with their corresponding chromosomal locations, none of these sister pairs were genetically linked, except for *CsGH3.4* and *CsGH3.5*. The clades of CsIAAs with relatively strong bootstrap support (>90%), such as CsIAA1/CsIAA10, CsIAA14/CsIAA15/CsIAA16, and CsIAA24/CsIAA26, were also located in different chromosomes or far apart on the same chromosome*.* Based on these results, we can conclude that the entire genome or the chromosomal segment duplications are the main factors responsible for the expansion of the CsARF, CsIAA, CsGH3, and CsLBD gene families.

### Evolutionary analysis of the ARF, Aux/IAA, SAUR, GH3, and LBD gene families

To investigate the evolutionary relationships of the auxin response proteins in different species, the full-length protein sequences of auxin response genes from cucumber and other species, such as *Arabidopsis*, rice, maize, sorghum, and tomato, were used to build the phylogenetic trees. All 140 ARF proteins from the six species (tomato, rice, maize, sorghum, cucumber, and *Arabidopsis*) can be classified into four major groups (classes I to IV). Class I can be further divided into classes 1a-1, 1a-2, and 1-b, whereas class II can also be further divided into classes IIa and IIb (Additional file 2: Figure S1-1). Similar results were found in our previous study [26].

All 144 IAA genes from rice, maize, sorghum, cucumber, and *Arabidopsis* were divided into two classes (classes I and II) in accordance with a previous study [27] (Additional file 2: Figure S1-2). According to previous study [18], 60 GH3 genes from rice, sorghum, cucumber, and *Arabidopsis* were classified into three major classes (classes I to III) (Additional file 2: Figure S1-3). A total of 133 SAUR genes, including 61 CsSAURs and 73 AtSAURs, were divided into two classes based on the phylogenetic relationship and the methods reported in a previous study [19] (Additional file 2: Figure S1-4). Up to 39 CsLBD genes and 42 AtLBD genes were divided into two classes according to the method of Majer and Hochholdinger [20] (Additional file 2: Figure S1-5).

Classification of the auxin response genes from phylogenetic trees revealed that most classes or subclasses contained genes from different species, implying that these genes originated prior to species differentiation (Additional file 2: Figure S1). However, one class of GH3 (class III) and one subclass of ARFs (class Ia-2) (Additional file 2: Figure S1-1; Additional file 2: Figure S1-3) only contained genes from the *Arabidopsis* genome. This result, which is consistent with previous studies [18,26], indicating that these genes were generated over the long-term evolution of *Arabidopsis* and may have species-specific functions. Some clades contained sequence representatives from *Arabidopsis*, tomato, and cucumber, but not from rice and sorghum. The combined phylogenetic analysis revealed eight triplets and four sister pairs of ARF family genes among rice and sorghum, as well as one triplet and six sister pairs among *Arabidopsis*, tomato, and cucumber. However, only one sister pair (OsARF14/AtARF14) was found in the ARF gene family between monocots and dicots, indicating that the auxin response genes experienced significant evolution for a long period after the divergence of monocots and dicots.

### Expression profiles of the five gene families

**Figure 4:**
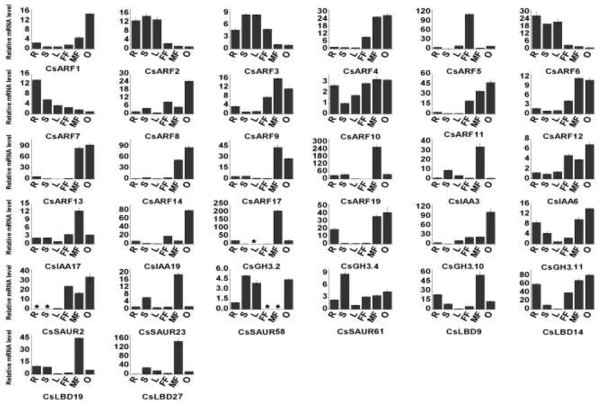
**Expression profiles of 32 randomly-selected auxin response genes in different cucumber organs.** QRT-PCR analysis of total RNA isolated from the root (R), stem (S), leaf (L), female flower buds (FF), male flower buds (MF), and ovaries (O) were used to assess the transcript levels of selected genes in flowering cucumber plants. The data were presented as mean ± SD normalized relative to *EF1a* (accession number EF446145) gene transcript levels. All samples were run in triplicate, and the entire assay was performed twice for each biological pool.

Transcript abundance in particular organs at a given time is an important factor in elucidating the function of a corresponding protein required in developmental, metabolic, and signaling processes. Although the expression of most of the 32 selected auxin response genes can be detected in most selected organs, their expression levels varied considerably. *CsARF1* and *CsGH3.19* were mainly expressed in cucumber ovaries (Figure 4), implying that they might have important functions in the development of the ovary. *CsARF9*, *CsARF17*, *CsARF19*, *CsIAA3*, *CsIAA17*, *CsGH3*.2, *CsSAUR23*, *CsLBD9*, *CsLBD19*, and *CsLBD27* might have crucial functions in male flowers because of their higher expression levels in this organ than in other organs (Figure 4). *CsARF5* was mainly expressed in female flowers; thus, it may have a crucial function in the development of female flowers (Figure 4). *CsARF2, CsARF3, CsARF6*, and *CsARF7* may have more important functions during the vegetative growth of cucumber plant because they are mainly expressed in vegetative organs (roots, stems, and leaves) (Figure 4). By contrast, *CsARF4*, *CsARF9*, *CsARF11*, *CsARF12*, *CsARF13*, *CsARF14*, *CsARF17*, and *CsIAA6* may be more important during reproductive growth (Figure 4).

**Figure 5:**
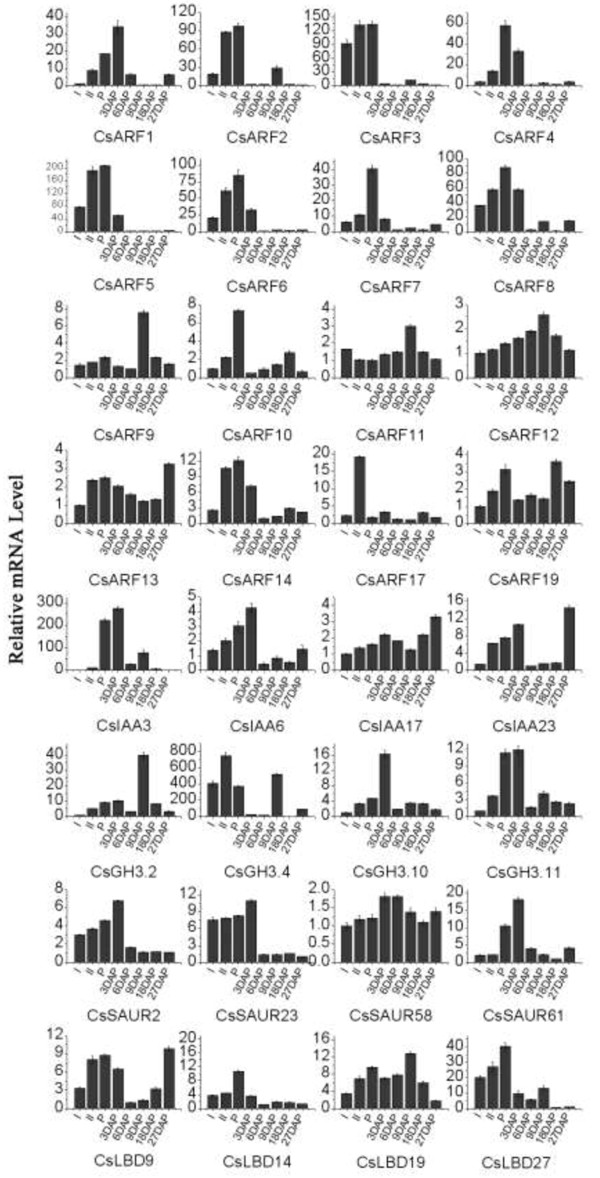
**Expression profiles of all the 32 cucumber auxin response genes during cucumber fruit development using qRT-PCR.** The ovary and fruits were sampled at eight stages, including two stages before pollination (Stages I and II: The ovary was approximately 0.3 cm and 1.5 cm in length, respectively), the pollination stage (P), and five fruit developmental stages [3, 6, 9, 18, and 27 days after pollination (DAP)]. For more details, see Figure 3.

During fruit development, 19 auxin responsive genes, including *CsARF1*-*8*, *CsARF10*, *CsARF14*, *CsARF19*, *CsIAA3*, *CsIAA6*, *CsGH3.10*, *CsGH3.11*, *CsSAUR2*, *CsSAUR23*, *CsSAUR61*, *CsLBD14*, and *CsLBD27* experienced mRNA accumulation during ovary or young fruit development. However, these genes showed a relatively low expression level during the subsequent fruit development (Figure 5). This result indicates that these genes mainly function in ovary or early fruit development. *CsARF9* and *CsGH3.2* were expressed mainly at 9 days after pollination (DAP). The relative mRNA level of *CsARF17* at 9 DAP was much higher than that at other stages (Figure 5). The three aforementioned genes might have stage-specific functions. *CsARF11*, *CsARF12*, *CsARF13*, *CsARF19*, *CsIAA17*, *CsIAA23*, *CsSAUR58 CsLBD9*, and *CsLBD58* showed relatively high expression levels at all selected stages (Figure 5), implying that they might be functioning during whole fruit development.

**Figure 6:**
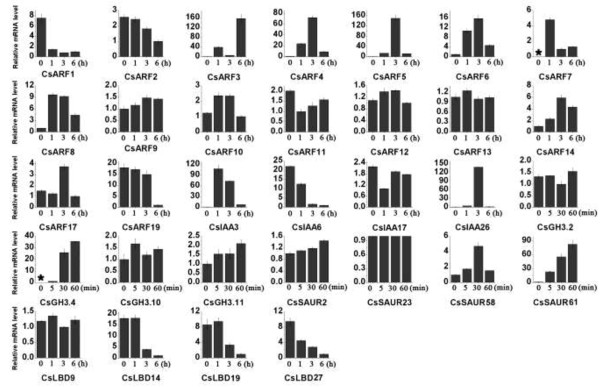
**Expression profiles of all 32 selected cucumber auxin response genes in response to IAA treatment.** QRT-PCR analyses were used to assess the transcript levels of these genes in the leaves sampled at 0, 5 min, 30 min, 1 h, 3 h, and 6 h after spraying 0.1 mM IAA in three-week-old tomato seedlings.

Although the ARFs and primary auxin response genes in *Arabidopsis*, rice, sorghum, and tomato are induced by exogenous auxin, they display differential expression patterns [18,19,23,26,27,57,58]. In cucumber, *CsARF3*-*8*, *CsARF14*, *CsIAA3*, *CsIAA26*, *CsGH3*.4, *CsSAUR58*, and *CsSAUR61* were up-regulated by over four-fold, whereas *CsARF1*, *CsARF19*, *CsIAA6*, *CsLBD14*, and *CsLBD27* were drastically down-regulated after IAA treatment in young leaves (Figure 6). Our promoter analysis revealed that two types of auxin-responsive elements, (AuxREs)-S00026 and -S000270, were identified in the promoter region of most of the primary auxin response genes, except in *CsIAA1*, *CsIAA3*, *CsIAA10*, *CsSAUR6*, *CsSAUR28*, *CsSAUR31*, *CsSAUR61*, *CsLBD17*, and *CsLBD24* (Additional file 4; Table S2). The diversity of numbers and locations of the auxin signaling transduction-related cis-elements may partially account for the different expression patterns of cucumber auxin response genes under IAA treatment. However, although none of the auxin signaling transductions-related cis-elements were found in the promoter regions of *CsIAA3* and *CsSAUR61* (Additional file 4: Tables S2-2 and 4), the mRNA levels of *CsIAA3* and *CsSAUR61* significantly increased after IAA treatment (Figure 6).

The mRNA levels of all five GH3 genes of Group II in *Arabidopsis* were up-regulated by exogenous auxin, suggesting that Group II-mediated auxin conjugation is a specific response to auxin application [40,59]. In the present study, the expression level of *CsGH3.4* belonging to Group II increased significantly after IAA treatment (Figure 6). However, the mRNA levels of *CsGH3.2* also belonging to group II showed no obvious change after IAA treatment (Figure 6). These results may reflect the functional divergence in the GH3 gene family between *Arabidopsis* and cucumber.

Increasing evidence proved that the auxin response genes are involved in stress/defense responses and that various environmental signals are integrated into changes in auxin homeostasis, redistribution, and signaling [60,61]. In the present study, promoter region analysis revealed that not only auxin-responsive elements (AuxREs) were found in the promoter regions of ARF, IAA, SAUR, GH3, and LBD family members (Additional file 4: Table S2). That is, many cis-elements in other signaling transduction pathways, such as drought-, salt-, and heat stress-related cis-elements, light signal transduction related cis-element, Ca^2+^-responsive cis-element, and calmodulin-binding/CGCG box, were also found. These results imply that these genes might function in connecting the auxin signaling transduction pathway with other signaling transduction pathways.

The GH3s were previously suggested to be linkers among the auxin, JA, and salicylic acid (SA) signal transduction pathways [62]. *AtGH3.11* and *AtGH3.10* are both members of group I. *AtGH3.11* can adenylate JA in vitro, but *AtGH3.10* shows no adenylation activity [21]. Many GH3 genes in *Arabidopsis*, soybean, and tobacco were found to be differentially expressed in various tissues in response to exogenous auxin and light stimuli [1,50,63]. In cucumber, all four selected GH3 genes were slightly induced by JA treatment (Additional file 5; Figure S3a). Only *GH3.11* mRNA was increased by approximately one-fold at 3 h after JA treatment. GH3 genes also affect SA signaling; for instance, GH3.5 in *Arabidopsis* was proposed to be a positive regulator of SA signaling [64,65]. Our research found that the expression level of *CsGH3.4* was up-regulated by more than twofold after SA treatment (Additional file 5; Figure S3b). Considering that *CsGH3.4* can also be up-regulated by auxin treatment (Figure 6), we suggest that *CsGH3.4* plays a specific role in the integration of auxin-SA signaling transduction pathways.

## Conclusion

Auxin controls a wide range of plant growth and development processes. In the present study, we carried out a genome-wide survey of auxin response-related gene including ARF, Aux/IAAs, GH3s, SAURs, and LBDs in cucumber (*Cucumis sativus* L*.*). Their gene structure, phylogenetic relationship, conserved motif, chromosomal location, promoter region and their expression profiles were also presented. Gene structure analysis revealed that most of the auxin-responsive genes had a conserved intron/exon structure, whereas some were more divergent, suggesting the possibility of functional diversification for these genes. Most of these genes possess auxin-responsive elements in their promoter region. Quantitative real-time RT-PCR analysis showed that the *CsARFs*, *CsAUX/IAAs*, *CsGH3s*, *CsSAURs*, and *CsLBDs* genes were expressed in at least one of the cucumber organs or tissues. However, different members of auxin-response genes displayed distinctive expression patterns in different cucumber organs and tissues. Furthermore, most of the detected auxin response genes were up-regulated during early fruit development. Some were expressed in a developmental stage-specific manner. Most tested genes were up-regulated by exogenous treatment with auxin, JA, or SA. However, the genes showed varying dynamic expression patterns. Our data imply that the auxin response genes may be involved in various vegetative and reproductive developmental processes and may have different functions during plant development. Characterization of selected members of these five families in cucumber is underway in our laboratory so that we can accurately determine the molecular basis of auxin regulation.

## Methods

### Searching for auxin response genes

To find previously identified and all potential auxin response genes in cucumber, we initially surveyed the cucumber Genomics Database (http://www.icugi.org/cgi-bin/ICuGI/genome/index.cgi?organism=cucumber) through TBLASTN using the protein sequences of the previously known auxin response genes as queries. The query consisted of 100 ARFs, 91 IAAs, 199 SAURs, and 49 GH3s sequences from *Arabidopsis*, rice, maize, sorghum, and tomato. Meanwhile, 42 AtLBDs and 36 SbLBD from *Arabidopsis* and sorghum were used for searching the LBD family genes. All predicted peptide sequences identified in this initial search were used as query in the BLASTP searches against the Cucumber Genomics Database and NCBI to find their potential functional domains, such as AUX_RESP (PF06507.5), Aux/IAA (PF02309.8), GH3 (PF03321.5), or DUF260 (PF03195.6). The Pfam 26.0 database was used to confirm the presence of auxin response-related domains in the predicted auxin response genes under a E-value level of 1.0 (http://pfam.sanger.ac.uk/). The genes without anticipant domains were removed. Based on the combined results from all of the performed searches, we identified all members of auxin response-related genes in the currently available cucumber genomic databases.

### Mapping auxin response genes on cucumber chromosomes

To determine the location of all auxin response genes on chromosomes, the nucleotide sequences of these genes were further used as query sequences for the BLASTN search against cucumber whole genome Scaffolds data (version 2) (http://www.icugi.org/cgi-bin/ICuGI/genome/blast.cgi?organism=cucumber&ver=2). Finally, the locations of all the cucumber auxin response genes were detected.

Subcellular localization prediction of each of these family genes was carried out using the CELLO v2.5 server (http://cello.life.nctu.edu.tw/) [66].

### Gene structure analysis, multiple-sequence alignments, and phylogenetic analysis

To detect the intron/exon structure, the coding sequences (CDS) of auxin response-related genes were aligned with their corresponding genomic sequences using spidey tool available on NCBI (http://www.ncbi.nlm.nih.gov/spidey/). The nature of the predicted protein such as PI and molecular weight were predicted by ProtParam tool available on Expert Protein Analysis System (ExPASy) proteomics server (http://web.expasy.org/protparam/). ClustalX v1.81 was used for multiple sequence alignments [67]. Phylogenetic relationship analysis was performed using MEGA 4.1 through the neighbor-joining method [68]. The Multiple Expectation Maximization for Motif Elicitation utility was employed to detect conserved motifs of cucumber auxin response family genes (http://meme.nbcr.net) [69].

To investigate cis-elements in the promoter sequences of cucumber auxin response genes, 2000 bp of genomic DNA sequences upstream of the initiation codon (ATG) were downloaded from the SGN database (Additional file 6: Figure S4). The PLACE website (http://www.dna.affrc.go.jp/PLACE/) was employed in the identification of *cis*-regulatory elements in the promoters [69].

### Plant growth conditions in relation to IAA, JA, and SA treatments

The cucumber (*Cucumis sativus* L*.* cv. Jianyan) plants used for expression analysis were grown in a growth chamber under 28°C/18°C (day/night) with a 16h photoperiod. The roots, stems, leaves, female flower buds (approximately 3 d before anthesis, excluding the ovary), male flower buds (approximately 1.0 cm in length), and ovaries (3 d before anthesis) were collected from flowering cucumber plants.

To analyze the expression patterns of cucumber auxin response genes at different developmental stages, cucumber ovaries or fruits were collected at the following eight developmental stages: ovary initiation stage (approximately 0.3 cm length, stage I), ovary elongating stage (approximately 1.5 cm length, 3 d before pollination, stage II), beginning of fruit development (0day after pollination, DAP, stage III), fruit early growing stage (3 DAP, stage IV), middle developmental stage (6 DAP, stage V), marketable maturing stage (9 DAP, stage VI), seed developmental stage (18 DAP, stage VII), and seed maturing stage (27 DAP, stage VIII, the fruits totally turned yellow). All flowers for each experiment were hand-pollinated on a single date.

Three-week-old cucumber seedlings with three fully opened leaves were sprayed with 0.1 mM IAA, 0.1 mM methyl JA (Sigma-Aldrich, WI, USA), or 1.5 mM SA on the seedling leaves. The plants were sampled at 0 min, 5 min, 30 min, 1 h, 3 h, and 6 h after auxin treatment and 0, 1 h and 3 h after JA and SA treatments. The experiment was repeated three times, and 15 seedlings were used in each treatment in each replication. All materials were stored at −80°C.

### Expression analysis of auxin response genes using qRT-PCR

Thirty-two auxin response genes belonging to five families were selected based on the phylogeny trees so that the expression profile of at least one gene of each branch in the phylogeny trees would be checked using qRT-PCR techniques. The primer pairs were listed in Additional file 7: Table S3 and the specificity of each primer to its corresponding gene was checked using the BLASTN program of the cucumber genome database. A sample of cDNA (1 μg) was subjected to RT-PCR in a final volume of 20 μl containing 12.5 μl SYBR Green Master Mix Reagent (Takara, Japan) and specific primers (3 pmol). Two biological and three technical replicates for each sample were performed in the RT-PCR machine (BIO-RAD CFX96, USA). To normalize the total amount of cDNA present in each reaction, the *EF1a* gene (accession number EF446145) was co-amplified as an endogenous control for the calibration of relative expression, The C_t_ method of relative gene quantification recommended by Applied Biosystems (PE Applied Biosystems, USA) was used to calculate the expression level of different treatments.

## Abbreviations

ARF: Auxin response factors; Aux/IAA: Auxin/indole-3-acetic acid; AuxRE: Auxinresponsive cis-element; DBD: DNA-binding domain; GH3: Gretchen Hagen 3; LBD: Lateral organ boundaries; MEME: Multiple Expectation Maximization for Motif Elicitation; NLS: Nuclear localization signal; QRT–PCR: Quantitative reverse transcription–PCR; SAUR: Small auxin up mRNA; SGN: Solanaceae Genomics Network; TAIR: The Arabidopsis Information Resource.

## Competing interests

The authors declare that they have no competing interests.

## Authors’ contributions

J W performed all the bioinformatics analysis and drafted the manuscript; LSY carried out the qRT-PCR analysis and promoter analysis; XG helped in bioinformatics analysis and data mining; LC finished the hormone treatment; YH helped in expression analysis; J W participated in data analysis and writing the paper; GL designed the study and drafted the manuscript. All authors read and approved the final manuscript.

## Supplementary Material

Additional file 1: Table S1 Summary of ARF, AUX/IAA, GH3, SAUR and LBD family genes in cucumber. Click here for file

Additional file 2: Figure S1 Phylogenetic relationships of ARF, AUX/IAA, GH3, SAUR and LBD gene families between cucumber and some other plant species. Click here for file

Additional file 3: Figure S2 Multiple sequence alignments of the full-length proteins of CsARF, CsAUX/IAA, CsGH3, CsSAUR and CsLBD in cucumber obtained with Clustal and manual correction. Click here for file

Additional file 4: Table S2 Cis-elements in the promoters of *CsARF, CsAUX/IAA, CsGH3, CsSAUR* and *CsLBD* genes in cucumber. Click here for file

Additional file 5: Figure S3 Expression profiles of four selected CsGH3 genes in response to JA and SA treatment. QRT-PCR analyses were used to assess the transcript levels of these genes in JA **(a)** and SA **(b)** treated plants. The leaves were sampled at 0 h, 1 h and 3 h after spraying 100 μM MeJA **(a)** and 1.5 mM SA, respectively, in 3-week tomato seedlings. Click here for file

Additional file 6: Figure S4 Promoter regions of *CsARF, CsAUX/IAA, CsGH3, CsSAUR* and *CsLBD* genes in cucumber. Click here for file

Additional file 7: Table S3 Primer sequences for qRT-PCR expression analysis. Click here for file

## References

[B1] Hagen G Guilfoyle T Auxin-responsive gene expression: genes, promoters and regulatory factors Plant Mol Biol 2002 49 373 385 10.1023/A:1015207114117 12036261

[B2] Guilfoyle TJ Ulmasov T Hagen G The ARF family of transcription factors and their role in plant hormone-responsive transcription. Cell Mol Life Sci 1998, 54:619–627.3 Liscum E, Reed JW. Genetics of Aux/IAA and ARF action in plant growth and development Plant Mol Biol 2002 49 387 400 10.1023/A:1015255030047 9711229 PMC11147363

[B3] Liscum E Reed JW Genetics of Aux/IAA and ARF action in plant growth and development Plant Mol Biol 2002 49 387 400 10.1023/A:1015255030047 12036262

[B4] Leyser O Molecular genetics of auxin signaling Ann Rev Plant Biology 2002 53 377 398 10.1146/annurev.arplant.53.100301.135227 12221981

[B5] Ulmasov T Hagen G Guilfoyle TJ ARF1, a transcription factor that binds to auxin response elements Science 1997 276 1865 1868 10.1126/science.276.5320.1865 9188533

[B6] Tiwari SB Hagen G Guilfoyle T The roles of auxin response factor domains in auxin-responsive transcription Plant Cell 2003 15 533 543 10.1105/tpc.008417 12566590 PMC141219

[B7] Berleth T Krogan NT Scarpella E Auxin signals–turning genes on and turning cells around Curr Opin Plant Biol 2004 7 553 563 10.1016/j.pbi.2004.07.016 15337098

[B8] Dharmasiri N Dharmasiri S Estelle M The F-box protein TIR1 is an auxin receptor Nature 2005 435 441 445 10.1038/nature03543 15917797

[B9] Guilfoyle TJ Hagen G Auxin response factor Curr Opin Plant Biol 2007 10 453 460 10.1016/j.pbi.2007.08.014 17900969

[B10] Ulmasov T Murfett J Hagen G Guilfoyle TJ Aux/IAA proteins repress expression of reporter genes containing natural and highly active synthetic auxin response elements Plant Cell 1997 9 1963 1971 10.1105/tpc.9.11.1963 9401121 PMC157050

[B11] Tiwari SB Wang XJ Hagen G Guilfoyle TJ AUX/IAA proteins are active repressors, and their stability and activity are modulated by auxin Plant Cell 2001 13 2809 2822 10.1105/tpc.13.12.2809 11752389 PMC139490

[B12] Tiwari SB Hagen G Guilfoyle TJ Aux/IAA proteins contain a potent transcriptional repression domain Plant Cell 2004 16 533 543 10.1105/tpc.017384 14742873 PMC341922

[B13] Szemenyei H Hannon M Long JA TOPLESS mediates auxin dependent transcriptional repression during *Arabidopsis* embryogenesis Science 2008 319 1384 10.1126/science.1151461 18258861

[B14] Kim J Harter K Theologis A Protein–protein interactions among the Aux/IAA proteins Proc Natl Acad Sci U S A 1997 94 1786 1791 10.1073/pnas.94.22.11786 PMC23574 9342315

[B15] Ouellet F Overvoorde P Theologis A IAA17/AXR3. Biochemical insight into an auxin mutant phenotype Plant Cell 2001 13 829 842 10.1105/tpc.13.4.829 11283339 PMC135541

[B16] Hardtke CS Ckurshumova W Vidaurre DP Singh SA Stamatiou G Tiwari SB Hagen G Guilfoyle TJ Berleth T Overlapping and non-redundant functions of the *Arabidopsis* auxin response factors MONOPTEROS and NONPHOTOTROPIC HYPOCOTYL 4 Development 2004 131 1089 1100 10.1242/dev.00925 14973283

[B17] Jain M Kaur N Tyagi AK Khurana JP The auxin-responsive GH3 gene family in rice (*Oryza sativa*) Funct Integr Genomics 2006 6 36 46 10.1007/s10142-005-0142-5 15856348

[B18] Jain M Tyagi AK Khurana PJ Genome-wide analysis, evolutionary expansion, and expression of early auxin-responsive SAUR gene family in rice (*Oryza sativa*) Genomics 2006 88 360 371 10.1016/j.ygeno.2006.04.008 16707243

[B19] Majer C Hochholdinger F Defining the boundaries: Structure and function of LOB domain proteins Trends Plant Sci 2011 16 47 52 20961800 10.1016/j.tplants.2010.09.009

[B20] Wang SK Bai YH Shen CJ Wu YR Zhang SN Jiang D Guilfoyle TJ Chen M Qi YH Auxin-related gene families in abiotic stress response in *Sorghum bicolor* Funct Integr Genomics 2010 10 533 546 10.1007/s10142-010-0174-3 20499123

[B21] Shuai B Reynaga-Pena CG Springer PS The lateral organ boundaries gene defines a novel plant-specific gene family Plant Physiol 2002 129 747 761 10.1104/pp.010926 12068116 PMC161698

[B22] Jain M Kaur N Garg R Thakur JK Tyagi AK Khurana JP Structure and expression analysis of early auxin-responsive Aux/IAA gene family in rice (*Oryza sativa*) Funct Integr Genomics 2006 6 47 59 10.1007/s10142-005-0005-0 16200395

[B23] Wang Y Deng D Bian Y Lv Y Xie Q Genome-wide analysis of primary auxin-responsive Aux/IAA gene family in maize (*Zea mays* L.) Mol Biol Rep 2010 37 3991 4001 10.1007/s11033-010-0058-6 20232157

[B24] Kalluri UC Difazio SP Brunner AM Tuskan GA Genome wide analysis of Aux/IAA and ARF gene families in *Populus trichocarpa* BMC Plant Biol 2007 7 1 14 10.1186/1471-2229-7-1 17986329 PMC2174922

[B25] Wu J Wang F Cheng L Kong F Peng Z Liu S Yu X Lu G Identification, isolation and expression analysis of auxin response factor (ARF) genes in *Solanum lycopersicum* Plant Cell Rep 2011 30 2059 2073 10.1007/s00299-011-1113-z 21735233

[B26] Wu J Peng Z Liu S He Y Cheng L Kong F Wang J Lu G Genome-wide analysis of Aux/IAA gene family in Solanaceae species using tomato as a model Mol Genet Genomics 2012 287 295 311 10.1007/s00438-012-0675-y 22314799

[B27] Ellis CM Nagpal P Jeffery YC Gretchen H Thomas J Jason RW AUXIN RESPONSE FACTOR1 and AUXIN RESPONSE FACTOR2 regulate senescence and floral organ abscission in *Arabidopsis thaliana* Development 2005 132 4563 4574 10.1242/dev.02012 16176952

[B28] Lim PO Lee IC Kim J Kim HJ Ryu JS Woo HR Nam HG Auxin response factor 2 (ARF2) plays a major role in regulating auxin-mediated leaf longevity J Exp Bot 2010 61 1419 1430 10.1093/jxb/erq010 20164142 PMC2837260

[B29] Rogg LE Lasswell J Bartel B A gain-of-function mutation in IAA28 suppresses lateral root development Plant Cell 2001 13 465 480 10.1105/tpc.13.3.465 11251090 PMC135515

[B30] Qi YH Wang SK Shen CJ Zhang SN Chen Y Xu YX Liu Y Wu YR Jiang DA OsARF12, a transcription activator on auxin response gene, regulates root elongation and affects iron accumulation in rice (*Oryza sativa*) New Phtol 2011 193 109 120 10.1111/j.1469-8137.2011.03910.x 21973088

[B31] Wang H Jones B Li Z Frasse P Delalande C Regad F Chaabouni S Latche A Pech JC Bouzayena M The tomato Aux/IAA transcription factor IAA9 is involved in fruit development and leaf morphogenesis Plant Cell 2005 17 2676 2692 10.1105/tpc.105.033415 16126837 PMC1242265

[B32] Guillon F Philippe S Bouchet B Devaux MF Frasse P Jones B Bouzayen M Lahaye M Down-regulation of an auxin response factor in the tomato induces modification of fine pectin structure and tissue architecture J Exp Bot 2008 61 1419 1430 10.1093/jxb/erm323 18267945

[B33] Jong MD Wolters-Arts M Feron R Mariani C Vriezen WH The *Solanum lycopersicum* auxin response factor 7 (SlARF7) regulates auxin signaling during tomato fruit set and development Plant J 2009 57 160 170 10.1111/j.1365-313X.2008.03671.x 18778404

[B34] Hendelman AK Buxdorf R Stav M Kravchik T Arazi inhibition of lamina outgrowth following *Solanum lycopersicum* AUXIN RESPONSE FACTOR 10 (SlARF10) derepression Plant Mol Biol 2012 78 56 576 10.1007/s11103-012-9883-4 22287097

[B35] Staswick PE Serban B Rowe M Tiryaki I Maldonado MT Maldonado MC Suza W Characterization of an Arabidopsis enzyme family that conjugates amino acids to indole-3-acetic acid Plant Cell 2005 17 616 627 10.1105/tpc.104.026690 15659623 PMC548830

[B36] Staswick PE Tiryaki I The oxylipin signal jasmonic acid is activated by an enzyme that conjugate it to isoleucine in Arabidopsis Plant Cell 2004 16 2117 2127 10.1105/tpc.104.023549 15258265 PMC519202

[B37] Meclure BA Guilfoyle TJ Characterization of a class of small auxin inducible soybean polyadenylated RNAs Plant Mol Biol 1987 9 611 662 10.1007/BF00020537 24277197

[B38] Gee MA Hagen G Guilfoyle TJ Tissue-specific and organ-specific expression of soybean auxin-responsive transcripts GH3 and SAURs Plant Cell 1991 3 419 430 10.1105/tpc.3.4.419 1840920 PMC160011

[B39] Knauss S Rohrmeier T Lehle L The auxin-induced maize gene *ZmSAUR2* encodes a short-lived nuclear protein expressed in elongating tissues J Biol Chem 2003 278 23936 23943 10.1074/jbc.M212585200 12695517

[B40] Spartz AK Lee SH Wenger JP Gonzalez N Itoh H Inze D Peer A Murphy AS Overvoorde PJ Gray WM The SAUR19 subfamily of SMALL AUXIN UP RNA genes promote cell expansion Plant J 2012 70 978 990 10.1111/j.1365-313X.2012.04946.x 22348445 PMC3481998

[B41] Kant S Bi YM Zhu T Rothstein SJ SAUR39, a small auxin-up RNA gene, acts as a negative regulator of auxin synthesis and transport in rice Plant Physiol 2009 151 691 701 10.1104/pp.109.143875 19700562 PMC2754634

[B42] Xu B Li Z Zhu Y Wang H Ma H Dong A Huang H *Arabidopsis* genes AS1, AS2, and JAG negatively regulate boundary-specifying genes to promote sepal and petal development Plant Physiol 2008 146 566 575 18156293 10.1104/pp.107.113787 PMC2245835

[B43] Lee HW Kim NY Lee DJ Kim J LBD18/ASL20 regulates lateral root formation in combination with LBD16/ASL18 downstream of ARF7 and ARF19 in *Arabidopsis* Plant Physiol 2009 151 1377 1389 10.1104/pp.109.143685 19717544 PMC2773067

[B44] Goh T Joi S Mimura T Fukaki H The establishment of asymmetry in *Arabidopsis* lateral root founder cells is regulated by LBD16/ASL18 and related LBD/ASL proteins Development 2012 139 883 893 10.1242/dev.071928 22278921

[B45] Soyano T Thitamadee S Machida Y Chua NH ASYMMETRIC LEAVES2-LIKE19/LATERAL ORGAN BOUNDARIES DOMAIN30 and ASL20/LBD18 regulate tracheary element differentiation in *Arabidopsis* Plant Cell 2008 20 3359 3373 10.1105/tpc.108.061796 19088331 PMC2630433

[B46] Saito Y Yamasaki S Fujii N Hagen G Guilfoyle T Takahashi H Isolation of cucumber CsARF cDNAs and expression of the corresponding mRNAs during gravity-regulated morphogenesis of cucumber seedlings J Exp Bot 2004 55 1315 1323 10.1093/jxb/erh144 15133054

[B47] Fujii N Kamada M Yamasaki S Takahshi H Differential accumulation of Aux/IAA mRNA during seedling development and gravity response in cucumber (*Cucumis sativus* L.) Plant Mol Bio 2000 42 731 740 10.1023/A:1006379804678 10809445

[B48] Huang S Li R Zhang Z Li L Gu X Fan W Lucas WJ Wang X Xie B Ni P Ren Y Zhu H Li J Lin K Jin W Fei Z Li G Staub J Kilian A van der Vossen EAG Wu Y Guo J He J Jia Z Ren Y Tian G Lu Y Ruan J Qian W Wang M The genome of the cucumber, *Cucumis sativus* L Nat Genet 2009 41 1275 1281 10.1038/ng.475 19881527

[B49] Ulmasov T Hagen G Guilfoyle TJ Activation and repression of transcription by auxin Proc Natl Acad Sci U S A 1999 96 5844 5849 10.1073/pnas.96.10.5844 10318972 PMC21948

[B50] Takase T Nakazawa M Ishikawa A Manabe K Matsui M DFL2, a new member of the *Arabidopsis GH3* gene family, is involved in red light-specific hypocotyl elongation Plant Cell Physiol 2003 44 1071 1080 10.1093/pcp/pcg130 14581632

[B51] Shen CJ Wang SK Bai YH Wu YR Zhang SN Chen M Guilfoyle TJ Wu P Qi YH Functional analysis of the structural domain of ARF proteins in rice (*Oryza sativa* L.) J Exp Bot 2010 61 3971 3981 10.1093/jxb/erq208 20693412 PMC2935870

[B52] Gray WM Kepinski S Rouse D Leyser D Estelle M Auxin regulates SCFTIR1-dependent degradation of AUX/IAA proteins Nature 2001 15 414 10.1038/35104500 11713520

[B53] Sato A Yamamoto KT Overexpression of the non-canonical Aux/IAA genes causes auxin-related aberrant phenotypes in *Arabidopsis* Physiol Plant 2008 133 397 405 10.1111/j.1399-3054.2008.01055.x 18298415

[B54] Audran-Delalande C Bassa C Mila I Regad F Zouine M Bouzayen M Genome-wide identification, functional analysis and expression profiling of Aux/IAA gene family in tomato Plant Cell Physiol 2012 53 659 672 10.1093/pcp/pcs022 22368074

[B55] Reed JW Roles and activities of Aux/IAA proteins in *Arabidopsis* Trends Plant Sci 2001 6 420 425 10.1016/S1360-1385(01)02042-8 11544131

[B56] Yamamoto KT Mori H Imaseki H cDNA cloning of indole-3-acetic acid-regulated genes: Aux22 and SAUR from mung bean (*Vigna radiata)* hypocotyl tissue Plant Cell Physiol 1992 33 93 97

[B57] Thakur JK Tyagi AK Khurana JP OsIAA1, an Aux/IAA cDNA from rice, and changes in its expression as influenced by auxin and light DNA Res 2001 8 193 203 10.1093/dnares/8.5.193 11759839

[B58] Paponov IA Paponov M Teale W Menges M Chakrabortee S Murray JA Palme K Comprehensive transcriptome analysis of auxin responses in *Arabidopsis* Mol Plant 2008 1 321 337 10.1093/mp/ssm021 19825543

[B59] Park JE Park JY Kim YS Staswick PE Jeon J Yun J Kim SY Kim J Lee YH Park CM GH3-mediated auxin homeostasis links growth regulation with stress adaptation response in *Arabidopsis* J Biol Chem 2007 282 10036 10046 10.1074/jbc.M610524200 17276977

[B60] Shibasaki K Uemura M Tsurumi S Rahman A Auxin response in *Arabidopsis* under cold stress: underlying molecular mechanisms Plant Cell 2009 21 3823 3838 10.1105/tpc.109.069906 20040541 PMC2814496

[B61] Wang H Tian C Duan J Wu K Research progresses on GH3s, one family of primary auxin-responsive genes Plant Growth Regul 2008 56 225 232 10.1007/s10725-008-9313-4

[B62] Tanaka S Mochizuki N Nagatani A Expression of the AtGH3a gene, an Arabidopsis homologue of the soybean GH3 gene, is regulated by phytochrome B Plant Cell Physiol 2002 43 281 289 10.1093/pcp/pcf033 11917082

[B63] Jagadeeswaran G Raina S Acharya BR Maqbool SB Mosher SL Appel HM Schultz JC Klessig DF Raina R *Arabidopsis* GH3-LIKE DEFENSE GENE 1 is required for accumulation of salicylic acid, activation of defense responses and resistance to *Pseudomonas syringae* Plant J 2007 51 234 246 10.1111/j.1365-313X.2007.03130.x 17521413

[B64] Nobuta K Okrent RA Stoutemyer M Rodibaugh N Kempema L Wildermuth MC Innes RW The GH3 acyl adenylase family member PBS3 regulates salicylic acid-dependent defense responses in *Arabidopsis* Plant Physiol 2007 144 1144 1156 10.1104/pp.107.097691 17468220 PMC1914169

[B65] Yu CS Lin CJ Hwang JK Predicting subcellular localization of proteins for Gram-negative bacteria by support vector machines based on n-peptide compositions Protein Sci 2004 13 1402 1406 10.1110/ps.03479604 15096640 PMC2286765

[B66] Hompson JD Gibson TJ Plewniak F Jeanmougin F Higgins DG The CLUSTAL_X windows interface: flexible strategies for multiple sequence alignment aided by quality analysis tools Nucleic Acids Res 1997 25 4876 4882 10.1093/nar/25.24.4876 9396791 PMC147148

[B67] Saitou N Nei M The Neighbor-joining method: a new method for reconstructing phylogenetic trees Mol Biol Evol 1987 4 406 425 3447015 10.1093/oxfordjournals.molbev.a040454

[B68] Bailey TL Boden M Buske FA Frith M Grant E Clementi L Ren J Li WW Noble WS MEME SUITE: tools for motif discovery and searching Nucleic Acids Res 2009 37 202 208 10.1093/nar/gkp335 PMC2703892 19458158

[B69] Higo K Ugawa Y Iwamoto M Korenaga T Plant cis-acting regulatory DNA elements (PLACE) database: 1999 Nucleic Acids Res 1999 27 297 300 10.1093/nar/27.1.297 9847208 PMC148163

