# Supplementary material for: Genome-wide identification and transcriptional profiling analysis of auxin response-related gene families in cucumber
Source: BMC Res Notes. 2014 Apr 8;7:218. doi: 10.1186/1756-0500-7-218 (PMC4108051; doi:10.1186/1756-0500-7-218)
Supplement: Additional file 1: Table S1 — Summary of ARF, AUX/IAA, GH3, SAUR and LBD family genes in cucumber. [file 1756-0500-7-218-S1.doc]

Additional file 1: Table S1. Primer sequences of cucumber Auxin response genes for Real-time PCR

| gene name | Forword(5’-3’) | Reverse(5’-3’) |
| --- | --- | --- |
| CsARF1 | GTAACGAGCCCAGATCCTC | CCCTGACATCAAACATCTTC |
| CsARF2 | TACGAAGGTCCACAAGCAG | CACCACCAAATTCAAACAG |
| CsARF3 | GCTCAGCTAACACTAGAAGC | CCCTGACATCAAACATCTTC |
| CsARF4 | GATCCCGATAAAGGATGG | GAAACCGCATCACAGAAG |
| CsARF5 | GTTGCTTAATGACCCAAGAG | CAGCTAACAAATTCCTCCC |
| CsARF6 | CAAGGCAGACCCAACCAC | ACGGTGACTGGGATTGAG |
| CsARF7 | GCTGGTGAACGACCTAATC | GTCATCTGGCATCCCTTG |
| CsARF8 | CGCTCACCCAAACTCTATG | TGGGCACATTAGAAGGAAG |
| CsARF9 | AGAACTTCGTCCTCGTAAC | AACCCATCTTCACATCCTG |
| CsARF10 | TTGATCTTTCATCCCTCAG | CATGCTTTGTAGCACCTAATG |
| CsARF11 | TACCCAGACCTATTTCGTTG | GCATCCCCACTTCATAACC |
| CsARF12 | AGGGTTATCAAGCCACTG | GACCGAAAGGTTGAGTGTC |
| CsARF13 | ATGGCACATTCTACTCAAG | TTCTGTAAGTATGGGTCGG |
| CsARF14 | CCATCATTTCACCCTTGC | GGGATTGTTTCGAGGAAG |
| CsARF17 | ATCTATACGAGTCCTCCTGC | TGACCGTTTACGCTTCTTG |
| CsARF19 | AATGTCGGACAATCAGGTTC | AATTGGACGAGTTGAGAAGG |
| CsGH3.2 | AAACGCAGTGAAGAACTCG | CACGTAGTGACCTGGAATTG |
| CsGH3.4 | GCGAGAAAGATGCCAAAG | ACCGGTTGACATATTCAGTG |
| CsGH3.10 | GAATGTGTTGCTCAGAAATG | TTTCCACAATTCGAAGCTC |
| CsGH3.11 | GACGCAGTGAAAGTAATGG | TTCTCCGTGTTCTTGTCG |
| CsIAA3 | AAACCTCCCAACCTCCTC | GCTTACTTTCACGTACCCTC |
| CsIAA6 | AAGAACGCCGTCATAAGC | GCTACCGTACATCTTCAAGTC |
| CsIAA17 | GCCAACACCTCAGATGAC | ATCCCTTCCTTTACCAGAC |
| CsIAA26 | TTCTGTTCCAGATCCCAC | AAGCATCTTCAAAGCCAC |
| CsSAUR2 | AGAATAGTATCAAGGTGGTCG | CCCTTCTTTCACATCCTCC |
| CsSAUR23 | GGCAGAGGAAGAATACGG | TGGTGTTGTTGTTGGTGG |
| CsSAUR58 | ATCTCACTGCCAAGAACAAG | CCCAAATACCTTATGGGC |
| CsSAUR61 | CGCACAGTCTATGTTGGTAAG | AACCTCACAAGAAACCACC |
| CsLBD9 | TTGTTGCCAAATCATCCC | CCTAGTTGCGAAATAGCC |
| CsLBD14 | GCCAAGTGAATTAGCTCTG | TTCGGCTTCTATCTTTCG |
| CsLBD19 | GGATACTATTCTCAACGGC | GATGAATCATCAAGGTTAGG |
| CsLBD27 | GGCCAGGCTTAGAGATCC | TATTAGCGTAACGAGCGAG |
| EF1a | ACTGTGCTGTCCTCATTATTG | AGGGTGAAAGCAAGAAGAGC |
