# Supplementary material for: Genome-wide identification and transcriptional profiling analysis of auxin response-related gene families in cucumber
Source: BMC Res Notes. 2014 Apr 8;7:218. doi: 10.1186/1756-0500-7-218 (PMC4108051; doi:10.1186/1756-0500-7-218)

Additional file 2: Figure 1**. Phylogenetic relationship of Auxin-response genes in different species**

Suppl Fig 3 -1 Phylogenetic relationships among tomato, rice, maize, Sorghum, cucumber and Arabidopsis ARF proteins. The unrooted tree was generated using MEGA4.1 program by the neighbor-joining method. Bootstrap values from 1,000 replicates are indicated at each branch. Triplets were colored in green, sister pairs were colored in yellow, branches which contain members with very strong bootstrap were colored in red.


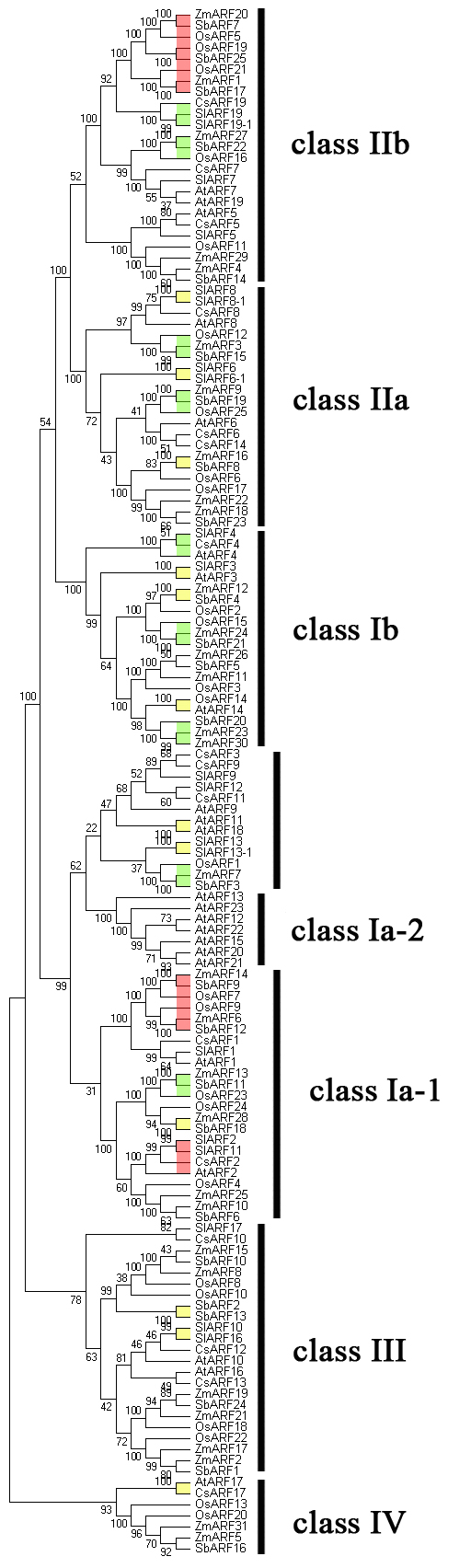


Suppl Fig 3 -2 Phylogenetic relationships among rice, maize, Sorghum, cucumber and Arabidopsis AUX/IAA proteins. The unrooted tree was generated using MEGA4.1 program by the neighbor-joining method. Bootstrap values from 1,000 replicates are indicated at each branch. Sister pairs were colored in yellow.


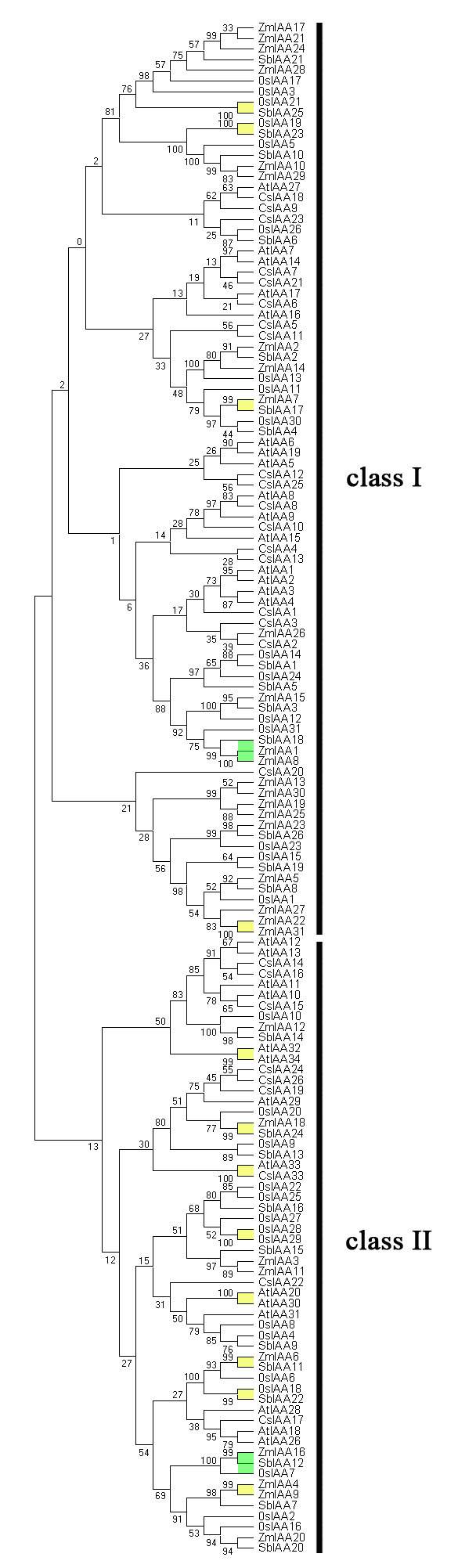


Suppl Fig 3 -3 Phylogenetic relationships among rice, sorghum, cucumber and Arabidopsis GH3 proteins. The unrooted tree was generated using MEGA4.1 program by the neighbor-joining method. Bootstrap values from 1,000 replicates are indicated at each branch. Triplets were colored in green, sister pairs were colored in yellow.


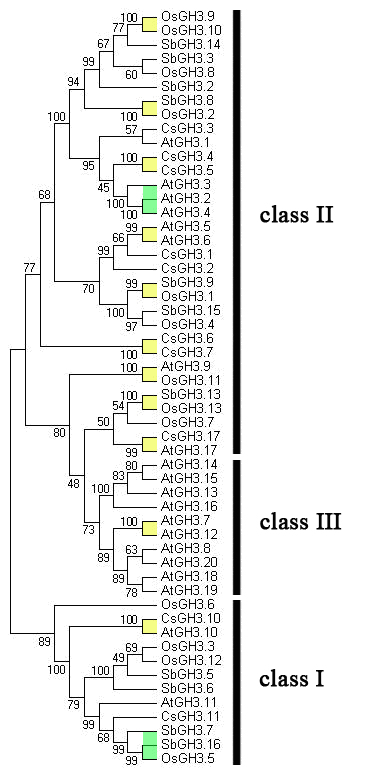


Suppl Fig 3 -4 Phylogenetic relationships among cucumber and Arabidopsis SAUR proteins. The unrooted tree was generated using MEGA4.1 program by the neighbor-joining method. Bootstrap values from 1,000 replicates are indicated at each branch. Sister pairs were colored in yellow, clusters were colored in blue.


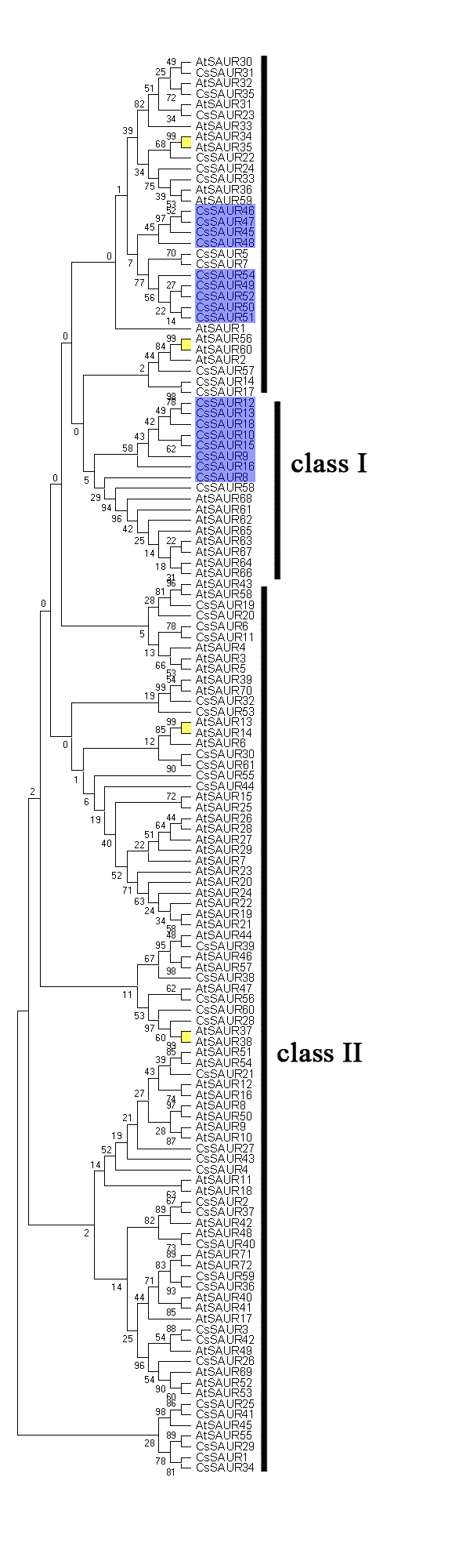


Suppl Fig 3 -5 Phylogenetic relationships among cucumber and Arabidopsis LBD proteins. The unrooted tree was generated using MEGA4.1 program by the neighbor-joining method. Bootstrap values from 1,000 replicates are indicated at each branch. Sister pairs were colored in yellow.


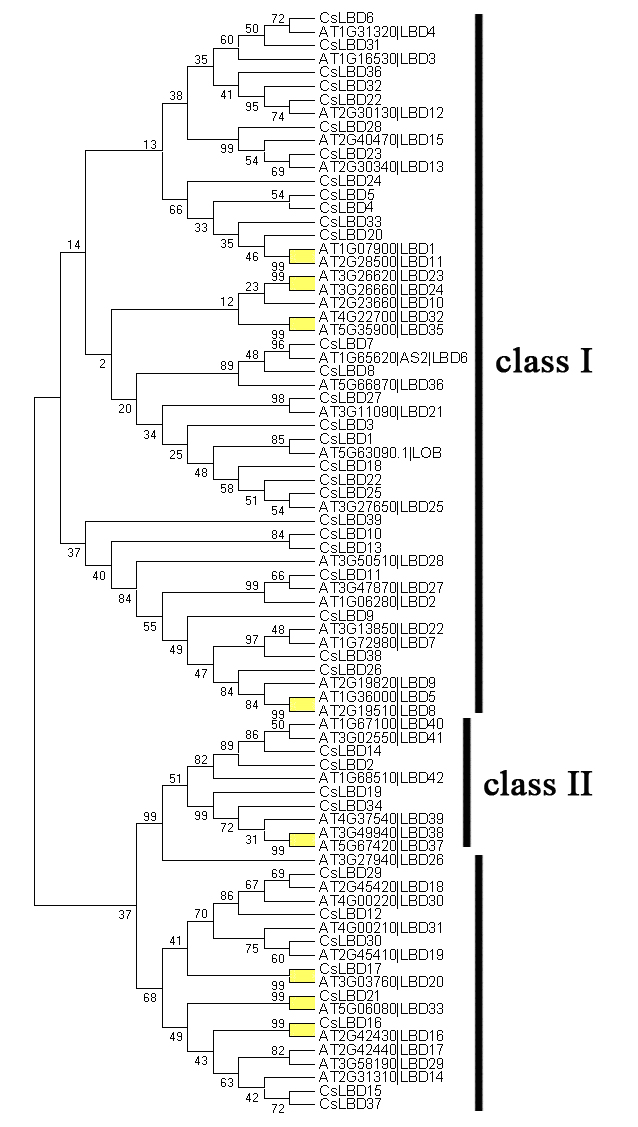

Supplement: Additional file 2: Figure S1 — Phylogenetic relationships of ARF, AUX/IAA, GH3, SAUR and LBD gene families between cucumber and some other plant species. [file 1756-0500-7-218-S2.doc]
