# Supplementary material for: Genome-wide identification and transcriptional profiling analysis of auxin response-related gene families in cucumber
Source: BMC Res Notes. 2014 Apr 8;7:218. doi: 10.1186/1756-0500-7-218 (PMC4108051; doi:10.1186/1756-0500-7-218)

**Additional file 3: Figure S2. Multiple sequence alignments**

**Additional file 3: Figure** **2-1** a Alignment profile of cucumber ARF proteins obtained with the ClustalX program. The height of the bars indicates the number of identical residues per position. The shaded regions indicate the high sequence similarity among DBDs regions. Motifs III and IV are consensus sequences shared by Aux/IAA proteins. b Multiple alignments of Motifs III and IV in cucumber ARF proteins obtained with ClustalX. Black and light gray shading indicate identical and conversed amino acid residues, respectively. Conserved domains are also underlined and correspond to part a


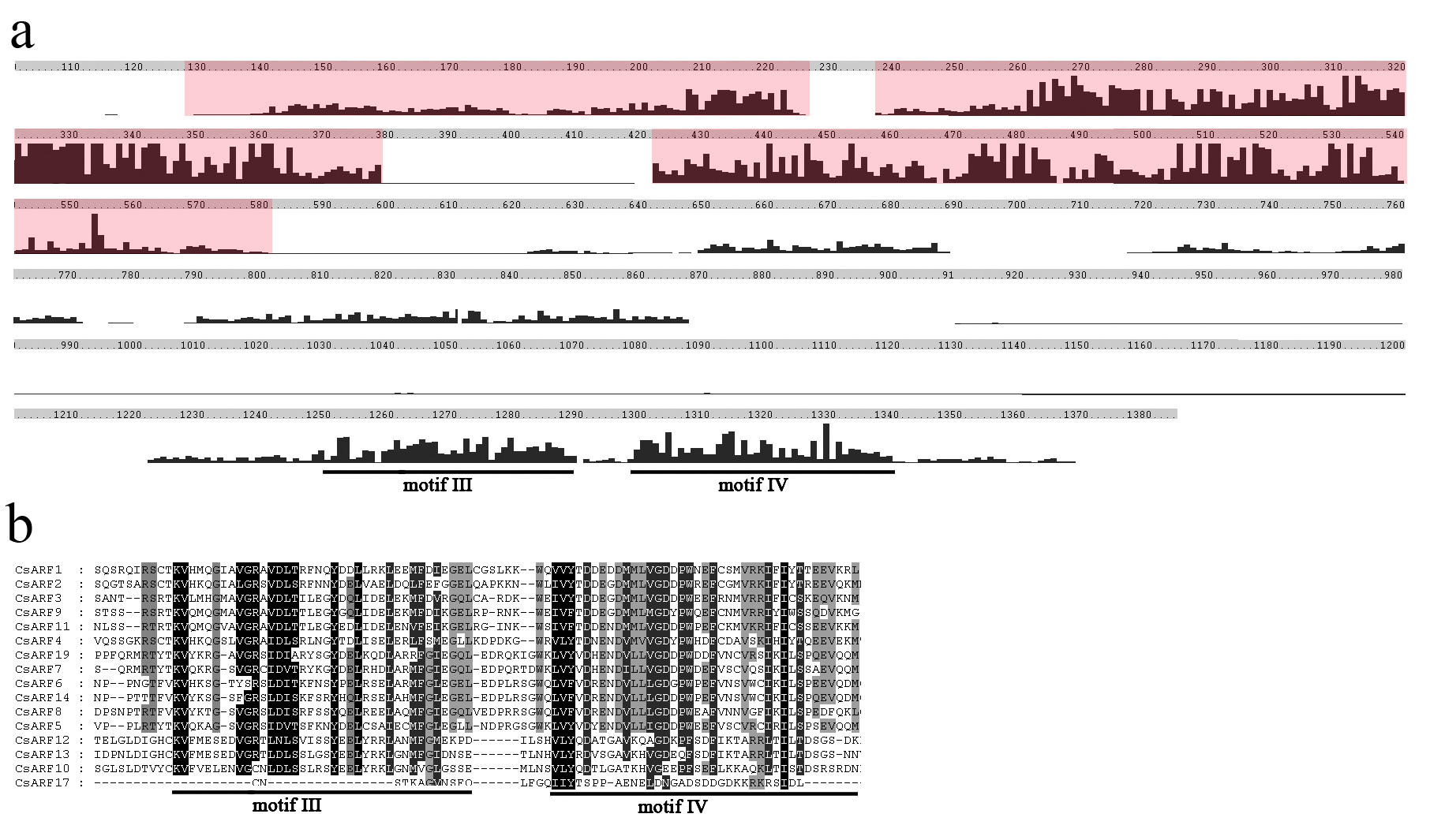


**Additional file 3: Figure 2** a Alignment of cucumber Aux/IAA proteins obtained with the ClustalX program. The height of the bars indicates the number of identical residues per position. b Multiple alignments of the domains I–IV of the cucumber Aux/IAA proteins obtained with ClustalX and manual correction. Black and light gray shading indicates identical and conversed amino acid residues, respectively. Conserved domains are also underlined and correspond to part (a). The LxLxLx and LxLxLxLxLx motif were also marked


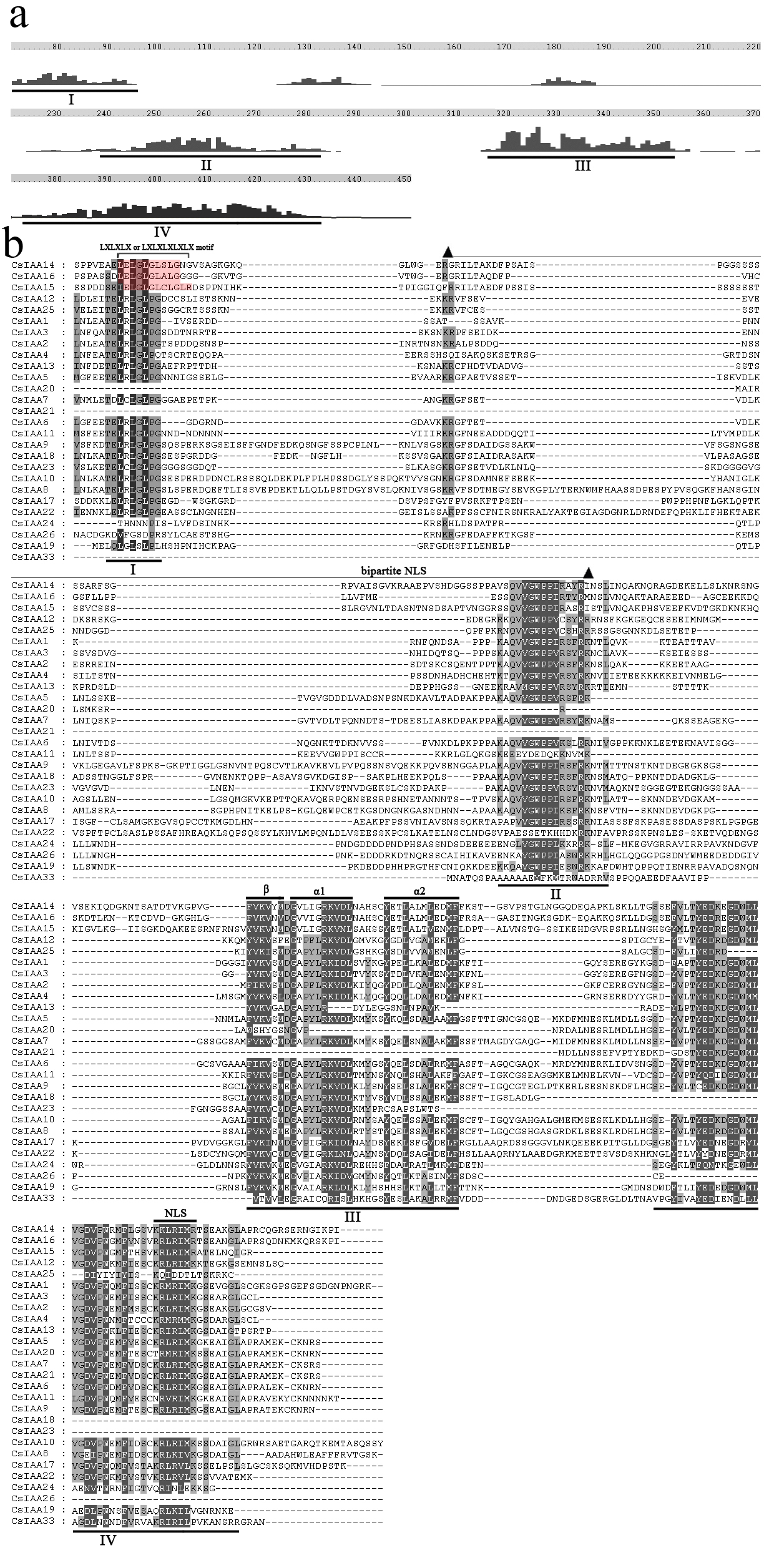


**Additional file 3: Figure 2-3** a Alignment of cucumber GH3 proteins obtained with the ClustalX program. The height of the bars indicates the number of identical residues per position. b Black and light gray shading indicates identical and conversed amino acid residues, respectively.


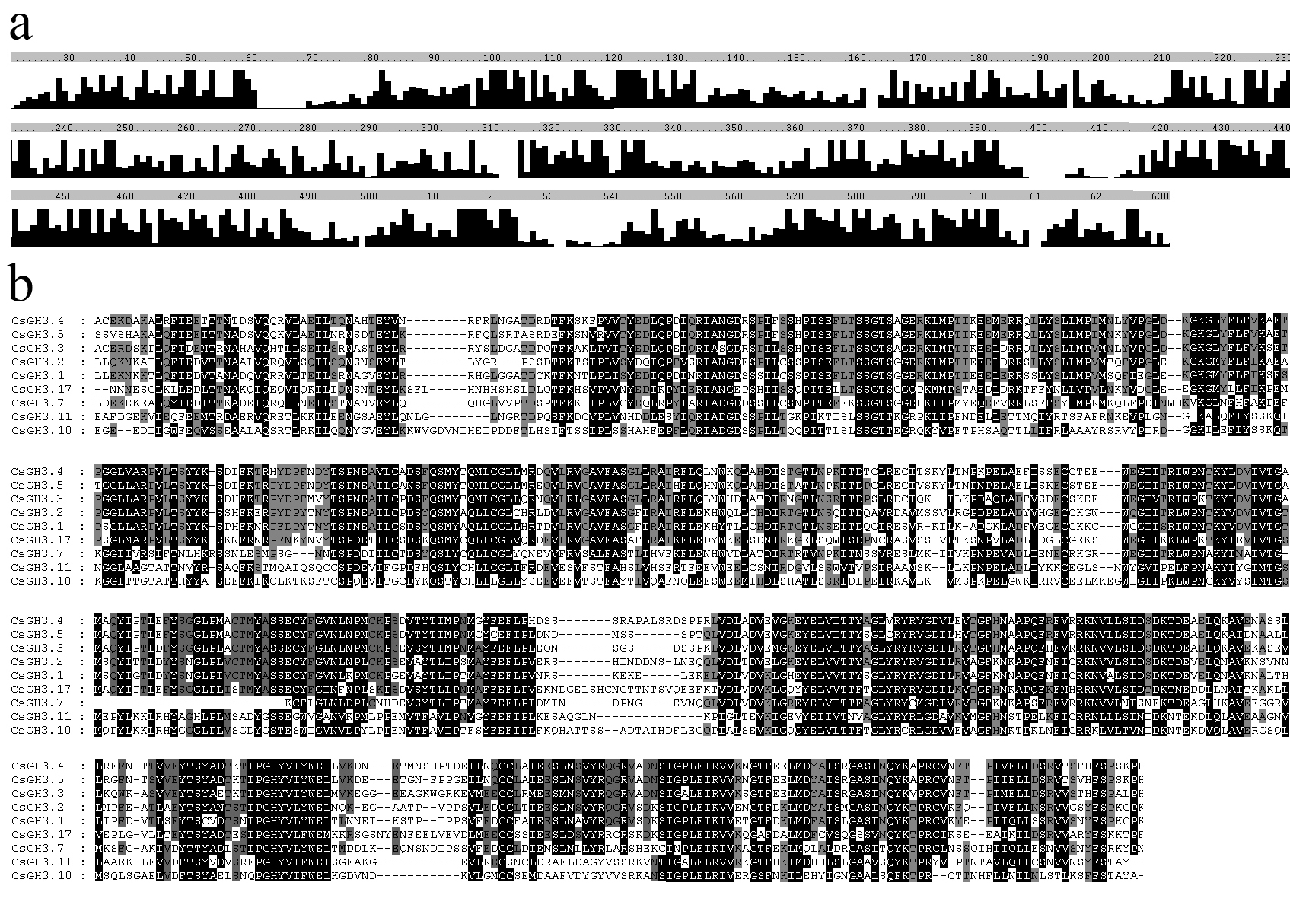


**Additional file 3: Figure 2-4** The five conserved consensus motifs in SAUR of cucumber SAUR proteins were found by MEME. The symbol heights represent the relative frequency of each residue. The numbers of sites and e-value for each motif are also shown.


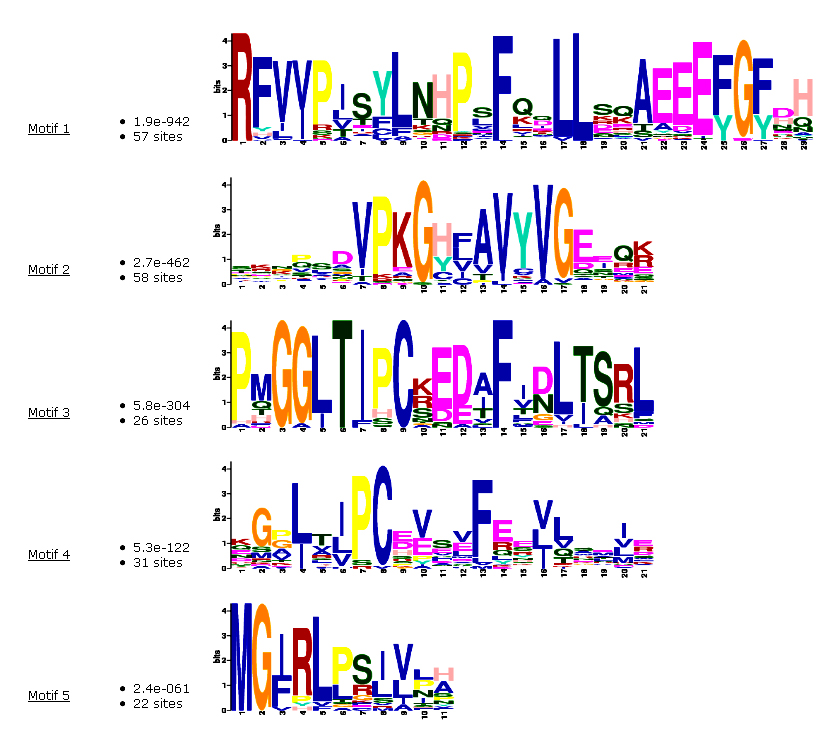


**Additional file 3: Figure 2**-**5**. a Alignment of cucumber LBD proteins obtained with the ClustalX program. The height of the bars indicates the number of identical residues per position. b Multiple sequence alignment of LOB (DUF260) domain of CsLBD proteins. Conserved region of proteins are separated by three blocks, C block, GAS block and coiled-coil region.


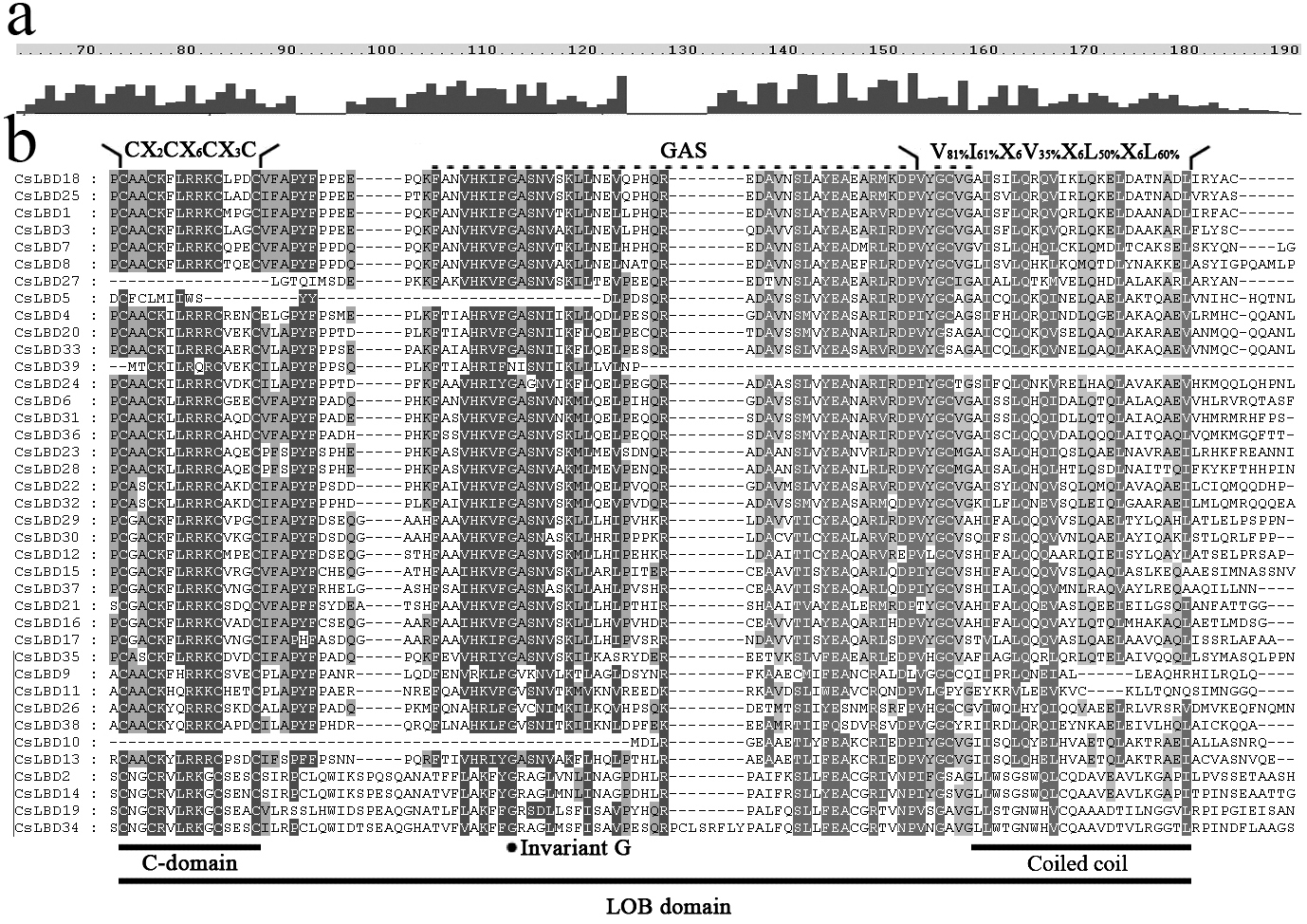

Supplement: Additional file 3: Figure S2 — Multiple sequence alignments of the full-length proteins of CsARF, CsAUX/IAA, CsGH3, CsSAUR and CsLBD in cucumber obtained with Clustal and manual correction. [file 1756-0500-7-218-S3.doc]
