# Supplementary figures and images for: Genome-wide identification and transcriptional profiling analysis of auxin response-related gene families in cucumber
Source: BMC Res Notes. 2014 Apr 8;7:218. doi: 10.1186/1756-0500-7-218 (PMC4108051; doi:10.1186/1756-0500-7-218)

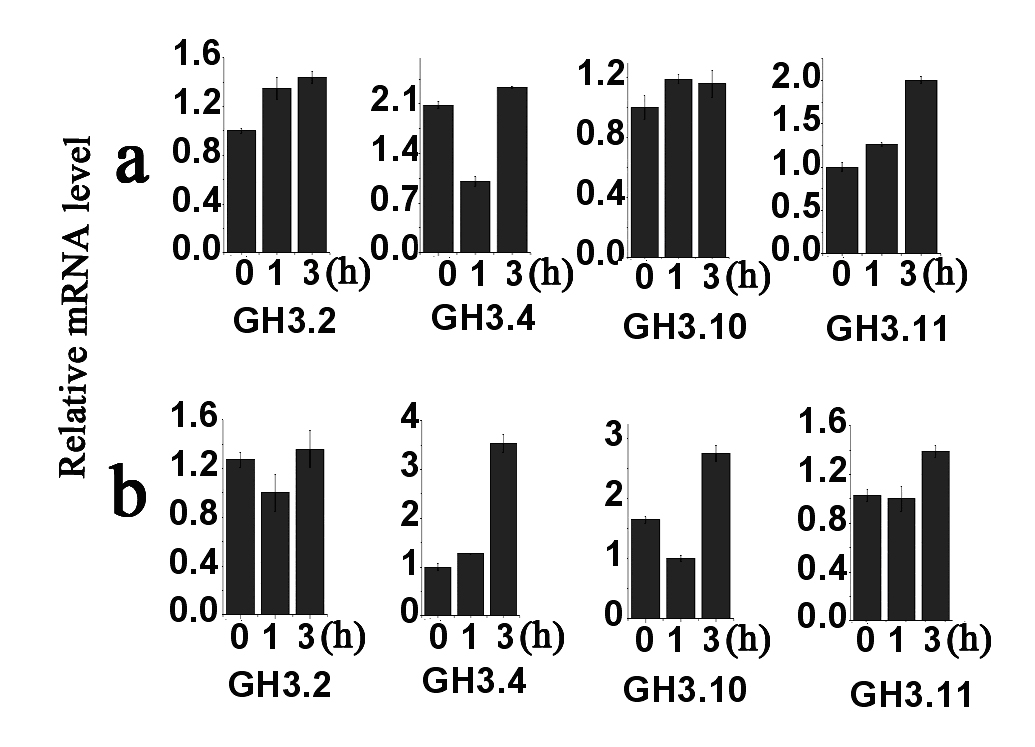

Supplement: Additional file 5: Figure S3 — Expression profiles of four selected CsGH3 genes in response to JA and SA treatment. QRT-PCR analyses were used to assess the transcript levels of these genes in JA (a) and SA (b) treated plants. The leaves were sampled at 0 h, 1 h and 3 h after spraying 100 μM MeJA (a) and 1.5 mM SA, respectively, in 3-week tomato seedlings. [file 1756-0500-7-218-S5.jpeg]
