# Supplementary material for: Genome-wide identification and transcriptional profiling analysis of auxin response-related gene families in cucumber
Source: BMC Res Notes. 2014 Apr 8;7:218. doi: 10.1186/1756-0500-7-218 (PMC4108051; doi:10.1186/1756-0500-7-218)
Supplement: Additional file 6: Figure S4 — Promoter regions of CsARF, CsAUX/IAA, CsGH3, CsSAUR and CsLBD genes in cucumber. [file 1756-0500-7-218-S6.doc]

**Table S2 Summary of ARF, AUX/IAA, GH3, SAUR and LBD family genes in cucumber**

**Table S2-1. ARF gene family in** cucumber

| Gene | Generic name | Predicted protein (aa) | Deduced polypeptide | | | Predicted CDS ID | Subcellular localization | Unigene or est | Intron | Chromosome number | Strand direction | Location |
| --- | --- | --- | --- | --- | --- | --- | --- | --- | --- | --- | --- | --- |
|  |  |  | Molecular weight (kDa) | PI | Domain |  |  |  |  |  |  |  |
| CsARF1 |  | 677 | 75.5 | 6.05 | DBD MR CTD | Csa010564 | Nuclear | CU123278 CU143420^P^ | 13 | 5 | - | 2352370-2361243 |
| CsARF2 |  | 841 | 93.4 | 6.22 | DBD MR CTD | Csa005276 | Nuclear | CU096172 CU110991^P^ | 13 | 1 | + | 53872-58231 |
| CsARF3 |  | 680 | 75.7 | 6.02 | DBD MR CTD | Csa012805 | Nuclear | CU103353 CU131644^P^ | 13 | 5 | + | 4432271-4438002 |
| CsARF4 | CsARF5 | 733 | 81.1 | 6.81 | DBD MR CTD | Csa020090 | Nuclear | CU096027^C^ | 12 | 6 | - | 14087464-14093910 |
| CsARF5 |  | 1017 | 112.9 | 5.59 | DBD MR CTD | Csa017897 | Nuclear | CU104338 CU175800^P^ | 17 | 3 | - | 36270368-36275514 |
| CsARF6 | CsARF4 | 816 | 90.8 | 6.03 | DBD MR CTD | Csa021954 | Nuclear | CU108219^C^ | 11 | 1 | - | 19276890-19281043 |
| CsARF7 | CsARF1 | 1081 | 120.2 | 5.97 | DBD MR CTD | Csa009210 | Nuclear | CU101380^C^ | 11 | 2 | - | 18593-33432 |
| CsARF8 |  | 647 | 71.6 | 5.88 | DBD MR CTD | Csa019265 | Nuclear | CU112849^P^ | 9 | 5 | - | 12784412-12794654 |
| CsARF9 |  | 616 | 69.0 | 7.68 | DBD MR CTD | Csa019361 | Nuclear | CU100116^P^ | 11 | 4 | + | 4475311-4478267 |
| CsARF10 |  | 703 | 77.6 | 6.46 | DBD MR CTD | Csa020560 | Nuclear | CU161135 CU148288 CU127105^P^ | 2 | 6 | - | 18334038-18336533 |
| CsARF11 |  | 693 | 77.8 | 5.76 | DBD MR CTD | Csa007296 | Nuclear | CU113581^P^ | 12 | 7 | - | 11622142-11625676 |
| CsARF12 |  | 716 | 79.0 | 8.75 | DBD MR CTD | Csa015176 | Nuclear | CU096785 CU112744^P^ | 3 | 6 | - | 10069492-10072346 |
| CsARF13 |  | 694 | 76.1 | 6.33 | DBD MR CTD | Csa011935 | Nuclear | CU161689 CU134357^P^ | 2 | 6 | + | 20786599-20789036 |
| CsARF14 | CsARF3 | 916 | 101.9 | 6.18 | DBD MR CTD | NA | Nuclear | CU105174^C^ | 13 | 6 | - | 28272966-28277970 |
| CsARF17 |  | 549 | 60.2 | 6.24 | DBD MR | Csa022361 | Nuclear | CU110236^P^ | 1 | 2 | + | 14787528-14789826 |
| CsARF19 | CsARF2 | 1107 | 121.9 | 6.10 | DBD MR CTD | Csa012237 | Nuclear | CU103918^C^ | 13 | 2 | + | 5286910-5293852 |

**C and P: The unigenes containing the whole or partial ORF of relevant CsARFs, respectively.**

**Table S2-2. IAA gene family in cucumber**

| Gene | Generic name | Predicted protein (aa) | Deduced polypeptide | | | Predicted CDS ID | Subcellular localization | Unigene or est | Intron | Chromosome number | Strand direction | Location |
| --- | --- | --- | --- | --- | --- | --- | --- | --- | --- | --- | --- | --- |
|  |  |  | Molecular weight (kDa) | PI | Domain |  |  |  |  |  |  |  |
| CsIAA1 |  | 203 | 22.3 | 7.68 | I II III IV | Csa021321 | Nuclear | CU105549 CU091868 CU110764 CU098573^P^ | 4 | 2 | + | 10523366-10524341 |
| CsIAA2 | CsIAA3 | 197 | 22.0 | 6.41 | I II III IV | Csa006680 | Nuclear | CU108001^C^ | 3 | 2 | + | 2007336-2008389 |
| CsIAA3 |  | 196 | 21.8 | 6.74 | I II III IV | Csa016714 | Nuclear | CU099538^C^ | 3 | 7 | - | 13973767-13974930 |
| CsIAA4 |  | 222 | 25.7 | 8.46 | I II III IV | Csa003118 | Nuclear | CU112382 CU123003 CU109332^P^ | 3 | 5 | - | 6374148-6375825 |
| CsIAA5 |  | 230 | 25.4 | 8.28 | I II III IV | Csa016715 | Nuclear | CU108398^C^ | 4 | 7 | - | 13954294-13956513 |
| CsIAA6 |  | 236 | 25.9 | 9.03 | I II III IV | Csa010933 | Nuclear | CU107868^C^ | 4 | 1 | + | 27692784-27695296 |
| CsIAA7 |  | 237 | 25.9 | 5.76 | I II III IV | Csa000125 | Nuclear | CU097123^C^ | 4 | 3 | - | 9474497-9476455 |
| CsIAA8 |  | 404 | 44.3 | 6.39 | I II III IV | Csa012115 | Nuclear | CU102278 CU096599^P^ | 5 | 6 | - | 24432911-24441612 |
| CsIAA9 |  | 356 | 38.4 | 7.46 | I II III IV | Csa009839 | Nuclear | CU107408^C^ | 4 | 7 | + | 17858020-17860371 |
| CsIAA10 | CsIAA2 | 427 | 47.5 | 5.86 | I II III IV | Csa018571 | Nuclear | CU084765^C^ | 7 | 2 | + | 18484801-18489177 |
| CsIAA11 |  | 222 | 25.3 | 6.63 | I III IV | Csa025885 | Nuclear | CU141235^P^ | 4 | 2 | - | 10948961-10951412 |
| CsIAA12 | CsIAA1 | 188 | 21.3 | 8.45 | I II III IV | Csa020459 | Nuclear | CU092895^C^ | 1 | 3 | - | 21475249-21476183 |
| CsIAA13 |  | 184 | 20.4 | 5.15 | I II III IV | Csa020481 | Nuclear | NA | 4 | 1 | + | 14747630-14748914 |
| CsIAA14 |  | 321 | 33.9 | 8.73 | I II III IV | Csa000184 | Chloroplast | CU085350 CU169848^P^ | 4 | 3 | - | 8911580-8913576 |
| CsIAA15 |  | 281 | 30.5 | 7.07 | I II III IV | Csa001390 | Nuclear | CU121762 CU123964^P^ | 3 | 3 | + | 23687801-23692415 |
| CsIAA16 |  | 285 | 30.8 | 6.01 | I II III IV | Csa016993 | Chloroplast | CU137205^P^ | 4 | 2 | - | 9657165-9658996 |
| CsIAA17 |  | 326 | 35.1 | 6.85 | I II III IV | Csa021916 | Nuclear | CU094506^P^ | 4 | 3 | - | 20565887-20568448 |
| CsIAA18 |  | 233 | 24.6 | 7.78 | I II III | Csa021532 | Nuclear | CU108318^C^ | 1 | 1 | - | 11632576-11633848 |
| CsIAA19 |  | 207 | 23.9 | 6.25 | I II III IV | Csa006091 | Nuclear | NA | 3 | 2 | - | 19637100-19638146 |
| CsIAA20 |  | 94 | 10.9 | 7.80 | IV | Csa021533 | Mitochondrial | CU108318^P^ | 3 | 1 | - | 11631185-11632424 |
| CsIAA21 |  | 70 | 8.0 | 4.54 | IV | Csa021313 | Nuclear | CU141493^P^ | 2 | 2 | - | 10496540-10496942 |
| CsIAA22 |  | 342 | 38.0 | 6.76 | I III IV | Csa002198 | Nuclear | CU088110 CU100895 CU123385 CU132531^P^ | 3 | 3 | - | 38480318-38482711 |
| CsIAA23 |  | 179 | 18.4 | 9.05 | I II III | Csa021312 | Nuclear | NA | 2 | 2 | - | 10497038-10498072 |
| CsIAA24 |  | 218 | 25.0 | 7.08 | II III IV | Csa020973 | Nuclear | CU094186 CU122609^C^ | 3 | 1 | + | 12120546-12121494 |
| CsIAA25 |  | 160 | 17.7 | 7.59 | I II III IV | Csa020458 | Nuclear | NA | 0 | 3 | - | 21481071-21481485 |
| CsIAA26 |  | 187 | 21.2 | 5.77 | II III | Csa002030 | Nuclear | CU097208^C^ | 1 | 3 | + | 36797124-36798219 |
| CsIAA33 |  | 144 | 16.0 | 5.41 | III IV | Csa022634 | Nuclear | CU151620^P^ | 1 | 2 | - | 4434876-4436988 |

C and P: The unigenes containing the whole or partial ORF of relevant CsARFs, respectively.

NA no available

**Table S2-3. GH3 gene family in** cucumber

| Gene | Predicted protein (aa) | Deduced polypeptide | | Predicted CDS ID | Subcellular localization | Unigene or est | Intron | Chromosome number | Strand direction | Location |
| --- | --- | --- | --- | --- | --- | --- | --- | --- | --- | --- |
|  |  | Molecular weight (kDa) | PI |  |  |  |  |  |  |  |
| CsGH3.1 | 602 | 68.0 | 6.32 | Csa019599 | PlasmaMembrane | NA | 2 | 3 | - | 20356352-20359760 |
| CsGH3.2 | 604 | 68.0 | 5.67 | Csa020596 | PlasmaMembrane | CU144092^P^ | 2 | 3 | - | 4620719-4622720 |
| CsGH3.3 | 599 | 67.7 | 5.98 | Csa008779 | Cytoplasmic | CU097223 CU117970 CU120462 CU090551 CU169871^P^ | 2 | 3 | - | 13606127-13608128 |
| CsGH3.4 | 607 | 68.7 | 5.51 | Csa010152 | PlasmaMembrane | CU168769 CU163278^P^ | 2 | 6 | + | 23754397-23756416 |
| CsGH3.5 | 588 | 66.4 | 5.64 | Csa010151 | PlasmaMembrane | NA | 2 | 6 | + | 23735350-23737326 |
| CsGH3.6 | 598 | 67.7 | 6.66 | Csa006255 | PlasmaMembrane | NA | 2 | 2 | - | 6678628-6683496 |
| CsGH3.7 | 571 | 65.4 | 5.93 | Csa007627 | Nuclear | CU161234^P^ | 3 | 6 | + | 8671537-8674424 |
| CsGH3.10 | 589 | 66.4 | 6.27 | Csa007733 | PlasmaMembrane | CU158443^P^ | 3 | 6 | - | 8559337-8562525 |
| CsGH3.11 | 568 | 63.5 | 6.02 | Csa000346 | Cytoplasmic | CU097359 CU158708 CU128053 CU131530 CU151162^P^ | 3 | 3 | - | 6877664-6880061 |
| CsGH3.17 | 611 | 69.6 | 5.80 | Csa003015 | Nuclear | CU131263 CU125711^P^ | 3 | 4 | - | 1229695-1231885 |

p The unigenes which only contain the partial ORF of relevant CsARFs

NA no available

**Table S2-4. SAUR gene family in** cucumber

| Gene | Predicted protein (aa) | Deduced polypeptide | | Predicted CDS ID | Subcellular localization | Unigene or est | Intron | Chromosome number | Strand direction | Location |
| --- | --- | --- | --- | --- | --- | --- | --- | --- | --- | --- |
|  |  | Molecular weight (kDa) | PI |  |  |  |  |  |  |  |
| CsSAUR1 | 167 | 17.9 | 9.06 | Csa024822 | Nuclear | NA | 0 | 1 | - | 6063783-6064286 |
| CsSAUR2 | 143 | 19.2 | 6.91 | Csa017224 | Cytoplasmic | CU104090 CU174707 C | 0 | 1 | + | 8693129-8693560 |
| CsSAUR3 | 153 | 17.3 | 9.19 | Csa016792 | Nuclear | CU101110 P | 0 | 2 | + | 12495610-12496071 |
| CsSAUR4 | 109 | 12.4 | 7.76 | Csa016791 | Mitochondrial | CU102586 C | 0 | 2 | - | 12515138-12515467 |
| CsSAUR5 | 97 | 11.0 | 8.69 | Csa016789 | Mitochondrial | NA | 0 | 2 | - | 12534435-12534728 |
| CsSAUR6 | 97 | 10.9 | 9.58 | Csa016793 | Mitochondrial | NA | 0 | 2 | + | 12536463-12536756 |
| CsSAUR7 | 95 | 10.6 | 7.77 | Csa016788 | PlasmaMembrane | CU124497 P | 0 | 2 | - | 12538088-12538375 |
| CsSAUR8 | 95 | 10.6 | 7.87 | Csa016794 | Mitochondrial | NA | 0 | 2 | + | 12549855-12550142 |
| CsSAUR9 | 95 | 10.9 | 9.43 | Csa016796 | Mitochondrial | NA | 0 | 2 | + | 12553060-12553347 |
| CsSAUR10 | 97 | 10.9 | 9.03 | Csa016797 | Mitochondrial | NA | 0 | 2 | + | 12566233-12566526 |
| CsSAUR11 | 97 | 10.9 | 9.39 | Csa016798 | Mitochondrial | CU167107 CU162172 P | 0 | 2 | + | 12568124-12568417 |
| CsSAUR12 | 97 | 10.9 | 8.89 | Csa016799 | Mitochondrial | CU102247 C | 0 | 2 | + | 12571177-12571470 |
| CsSAUR13 | 97 | 10.9 | 8.66 | Csa016800 | Mitochondrial | CU093849 P | 0 | 2 | + | 12575395-12575688 |
| CsSAUR14 | 112 | 12.2 | 9.93 | Csa016785 | Mitochondrial | CU117979 P | 1 | 2 | - | 12579036-12579264 |
| CsSAUR15 | 141 | 15.9 | 7.93 | Csa016801 | Mitochondrial | NA | 1 | 2 | + | 12581083-12582815 |
| CsSAUR16 | 97 | 11.1 | 9.37 | Csa016802 | Mitochondrial | CU144375 P | 0 | 2 | + | 12585104-12585397 |
| CsSAUR17 | 80 | 8.9 | 8.74 | Csa016784 | Nuclear | CU114967 CU091596 C | 0 | 2 | - | 12586603-12586845 |
| CsSAUR18 | 97 | 10.9 | 8.79 | Csa016803 | Mitochondrial | NA | 0 | 2 | + | 12589680-12589973 |
| CsSAUR19 | 81 | 9.4 | 9.30 | Csa016804 | Nuclear | CU106621 CU112413 P | 1 | 2 | + | 12593201-12594118 |
| CsSAUR20 | 97 | 11.1 | 9.69 | Csa016805 | Mitochondrial | NA | 0 | 2 | + | 12599563-12594116 |
| CsSAUR21 | 115 | 13.0 | 9.15 | Csa003634 | Mitochondrial | NA | 0 | 2 | + | 17703185-17703532 |
| CsSAUR22 | 153 | 17.7 | 10.38 | Csa018026 | Mitochondrial | NA | 0 | 3 | + | 2476673-2477134 |
| CsSAUR23 | 111 | 13.1 | 8.96 | Csa000379 | Nuclear | CU104105 CU096264 C | 0 | 3 | - | 6585210-6585545 |
| CsSAUR24 | 169 | 19.5 | 10.52 | Csa017646 | Nuclear | CU089608 CU122098 P | 0 | 3 | + | 11491741-11492250 |
| CsSAUR25 | 120 | 13.5 | 8.67 | Csa019636 | Nuclear | NA | 0 | 3 | - | 29868119-29868481 |
| CsSAUR26 | 150 | 17.1 | 9.88 | Csa017895 | Mitochondrial | NA | 0 | 3 | + | 36303236-36303688 |
| CsSAUR27 | 108 | 12.2 | 9.06 | Csa002348 | Extracellular | NA | 0 | 3 | - | 36344332-36344658 |
| CsSAUR28 | 165 | 18.3 | 9.51 | Csa002266 | Nuclear | CU145239 CU105788 P | 0 | 3 | - | 37533565-37534062 |
| CsSAUR29 | 113 | 13.7 | 8.54 | Csa013679 | Nuclear | CU126628 CU113530 P | 0 | 4 | + | 18429649-18429990 |
| CsSAUR30 | 149 | 17.3 | 4.93 | Csa013718 | Nuclear | NA | 0 | 4 | + | 19298212-19298664 |
| CsSAUR31 | 121 | 14.3 | 7.05 | Csa015482 | Nuclear | CU178185 CU174661 C | 0 | 5 | - | 3400221-3400586 |
| CsSAUR32 | 167 | 18.9 | 5.45 | Csa011335 | Nuclear | NA | 1 | 5 | + | 18995279-18995753 |
| CsSAUR33 | 176 | 20.9 | 10.28 | Csa005513 | Mitochondrial | NA | 0 | 6 | + | 679430-679960 |
| CsSAUR34 | 172 | 19.7 | 9.30 | Csa008434 | Nuclear | CU154521 P | 0 | 6 | + | 6366889-6367407 |
| CsSAUR35 | 100 | 11.7 | 6.21 | Csa008479 | Cytoplasmic | NA | 0 | 6 | + | 7038335-7038637 |
| CsSAUR36 | 139 | 15.7 | 8.69 | Csa020734 | Cytoplasmic | CU113518 P | 0 | 6 | - | 9658479-9658898 |
| CsSAUR37 | 135 | 14.9 | 4.72 | Csa015210 | Extracellular | NA | 0 | 6 | + | 10249819-10250226 |
| CsSAUR38 | 125 | 14.4 | 9.18 | Csa020553 | Extracellular | NA | 0 | 6 | + | 18409128-18409505 |
| CsSAUR39 | 124 | 14.3 | 9.00 | Csa011892 | Chloroplast | NA | 0 | 6 | - | 20871455-20871829 |
| CsSAUR40 | 125 | 14.5 | 5.69 | Csa011886 | Nuclear | NA | 0 | 6 | - | 20952892-20953269 |
| CsSAUR41 | 135 | 15.2 | 6.08 | Csa011993 | Mitochondrial | NA | 0 | 6 | + | 21624854-21625261 |
| CsSAUR42 | 151 | 17.1 | 10.04 | Csa014013 | Chloroplast | CU127513 CU084978 P | 1 | 7 | + | 458719-459174 |
| CsSAUR43 | 99 | 11.0 | 9.41 | Csa014012 | Nuclear | CU132750 P | 0 | 7 | - | 468933-469232 |
| CsSAUR44 | 84 | 9.5 | 7.83 | Csa014011 | Nuclear | CU118953 P | 0 | 7 | - | 482447-482701 |
| CsSAUR45 | 96 | 10.7 | 6.71 | Csa014008 | Nuclear | CU091525 P | 0 | 7 | - | 489234-489524 |
| CsSAUR46 | 100 | 11.2 | 6.06 | Csa014007 | Nuclear | CU114371 P | 0 | 7 | - | 508489-508742 |
| CsSAUR47 | 98 | 10.9 | 6.72 | Csa014006 | Nuclear | NA | 0 | 7 | - | 510467-510763 |
| CsSAUR48 | 100 | 11.2 | 6.06 | Csa014005 | Nuclear | NA | 0 | 7 | - | 515918-516220 |
| CsSAUR49 | 89 | 10.2 | 8.91 | Csa014004 | Extracellular | CU107648 CU111952 P | 0 | 7 | - | 518490-518759 |
| CsSAUR50 | 78 | 8.9 | 7.68 | Csa014014 | Nuclear | NA | 1 | 7 | + | 533109-534140 |
| CsSAUR51 | 84 | 9.6 | 8.52 | Csa014015 | Mitochondrial | NA | 0 | 7 | + | 534533-534787 |
| CsSAUR52 | 84 | 9.8 | 7.69 | Csa014016 | Nuclear | CU114920 C | 0 | 7 | + | 536604-536858 |
| CsSAUR53 | 92 | 10.3 | 9.03 | Csa014017 | Mitochondrial | CU095159 P | 0 | 7 | + | 538992-539270 |
| CsSAUR54 | 90 | 10.5 | 6.81 | Csa014019 | Mitochondrial | NA | 0 | 7 | + | 547922-548194 |
| CsSAUR55 | 93 | 10.5 | 7.97 | Csa014002 | Mitochondrial | NA | 0 | 7 | - | 550924-551205 |
| CsSAUR56 | 75 | 8.1 | 10.01 | Csa014020 | Chloroplast | NA | 1 | 7 | + | 556533-557279 |
| CsSAUR57 | 113 | 12.8 | 6.58 | Csa014021 | Extracellular | NA | 0 | 7 | + | 569969-570291 |
| CsSAUR58 | 148 | 16.5 | 9.28 | Csa024696 | Chloroplast | CU166375 P | 0 | 7 | - | 1116438-1116884 |
| CsSAUR59 | 127 | 14.5 | 9.30 | Csa007553 | Nuclear | NA | 0 | 7 | + | 8613886-8614269 |
| CsSAUR60 | 158 | 18.2 | 8.68 | Csa010042 | Nuclear | NA | 0 | 7 | - | 17935526-17936002 |
| CsSAUR61 | 125 | 13.8 | 5.37 | Csa009878 | Cytoplasmic | CU108197 C | 0 | 7 | + | 18312324-18312701 |

C and P: The unigenes containing the whole or partial ORF of relevant CsARFs, respectively.

NA no available

**Table S2-5. LBD gene family in cucumber**

| Gene | Predicted protein (aa) | Deduced polypeptide | | Predicted CDS ID | Subcellular localization | Unigene or est | Intron | Chromosome number | Strand direction | Location |
| --- | --- | --- | --- | --- | --- | --- | --- | --- | --- | --- |
|  |  | Molecular weight (kDa) | PI |  |  |  |  |  |  |  |
| CsLBD1 | 184 | 20.3 | 7.63 | Csa005333 | Nuclear | NA | 0 | 1 | + | 669678-670232 |
| CsLBD2 | 244 | 26.7 | 8.43 | Csa005097 | Extracellular | CU116739 CU127950 CU087901 P | 1 | 1 | - | 2342714-2343538 |
| CsLBD3 | 166 | 18.7 | 8.75 | Csa003724 | Extracellular | NA | 0 | 1 | - | 5447872-5448372 |
| CsLBD4 | 183 | 20.6 | 6.06 | Csa017193 | Extracellular | NA | 1 | 1 | - | 8314172-8314857 |
| CsLBD5 | 136 | 15.2 | 4.63 | Csa017203 | Nuclear | NA | 1 | 1 | + | 8329219-8329733 |
| CsLBD6 | 170 | 18.6 | 8.32 | Csa014687 | Nuclear | CU170725 P | 1 | 1 | - | 29010522-29013600 |
| CsLBD7 | 218 | 24.3 | 8.49 | Csa012250 | Nuclear | NA | 0 | 2 | + | 5562563-5563219 |
| CsLBD8 | 309 | 34.8 | 7.07 | Csa018643 | Nuclear | CU135623 P | 0 | 2 | + | 13980403-13981332 |
| CsLBD9 | 191 | 21.4 | 5.73 | Csa011427 | Extracellular | CU092905 P | 0 | 2 | + | 16155908-16156483 |
| CsLBD10 | 93 | 10.4 | 4.71 | Csa018594 | Nuclear | NA | 0 | 2 | - | 18687683-18687964 |
| CsLBD11 | 235 | 26.3 | 8.74 | Csa001306 | Nuclear | NA | 0 | 2 | - | 21368546-21369253 |
| CsLBD12 | 231 | 25.0 | 8.93 | Csa000286 | Nuclear | NA | 1 | 3 | - | 7670021-7670964 |
| CsLBD13 | 154 | 17.1 | 7.61 | Csa008642 | Nuclear | NA | 1 | 3 | + | 12377547-12378211 |
| CsLBD14 | 302 | 32.6 | 8.91 | Csa014935 | Nuclear | CU105421 C | 1 | 3 | - | 16580929-16581934 |
| CsLBD15 | 243 | 27.0 | 6.04 | Csa001120 | Nuclear | NA | 1 | 3 | + | 19335095-19336409 |
| CsLBD16 | 241 | 25.9 | 6.74 | Csa001344 | Nuclear | NA | 1 | 3 | - | 19356643-19358148 |
| CsLBD17 | 222 | 23.7 | 6.37 | Csa002692 | Nuclear | NA | 1 | 3 | + | 32281650-32284171 |
| CsLBD18 | 177 | 19.5 | 7.58 | Csa009094 | Extracellular | CU114814 P | 0 | 3 | - | 33654878-33655411 |
| CsLBD19 | 205 | 22.4 | 6.28 | Csa009364 | Nuclear | CU133789 P | 1 | 4 | - | 21527028-21527757 |
| CsLBD20 | 173 | 19.1 | 6.56 | Csa012464 | Nuclear | NA | 1 | 5 | - | 7451299-7452289 |
| CsLBD21 | 220 | 24.2 | 4.47 | Csa017697 | Nuclear | NA | 1 | 5 | + | 8274251-8275156 |
| CsLBD22 | 167 | 18.3 | 6.48 | Csa018282 | Nuclear | NA | 1 | 5 | + | 9548497-9549129 |
| CsLBD23 | 245 | 26.9 | 9.18 | Csa016051 | Nuclear | NA | 2 | 5 | + | 9896277-9898151 |
| CsLBD24 | 161 | 18.1 | 8.91 | Csa012684 | Extracellular | NA | 1 | 5 | + | 17435150-17436220 |
| CsLBD25 | 164 | 18.1 | 8.54 | Csa011308 | Nuclear | NA | 0 | 5 | + | 18468619-18469113 |
| CsLBD26 | 308 | 34.7 | 5.25 | Csa018933 | Nuclear | NA | 1 | 5 | - | 26053646-26055930 |
| CsLBD27 | 150 | 16.5 | 5.51 | Csa005699 | Nuclear | CU097704 C | 1 | 6 | - | 1331948-1332757 |
| CsLBD28 | 255 | 27.9 | 8.61 | Csa005684 | Nuclear | NA | 2 | 6 | - | 1501376-1502752 |
| CsLBD29 | 240 | 24.8 | 7.07 | Csa025829 | Nuclear | NA | 1 | 6 | - | 5139372-5141747 |
| CsLBD30 | 213 | 23.1 | 6.92 | Csa025830 | Extracellular | NA | 1 | 6 | + | 5151168-5154005 |
| CsLBD31 | 173 | 19.1 | 7.65 | Csa020733 | Nuclear | CU149352 P | 1 | 6 | - | 9739705-9741889 |
| CsLBD32 | 162 | 18.1 | 5.47 | Csa011639 | Nuclear | NA | 1 | 6 | + | 11780863-11781713 |
| CsLBD33 | 228 | 24.6 | 8.18 | Csa020070 | Extracellular | CU175255 CU163327 P | 2 | 6 | + | 14015649-14017865 |
| CsLBD34 | 241 | 26.6 | 8.05 | Csa010196 | Nuclear | NA | 1 | 6 | - | 23923020-23923817 |
| CsLBD35 | 227 | 25.7 | 5.62 | Csa000736 | Cytoplasmic | NA | 0 | 6 | + | 28324801-28325451 |
| CsLBD36 | 162 | 17.5 | 8.14 | Csa007372 | Nuclear | CU127926 P | 1 | 7 | - | 8541062-8543495 |
| CsLBD37 | 184 | 20.2 | 6.28 | Csa021175 | Nuclear | NA | 1 | 7 | + | 13418918-13419695 |
| CsLBD38 | 201 | 22.2 | 5.57 | Csa009912 | Nuclear | NA | 1 | 7 | + | 18745979-18747557 |
| CsLBD39 | 49 | 5.7 | 9.93 | Csa009951 | Nuclear | NA | 0 | 7 | + | 19220543-19220692 |

**C and P: The unigenes containing the whole or partial ORF of relevant *CsARFs*, respectively.**

**NA no available**
