# Supplementary material for: Genome-wide identification and transcriptional profiling analysis of auxin response-related gene families in cucumber
Source: BMC Res Notes. 2014 Apr 8;7:218. doi: 10.1186/1756-0500-7-218 (PMC4108051; doi:10.1186/1756-0500-7-218)
Supplement: Additional file 7: Table S3 — Primer sequences for qRT-PCR expression analysis. [file 1756-0500-7-218-S7.doc]

**>CsARF1**

GGAAAAGAAAAAAAGAAAAGAAAGAGAGAGAAAGAAATAATAAAATAGTAAAAGAAGAAAAAAGAGAAAAGAGAAGAGTTGGGGGGAGAGGAGATTACGGAAGGTGGTGTTGAGAGTAAAGGAAGACTAAGGAATTGATGTATCCCTTTCGGGGCGCTCCTTTCTCTCCTGTTCCGCTTCTTCTTATTCTTAATTATTATTATTATTATTTTTAATTACTAATTAATAATCATCATTCATTCTTATTTTCTATATTCCAAATAAATAAAATAAATATAACCAACCAACTCCTTCCTCCTTCTATTCCCATTTCCCAAACCTTTTCTTTCTTTTCTTTTTCTTTTTTCTATCTGTTGTATAGTATGTATTTATGTGGTGTAAATTAAGAAGGAAGGGCACACACAGACACAGACCCATTTCTTTTCTTTCTTTCTTTTTTTTTTTTTTTCTTTTGGTAAATAAATTAAACAAATTCCCATTTCTTTCTCTTTCCTCTTACACACAAACAAAGAAATTTCCTTGTCGGCTTGGACCAAACACCATCTTATCGTTTCTTCTTCCTCTTTCCATTCCATTCCCTTCCCTTCCTTTCCCTTCTTCCATCCCTTTCATCCTTCTTCTTCTTTCTCAAAACCCTACTTTCTCATCTCCATTTCTTTTTACTTCTTCAACTGCTCCCTTCTAATCTTCAGGTTTGTGATTTTTTTCCTTTTTTTTCTTCTTGGTTTTTTTTTTTTCTTTCTTTCTTTCTTTTTGCTTTTTTATGTTTTTGCTTATTGTTTCTCCCCTTTTCCCCAAACCGGGTCTCTGTTAGATCCACCTTTTGGAGGTTTTTTGTTTCAATTTTCAGGTTCATAGACTTCTGGGGTTCTTCCTTTGTTTTTTTTTTTTTCTTCTTCTTTTGTTTTTTTTTTTTTTTTCTTCTTCTTTTTTCTGGGTGTTTGAGGTTAGGTGAAAGTGTTGAACTCATGTTGGGGGTTTTGATTCGAACTTCTCGTCAATGTGTTGTTTTAAGTCTTTGTTGATTTCAATTGGATAATCTTCTATTTTGCTGAATCACGAGACGGATAATCTTCTATTTTGTTCTTCTTCTTATTATTATTTTTTTTTAAATGAAATGGATGTGCGTGTCCCTTTGTTGGGTTTCAATTTTATCTTTGACATCGTCAATATTTCATGTTGTTTTGATCGGATTTGCGGTAAAAGTTCTAATAGATAGATAGCGGCTGCCTTTTGGGCGCTTCATTTAAGATGCACAAAACAAGGTGCGGCTGCCTCGCTGACAGCGTGGCTAAGCTAAGTGTCAGAGGCATTTATTGATCTATAAGGCCGCTCCTATCTCATAGATTCCATTTACTGTTGTTCTTGGTTGGTAATAAATATCTTGAAAGGAGGAGTATGTAAACACGTTTATCCCATGTGTTCTGCCCGGATAGTAAGATATTTAATCTGATTCTTGTGATGTTCTTTTTTAACGCATCAACCACCGGTTTTGCAGGTTGATTGGCACATAAATATCTCTCTGATATTATTATGTCCTTTTCAGCTGCTTCAAATCTTCCCTCAGGAGGACCCCATTCAGGTTTGTGGTTATCATTTTGGAAGTTCTGTCCATTTTATTTAATTTTTAGTCACTGCTGTTTTGGTGAGGGATTGGCTGTTGGTGATCTAAAAGAATGAACCTTGGATTTTCAGTTTAGGATAATCTACTGTTAAAATTAACACGTCGTAATTTTGCCTGCTACTCTTGCCTTCTTTGTTATGCTGAATGTCTGAAATTTTAATCCTTGAGTAAGCTTGACAGGTTGGCTTTATGCAATAATATCAAAGACAGTTCCATTTTCTAGGTTTGTGTAACTGGGTAAATTCTCTTGGGTTTGACCTTCAATGATCTTCACAGCAAAAATCATTCTCTCTTATCGGCTGGGGAAATTCTCCTTTGAGGAAGATGCCTGTCCTTTTTGTGGAAATTTATACACTTAAATATAGAGTTTCAAAAG

**>CsARF2**

ATCTATCTACCCAATATAAAAGAGAAGAAGGCAAAGCAAAAGAGGCCAGGAGATTGTTGTGGAATTGTGACTTACTGACTTAAGTGGTTTGGGAGTCAAAATAGCAGTGTTGAGGAGAGAGGAGGCAGGGCCCACTTCGTTGTGGTTATATTTGTTGTCATTGGGAAGCTCACAGTCACAGCTCACCCTAATTCAATAACTAACTAACTAACTAAGGTCTTGTTTTTTAAGCATATGCTTATGTGTTTTGTTACTACCTACTCCACTAAGCTTCTCTTTTAATTTGCACAAGTCATGGTGTTGTGATTTTTACTAAATTTTTGTATATAAAAAAGTTATAATTAGAATGAAGTGAAATGTACATCTATTAATAAAGCTTAGGTTTAGGATGTGGTGTATGTGAAATATTAACATTGATGAGGTCTTTATTTTTATTTTTTTATAAAAAAGAGATGTGTATATATATATATATATAAAAGATTGATGGGTGGGGTGGATCCCACCCAAGGCATGATGATGATGATGGTCATCCTCAATTGAGCAGTCGTCCGTACGCTTACTTTGTTGTTTCAGCTCGTGACCCCTTCCTTTTCCTTCTTCAAGGCAAAGGGCCCTCCCATCCTCTCCTATGCTTCTCCCTTTATGTGCACAGCCACAGGCTCACAGCCCCCCTCTCTTTCTTTAGTTCCCTACATCCTCTATTCTTCATTCCATTCAATTTAAACTAAATTACATTTACCCCTTTCTTTGTATTACAAAATTAATTTATATTGTCTTCATGAATTACCATTACAAAGAAACAGGGTTGCGAAAAAGAGGCTACAAATTATTGTTATTATTATTTTTTAAGAAAGAAAGAAAGAAAAAAGAAGTGAAGGGGGGACAAAGATTCCAACTCTATTTTGTTGCTTTAGGCCATACACAGCACACCAGCTCTTTCATTACCCAACACACTTGGGTCCCACTCTCCAGATTGACACGTGACCCTCAAATATTTAACTCTCTTCTCTATCTTTGCTACAAGTGCGGGCAGCGAGGGGAGTTTTACCTTTTACCCTTACAATTATTGCGTATTTGCAAGTTTAATAATGAATTAGTCATACCATTTTTTTTTTTTTTTGTGGGAGGGGGAGAAATCCAACCTCCATCAGTAGTACAAGCTCAGTTATACTCGTTACTTTTATTTTCAGTGTCAACAAAAAAAAAAAAGGACTCTTTATTGAACTGTTTGATAAACATTAAACATAGCAGATTATATTATATTATATTCTCAGGATCTCTCTCTATATACAGATTTGAATTTTTAATTCCAAAACGATGTTTTAAAGATAGAGGCAGCCCACATACGTATGGTAATGCCTTTGTCCCCAAAAAACAAAAAAAAGAAAGAGAGAGAGAAAGGAGTTTAGTTAAGTAAGGTTAGCGTGAAAAGGGGAGGGGTAGTTGAGAAGTAAGGGAGTGACACATGTTAAGCATCCAATCCAACGTTGCCCGTAGATACCACGACAAACCCATTCCCACGTTTCTATTCTATCGTAATTACTATTTTCTCTTACAACGCCGTTACCCTATCACACGCCGACACCTCCTAACGCCGTCTCTCTTTCCTTCAAAATTATTGCCTTTCCTTCTCTCTCCTTCCTTCCTTCACCCATTTCCTTTCCAATTCACCCCACTCCTCTCTGCCTCTTCTTACCCAATTCACCCTCCTAATTCCATCCATATTCCATCCTACTACTGTTTCAGACAGTTGTAGAGAGAAATCCCAAGTTCATGCTCAGCCCAGTTGCAGATGGCCACCGTATCCCCACCCACAATCTAGAGTTTCCTTCCATCCGATTACCCTTTTAACTCTCTCTCTCTCTCTCTCTCATGGGGATTCTCTCATCTTTCCTCCTTACTACCTAGGGTTTTCCACCTTCCTTCTCTTCTTTCCCGCTTTTGCATGACACTTACCAATTCCCTACCTACCTCCGAGATCTGTCATCAGATTCCCTAGA

**>CsARF3**

TTCCTTTGTCTTTAATATTCTTAACTACATCCATTCTTAAAAAATAAATAATAAAAAGTTGATCAATCATAACATTTTTGTTAATTAATATATACTTGAAATGTAATTTATATTTACTTACTTTTATTTAAAAAAATAAATTCTTAATATCTCGTTTCATTATACTACAAAAATTGAGCCTAGAACGTTCTATTAGAATGCTATTTGAGATGTGATATCTTAGCAATTTATCCTTAAAACAAATTTTGTTTACTAAAAATGTCCATCTTAAGTGATGAGAGTAACAGCGTTTTGATATACAAATTGATGTAATTCATTGATAGTGTATTAACTAGTAGATGATGGTGATTGTTTTATTGAGAATAAAAACTTAGAATACATACATATATGTCTTTGTGGTTGGAGTTTATATAACACACTTATCTAAACCATATAGTCAAAATCGTAAATAACTTAACATAGAAAATACAAAATTAACTTGTAATTGGTTGTTGGGAGTGTACCCAAATATCAAATTAAAGACATCTCGATAAAGATAGTGTGTTTTGAATCTAAGTTTAAGTTAAATAAATAATATAAGCAAAATTCGATTGGTATACGAAGTACAATTTTCATCATGAAATCAAATGTTCGAATCTTTTTCAAATCGTCGTACAAAAAAGAAAAGTCCATCGTAAAGAAAAGATGGTGGCATTTTCCCCCACACCCAACCAAAAAAAGAGGATGGTGGTAAAGACAAAAGGAGAATGGACCACAAGAAAAAATTTAAAACCAAACCGCATCTTCAACGCATGACTTCATTACAAAAATACATCAAAATTGTGTCTTTTAAGGCATCATTTTTTCGTATAGTCAGTCAAATGTATAATTGAACAGTTATTTGACTCAAACCTAAATTTCGATGAATAAGATTAAATCGATACAAAAATGGCTCCCACCTTATCTAAAACTTGTCATTTAGTAACTTTTGTTTTTTTTGGGCTTATGCCAAGCATAATTCAACATTTTACCCCAAAAAAAACTAAAATTTAATCAAATTTCCTATTCATTTTGTAATTTGCATTCAATATTATTGTTTTTAATTATGAAAATTCCTCGAAATTGAAGTGATGCATACATATTTATAAAAACGTGCATGGGGAAATTTAAAAAGAAGAAAGAAAAGAAAGAAAAAAAGAAAAAAAAGAAGAAAGAAGTATTATCCAGGTCAGCGTTGTAATGATATATGGTCATAACCCCGAAATGGAATATCTGTACCTAAAAATAAAATAAAATAAATAAAAAAGAAAAAAAAAGTCTATAAAATATTCTGCTTGGTTAAGTTGAGCCAACCATGTTGCCTGCTATGAAGCAGACAACGAAAGGGACGTTACTTAACGGTAACGTTGCTAACACCTCATGTCTTCCGTTACCCAAACAACACGCATGCCGATGCCGTACTATTTCCTCCGTTATCTAACCTCCTTTAGTTTCCTTCTTTCTCCGTCTTTCTTTCTTCCTTTCCTCACTCCTTTTCGTTTCTCACGCCGTCCATTTAATCATCTTCTCCGCTTACAAATCTAACCCTGGTTGAGTTTACAGCCTACTGAAGCTTCACAGTTCGGGCTGCTACAGAACTAGTTCCCGTATAATAGGACGCGCGCCAAGCCGTTTCACGATTCTTTTTCTTTTCTTTTCTTGTGACGGCGTTTTTCCCCCCTGACCAGCGACAAAGAGGATCGGCTCTTGATTTTGTTAACGGTGTGCGGTCACGAAAGGAGTTTAAGGTTTGTGTTGATGGTTTAATTTCGTTATTGATGTTGTTTGAGCTTTGACTGTGTGTTGTGGAATGGGAGATTTTGTGTTTATGTGTATTAGTTTGTGGAGGAAGGAATTGGAGTGAGTTTTTTCGGTGGTTTTGTACCTCATGGTTGGTTTTGATCGTGTGTGTTCAGGGGCGTTCGGGGATTTGTTTTGAGTTGCATTGATTTTTGGTTGTTGTTTTACTTTGGAGCTTGACG

**>CsARF4**

CTCTAGTCAAATAAAAAAACAAAATTACAAATACTAACTTACACACGATACAAAATATATATATAACAAATTTAGATCAAGCTCTTATAATTTATAAACGATAAACCATATCATTAGTGTTAATTAATGATAATCTTTTTTAATACTTACAAGTCTTTAAACACTATTCAATAAAATTATCCAATCATGTAAAAGTTAAAATTGTGAATTATCATTGAACTAAAGAAATTGTGAATGGAACAATATTAGTTAGGGAAGAAATACGCATCAATCAAAAAAACATATAAAATACAGAAAAGTATATTATTGGATTGAAACCTTTACAAGGCAGGAGATAAAGTATGGATAATCACTAGGTAGTTAAATCCTCTTTTAACTTTTGTTCATACAAAAACGCAAACCTTTAATAAAATTATAAAACAATAAAATAAATTTAAAAACTGTTAAAAACATTTAAAACATAAGAAAGTATTGTGTGATTGATTAAAGTTATTATTATTTTGTTTAATTTTAGTATTAAGATTGAGATGAAAGTGAGATTGAATTTGCTATATTTTTGACCATTTTTCTTAAAAATATTATTACGTTTCTACAAATATTTTGTAGGAAATATTTGAAAATCTATTAAAAAATGTAGAACTTTTGCAATACTATTGTAAAAGCAAATTTGAGGAGAAAGGAAAAAAAGGGTAACAAAAGAAATTAGAGGAAGAAACGAAACAAATAAAGGAAGAAGAAGAAGAGGAAGAGGAAGAAGAAGAAGAAGAGGAAGAAGAGGACGCGCGTTTTAAGGGAGTGAAAAACAGTGATTCCATAACTAGAAATAGAAAAAAGAACAAAAGCTTTGATAAGACGTGAGATGCAGAAAAGAGTTGTACTCATACTGCTCACTCCTAACTGGTAACGCTGTTTGAGCTTTCTCTGTCTTCCCATTTCCCATTTTTCAATTTGTAATTTTGTTGCAATGTGCGGGGGCTCTCAGTCTTTAACAAACACCTTTCATCCAAACTCTCTAACCCCACCGATGGCTTTGTTCATGGTCCCCAATTCTCTCTCTTTCTTGGGTTGTTTTCTTATTACATTAGGATTCAATTCATCTGGGTTTGTGATTATCAGTTTCCTTTTTGCACTTTGGGTGATAGGCAAAATGGGGTTTAAGAGATTTTAATTGCGTTGGCTTAATATGCCCAGTACCTGTTTGTTAAAATAACTAAGAGAAGGATCTTCTTGTTTATTAGGGGAAAAGATTGTACAAAGAGAAAAAGAAATATTATTTTTGTTGCATTAATGAAAACCAACCTTTTTGTCTTTGGTGGAGGGCCAGGGTCTCACCCCACACACACTCTGTTACAGTGAAGGAAATTATTAATAAAGGGGAAAGGAGGGAGGAGGGGAGGGGAAAAAAAAAAAAGATAAAGCCAAGGCAAAGCAAAGGAGAAGGGGATGGGGGAGTGTGGGTTTTGTTCTGTCTTAGCCTGTCCCCCCCACCCACCCACACATATACGCACTCCTCTCTCTCTCTTAACAGTCCAAAAGCCACCAAAGCATTGGCCTTTTTTCGCCTTTTGCTTCTGTGTTTCCTATGCAATTGTCTATGTCTGTTGCTTCTGCTCCCTCTTCTCAGTACCTGCAAATGGGAGAGGAATCATACTTTGAATTTCTTGTTATTATTTGATACCCTCCTTTTCTTTTTTTTCATTTCTTTTTTTTTTCTTCACTATCTTTTTAGCCTACTGGCTTAGCATCCATCCAAACACTACTTTCTTGTTGTTTTCTGTTCATCTCCTTTCACTTCTCTCCTGTTTTTAGCTTTATTGGTATCCATTCAGGATATGTTTTCAGCATTCTTTACCATGATTTGACCTCATTTCCAGTTTTCTACAGCTCTGGTCTTCTTGTCACCTTATTGCCAATTGATCACTCCGTGCCATTCCCCTTTCTTGTTTTAGTTCTGTTGGGAAGCATAGCAATAGGACACTTGAGTAACCCCTGGGTTGTTC

**>CsARF5**

AGCATGACCCATCAATTATGGAAAAAAACTCCCCTTTTCTCCACTCACCATTTCTCACCTTCCCAAATTCTCTCTTTTCTTAAACAAAAATAACATTTATGAAACCAAACTATCTTACATAAATCAATCAATTAACATGTTACATATTATATGTGTTTCTTACACATCAACAACCTTTAGTTGTTACAAGTTTAATTTTAAATATTTTAATTAAATTTAAGAGGCTTCTCTCTTATATCAAAGAATAATTTCAATTATATCTTTTGTATAAGGATTGGTTTTATAAATAATTTAAGAGCTTATAGATTTATACAACACATAAAACTAGCAAAACTTTGGTATGTCATGCTATCATTATTTAAGAACCAAGAAAACAAAATATAGCGCCAACAATATAGAGAATAGTCGTTAGCTATGGGAGTATCTACAAATATTTTACCCAAAGCTAAAATCTTTAAAAGTTTACTGCACTCACTTAAAAAAATTCTAACTACGAACGCAATAGCATATCCATTTTTCAAGACACAAGAAGAGTAACTAAAGAATTATACGAAGATATACAAAAAAAAGCATGCACTCTCTTTACGAGAAGAGAACCTACCAAAAACGTGTAAGAGAAAACTTGAATTTGAGCATTTAGATTCGTAATATCAACCATAAAAGAGATTTAGAAGAGGCAAAAAAGATACGTTATGATATTTTTATGTTGGTAGATTATGGTGTCGTGTATAGAATAACAAAAAGTTGAAGCCAAACAGAGGTGAGTTATGAGTGGATGAAATAGAATGTCCGATGTTTTTGATTGATAGTATATGCAAAGAAGCAGTTTGATTTAGGTTGAAATTGAAGTAGTTGGGTAAGTGTGAAACGTAAGATAGTAATTAAGGAGTTGAATTAGGGTGATGTAAAGAAAAGAAAAGAAAAGAAAAAAGTGTGTGTGTGTAAAAAGCTGAAGAGGAAAGGAGTCCATTAAGGGAGTGCCGCGTGTGACATTGTGACCTCGTCTGCAGGCCTCTCCTGTGAATTTGTCGCGAATCGCACGCGCGACGAACGGAAAGTCAAGATTTATATAGGGGAGCCACGAACACAAAGAAGACCTCCAAATCAAATCACCTTTCTCATTCCCTTCTCTTCCATTCCTCTTTTATTCTTTTCCATTCTTTTATATTTATTATTATTAACTTTACACAAACTTTTTTAATTTTCAAATCTCTACAACCATCACTTTAAATTTTTATTTATTTTTTTAAATAACTAATTACACAACTTTGTATACGGGACATTTACGCATTATTTGGTAGCTCAAAAATTAAGGTTACAAATCCTCCTTCTACTCCTAATTCTTTTAATTTGTTATATATATCCTACCAATAGTTTAAAGAATTCAATTATTGTATTTAAGAAAAAGGCAAATCAAGAGAAAATAGATTAATTTTCATTTAAAATTGGTGTTTCATCCTATAAATTTATCAAAATATTGTAAATTCACTAATTCCTATAATATAATATAAAGTTTTTTGGAGAATTAAAATAAAGTGAATTTTATAATTGATGGTAAACACAAAAAGTAAAATAAAAAAAGAATATAGTAATATAAAAATAAGAAAGAAAGGAATGACAAAATTGAGGAGATTAATTAATGAAGTTTAGCAAAAAAAGAGGGAGAGGGGATCGAGGAAGACGAAAATCCAAAGGCTCTTTTTCAAACCATCGCCAATCCACGTGCTAAAATGCGCCTTCTCTTAACATTCTTCCATTCCACCAATCCCATTCTCGCTTTTTTCCAAAAAGACAATCTCTTTCTCTCTTTTCTTCTCCCTTTTTCTCTCTCTCACAGTCCCTTTAGCTTTCTTTCTGTACTGTTTTCGTGTTCTCTTAGCCTTTCTCTGCTTTTTCCCACCATTTCTTTACTATTATTTCCCCCCCCCCCCCCCCATTTGCTTTTCTTTTCATACCCTTTTCATTTGGGTTCCTCAAAAACAGTTTAAAGCTGCAAACAA

**>CsARF6**

AAACAGAGGAGTCAGAGAGAGAGAGAGAGAGAGAGAGAGAGAGAGAAATTGGTAGAATGGTAAAGCAATAGTAGTACTACTACTAATCTATCTTTGCAATTTCATAGCCCATCTATCTATTTATCTACCACCACAGCAGAGCCTCAAAACATTAGTTTTAGTACCTTACCACCACGACCACGACCTCCATAACTTTCTATCACTAGCTACTACTCGACCCATTAGCCTTGGATTTCCGTTATTCAAAACAAACTTAAGATACATCCACCAAATGGAGCTCGCATGATGTTGTTTTAATTCTTTTTGGTTTTTGGGATTTTGATCGTTCTTCTTCCTCTTGCTTCCCTCGTTTCTGAAAAGTTCACCACTCACTCAGTGCGTAATGGGTCCTAGTTGATACCAGAAATGAACATGGGGGGAAGGTCGCTTTTCTTTGGTGTGCTTCTTCTACTTCTTTGAGATCTGGGTTTTTTCCTTCTACTAAATATCTCAAACCTTTCATGTTCTTCCTTCAAGATCTGGTGGCTCCCTGGCGTTTGTGGATCTTTAACTCTGTAGTAGTTTCAGTGCAAGAACTCATTAAGAATCAGTAATTTGTGGGTCTGTAGGAGGAGGAGGAGTTGGCTTTTGGTGAAGCTTAATCTGAGCTTTGTTTTGTAATTGTAAATTAAGAGATTGATATAATCTTGGAGGGAGTGTTAGTGCTGATTTGAAGCTTCGGATGAGGCGCCAAATCACTGTGTTCTGTTGTGAAACCGGCTTCGGTTGAGGATTCTAGAGTGGGCACCAAGTTTCTGATACTGTACAGATTGAAGCACGTTTAACTTGAAATTACAATGGCGCTTTAGTTTGGGGAGAGGAATACTGTACTTGTGGAGTGAACAAGAGCCAGATTCTTCTGGGTCGCGGATGTGTTCACATTAGCTATTTATTTATCACTTCGATTGAAGGATATTGTCAAGTTTCGGTAAGAATGAGGCTCTCTGCGGGTGGTTTTAGTCCTCAGGCTCCGGAAGGTACGCCATACTTTGAACTTTGAGCTACTTTTGCTTGAATTTTGTCTGTAAATACATTACGGGAAGTAATCAAAGATTGGAGATGATTCTGCTTTTGATAACTTTCCTATTTGATTTATACCCTTAACGTTTTTGTTCTTCATTTTTGGATTGATTAGGGAGGCTTGCAATTTAAGTCATTTTTTTGCCTTCCGGTGGCCTTGGATTTTGCAGCGTCATGCTTAGATTTATGTCCTTTTCCTGCCTTTGGAGTTTTGTTTTATATTTTTCCAACCTCTCTGTGTGTCAACCATGAAAGTCTTTCTCTCTCTCTTTCTCTCTCCCTCTCTCTCTCCCAATGAGTGGTCCAGCCATGGGATTTCCTAATTGGAAACATTGGCCTTTTTTTATGAAAATTGACCATCAATTTTGCAGGCGAGAGAAGGGTCCTGAATTCCGAGCTTTGGCATGCGTGCGCTGGTCCTCTTGTTTCTCTGCCGGCTGTTGGCAGTCGTGTTGTTTATTTTCCACAAGGCCACAGTGAGCAGGTGAACCCCATGAATTTTCATCTTTATTCAATGGCCACTTCAAATTTCTGACTTTCTTTTGAGATTTAACAACCTCGATGGTTATTTATGTGTTTGGTTGCTACTTACTCTGTTATGCTCTATGTTTTAATTGTGATTTTGACAGAAGTCAGCCTGTTTGATTGATATGATCTCTTATCTGTAGTTTTTGTAAATTTAATTGAGAAAGTTTTGGCATTTGGGATTATGTGTTGTTTGGTCATCTGATTGTTGTTCATTTACGAGCTTTTTGTTTGTGCAGTCTAGTGAGAGTATACTGATCCGTTTCATGGAATCTTTAAGCTCTTTAAATCTTAAAGCTTACACCCACTTGTTTCCTGTATAGGTTGCTGCATCTACCAATAGAGAGGTTGATGCCCAGATTCCGAATTACCCAAGTTTGCCGCCGCAGCTCATTTGTCAGCTTCACAACCTGACA

**>CsARF7**

TGGAGCACTGAATTAGTGACTAACTGGCATGAGCATGATAGCCACACAAACCCCAAAAGAAAAAAAAAAAGAAAAAAAGTGTTAGACCCACACCACAATTAGGTCGGAGTTGGAGGTAGTTAGCCCGACAAGTGCTGCTACACTTCACATGGTTGTCTGTGCTTTCCTTCGAGTTTTCTCTGCCTTAATTACACATTCTCTCTCTGCCCTTTCATTTACCATTTCTAAATCCAAACAAAAGATTTTGTTAGCTGCGTGTGAATTTGGGGGCCACTATCATATGGATGATCTAGTTTGAGTTGGGTACACGGTCCGGGGACTTAAAAGCTGAAATACAGAGATAGTTACGGGAAAATTACTTTGAAACAGTGGAAGCTTTCAGAAAGAGGAAGATTAAGAAAAGTAGGACCCAAACACTAACCATCCATCATTATTATTTGGATGGTGGGAATTAATTCCCAGGAATATGTGTTTTCACGTCTGTAATTTCCATGAAGTCCTTACTCCAACAACGCCCGCATCATTCCCTGCATTTAATTGCTAGCTGTTCCATCCGAGGGTGGGAATCATTTGAAGACGAGACTAGACGACCCATGTGGAAAGCTCCTGCTTTTGCGGCTATTTTACTTTATTTTCCTAAAAAGCCAAGAATATAAAATGTTGCCCTCTCCAAAAGTGAAATCTTTTCCTCTATTCGTGACTATGAGGGGGAACAGGCTGCACAAGAACTTGGCACTGACGGGATCGGTTGCTACAATTTCTGGAGTTGGAAAGTCGTTAACAATTAACAATCTTTTATTACAGTTTTAATTTTTTAATTTTTTTAAAATAATGTTAACATTCCTTGGTGTGGTGTGGTCCAGCGCTCATAACTATTGACTCTGGCCTAGAGCGGAACCGAAGGCCACGGCCACCCAATTATTAAACACTCACTATGCAAGCTATCGATAAAAAAAAAAAAAAAAAAAAAAAAAAATCCAAGTCCCTCACACTATTCACTGGAATCAATCATTTCCCTTCTTTTATTTCTGGGGTTATGTTATTAATATCATAATGAGATGGATCTTATTTTAAAAAAAGAAAAATATATAATTTGTATTGTATACCCCTTTTCCTTGTTCCGTCAGTCCAGTTGATGTCGGCCTCTGTCTTCCTTCCTCTTTTTCTCCCAAACCTTTTTGCTTTAGCTTTACCTTAGGCCTTTGATTTCCCGGCGACAAGTCTGTGCTGTTTCATCCCCGCCACAATTTCGCTACCCACTTCTTTCCTTCACAAGCCAAACTGTAACACTATACCACTTCTGTCTCCCCCTTTTTGCTTCTTTCATTTAATTTTTCCTACGTCTGACGCGGTGGACTTGCCGGCGACTTTTGTCTTCAAGGAGCTGAGCTGAGGTAGGCAGAGTCCGACTTTGCTTTTTTTTTTCTTCTGTGGAGGAAGCATGTCAAGATTTGGACTCTGGGATGGACTTGGACGCAAAAAGCGTTTATTGTAATTTCCGCGGATTTAACAAGCTCCATCTTTTTTAGTTCAATCCCATTACTTAAATCTCTGGTTGGTTGGAACTTCAACGAAACCTGTTGTTTACACTCGTAATTACTATTTACAGCCATTTGTTTGGCGCCTTAATGTCTGTACATCCTGGATGCGCTACTTGGGAAGACTCTTTTGCACCTAAGTTCTCCTATATGAACTAGTTGAAATTGCTGCAAAACTGATAATTCTACTAAGGACTTGGTTTCACTCAGCTACATCCTTAGGGTTTGAGTAAAGGCTTTTTATACATAGTAAAGAGGAGGAGGCTTCTATCTTGGGATCGGAATAGAATAGCGATGCCGCTCCTTGATGTCTTGTCAATTTGGTGTACTAATGATTTGCAAAATGGGCTAGGCCTCTCCTTCATATAATATCTACTTCTGCTGCGCTGCATGGGTTTGTTCTTGCTGGTACGAAGGTGGTGATCGGATTTCCTTTTAGATTTGTCGGTTTTATCTTACAAT

**>CsARF8**

GGGACTATATTTTAATTCAAGTTATTCAATAGTACGTAATTTTAATATTGCATCATGCCTTTAACCCATACCCATTTAATCGATCTAAAATATTAAATGGATATAAACTTTTGCCCTTGATTGATTTGCCACTGAGTCCATTGGTACTTTTATGGATATAGAGACCAATGGTTAGACTTCCTTGAGTAGTTTATCATTTTTTTCCTTTCCTTTTTTTTTTAATTGTTATTATTTATTATTTTGTAAGTTCCTTCTTGTCATGTTCTTTCTTCTTGTTCTTGTGGAAACTTAACATCTATGATAGTATATTGAGTGTAATTGTTAACGGGGAAGTCCACTTTCTTCTGAAATGATCTCTAGATTGGTTTCACTGCCGCTCATAAACACTTCCCAAGTCTCATTGTCTAGCCTTATGTAAAGACATCTCCATTGCAAATATGTGTAAGAGGAAGCAGCCTCCTGGGATTTGGGCTTTCATCTAATCATAAGGTTAAGCTTTCGGGTTTAACCTATCCACCATTCTTCTCTCTCCCCAAAGACCATTGGAGATGGTCTCTAAGTCTCCGTTTCTTATCATATTCTGTGAATTTTATCTTGCCTTCTTGGAGGTTGTTCTTTTCTTTCATCTTCTCTCAATAGAAGCTTGGTTATTCATAAAAATAAGATTATGATACCATGTAATCTTATATTGCAAGAGTTTGAATACAAAATCTTCTGCTTTGGTACCATGTAAAATCACTTAACTCAAAAGTTTAGGCTTTAGGGTAATGAGCTACAACGAATTTAATAATTTCAATACTCTAACGGTATCAATACGATCAACAACTTGCATAAAAAGGCCCCTTTCAAAATTGGTGCATTCTTAGTCATAAAAGTGGAAACCATGTGCTCCTTGTCACCTCACTTTGCTAATTCTTTCTGGGCAAAAAACGTAATATAATGATTTTCAAGGAAATTTCTAATAAAAAGTATAATCTCCACAACAAATGCTCATTCAAAGGAAAAGAATAAGAAATAGTATTTCAGTTAAATGATGTTCCGCTGGGGTGGGGATCTTATCCCCTTTGTTCCAATTCCCCATTCCCTGCTTAATCAAGGATTAGGATTTTGGAGCAGGTTGCTGGTATAAAAATGAACATCTCCATCTGTATTTGCGGTTGAGAGATGGAGGGTGGATGGGTGAGAGAAGAGAGTGAGAGAGTGAGTGAGCAATAGCATTATTGGCAAGAGTACTATTAAAGAGGCTTTTGCCTTTTATGAAGGCTGGTTCAATTTTTGGTATTACCTGCTTGTATTTCATTCATTTTCTCTTTGATGGTATGGTTTTCATAAAAAAAATAACTAAATCTAAATTCAATTATTTATTATTAAATTAAGTAATTAGTATTTATGAATTACTGATGCACCATGGAACCCCTTGCCTGCCCGCCTCAATTGCACAACAAATGCCTCGTAAAATATTATTTCAAGTTAGGCTTTAGATGGATCAAATCATTTATAGTACTACTTTTTGGTGGAAGAACAAGTCTTTTGTTCCATAATGAAGGGTAGAGTCACAAAGTGGCTGACTGACACTGAGGATGCAAGTCATTGTTTCTCACCAACCTTCTTATACTAGTATTTCCATTTTTAGATAATATAATATATATAACCTCGTTTGGTTTTTGTAGGTTGGGAGGATGTCTTATTTTCCCTCCTTGTACTCTTTCGTTAAAGAAAAGTAAGTTTTTTAATCAATACATCCACTCACACCTGATTATCAAATTATGAAATATACCTTTTACTATTTATAGTCTGGTCCTGCTAACTGCTAGTGTCTCGAGGAGGGGTAGGTTGACAAGTTGTTTATGGAAAGAGGTAGAAGAAAACAGAGAAGAGATTTATCTCACTTGATGAAGTGGGTTCTCCAAACCCACATCTATGGAGCTGTGTAGTTAAAAAAAAATCTTGAGTTTAAGATCTGAGCCATCCTTGGGAGACGATTCGTGCGATTACTGGCC

**>CsARF9**

AAAAGTTTGAAGTTTTTTATATGGTATGCGTTTTAATTAGCTGGAATCACGCCAAACTCGAGCATGAAATGACGGTAACGCTTCTGCAGTTTCCTTAAAACGTCCTTAAAATATGGTATGTCGAACATCTCATTTATACGTTACAACCACGTAAACTTCCAAAAAAAACAATTGACAGGATACATAACAATTATATGAGATTAGAGTTTATAAAATCCAAGTCGGATTAAATGCGTAGATTAGACGAACAATAAAACTATCAACCACAAATACTAAACAAACAAAATCAATATATCATTTACATATCTAAGTAGTATTTATGTTGTAGTTAGGCTTCAATTAGATAAAATAAATCCATCATATATAGAGTAAAAAGACACCAACCTAAAAATATAAACTTAACTACGTACTAATAATTATAAAAATGGAAAATTATTAAGCAACGAAAATCATGTTAAGTGGGATGAGATTTGTTTTAAAAAAATGACAATTAAGAAACATAATGTCTTAACTAATAGTTAAATTATGTAGTAGTAATAGTGATCTCGTATTTAATTAAGACTGAATTATGTTTAGAAGTAAAGAAATGAATAAACAAAATCCTCAAGCATCAAATTAAAAACAAAGAGAAATGGGAGATTAAAAAAAGTTGGAAGGTGGAGTGGATTTATTCGATGAAAACCGATAAGCAAAGAAAAAAGGAATTCATAAGAGAAACAATGTAGTGTGATCGATATAATGTTTATTCTTCTTATAACAAAATCATGGATTGCGACTCTGTTTATTAGAAAATAAAAAAGAAAGCGAAAAAGACAAGTAGAGAGAGAGAGAGAGAGAGAGAGAGAGAGAGAGAGAGAGAGAGAGAGAGAGAGGGAGGAGAAGAGGCAGCTGAAGCAAGAAGGCACCGGTATAATAGGATGCGAGGAAGGTTTTTTTGGCATTCTCTGTAACGGCGTTTTTCCCGGATTCCCAAAAAACAAAACCCAGAAAGAAGAAAGTTGAACAAGGTGAAGAAGAGAAGCAAAAAGCGGTTTTTAGAGTTGAGTTTTCTCCTTAAAAACGCTTCAGTAACGGCAATCCAAAGAGGGTGTGTGAAGAAGTGGAGGCGGGTGGAGCGTTACAGTTTTTACACTTACTCAACGGACAACACTTGTACTTCTTCTTCGTCGTCTTCTTCTTGGGAAATCCTTTAAATCCCCCATCAATCTTTCATTCTGCTTCAAATTACAACTGGGTTTTATGTGTTTCTAGTTGTTGAGCCAATACTGTTTCTCCTCTGTTTTCTGTTGTTGACTTGTGGGGGTGGGGGTGGGGGGGNNNNNNNNNNNNNNNNNNNNNNNNNNNNNNNNNNNNNNNNNNNNNNNNNNNNNNNNNNNNNNNNNNNNNNNTGGGGGTGGGGGGGGTGGGGGGGAAGCTTTAATAATAATGGCTAATCGGGGTGGAGGGTCGCTTTTACCCTCAAGTAGTACTTCGATTCAAGGTAATTTCAAATGGGGGTTTCTTGTTTTGTAATCTGATTTTGGGTTTACTTTCTCTAAACCCTTTTCTTCTCTGTAAAATTGTGGTTTCTTAGGTTGGTGTGGAGATGAACTTTATACTGAGCTATGGAAGGTCTCGGCTGGACCTCTTGTGGAAATTCCTCGAATTAATGAGAAGGTTTTATATTTTCCTCAAGGCCATATGGAACAGGTAAGTTCGTTGAAGAATCATTTTGCTGCTTTTCAGATCGACCTCTGCTGCTGGATTTGTGTTTTATGATTTCTTTCTTTGATGTGTTTGGTTATAGGGTTGTTTTTGTTGTTCCCTTTTTGAGTTTGATTGAAGTTTTTTGATTCATATAGTTGGAGGCATCTACAAATCAGGAGCTAAATCAGAAACTTCCTTTGTTTAATCTTCCCCCCAAAATTCTTTGTCAAGTAGTTGATACCCGGCTGCTGGTACCTTCTTTTCCCAATCCTTTCTTCTTGTTCTTGTTGTTCGTCTTTTTGGCTTTTGAGAAA

**>CsARF10**

GTCCTGAAAAAAAGAGAGTGAGGTCACGCGTCAAGAGGAAGAGTGGGAACAAAAAAAGCGGGAAAGGGGAAAAATAGAAATAATAGAAATAAAAAATTCGAGCAAATCGATGTTTGGGATCCAATAAATAAAATTACAAAAATTAAATTAAATTGCTATTTAATTAATTATATATATAGAGAGATATAAAATTTTATAGAAAAAATTGAAATAACAAACAAGGAAGAAGAAGAAGGAAAAGGACACCTGAATGAATGGCTGAATTTGTGGTTTTCTTGGGATGTGGTTGTTACACTCTTTGAGATCTAAAAAATAAAAATAAAAAACAAATATTAGAGGTTTTGATATTATTATTTTACAATTTGGAAATGGTAATTTTTTGTTTATTAATGTAAAAAAAAAAGATAAATATTGGATGTGTTTACTCGGCGCTGTTCCGAAAAATTTCCGAAAAAAATCTCATTCCTTACTTTCAACTATAATGCGAAATGAAAGGAAAGAAAAAAGGTGTTTTGTTACAAAAAAAAAAGAAAGAAAAGAAAATAAAGATATATGACGTCATATAGTTAAAAAATAATAATTAGAAAAATATCGAAGAATGTTTTTATTTTTACTTTTTTTTTAAAAAAAGATTTGCAATATTTTTGTCTATGTTGAATTGACTACACTTTTAATTAACTTTATGTAGTTATTAACATAATCCAATTGACCAAAATAATGATTTGACTTTAGAGTTAATGTCACAAAATGATGGTGAAAAAATCACAAACTTATGCAAATAGATATTATTTGAAAATAATAATAATAAGGAACGAAAATTAGTTTAATTAAAATATCTAAAATGAGAGAGAGAGAGAAAAAAAAAAAAGAAAAGAGAGATGGAATTTGGGATTAAATAAATAAAGAGAAAATGAAATCGACAATATAAAAAAATAAAAAAGGAAGAGAAAAACAATTGAGAAGAGAATATAAATAAATCTCTCAGTTTCTCACTGGCAATTAAAGTGAGGTCGGCGAAGAAGAAAGAGTTTTCAGGCGAGTGTTGGTGAAGGAGGAAGCACTTCATTTCTAGCTCTCTTTTCCTCTGTACGGACGGTCTTAATCATAATTATCTTTATCAATTCTTCTCTTTCCCTTCTAATTTCAGCTTTTTTTCCTTTTCATTTCTTTGTTGTATCTGTGAAGTAAATTAAGACGGTTTTTTAGATTTGTATTATGGGTTTCACTCAGATTCTGTAATAACAGCTTGACAATTATTGAGTTAGTCTAGAGTTTTTTGCTTCTTTTTTTTTCTTTCTTTTTTTTTTCTTTTTCTTTTTGTATTTTTGTTCCGTCCATTTGGGGATTTGGGGAAACGATTTGGTTGATTGTGGAAGAATCGGTGTGGGTTTTGGTTTGGAATCTTGAGTTCTGTGTAAATGACAGCGAATTTGAGTTTGGGGTTTCACTGAAGTTTCATTCTCTTCGATTCTATTTGGATGTTATTTCGGAGGAGTCAACTTTTGATTCAGGATAAGAGGTAGGATAAATTTTTGTTTTTTGTTTTTTTGTTTTATTTTTCATTTTCTTGATGAATGTGTTTTTAGCTTCTGTAAAAATCACAATAGATCGACTGGTGGATATTTGTTTTTTCCTTGACTCATTTTGATGAGAAAATGCGTCTAGTTGTTTTTGTGGCCGATTTCTGATATTTTGTATTATTATTCAGCTTTTGGGTGTCTTAGAGTTTATTTTTTATTTTTTTGTTCTGATTTTTCCTTTTCTATTTGTTTGTTTGTTCTCCAGTAATCAAGCTTGGGCACCGGAGCTTTTTGTTGTTCTTTAGGATTTTTGGTATTTCCCCCCCTAATTTGTGTACGTTTATCAGAGGGTGATTGATTACTGTTCGCCTAGTTTTGTTAAATTCATTTATCTGTGAGGATTTTGATTGAGAATCAATAAGGAAGGAAGAGTTGTTTCTGTGACTTGGAGGACCTTTTGGTTTTATTACGACTAGAGGA

**>CsARF11**

CAACAAGGACGAATCTGTATGTTCTCGAATGGTGTTAATACAATAGTCTAAACGCGAGGTAATGAGACCAAGCGTAGAGAACGATTTGGAATCCTTGACGAAAAGAATAATTGACTAATTGGTGTTTATGCGAAATGTGTATTGATATGATTTGCGAGTTGATTTAATTCTATTGTAAAATTGTTTATATGCATTTTGACATATTTTGCAAAATAAAGTGTTTATTGATAAACTGATTTGTGTTGATATTTATGATTTGACGAAACAGATTTCATAAAACCTCAATAAGTCTTTTAGACTTACGTTTTAAAATTTTATATTCAAAGATTTGACGTTTAAGCCAAGTAAAGAAGAAGGTTGAAGGGCTTGCATCGAGTTGAGGAAGCCACCAGACGTTAAAAGTGTACGTTTCAGATTATGTTACTTATTGCAATTGGCGTTTAAGTGGACTCCCGTTGTGTTTTGAGTTTCGCTCGATAGTTTAAAAGAAGTTGAGAATGAGCTAGACGATAATACTAATCGCAAAGTTTAAAACTAAATCACGTCCTATCCGCATTACTTCCTACGTTTTGCCTGTCTTGCGGATTGAAGTGTGATAATCATGTGCATCAAGTGTATTAAGATTCAAAGGTTATCAAACAGTGTCAAATTTATATAATGTGTGTCAAGGAGTATTATGTATATTAAATGATAGTAGGTGTATCAAATGTATATAAATAACGAGGGTATTTGTGACATTTTACATTTTGTATATGTGAGTCGTGCTTCGTTTTTATCAGTTTTGCAAAACAATGAAGTTGTGTGGTATCATTTTTGCTCAAAAAAGTGTGATAATCTTTTAACTTTGTTGTATGTATTCATAATATATGTCCATTAAATTTGTACATATAGACTACCTTACCACATGAACTTACATCATTTATGGAAGATATTTTTAGAACCCATTTCAAAGTTTAACACCCCCTCCCAAGAAAAAAGAGAGAGAGAGAACACAAACTTATTATTCCAAACTTCAAAACTATAGTATAGTATTCAAAAACCCAAAATTTTAATTTTTTTAACCAAAAAAGAAAGAAAGAACAAAAGAAAATAAAGGAAAGAAAATTAGGAATGGCCCCCCTCCCTATTTGAGCAAAAAAAAGTTAGAAAAACAGAGCTTTGTTTGTGCAAAAATCGAAGAGAATAGAATGGACCCATTTTGAAAGAGAGAAAAAATGGTGAATGAAATGAGATGGCAGAATTTGAAAGGGTATTGAAAATTGTAAGCTTTGCTTTGTGGGACTGAAACAAAAAAACAGAGTCAGACCTCCATGATTCCATTATATTATACCTCTCTCTCCATTCCAATTCCCACAACTGTTCAACAAAATTCAAAAACACTCTCTACATACATAATAGAGAGTGAGAGAGAGAGAGAGAAATTTCTTTTTATCATAGACGGCGACAGCTCTTGCAGGCAGAAATGGATAGGCAACACAACAGAGGCCTCTCAGAAAAGCCATTTTTTGGGGATTTGGTAACGTTAAGTCAGTTCTTTTAGAGAGAAACAGAGAAATTAAAGACAAACCAACAATTTTTTTTTCCCTCGGAAGCGTTTTTCTATGCTGCAGAAAGCTTGATTTGTAACTGAAGGAGATGAGGGAGAGAAAGAAATTGAGCAGATAATAATCACAGACACAGTCTGTTACAGAAAAAAAGCAGAAAAAGGAAACGAGGGTGGGAAAAATAAACTTGTACGGTTAATTATGATTGCTTGCTTAACCGTCCTGTTTTCTTAGCTTTTTTCCTCTCTCTTTCTTTCTTCTGTGGCTTGTCCTTTGATTGATCGGTAGATGGGGTAGCAGTAGCACGACAGTTTGCCATTCTTTCAGACTGTGAAAAACAGAGCAAGAGAGAGAGAGCTTTTTCCCCTTTCTCTACTTTCTCCTCTTCTTCTTTGGTTTTTCTTGTTGTTCTTCAATCTTTGGCATTTTAGACTGAGACCCATTTTTTCAATTTGG

**>CsARF12**

CTCTTAAACTTAGTTTTTAATGCATGGAGTGAAAGATAGAAGGAAAATTGCATTTAAGAGCCTAAAATTAAAAATTTCCTCGAAAAAGTTACAAAATTTATCTTCATCATTATTTGAAAGTAAGTAAACATTTTAAGTTTTATCTTACATTTAAATTTTAACATGCATTGAATGTTAAAATTTAAGTTTTATCTTATTCTTCTTTTAAATTTCTATCCTTTTACATCCTATTATGACAATTAATTTTAACATGCAATCAATGTTTGAGGATAAAATTGAAAAGTGGTGAGACCGTAAGAAACCAAATTGGACTTCTTGTCCAACAAACTAAAACTTTTGAATAGAATGCTTAAATTACTAAATTTTGGAATTAGATTATGGTAATCGAAATTTTGGAATTAGATTATGCTTAATTTCAATTTAAGAGAAAGAGACTAATTCTTCCGACCAGACAAAGGAACAAAAGAAACGAGTTAACTAACTATAACCAACACTAGTAAATTGGTAAAGAGATATAAGAACCAAAATTTATTTGATGTTTAAGACGTGAATGATGGAATTTAATTGTTTATCATTGTTAAAATTAAATTTAAAGTTTTAGTAGAAGAATGTTTGAGTGAAAAATAAAGATTAAATATTTAAGTGTTGAAATCTAAAAACTATATTAAAATGAAAGATGAAATTGATATTGATATGGATGTGTTTGAGTGATTATGTTATTAATTTGTTGGAGTAAACATAGATAGATGGATAAAGATTAAAGAGCAATAATAATAGAATAAAGTTGATTAGAGTAACTATGGATTGGGTCATCAGTTGTATATTCAATAATACAGACATTTGGTGGACATACACATACTTAGCGCTCGACTGTAATTGACTCCAAAGCTTCCCAGTGAAAGTGAAAGATTCTTCTATCCCATCCATCTGTAGTTGGTGTAAATAGATGTGTACATATATATGGCCCAAAGGAAACAGAGGGAAAAACAGAGAGGTAAAAGAAAGAAACAAAGAAAGAAAGAAAGAAAGGGAAAGGTAGGAAGGGTAGGAAAGGTAGGGTTTAAAACAAGAGAAGAGTAAAAACAGGATTTGTCTGCTACTTCTTCATTCACTGTACCCTTCGTTTTAGGATTTCATATTCATCATCCACCATTCCATTTCATTCCCCACCCCCCNNNNNNNNNNNNNNNNNNNNNNNNNNNNNNNNNNNNNNNNNNNNNNNNNNNNNNNNNNNNNNNNNNAATAACAAGTCAAAAAACTTAACCCCCCCCTTTTCATCCCGCGATACGATACGAATCCAATCCCCCAACCATAAACACACACAACCTCCTTCTCATCTCTTTCTTTCCCTTTTCCTTGTTTTCTCGTTTTCCTTTGTCCATTTTCAGATTGTTTGTTTGGTTCTTTTGTTTGTTTCCTTCCCCTTCCCTTCCTTGGCTTCAACCAACACCCCTCGCCATTTGGGATCTATACACATACCCCCCCCGTTTTCACTCTCTCTTGAATGCTTCACAACAACCTCCGACCTGTGACGATTTGTTCTTTCTTCTTCATCTTCTCACTTGGGTTTCTTTTCAAATTTCATTTTCTGGGTTTTATTTTGACTTCAACTTTTAGTAATCCAAGATGCTGAATCTGAGGGTTTTGGGCTTGGTTATTGCATTCCCACTCTGAGCTTCTTCTTCACCTCTTCAGTAAAGGAAGCTGTATTATTGACGACAAACAAAACTGGGTTTGGGTTTGTTTGAGAGGGTGAGACTGATCTTAAGGTAACAATTAATTCTGATTCAATTATTATTTCATGCTAATGAAAATGGGGTTCTCTGTTTTCTTCATTTAATTTTATTCGTTTCGTCTCTTTTTCTTGGTCAGTTATTAAACACCCTTGATGTTGAATGCTCATTCTAGTTTGAAGAGGGGCTCTGTTTTTCTCATTAGTTTTCTTTGATTCCTTTTCCTCAGGGTTTATTTTACATTTGGGGGAAAACGCAATCATTCGA

**>CsARF13**

ACTGTTAGTAAAAAAGTAGGTCATTTGAATACATTGTAATTTTCTTTTAATTTTCTTTCAAACTTTAATCTAAGGCAAGTGTCCTTGTCACACCCCGTTTTCATTCAATAATTTTATTCTCGTTTCAATTTCCTAAATCCAAAAAGCTCCTAAATATTTGAATTGGCATAAGTTGGTAGTATGCACATATTCATGAATATCCTATCGTGATATATAGAAACATTATTATTTTAAAATTTGTTTGATTTTGTCATTTAAAATTTTCTCCTTTAACTTAAAACTCTTTTCCTTAAGCTCCTCATGTGAATGCCCATTTCAAAAGACCATTCTCTTTCCTACTTTTGTACTACATCCTGACCTTACCTAAAAGTTAAAAAGTTAAAACTCACCCATCTTTCTCATTCCAACAAACAAATTTTTTTTTTTTTTTTTGTAAACATGAAATATTTTTTATTTTTGTATAGTGAAAAAATGAAGAACATTTGAGATAGATTCATGTAATCATGGTTCTTTTCGGATTACTCCTCTCATCCCTTTGCTCACGTGATTGCATTTAGCTACAACTTTATCTTCATATCTCAATTGAGAAATTTAAATATCATTAGCTTTTTAGAAAACTTAAAACTTCATAACTTTTTGATGCTCTTGTAAATGAGTTTATATATATATATATAAATAAGTAGGTAATAAGTAGTGAAAATCAGGAGAAGTAATTAATTGGCTTCCATTTTTTTACCATATGGTTTCCCTAAATGAAGCTCTGAAAAGAGAAAACACACCCATCTCTCTCCTTCTTCACATTTTCTTTTAATCTTTTTTCTTTTAAAACCCTTTTGTTTTCTTTCTTTTGAAACAAATCAAAAGTTAAACGACAGAAAGAAAATGTAGAGAGAGAAGAGAGAATTGTAAATTAATAGAATAATAAAAAAGGAAAGAGAAAAAGAAAAAGGATAGGATGGGTTTTAAAGATCTTTGTTTTGGTGGCTCTTCAAGATACACTTCTCTCACTCTCTCTCTCTCTCTTCGGATCCAAAGAAAAAGGTAAGAATTCCCTTTTAATTTTATCTTCTCCTTCTCCTTTCTGTCTTAACATCATCTTAATCCCTTTTTTCTTTTTTTCTTTTTCTCTTTTTTTTTTTCTTTTTTTTCTCTTTTTTACTTCTGCTCTCTGCTTTTGGACTCTTCCATGATTTTGATGATCCAAACCAAAAACCTCTCTTCTCTCTCTTCGCTTCTCTTCTGTGAGTGTCCACGCCTCTTTTTATCTGCTTCTATGTCGGCCATCGACTCACTCATTCAATCTTTGAACAACAACAACAAACACCAAGAACCAAACTACGATTACCTTCTCTTTTTCTCTCTGTTTTCCAATTCTCTCTTCTTCCCTTCTTCTCCTCTCTAGGATTCTCTTTTCCCTTCCTTCCATCAATTCCCACTTGGCTTTCTTCCCTTCTCTTGATCGACCTCACTCTACCCTCCATCTCGATTTTTGTTTCTAACTCTGTTACAGTTGCAACCCCCTTTCTTCTTCCTTTTTAATCTATTTTTATCCAATTTTTGTTCATGGGTTTTGCGTCTGGTGGTATATGAAGTGGTCAAAGCACGGTGAAGAAGCTCCTAGAGAACCCCTTTGAATTTGGTGAATGGTAGGTTTCTGGAGCTGTGGAATTTTGTTGCCATTTATAAGGGGCGAATTCCCGAGAACGGAAGGGTTGTTGGGAAATTTTTGATATGGGTATGGAGATTTTGGAGACGTATTCGTCTCTTCTACCTTCTGCAATGTTCTGTTTCTGAGAATTTTGGAATTCAATTTCTGGATTTTGCTGTTGTTAGATGGTCGACGGGAAGGAGTTATTTATGTTCTACAAGGTAAGAATCTTTTAGGGTTGGGTTGGGTTGTGAATACACTTGATATTCAGTTTTTTTTTTTTTTTTTTGAAATGTTAGTTTTTCGTGGTTGGCAGAGGAATTAATTGGCTTTGATTGAGATGATTACGTTT

**>CsARF14**

TACGATACAATTTTAAATGTATGAAAACTAAATTTATAATTAAGGTTAAAGTACCACATTGATTTTTATATTTTCACATATTAAATAAATGCTAAATTTAGTAATCAATAATAGCTTATTATTTAGAAATATACTCAACTCTATTTTCTTAATTAAAAGTTATTATTAGGTTAAACGGTGAATTAGTGCTACATGATTAAAAGAAAGCTAAAATTCAACTCCTATGTTTTTGTAATAATTAGTACGTAGTTTCTAGGGTTTCATAAAATCTTTTCAAATAGTTATTTAAAACATTCCTAAATAATTTTTCCACTGTCCATCAAATTTGATCAAACTATTTTCGAGGTTATCACACCATATGAACTAAAATCTATTTTTTAAAACCACAACTAAATTCTAGCTTTTTTTAGGAGATTCATTCTTTAATTTAAAATAATTATTTTATTTAACTAATTGTGTTAAAAATGAATTTTAAGACACTCAATTTAAGATATATTAAAGAAAAATTGTCAGAAAAAGAGAATACTTCTTTTAAATGGTTTTATTCTCAATGGTAAATAGTTTGATAAAAAAGTTTTATATATCAATTTACTAAAATAAACCAAAATTTTATATTTTATCACGAGTTATAATATAAGTTTTGTTATATTTTATAAATAATTTTTGATAATTGTTGGAAAGTATAAAAACTAAAATTACGATTATAATATATAAAGAAAATTCAACCTAACCTATATAAGGGTATATAATAGATTAAAATTTGGGGGTGAAAAGAATAGAAAGTATGAAGAAAAAGAAAAGGGAACAATGAAGAAGATGGAAAAACAAGAGTTGATTTTCACAGGGGATTCTATAAAAATCAGAGATTTCTTTCCAACACTCTCTCATACTCATATTCATAGAGCTTAATCTCTCTCTAGACTCTGTCTTTTACCTTTTTCCCTAATCTCTCACTCTCCTACACAAAAGACAGCTTCAAATAATATTGGGTTTCGTTTCGTTGATTGATTGTGTGTTTTACAGAGTACCCAACAACAGAACACACACAAAGAATTCGAAAGAGAGACACAGAGAAGCTAAAGCCATCCCAACTTTAAAGGGCTTTATTTGTTCAATAAAAGCTTTCAGTGAAGTCACCCACCATTGGCAACTCCAACTCCACCATTACTATTCACTCTTCTCCTGCAATTTCAGCAACTTTCCATTTCGTTTTGTCACAATAAAAAACAACCAATCTTGGCTCCACCATTCAACCCTTTTGTTTCTTCAGTGTATACCCAGTTCATTCAATTGAACTTGGAGCCCCCATCATTATTTTCTTTTACCTGAAATCATTGTGTTTGTTTCCCCCGATGAGAATGGCCGCTAAGCTCTAGCCTCTTCTACTGGGGTTCTACTGTGCTGAGATCTGTCTCTTTCTTTGCTTCTTGCTTTGGGGTTTTGGCGTTTTTGGGGGTTGAGATCTGATTTTTCCCCCTTTTGCATTTCTGGGTTTTGTTTGATTTAACTCTGTTCTTGTCGGGTTATTGAAGGAATTTGATTGAATCGTTGGAATTCTTATCCCCTTAAGTTTATAATTATAGTTTTTTTGGTTTAACAGTGGATTTGTTGGGCTCTGGGTATTGGGTTTTGTTTGTTGAGCTTCGAGGTTGCTGTGGCATGAAGTCTCCGATTGAAAAGTTGTGTATATGGGGGGTTTGGGTCGAGAAAATTAGATGCGGGTGAGGTTGAAATTGATCGTGTTCTTTAGCGAAACCGGCTTCGGCTGAGATTTTGGGCGGTGGGGGTTGGGTTGTGATATTCCCAGATGAGGTTCCACTGTTTGTAGTTTACTTTTTGAAGATTCAGATTGATTTGCTGGTCGATTTATGGTACTTCTGTAGTGATAAGAAAGGTTGTGCAAAAAAAGTTGTTCTGCAGTCGTTGTTGCGTTCTTGGAAGATACTTTGGTGCTTCAAATTTGTGGGTCAATTTCTTTTTGTTTGTTGGATCTCACTGAA

**>CsARF17**

AACACGTCAATTAATTTATTGACATGAAAAACTTGCGTATAAAACGTTCAAAATTGACATTGTATTTACGAAAATTAATGAAAAATGTTTTTCAACAAAAGTTTCAAATTTTGGTAAATTAAAAATACTAATATTTGTGTATTGAATTTATAATACGAATTTTTTAAAAAGAAAAAAAAAATCAACAGTACGAATTAAAAAAAAAACTTTCTCATCATCTTTCTCACATAATCCCTCCCTCAAACAATAATTATCATAATCCTCTTCATTTATTTCCTTCTCAAACCATGGTTATAATAATTCTTTCCTTTTTATAATTTGTTTTCTATTGTTCCTTTCCTTCCCCAAACGGCCCCTCCAAAATATTAACCATAGCTTTGAATAATTCTTTTTAAGAAATGAAATATTAATCAAAGCCAATTCGTATTTTAAACAGTTTGGTTCTTTCAATAAACATTAAGTTCACTTTCTTTAGGAAAATTATTATTATTGTTTAATTAATNNNNNNNNNNNNNNNNNNNNNNNNNNNNNNNNNNNNNNNNNNNNNNNNNNNNNNNNNNNNNNNNNNNNNNNNNNNNNNNNNNNNNNNNNNNNNNNNNNNNTTAAATTTTGATTTGTTTTCAAATATCTAATCTGGTATCAATAGTAATATTTAACAGTTTTAAATATGTACATCATGTTGTTTAAATACGTATTGTCCGTTTTTTAAAATTACAAATACCAATTATCTTAATAAAAACGATTACATTATCACTAACAAATGTGATTATATTGTAAATATAGTTTTGTTTGATTCATGCATTGAAATTTCAAATAAAGCAAAAATTCTTACTATTCTAACATAATTTATTCTTTGTAGATTTAATTAATTTTATGAAACTTAGATGGGGAGTTGTTATAGTTTATTTACTTAATTTGGAATATTTAAATACCTAACCATTTAATCTCTATACCTTAACATTTGTAACATATAGACTATAAAATTCATCTAATTATTTTAATTTATTTAATTAAACTATTAGTTACCGAACAGTAAGTATAGAGTAAATTATTACTATTGTTTAATTGAGTATAATTGAACTGTATAAACTATATACTTCAAAATTAGAGATTTCATTCTCTCACCTGATAAATTATATAAAATACATTAAATTTTTATTANNNNNNNNNNNNNNNNNNNNNNNNNNNNTATTTTTTTTTGTTTCTTCTTATTATTCTGTCAGAAAAAGTAAATTGAATTATATTTTATTATTTAAAAAACCAACATGTTATCATAAATGAATATCTATTATTAAAAAATTTAAATCCACGAGGGAATCTAAAATTTGAATGGCTTCTAATAAGATTTGTTTTTAATTTCAATTATAAATCAATAAAATTTTAAAGAAATTAAGATTAAAATAATATTATAGTTCTAATATATTTGTAGTCAATTGTTTATAATTACATCCGGAGAATATATCTCAGAATCTTTATTAAAAAATAAAAGATAATCAAGGTGTAGGAAATAATGATTTAAAGAAAAAAATAAAAAAGTAGTAATTAAAGGGAAGAGAAAAAAGGAAAAGAAAAATAGAGCAAAAGAGTTTAACAAAAAGATCAGAATTTTAGTTAGTCTGTCGGGGGTAAAACCGTAAAATCAGTCCCAATTTAAAGGCTTTCCGTTACGTAAGTCTGTTCTTCTCCCGTTGCCGATTCCTGTTTCTCTGACCCAATACGCCATTCTCTCTTTCTCTCTTTCTCTCTTTCTTCAAAATGTTTACATATCTTTTGTAGAGAGAGAGAGAGGGATGAACATGAAGAAGAAGATGAAGACATAGTCACAGTTCTTTCCCAAATATCTCTCTCACCATCCCTAAATTTTCTATATAACAACAAAAACCCTAACACACAATTTCATCATCTCAAACTCTCTTTCTTAATTTTGTTTGCTTTGCCCTAACAATTACACAATTTTGCATTGTTCATTGTTCGATCACCGCCGGTTCAGTTCCGGCATG

**>CsARF19**

ACTCAAAAATTAACTTTTCATGAAAGAAAAAACATTCAAACCCATTGTGTCATCTTTTAATTACTTATTAGTTAATTCTATGAATTAAAAAATGGAAATAGAAACCATAAAAAAAAAATGTATCTGTCATTTTATTAAATATTTGATATTTGTTTTTTAAAAAATATAAAGCTCATAAACATTGTTTCCTCTCATGATCTCTAAGTTTTATTATTTAATTTACTTATGTTTTAAAAAATCAAGCCAACTATTTAGAAAACTAAATAAAACTAGCTTTTAAAATTGGAGTATAAATTCATATTTTCTTTTAACTATGATATAAAATTTCAGAAAAAACACAAAATTCAAGAAAACAGAAAAGAAAAAAAATGAAATTGTTATCTATCAAAGTCTTATTTAAAAAAAATAACAAAAAAAGTAAAATAATCAGCCAAATACATATATAACTTGAAAACTAGAAACACAAACACATAAGTACGAAGATTAGTCCTTTACTAAAAGGACCAAGAGTACAAAAAAAAATCTCTCAAAATAGATTTGGCCTACTTAATAACTTAAACAACAAATGAAAAGAAATTAGTTAGTGATTATACTAGTTAGTGAATTATAATAATCTATGTTTGAGGTATAGATTATTTTGGTCTACTTTTTTTAGCTAGTAAGAAAGTAGTAAACACGGTGTGAAATGAATATAGTAATAGTATAATTAATTGTAGATTATTATTGTTGAAACTTGGAAGACAAAGTATACTACAATAGTTTAGTAAGCAACTCAATTGAAGTTTGAGGCAAAGGCCGCATAAGGTTACTAAAGAAAGCCATAATTTGTTTTCTCCTTGAGTTGAAAAGACAATTAAAATGAGAAAAGAAATAAAGAGAAAAGGGGGGAGAGGAAGAGAGGGGAAGGAGAAGAGGAAGGGGATAATAAAACTTAGCGAGAAAGGGGATAGCAATTAATAACATGGAGATAGTGATGGTCAAGAAAATGGTCAAAATTAGCACATGATGCTGTCTAGTTAAATGTTTTTTATTTTTGTCCTCTTTATTTATTTATTTTTTCTCTCTTTAGATAGTGGGTTGGTTGATTATGATGGAAGGGCATTTTGGGGATTGAGTAAACAGTTTTATACAAAGATTAACCATGATTAAGGTGTGAATTAAAGAAAAAAAAAAAAAACCAAACCAAACCCCTTCAAAAAGAAAAATAGTAAGAGAAAAGAGAGAGAGATAGTACCAAAGAGATTGCCATTTTCAAGGACCCAGAAGAGAAAGCAAACTCAAACAGAAGACATAAACAAAGCTAAAGCAGAAGAAAGAGAAAGTTCTTAGTTTAATTTCAAGGAAAGAGAAGAAAAAGGGTTGTTTTTTTGTTTTTGTTTTTCTTTCTTTTTTTGGGGGTTTTTGAGAGAACGAAGAAGAAGAAGAAGAAGAAGAAGAAGAAGAAGAAGAAGAAGAAGAAGACCATTGGGAAGAAAAAAAAATGGAAAGTTTTGATGTGTTTGAGAGAAGAAGTTGTCTGGAGATTGTAGGGGAAGATAGGGAACAAAGCTTCAGCATTTGGTCTCTCTGTAACTAAGCAAAACCAAACCCTTTCTTTTCTTCTTTGTCTCCTCTTTCCATTTTTGTTTTTGTTTTTTTTGTTTTTTCTTTTTTGGGGGGATTCTTTTTAGTTTTCCGATTTGCTTTTCCGGTCACTTTGCCGGAATCCTTCGCCGGAGTTCCATAGTTGTTGTTGACCTTGTTCATTTCCTTCGTTGTTTTTATCTTTTTACTTTTCCGGGAAAATTTGAAGGACTTTCCTTCTTTTTGGAGGTGATCTAGGAAGTGGCAGAGTGATTGCTAAAGCTGACTTTTTCCAGAACTTTTGGCTATACGATCTGTTTTAGTTACTGGGAAATTTCCTTTTCGCCGGAGAAGATGAGCCAGTGAGATTTCAGTTAGTGTTTCTAGTTTTCGTTTGGTTTTAATATCTATTTCAATCACCTGTGGAAGTTCAAGTA

**>CsIAA1**

GCAAAATTGAAAATAGAGAAAATGATACTGAGTATCTTAGAGATGTAAGATGAGAAACAAAAGAGTGGGTGAAGGTTGATAATGTGGGGGCATTAATAGCAAAGAGAGAAGAAAATTGGGATGGAGCATGGAGGAGGCGCACGAGGGTGGGGGGGGATGATGGGCCCTCATCGAGGAGGTGCATCGTATTGGTATCTCCCTCCACATATTTTCATTTACAAACCAACCATTAGACATTGTGGGCCAACCAATACACATTATGTCCATTCTCTCACAGAAGAAAAAAAAAAATTATATGCTTCTTTCCTTCCTTACTTACCACAAACACAGCTTTTATTTAAAAATGATGTGAAAATCACTCAAGAACTAAATCCTCTAATAACAGGTCTACGCTAGAAATTTTCCAATGTACATGCTCAAAAATGAGAAAATTCTATCATTTTAATATCTTTAAAAAAGGTCGCGTTTTGTATATTTTGACTAGTACTAATTTGTTACTCATTTTCGAAATTTACATAATGAACATTAAGTTTTTTTAAAAAAAATTAAGCTTATAAATACTTATTTTATCCTTATATTTATTTTGTATACTTTTTACTACCAACTCATAGAAGGAGGAGAATTGAAGGGTTGTTAATACTTTTAACAAAGGAATCTAACTTTTGTTTTGTTATTTTAATACTTTCCCCCCATTTATCAAACTAGCTAGTCAACTCAAATACTACTTTTGACTTAGTCCACTTAAAGACCTCTCTTTTGTTCTTATTAATAATCAACCTTTTCTATTTTAATAAAATTTCAAATTAACTTCTTCTAGAATCAACATCTGATTGAGGTGAATTATTCTTCAATTCTCTTCTTTTTCTATTTGTATCCACATTCTTACTCTGAGTTATATTAATCCTATCAATCTATTGTTGTGCAACAGAACTTCATAATTTAATAAAAAAATGTAAAAAAATGAGATTTGAACTTGAATTTAGAGGAGACTGAGAAATCGTCTTATTTAAAGAGCATACAGTTGGGACTCAATCTCCCTAATCCTATACTAAATTTGTTAGTTGTCTATAATACATATACTCGTTATAAATTAATAGTAAATTTTGTTATTAAAAGAATTATTTTTTAAAATTTCTACGTTTAAGTTGTTTAAGTTGAGATGAAGAGGTATGAAAGTTGAGATGAATTGGAGAAATTAGGAAACTAGTTGAGATGTTGAGTTTGAATTATGCATAGTTCACTCTTGTATACTTCCTATACAATTTTGTAAACTCTCACGTTCAAATTTACAACAAGAACATGCATTATATAATAACAATCATTCTAAAAATGTAGTATACAAACCTACAATTACCATAATCGTAACAATTATAGGAATATTATAAGTTTGATATTTGAATTTGTATGGTTGAAATTGTAATAGGTATATAAGATTGAGGTGGGTTGTATAAATGTTGGGTTTGTATTCTAAAGAATAGGATGTGACAACTAATAAATAAATTAGCCAAATGATGGGTCCACATAGCTTACAACCACAACGCACATTCAAAACTTTTTACCAAACAAAATTCAAAAGGAAAAGAAGAAAGAAAAACTCACACGGCCACACGGTCACACGGTCACACGGTCACAAGGCCACACCACACCCAATTCCAAATAGGGGTATCATCGTAAATTGCTGGCCCCCACTTCACATGGCATTGCTTGTTCCTTAATATCTACGCTCCCGATTTGCGTTCCCACAACCACTAAAATGCATCTTGACCGTTCAATTTTTTCCCCTTCCCATTTGGGCGGCTTTTCTTGTCCCTCCTCTGTCCCCTCCCTTCTTCTTCCCTTTGCCTCACTCTGCTCCCATTTCTACCGACAATCCCCCATTTTCCTCTTCCTCTCCCCATTCCCTTATCTCATTCTTATCTATAAAACAACTACCCCATTTCCAAACAAAACAAAAACCCCTTTTCTCTTCTTCTTCTTCTTCAACCATAAGCCAGAGCAAA

**>CsIAA2**

AATCAATTGTCAATCTACATACATCCATGTAATTAATAACTAGTTTATACTATACAAAGTTTAAATTACATCAAGTAATTTCTCTTTAGTTTAATGTGTTGACATGTCGACATGAAATTAAAGTCTTAATTTTACAAATATGTCCATGGATATTTTCGAAATAAATTACAAAAACTAACAAAAAAGTTAGCAAATAAAAAATTAACATGTTTTTACAACAAGTCGACATATCTCTTACGTATTAGTCACATTTTGTAGATTATGTTATATTTTATGTTACGTGAATGTTTTTACAATTAGATGAAAAAAATCGATTTGATTTGCTAACTCGTACGACTTTCTTAGAAATCGAATGTTTAGAAAAAATATCGATTTGATATGTATCAAACCTTAAAAAACGTGAAAATATCGATGAAAATATCGACAAGTGATCATGTCGGACGAAGAATTCTCGAGAGAGAGAGAGAGAGAGGAAAGAGATTTTAATAAGGGATGGAGGTGAGCACATGGGAGGGCCGCTGCCCCAACCGCCATAGCCAAATAGTTTTTTCTTTCTTTCTCTTAAGAAATAAGGAGTTGAAGAGGCAATAACCATTAGCACCCACCATAGTTGTCTCACCCATAAACACCTTACACCACCACCACCACACCCCCATGTGGGTCCAACTTAATTCCCTATCCAACGGTTCTCATTTCCATCTTCTTTAATTTCTACTTTCCCTATTCTCAACCCTCAGCCGTTGGATTCAAAAGAGAGGCTTTTGTCCTTCCAATTTAAAGACAGCCTCCTTTAATTTGTGTAGCTCATTGCACAAACACCCTTACCTGCCCCATGTCCCTACCTCTTACCTATTTGATTCCTCTTGCCTTTCCCATGTTCCCTCCCCCACTCTCCTAATTTTCTCTGCCCTACTTCTTTATTTTCTTTTAATATTTATTCAATTTTTCTGTCTACTTGTACTTCAACGCGTTTCTATAAAATCCTCTCGTTCGGATTCTTACGAATATGTTTGGGATAAGAAATACGTTATTACAGTTAAATCATTGTAATAGTTTGTGTTTGATAAGTAAATTATATCAATCTAGGGTTACAATAATATGTGTTTTAATTGAAAATAATGGTAAACTTTGTACATGTTTTGAAAATAATGTTTACTATAGTTAATTGAAGATTTTTTAAATAGTTACATTCTAATTCATGGTGTATGAGTTTTGAATTTAAAGTTAATTTTGAGTAATTTTTGTTTTTTTATAAAAAATAACAAAATTGGTTACTGGATGGTTAACCTTGATAACTAAATCAACCTATAGAGTAAATAAAACTAAAATATGAAGAAGAAGAAAAGTATATACAATTATTGTTAGACTTTCCTATTGGTTTCATGGAATCCAACCTAAAACAAGTATAAGGTCAAAATATACCATTTTGATCCTCTATTTTGGATTCAATTTTATTTTCATCTTTTATATATTTAAACTTAATTTTAATCAAAATATTAGTTAACCAACACTAATTTAAAAAATATAAAATAAAAAGGTAAAGAAATCCTTTTAAATGGTTTTATTTTACTCTTTAAAAACTTACAATTTTATTCCATACATTGAATAATCTTTAGAATTTGTTTTGAAATTAATAAAAAAATAAGTTGATGTATAATCATTTTAATTCAAACTACAGACCTCTTCATTAATAATATTTTGGTTAAGCCTTTGTTAGGAATAATTCGATATCCTAACATAAGAGAATCCTCAACCACAAGATATAGAGCGCGTGGCGAAGACGCACTTGGTAGGGAAGTGGGGGAAATGATAGGCTCTTTAGCTTTAATCGGCTTCTATAAAATACGTTTGAGCGTTATTTGGGCCAACAAGCATTTCCATTTCTTCTCTTCATAACTTTCCTCTCCGCCTTCTGTTTGTTTTCCGAGAAAAAAATCATATTGTACACAAATCCATCGGAAAATTCAGCAAACCCATTTCAGAGAAGCAAAAATTCAACA

**>CsIAA3**

TGTACGTATGTAATCACAAACTTGAAGAAATAATATATATATATAAATAAATAAATAATTTAAAGTGAAATGTATGAATAGTCATTTCAACAATCATGCATGATTGAAAAGTCGTACTACATTACTAATTAACAAACCTGAAGTGAAAGAATTACAAAATACAACTCATAGACTAACCTAAATAAACTTAGCTACTCAAGTGTGTTCTATTTGCATTTTCTTAGCATTACAGTATAGTAGAGATGGTAGATTAGATATCCAACCACGAAAGAGAGAACAGATAACAATTATAATTTTATCTCAAAGCTCGTTTTAACACTTTTTAAATTACATTTTAATTTGTAAAAAGTTACTACTATTAACTAAAACTAAAAGGAATTTAAATTACAAAATTCTTAAATAAAACATACATTTTCAAATCTCTATATCCCTTCCTATGTTTGGATCATATTATCAAGTATCATCACGACAACTCATCATTAACTAATATGATAGGCGATGCCAGTAGACTAACTACGACCGAGTTGAAAAAGAAAAAGTATACCTGAGAAAAAAAATGGGCAGTTTCGAAACAACTTCCTAGATTTCTTGAGAGAAAAGTACAATCAAAATCAAAATTAAACAGTAAAACCCTAAGCTTTTGACAGCCCACATGGATTCCTACCAAAATGCTAATCAAATCAAATCAATCTCATAAATGTTAAAATTATTTTCTAAAAAATTAAAATAAAAAGAAAAAAGGGGTCCCACAATAGAAGGCCCCATATTGTTCCACTTTGGCCAATCAGTCCAAGTGGGGCGCTCTCATCACATGCTGCTGTTTTCTAATGCCTTTGAACTCAATCCGACGGCGCAGGATTAGGGTCATCAGAAAGACTCAATCAGAACCATTGGTTTATGGGTCCAAGCTCTTTTAATACAAATGGCTGTAATTGTCTATTGCTTGTCCCCTCCACGTCGGACCACATCCTCCACCCTGTCCTTCTTTTCGGGAACATTCATATCTGTCGCTTGACACACACGGGATGCTCTATTCCACGTGCCACCGCCATATACTTGGTCCTCTCCCCACGTGTCACCATTCTTGTATTTCTTTCTTTTTAATATTTTAGTTAAGCTTAATTTTTCTTATTTTTATTTTCTAAAAAGACCAATTTTAAAATTCATCGTAATTTTAGAACTTCAACTTATAAATATTGTTGCCTATTTGTGTAGAATTTAACTAATTTTAAGATAAATATAAATCTCCTCTCCACGTCATTTACAGCACTCATAAAAAAAGGGTAAGATTTAGAGAAATAGTTTACTTAATGAAATAGAATTTACAGAAATGTTAACAACGCTCTATTTATGTATTCATTTTACTTCTTCTACTTAATATTTATTTAATTTAATAAATATATTTTCAAGCGAAAGAAAGATAATATAGGAATACTTTCAAAAGTTAGAAAAAAATACTATTAAATAATAAAATATATATCCTTTTATAAAATCAATAAATAAACAATAAATATGAATTTATTGAAGAGAAATGAAACCAAAATTAATAGGCAATATCAAAATTTACTAGTTAGTCTAGTGATAAATTTATCTTTCAATATGTTCTAGATGTACGTTTGTCACACTCTTTATAAATTTATTTTGAATTAAAATGTACTCATCAACAAATTTTTATAAACAAAGTAGAGAGATTGAAATATAATGATAATAAGATTTTTTTTTCATTACAAAATTTCATATAATTAAACAAAAAAATTAAATACAATGGATAGGAAAATAATAAATGAAAGAGGGGACACGTGTCAAATAAGCGATGGTGGAGTTCATGCAGACCATACCGTGCGTGTTCCAGAAATGTGGTTCTTCCCATTATATATATTCTCTCTTTCATTACAAGCTTTCACAAACACGAAAGAAAGAAAGAAAAACCTTTCCACATTATCATTTTTATCGTTTACTCAAATTCTTCATACCC**TTTCCAATTTTAGTTTACAAATCTA**

**>CsIAA4**

AAGATAAAGTTTAAAAATAAAATCATTATACGATCATTATAATAGATAACAATTTTAAGAATAATAATCAAGTAAATAGTAACATTTTAAAAAGTTACAAATATATAAAAATATATCGGTGATAAACTTATATCATTAATAAGCTTTTAAAATTTTACTATATTTATAATTTTTATTATAATTGATAGTCATTTTAAGAATAATAGTTAATGATGTAGCAACATTTTAAAAATAATTGCAAATATAGCAAAACTATCGAAAGTCTATAAGTGGTAGACTAATATTTACAATTGATTCTATGGGTGATATAATTTGATCAATTTTGCTATATTTATAAATTTTTTAAATTGTTGTAATATGCATAATTATTTTAAATATAATCGTTAAATTTATAACTATTCTTATTTTAAATCTAATTTTTATATTTGAAAATATAGGATGGTGAATGCACTTTTATGTTATGTGCTATTCCAATAGAATGGTGTCACGAAGAAGATATAGAATTGGATGTAATCATGGGTGGCAAAATAATTACAAAAAGGGGCGTTAGACGTGGCCAAAAAGGCAACCAAAGAATTTAATTTGATTTGGCAAAGTTAGGAAGGCGGAAAGAGCATTAGAAAAAGGCCAAAGAATTTCCTTCTGGCATGCCTGTCCCCAATCAGAAACGCACACGTGGGAGCCACTCCAACCACGTGGGATGTCTTTGCTAGCTACATCCAACGGCCTAAAATCCATCCATCTAAAACCAGAATTTTAAAATTGAATCCCAGCCGTTGATGATGGGCTGGAATCGTCCCTCTCTTGCACCCCTCATTGGCTCATTTCGCGGTTGTTGCAACTCTGCCCTACAACTTCACCTCTCAGCATTCACTCTCATTCCCCTTCTCCTCCGCCTCCTATCCCACGCGCATCCTCCACCACAACGGTATCACTTCTAACCTCTCTCTAAAATTGTATTTAATAACTCTACATAAATCAAGTTTTTTTAATCCTTTAGTTCGTTACTTGAACGTTGTAATCATTGACACCAATATTTGGAACATATCCTTTTTTTTTTCTTTTTTGAAAAAGATTTTAAAAAGAAAAATAAGTTTATAACGTATATAGACAATTGATGAGCTGGAAAATGATGGATATAACCTAATATGCAGTTAGACGTTTTTCATTTTTCTCTACTTTTAAAGAAGTGAGTATATGATATGTTTTTGTCATCAACTTATTTGGGTGAGAGTTTGACTTGATTTCCAAAAGAAATATAGTCTTTGGTAGTTAATAAAGAATTGAAATTCGTATATTTAGAAAAGTTTTTTAATCGAAATTGATATGGTTATTGTTACGTAGGAATATACTTTATTACATATTTGTTTGTGACTTTGTGGGTTGAATCTTTAACTTATAATTAATTGAGTTATTCTTGGATTGTCCATATTAAATTAAAAAAATATCACTTTTTGTTATTTAGATTTGAACTCAGGAGCGTTTAGGGTTTAAAATTAGAAAAACCTTTGTCCATATTTGAAAAAAATAGGTTTTAAAAATTAGATCTATAACTCAAACTTACTGTTTTTGCAAAAATGACAAAAAACAAACAAACAAAAAAACGTGAAATGTTAAAAAAGTGCGAGTACATATAATACATTATATATACTAAAAAAATAGTAGTGTCAAATAAAAAGAATTTATTAATAAGAGACTGAAACACAGTTTTACCATTAAACTAACTACTTGTATCGGTATTACTTTACAAGGTCGAGGGTGAGTCTTAATTTGGGACGTTTGCAATTTTTAAATAAGAAGGAAGTGCTCACGGGTATAAAATGGGGCGCGTGCGAAATAAAATACGCGTCCTAATAAATGCTGGCGAACAGAAAAGGCTTCGAGCACACACACAAGATGTCACTAACGAACTCTTACAAATAAGCCAACATTCCAATCCAAATCCCTTTCATCTCTTCATTTTCCAACGCATTTCGCTCCAGTTCTACATCAATCAAGCC

**>CsIAA5**

ATGTTAATTAATGATGCTCAAATTAGTTTAGTCTTTTATATGTGTGTATATTCACACTAATATCACTTCAGAGACGTAATTAGTAGGGTCTGAGTTATTAGACTTAATTATTCTAACACGATTCTTCTCTGCATTCTTTTGAAAAAGTTTCTCTCTTAATATTGCATATAACTTTGAAGTCATATATATACATATATATTTACTTTAGCTAAACTCTTAACACAGATCAGAGTTGAACAATTTTATCTATCTCCCTTTGTTCTATATGATGTTCTCATTTATATACAATAGTGATGTTTAATAATCATATATTTTAATAATTTTTGGTGATCAATAGTGATATTACTCTCCTTTTTTTTGTTCAACTATTCTTAAAAAACAAATATGGTGTACTACTTTTTTCTTAACAACATTGTTATATGTTTAAATTCTTTGTCAATACTTTAGTTACATATAATAATTTAATCTATATATTTTCAAATTTAAAATAATTTTATCACTACCATATAAATTTCCCTCAAAATTATTTAGTTTGTCCATCTCAGTAAAAAAAATCCATTAAAATAAAAAGAAAAACTAAGAAATTTCATGATATTACTAGAGACTTTTTTATATATAAAAAATGAACAAAAACATAATAAAATATAATAAAATTTTAAATTTTAATAAAGGTAATATAATTGGAAACTTCGATTTAATAAAGAAAAAAGTCAATTATTGTTGAGTTAAATTCTCTTCCAACGTTAGTTTAGGTTAAGCTAGTTTACATGATTTTCTTTCACTAATTAAAATAGAAGGTTTGATACCTCATTCTCTCCAGTCATCGTACTCAATAAAAAAAATATATAGAAAAAAAATTAAACAAGAAAAAAAAGAAGTAGGTATGTGCAATATATCCTAATTCTTAGAAAGCAACATATCAAAACCTCCTTTTATAGACTATATACCACTCTATATTGCTTACTTGTTTCATGTACTTGTCATATGTTTAACCTACCCAATTCACCTTTCGAAATTTAGGTTAAATTATATCAAACACTACTAAAATTACTTTAAACTTTGAATTTTGTTAAAAGAAAATATTACAAAATTTTAAATTTGTCCAAAACAATATCTTTCATATATTAAAATAAAGAAGATGACAGAGAGAAAGGATTCGATTTTGTTATCAATTTAATATCCTTCCAAAGACATATTGTAAAATTTATTTAGATATAATCAACTTTTACACATGAATTTATTAGAGGGCATTACTTATCATGCTAGTTTATGCATAGATAATGTGTATTTCTTTTATGAAAAATGATGTCATGTATGCTTTTCAATTTTGACACAATAGCAATCCATTAATTTTTACCTTATCTTAATGCAAAAATCTTGCTAACATATATTTTGTTAAAGAAAAATAAATTATAAAGATTTTCAAAGTAAAATCCCAAAACCTAATTTAACAACCCAATTTTTGAAAAAATAAAAAAGAGTAAATAGAAGTACAAGTACCAAAATTCTAGCTACCTTATATCTTATAATATACATTCTCACTTACTAAATAATTAAGAAAGAAATTTTTTGAAAAAAGGCAGTGAAAGCCAAGAGAAAGCCAGAGAAGACGGTGGATGGTTTTTTCTTTATATGTATTATTATTACTATTTCTTTCTTTCTATTATCCCTACTTCTTTTTTTTTATAATGAAATATTAACATTACCCACCTGTCATTTATCCCACAAAACTTGGAAGAATCCACCTCACCTTCACCTTCCACATGTCACATTCTCACAAGTCCCCAACTCCAAAGCCCCAATCCTTTTAAAAATTAAAATAACCCACCTCACCCCCATGTAACTTTAGCACACAGCCCCCCTCTCACCGGCGGTTTATTTTCATATTGTTTCCATTATCATAACCACATACAAAAACCCAACCATACACAAAAAAAAAAGCTCTCTATCCTTTTGCAACACTCATAATTCAAATAGAAGAGAGAAAAAGAAAAAAGTTT

**>CsIAA6**

TAAATAAATTACTTGCTGGAAGAGATAGGAATGGCGACAACACTAGAGGACATGGGCAGTATTTTAGGGTTTTCTATCGTTCGCTTTCTCTTCAAACGTGGAGGAAATTTATGTCGTTTGCGTTCACAGATTTGTGTTTGGGAATGGAATTTAAAGGAAAACTCCCAAGGTAATCTAATATGTGTCGGGAAGTTCCCTTAATTGTCACGCCTTACTGAAAGCTTTGGCAGTTGTCAAACATCTTCTGACGCCTACACAATACGACGTCGAGAGATTCAACTAACTTTCGATGTATTTTTTATGTGTCGGAAGATATGGTATCTCCCGACGCTCCATTTGTTGTGTCATAATATGTGTCCTATCTCCCGATAGTGAAAATCTACGTCTGGAGTTTCAAATTTTCTTTTAGTGATGGGCCACTCCTTTCGTGATTGACTGATTTTGTAATGAAACTCTAAACTATCAAATAAACTAAGGGTCCGTTTGTATTGAATTAAGGATTGAATTCAGGAAAAAATATTTTTTAAGAAACTCATTTTTGTTTAAACACCTTTTCCTAAAAATTGTTTAAAATACACTTAAAAATATATTTTGAATGTCCTCCAAACACTTTAGTTTTTTTTAAAATAACTTACTTTTTAAATTAAACACTTGAAAATGTAATCTAAACACACCATAAGTTTGTATCTTTAAATTGCTTACTAGTTCTCTTATGCAATCTCATGTCTTCTTGACATTTGTTTGAGGAGGGTGACACGATTGACGCATATACATATTAGTTACATAGCTATAGGTTTGTTACAAATTGTGAAAGATTCTAATATAGGTTTGTCTTTTAATTATATCTTTCAAAATTTCAATATATCAGTCTATAAATATTTTTACAATAAAAATGTATTTTTAAAAATGTGTTTTTTTAATAAAACGTAGAAATAAATAATGTGGGGTTTTTTTAAAAAAAAGAACAAAACACGAAAATATTTACATCGTATAGAACAATTTTAAAAAAGGAAAATGTCCACAACGTGAAATAACAAAAATACATCTGTAATTAATCGATCACACAAGAGTGAAAAATGATTTTGTACTCAATCTAAACGATCTCGTAGCAAGTCTAAATGATCACGTACCAAGAGTCTAAACGATCTTGTACACTTGTACCTGATCTAAACGATCTTGTATCCAATTTCCAACGATCTCGCACCCTTGTACCCAACCTAAATGATATTGTACCCTTGTATCTAGTCTAAACGATCTTATATCAAATCTAAACGATCTATTAGTGATAGTGGTTTGTCTCTGTCTATCTAGGATATACAAATGATCGTTTAGATACTGGTACATTAATCATTTAGATACTGGTACATTGATCGTTTAGATCCTGTACCAATAACATTTAGATCTTGTACCAAGAAAAATAAAAAGATGATAATGAAGAAGTGGAAATATGAAGAATGAGAATTAATAATATAAAAAAGAGAAGAATAAATCGAAAAGAACTGAAAAATCATCTCGCAATAATCAAAATCCAATAAAAATAAATATGAGATTTATGAAAAAAACAATTAGCTTCGTAGCCTTTTATTAAATTGTCATACGAACCTTAAATATTTAGCACTATTGTTATATCTATCGAAATTTACCAAAATAACAATATGGGGGAGAAAAAATATAATAATAAAAGAATTAATTAAGAGCATGGCCCCCTTGTCCCAACACAGAGGTACCTATGATTGAACCAACTTGATTTCATTGGTTAAAATGAGTGAGTCAACTTGGTTTTTAATAAACCGTGAGTGTACAACTTATAACCTAAGAAAAATAAGAAGTATATAAAAATTCCCTTTACATTATATTCTTCTCATAACCCACTTTATAATTTCACACTTTCCCTTATTTTCTCCTCCTCCCCTCCATAACCACTCCTTTTCCCCATTCTTTCACCTCCAAAAATTAAAACAAAACAATACAAAAATATTGAGATTGTGAAGATACTCAAA

**>CsIAA7**

TTCCAACTTTGAAACCGTGTTTACTATTTTATACACCTAGACTAAAAAAGTTCACCTCAAACACAAATTATTATAATGTATAAATTATATAATAATTAATCAGTCGAACGTTTTAAACAACAACTAAAGCTTTGTACTTTGGGGTCATGGTTGAGCGAGAGAGATAAGAAAGGGGAGAGCTGTTTGGGAACGAAGAGGAGGATAACCATAAACATACATAAGGACGAAAAACATTACATGATTTTGACTCGCATATAAAAGATATATATAAAAGTGAATGAGTGGGAGATATATAGTGGGGGCTTAGAGAGTAGTGTGTTTGATGAAAGAGAAGACGTACGTGTGGTTAGGAAGGCATTCACATCAGAGATTTAATAAAGGATTGGTTAGAGAAGAAACATTGTGAATGTGTATATATGTGTGTGTATATATATCAAAGCTTTAAAACAGAGAGAGAGAGAGAGCAATTGAGGGGCTTTGAGTTTTAATTTGAGAGAGCGACGTTGACAGAGCAAGGTTACCTGCTACCTTTACAAATGTTTCCTTCTCTCTCTTCTTAGATGTGACTTGTCTCTAACTTACCACCATATTCTTTTAAATTTAAACATACCTCCTCTCTTCTATTCAATAAACCCTTCTTCTTGATATTTTAGCTCTAACATTTTTTTACATCGTCTCATGTTGATGAAAACATTTACGCGTTTTTGTAGGAAAATAGATAGACTAAAATGAACGTTTTATGTATTTGAGATTGTTGTGTTTGTTATTGAGACTATACGATCAATTTGTGTGCAAGAATTTTGTTAGACTATAAGTCAAGGTTATTATTATCACTTTAGAAATGCATGGACTAAAATTGACTTGTGAAATAATCATGAATGAACCAATAGTACGCATTTATTTGAAGAGGTTTTCACAATGTGGTTGACAGTTTGTATTGGATGTTTGTAAAAGAAGAACACGAACTGAATCCATATTTTAAGATTACCATGAGAATAGTCATTGCGATCGTAACCTCCTTATTGAATATTATATATTTTTTCAATAAAGATGTTTATAAGTAATATATAAACTAAAATAGATAAAAGGAAGAAATTATTCAACGATAACTTTAGCTCATCCTAGTGTGTGTTTTGATTTGTATATAAATGTCATATATGGAAATGACTGGCCATTTATTCTCTCATCCTTTGTTTGTTACCTAATATAATATTTGTTTTCATCCTTTAAGCCAATAGAGTCTTCATATTTTCACTTTATCGGATTATCAATTCTCAATTATAAGAGATTCTAGTGTCCATTCATGATGGTTTAGAAACACAGTGGTCTGTTTGAATTGATTTTTCAAATTCCTTGAAAGACAAGAAGTATGGTAGAGTGAAAAAAACATTTTATAATAAAAAGCTAAAAGAAACGAATGGAAAGTTGTGTTTGGTTCAATGTTTTTGGTGGTGCTAATACAAAGAATGGTTGTTAATTAACATCATGATTTGGATATGATATAAATGTGTACTAGCTAGAGTGAGATTTAATTTGGAAAGAAAGGGTGGGTGTGTGTAACATTGAGAGGTTGGGTGGAGTTGAGTACATGTGATTAATGGTTGACTAATTGGAATTAGAAGAAAAAGATATGATGAATCATAATTGGAGGCAAAACCATGTTACCTTGGCTCCCTCCAAAACCAACCACAACTTGCCCAAATAAAATCACATAATATAAGTTTTTAGGGGAGAAGAAGTCTCAAGATCAACGGTCCTATATTTCTCCACGTGGCAAAATCCCATTAGTGAGGGAGCTTGATAAGACATGCACATGCAACCACACGCTACACAAACTTCCACGCACCTTAATATTATTTCTCTTCTCCTCAAACACTCCCCAACCCCAAATTTCCAAATTATCAATAACATAAAAAAGAGAGAAAGGATCAAGAAAGTGGCAAGCCAAATTCTAAAGCTCTCAAATTTTCTCTCCCTTATTACCGGAACAACAATTTTCC

**>CsIAA8**

AGAGTAGAAGAAGGAAGTAATTATAAGTAAGAAGGAGAATAAATGAAATGTTTAAAGGGAGGGGAGGGGAAAGAAGTAGAAAAGAGTTGAAAAGTGGAGGGGAGAAGGGTAAAGTGGAGATTTCGAGGTGGACAAGCGCATTTTTGGAGTCGCCCAACGAATTCGAAGATGATTTTTGATGGCATAACTTAGGAGACGACACACACCAACACAAAAAGCATTCACTCCATTCCTTCGCATATTAATGTTGCCTATCAAGAACCTTCCACGTACTCACTCAATCACAGCCCTCCATTCCTTTCTTTTCACTTTTACCTTTCTTTTTCCTGCTCCAACTTTCAATTTTCTGTTGGATCAAACTTCACATTTTTCCTTCTAATTTTCAATTTCATTTTTATAAATATTTGGTAACACCCAACTTTAAATAAAGTGTTAATAAAAAATGTACTAACATATAACACACTCATTTGGAAAGTAGATTTCACTTCATCACACTCATTTTGTGTACATGAGTGATGCACTTTCAAAATATGTAATAAAAAACGAAAACTAATTGTGGAGGGGAGAGTTTGGATCCAAATTTATTTGAAAATTATGTGAAACTAATTAGCAAGATAAAAAAGATGGAAGATTGTATTTGAAATTAGAAAATTGAAAACAAAAATGAGAAGAAATGTGAAGCGTTTGTTTAAATAAAATGTAAAAAATGATAATAAATTGAAATAAAGATTCGATATATATATACACACTTTTTGGGATATAATCATTATGAAGATGTACGACAATACTGACAAAATAAAACTAGACAAAATTCATTCAATAAATGGGCAGACTTTTAACACTTATTTTCAACTCTGCCCCTTTCTCTTTTTTAATCTCCGGAGCTTGGAGTTTCCTCGGGGAAGAAACACCCAATACTCTTTTGTTGCTTTCTGCTTAATGGAAGGCCCTTAACCTCTCTCTTTCTCCTCCTCCCTCCCCTTCCTCCAATTTTTCTTCCCTTCTGATCTACAGCTTCTTCTAGGTAATAATCTCTACACTCTTCCTTCCTTTTTTTTTTTTTTTTTCTTTTCTTTACACGCTATTCAACATCATGTTGCCTATCTTTCCAATCCATTTTTCTTCAATGTCTAAGCTGGAGCTGTGTTCTCCAGTTTTGTTATGATCCAACTCTTCTTCTCTCTCGCCCTTAACGTGACTGAGAATATTCAACACACGAAGAGATGGAAGATGATGACCCCTTACTCTCATCGCCATGTTTTAATGAAAACCTTTCAGCCAACTACTGGTGCATTAGCAGTTGCTGTTTTTTTTTTTTTTTTGTTCATTTCTGTGAAATAAGGGTGTCGGAAATCTGTGTATCTCTTTTTCTGAAAATAAGAGAAGTTTTATTGATTAGTTTCTTTTGATTTTCTCATCTCCAAACACATGGACGACGTCTTGTGTTTTTTATCGACTAGTTTCTGTTGATTTTCTTTTATCTTGCTCTTCATTCCGATCAAAAGAAAAGCTGCTGCCGGTGTATATATTCATTTTTTTTCGTTTAGCTCGTTTTTATTTTCTGCTTTTGCTTCTGAATCAAGCCATGCAGTTCGGGTATTTCATGGTGTTAATTGTTGGTCAAAGTCAAGCTGTTTTTACAATGTATTTTGGGCCATAACGTTTGTATCAAGTGATCCTCAAGGGTTGATTCTCGAATCGCAATACTCCTCTACAATTTTTTCAGAACTTCAGATTATTGGCTAGTTTGAAAGGGATCTTTAACTTTTAACTCGAAGAAAGGGAGATCAATCGTGTGATCCCTTGATTTTAATTCCAATACTGCTTGTATAACCACTTTTAAACCTAAAAATTTGGATCCTTTTTGCCTTGAAAGCTATCTACTTTCTTTTTTAATTCAGTTCCCATCTTTCTGTAATATTGATCTAGTTTTGCTTATTTCATTTCAGGGTTTAAAACATTTTTATTAGATTGTATAGATATTTCAATTTTGAAGTTCA

**>CsIAA9**

AAATTTATATGATTTTAATTTTATATTATTGGCTGTCAAAATGACAAATTGCCTGAAACCCATTTTGCAATTGTTTTCAAAAATAGTTTTTGTAAGAAAAAATTAAAATGTTTGGAACAAATTTAAAATGTGTTTTAATTAGTTAAAAATGCCTTTTGCCTTTTTCTTTCTTTCTTTCCTTTTCAAAAGAATATATCAAACTCAATGTTTTTCCCCTCTTCTAAAATATTGCTTAATTATTTTATTCAAATGAGAAAAGGAAAAAAAAACCCTTTTTCAAAAAAGGTTAAAAATAACCAAAAGGAAGAAATAAAAGGCATTGTGTCATAGCAATCAAAGCAAAGCCTCAAAGGATGAATACATCACTGAATATATATTTGATTTTTAAAATTTTAATTTCTTGAAATTTAAGCTTGTGGTATTATTATTAAACTTTTATTTAGAATTTAGTTATGCTTTTCAAAATCATTATATAATGAAACCTAAATGAGACGAGGCATGTCCTCTTAATACATTAATAAAGTTAGGGTTATTCTTAATTCTCACTTTTTCTTTGGGTATAGGTAGGTATGATATATCTTTATATGTGGAGATCATTATGGACATATTTTATAGAAATAATAAACAAGTCATTCACTTCTATATATTTGTTATTCAATTTTATTCTTTATATACATATAATTGAATTATATTTTTATACGCAAAATACATAAATATTTTTTAAAATAGCAAAATAAATTAGAATATTTACAAGTTATAGTAAAATTTTGGATTTTATCAATGATAGAAACGGATAGATGTGAGTTTCAATACCACAGATGGAAAGTTTATTAACGTATGTTTTTTATAATTGAGTTGCGTTAGAAACAAATTATTAAAATAAAATCTATTGTAAATAATGAAATTAAAATAATTAAACTGATTTTGAAATTCTTTTAGAAATGGAAGAAGAAATAATGTAGTTTTAATTAAACATTGAAATGTTTGTTTGAATTAGAAAAGTGGCCGTTTAAACGGCAAAAACTGAATCACTTCATAAAATCAAATTGAATTGAAACTCATTGAAACCAAAATATTCGAGTGGATCCGATTTAAAAGAATTTGAAACCGAAAACCACGAACTAATATATGTTTATATTTATATATTTAAAAAAGAGAGTAAGTTTGATTTTTAATTGATGCACTCTTAGTTTGAAAATAAAAACGATTTGAACTGAATAATAAATATCAAAACCAAATTTAAAACCGAATCAAATTGTTTAAATACAAAAAGTTGGATTTGAACCAAAAATCAATTTGATTTTGAGGCGAAATTTGAATCGAACCAGTTGAGTTTTGCACCTTAAATTTACAAATGTAAACTTTATATATAATTATGTGAACATGAAAGCCAAGTCGTCCAGATGCAGGTGGAGGTGCCATTTAAGATCACTAGGAAACAAACAATGGAGGGTTGAAAGGAAGATTTTGAGAGTGAAACCAAGAGGTGTTCTTTTTCTTTCTAAAGAAATAAAATTTACCCAAAAGGGGGAAGAAAAGATACGAGAAATTTAAAAAGAAGAAAATGGCAACAAAACATAAATGGAAAAATTTCAGCTTCTGCCGATAACTGGCTCGACCATGAGCCGTGTCCTGTTCTATGCTTCTACTTTTATCGAAACCCTCATCAGAAATGACTCAGAACAAACCATTTCTTCACAAAAATCACTCCTAATCGGCTTTTGCTAAACACCCTTTTTCTTTTATTATTATTTTTTACATGATGCTCCAATGGTTAGTAAATGATGTTTGTTTACAGTGTTTTAATGTCCATTTAACTCTGTATTTCACTTCATTTGTGTAGTTGGGGAAAAGGGAAAGTTGCTATTGCTACAGAAACGGCCAACAAAACTTCTTTTTGTGTATAAACAAAGCTTACTCACACACCATTCTCCTACTCTCTTTGGGTTGAATGAGCTTCTGTTTCTTGGAATTTTGAACAACTTGGTTCTCTCATTTTG

**>CsIAA10**

TTATCATTAGTATTTTATTTTATTATAATTATTTAATATTCAAATAAATTATTGTTTTTAAATATCATCCTTATAAGTAATTTACAATAGAAAAAACAGTAAAAAGTAGTAAATTCGAAAAACTATTTACAACATATTTCTATTAATAATGAACATTGACAGAGGCTGATATGTTTCTGTCGGTTGTATTGATAGACAGTAAAATGTTGCGTAAATATTTTAACTTATTTTACTATTTTAAAAATGCTAATTATATTAATTAAAAATAAAAAAATGGTAAAAATAGACCAAATATTTCTACTAAAAATAAAATATAATATATATTTGGTCTTTTAGCGTTTTTTTTTTTCATGAATCGGTTTATTTTTTAATTATTTGAAAAAATCGAAAAAAAGTATTGATTTGTTTTTATTTAATTAAGTAACATAACAATAAAATTAGTTAATGTTAAATTTAGTTGATCTATTGCATTTAATTAATTAATCATTTAGATTTGTGTAAAAGTTACACTTTGACAAACGGGGGGCTAAATACAGATTTGTTTAAAACCGAGGACTAAATTGAAAATTTTGTACAAAAGAAAAAGAATGATTTTATAGAGAGAGGATTTTGTAATATGCAATTTGTATAATTTGTATATTTGTGTGTGTGTGTGTTTGTGTGTTTTTAAAAGTGGTTTTAATGCATTGAGTTTCACATCGGACACCAAAAGGCTTTTGTTACTTTGTGCCAAATGGAGGGTCCTTAGCTCCTCTCTCTCTCTCTCTACCCTTCAGTTTTCTCTCCTGCTTTGCTACTGCTAAGAATCAAAACACTTCTTCAATCTCTACTACTTCTGGTTAACTCACTGATCTTCAGATTTATTCAGGTATAATCCTTTAATTTTGCTGTTTCTTCCTTCATTTCCACCTTTTTCTCACACCTTGATTCTGCTCTGATTCTGTTTCTTCCTCTTCTTTTTTTTAATAGCGAATTTCATCTTCCTTCTTCGATTCTTTGTTGTCTTCATGTTAGTTTTCTTGCTTCTTTGTTACTGTTTTTCTTTTCTTCTCTATTTCTGTCTTCCTCGAGCTTATACAGTTTATGAATGAATCTCGTAGTCTGTGAGTTGAGAAAATTGGTGAAAGATGAAGGTACTTTGGTTATGAATTCACGTATATGTAGTTTTCGGTTGTTTTGGCTAATGTGTGCTGCTTGTTAGACTGAATCTTATTCTTCTTACTTTTTTTTTTCGTTTGCTATTATCTGAAAGAATTTTGGAATTTAAGATCTGCGACGTCTGTCCTTTGTTTTACAACTTGCTGAATAGGTAGTCAAGAGCTACGGATTTCACTCTTAATTTGTTTATTTATTTTTTGTTTGTGGAACGAATTTTCTTTTTTCTTTTTGATCTTTGGATTGATTTCGGTTTCAGTTATCCCTGTGAATTTAATGCTTTTATACCAGCAGCTACACACATGTTCATTTTGTAGATTTATTTGTTTTGGCTTAGATACTTTGTTTTCTCTGCCTCTTATGCTTCCTGATTTTTGGGGGGAAAGGCTACGAGTAGAAGATTGTTCTTGTTCTTTATTTGATTTTCTTTTCTACGTGACTGTTTCAGCCGCTCCCCAATTCCTAACAGTTATGCAAATAATTTAGGCGGTTGTTATATGTGAAATTCCGATATGTTATTTTGGAAACGTTTCTGTTCCTCACTGAATTTCACGTCATATTTGAGAGAAGAAGTCTCTTTGGGTTAATGAAGATGAAAGTTTCCAATCATAATGTCCATTTTCTTCTTTATTTATTTGATTGTTTTTCCTCAACTTGTTGGATGGTTAGAAGAAAGTAATTTGAAATTGGGAGTGGTGTCTTATAGTCTAGGCAGCTGGACAAATAACAAAGTAGAGAGAACAGAAGAATCTTTGCAGTTTGGCTCTCTAGTTGAAATTGGGAGATTCATCGTGTTTTGTTTTATGCAGGTATAAAATCCGGGCCTTTTTCTTTCTGGAATTTTG

**>CsIAA11**

CATCTATAGCCTAAAAATAAGCAACTCAACCAGGCTAACTCTTAACCAATGTCAACTTTGAATTCAATAGAACTCAATCATGACTACATATATTTTTGAAGCCCTGACCATCCCTTCTTTATTTAATACTTGAGAGTTCTACCGACACGTCTAAGTTAAAGAACATGATCATCACTTTGATATCACTTGTTAAAGATAACAACTCTCTACGTATCTCTACAATAATATGAAACATGATCTATTTTTGACAAAAGTTTGTTTGCATAATTTTACTTTTAAAATCACTAGAAAAGCCTGAAACCAATTGAGAGATATTATTGCATGTCCCTCATTTATGTACTGTTGATTCTATTGGCCTTATAATAATCTATTCCATATAGAACTTTGGTCATATCTATATATGTATGTACAAATTAAGAATTAACGGTAGATTTTGTACACAAAGAAGAGTTTGATATTGTTATTGTTGGGGTATACCTATTTATTTTTTCCCAATGGTCCAATAAATGAGAAAAAAGGGGTAAAACATATGACATTAAAGTATTGAAAAGTGCTTCTATCTGTGCATGAGATTTTGAAAGAATAATTTGTTTGGATTTATCTATGGGTTATTATCAAGGGCCATACCGATTCTTAAATTTGAAAATTTTCTATAACCACACATTCTTCCTGTCAAACCCCATCAATGCAATTACCTGTCCTTCACATCAATTATTGTGTTATTCTTACTTATGTCATTAATTTTTGTGGGCTTAATATTCATTACATACCACCCAAAGTTTTCTCTATTTAAAATATTTATTCTACACATCAAGTTCATGCATTTAAGCTATTGAACTACATGAATTTATGACAAAAAAAAAAGGGTTAATTTTTGTTGTTTGTGTTTGAGGTCAAATTGGAGGGTTTTGATATAAATATGGAGTTTTTGTTATGATTATGACAAATTATATATATAGATGATGTGGTCCAGAAGTGGTAAATATATGTGGATATACAAATGTATATGTAGACAAAGAATTATAGTCAAATAATTATTTGATAAATTTTCTAAATACACCATCACATATTAGGATATGATAACATATGAATATAGAGTGAAAGAGATTTCATATAAATCTTTCCCAAATTAAGTAGAGTTAGTTTTATAACCACTTAACTTCACGTTGCCAATCTTAATTTTTTATTATCTATATTCTAAAATACTTTTAAAAGAAAAAGGTTTAGACCATACTTAATTACTTTAATGATTAGCCTTGACCCCAACTTATATGCAAGCATGTATATAACTCAATTTACATACATTTGTAATAATCACTTAAACGTCTAAACTTCAATCTTCCATTACTTAATTGTTTTTTCATCTAAAAAAATAATTTTAAAATAATCGATCAATATATACTCATATAACTAATAATAAGAGAATAACAACATTGTTGACACCATAATTACAATTAGTCAAAGATCATATTATGTGTGCTTAATTATCTTAAGAGCGATGATTGTGTTCTTTGAATCTCAATATCAAACAAGGTTGAACTACAAAAACAAAATAAAAATTTAAAAAAAGAGATTAAAACACTACTTTCAATATATATCTTAAAGTTAATTACTTTATATATCGATAGATTTTTCTTTTTCATTCGCATTTTCTTAACATGCATTATTATTATTATTTAAACATTGTAAATGACTAGTTATTATTGATAGTAAAGATTAAAAGAAAGTGTAAATTACATAGACCAAAATGACAAAGCTCAGAACAGAAGAAGAAGAAGAAAAAGAAAGAGAATATTATTATTATTTGCATGAGTAGGGACCGTAATTAATAGAGTGGAAATTAAAGTGAAAATGAAGTGATTTTGGTATCCCAAAAATAAGGTCCTTTAATTTCCCTCTCAGAACAATATTATTATTATTATCTGAAACAAAAGCTAGAAACCAAAAAAGGAGGTTAGCAAATTCTCAAAGTAGCTTTGGAGAGAAAGGGAATAAAAGAT

**>CsIAA12**

GACTCACACGTCTCCAATAGTAAATTTATAATTGGAGTGAAGATGATCGACTTACAATACGAAGAAGAAAAAACTTCGATGTGATGCTTACCACAAAACTTTTAATACTTAGGTCAGTATTTAACCAAAGGCTAAGAGTAGAGGGAAGGACTTTCCTTATTTTGGACTATCATGTTCTTAAATAGAGTTATGTCAGGGGCCTTCATGATCATCCTTTTTAAGGTTAGGACTCATTCTCCTTTATTCGTTTGGAAATATTTTGGAAATAGGATGCTTGAGATCACCTGAATTGGGCGACTAAAGATAGGGTCGTTCTAAATAAAGGACTATTGCATATAAGACCTCATCAGCCTCAACCTCAACCTTTGAAGTCGAGGTGAGTCAATTTGGGTCATCTAATCCAATTGTTTTTTTATATATTTTAAAGGAACTCACTTAGGACCGACAATCTCATGGTCAGCTTAGAATAAGGTAAATCTCTCGACATAGTTATACTTTATCTTAAAGTTTATTTATCGGTTGAAAATTAGTTTGGTCAAATTCAACCATATCTATAACACCCTTACTAAAACTTTATTAACTAATATAATTCAACTGGTTAAGTAATTATACAATCTGAACTAAAAAATTACGACACAAAGGTTTAAAGCCATGACTTTATAGATATTCATTATCTTTTGTCCCTACTATACAATCAACTTAATTGGGTAACCAAATTGGCATGAGTTGTAATAATATATAACCAGCAACTGAGATGAATAGATTAGTCCACCAATAAGACAGCAAATATCTCTTATCAAATGAAAGCTATAATAAGTTTCAATTTAGCCCCAAAATTTTAGAAGTCTTAGTTTAATCTATAACTTAACTTCACAATCTAATCCCTAAAGTTCTGAAAGTTTTTCAATTTTGTCAAATTTTGTTCCTATAATAAGTTTCAATTTAGTCCCTGTAATTTAGAAGTTTGTAATTTAAGCTATAATAACTTTGCTTCACAATCAACAAGTCCAGAAAGTTTTGGAAGCTTTTCATTTTTGTCTCCAAGATTGAGGTCTAATTAATTTTTATTTGGTTCTATAATTTAGTTAAATTTCACAAACAGTGTCTAACCCATATATCTTCCTTACCCACTTTTCCCCCAATTTTTTACTCAGTACAGGGCCTAAATTTTTAATAAATATAATTCACACTATCAATTAATGAAAAATGGCAAGTGTAAATATAATAGGTGAAGTTGGTAACTGTAAATATGATAGGTGAGGTTTGACTAAATTATCAGTATTGGTCGCCAAATTGAAAAGAAACTTTCCAAACATGAAGTCTAAATTATATATACAAAAGAATTTAATCGTTTTTTTTATTCAAATTTTATTTTAACTTCTACAGAATTCCTGTTTGATTTATAGACTCTGACACTTTCTTAGATTATATATAGATAAAAAAAAAAGGAAAACGGAATAACTCATGTATCTTGTGAAGATAACGAACACAGTTTTTCAACACCTATCGAAGATATAAAATCTTGATGTGATGGGAGTTTCTGTTCTTGACAGATCAAGAGAGGAGGAACTCATCCTCTCTGTCAACAGGGTCCATTATTCTATACCAAACACAAACACAAACAAGAGAGAGCAAGATACAAAATAAATTGTTCATAATACACACACATATATAATTTAAAGAAAACAGTAAACCGTGTGGACAATACCAACACAACAAGGCAAAATCACATGGGGTTTGCTAAAAAAAAGCAAGACATAATTAACCAAACGGCAGTGATCAAATCCCACCAAAATCTCAAACAACTATCAGCCCTCCGATCTGTTCCACATCTCTCTAACCATCTATACCCTACAACTTCTATTGTTTCCCCTCTTTCTCCTCTCCACAAATTCCCCATTCTCCTCCATATGTATAAATAAACAATATCCCTTCTTCAGTTTCATACAAAAACATCATCACACTTCATAATATTTACTACACCAAATCCAAATTCAAGG

**>CsIAA13**

AAGTACTTTAGCTTTGTTTTAAACGATTTTTATTGAATACTATTGATCCAATAATAAAACACTTAAAATAAATTAAAATTATAACATTTTGAATGGTAAGTTATAAAAGTAAAATGCAAAACTGTCTATATTAGAAAAGGTTAACTTACAAATATATAATAAAATATTAAACTATTTACAATCCGTATGACAAAAAAAGTCATTGAAGCCTATATTGTCTTGTTTTCACAAATTATGGAGTCACTGAAGTCAAATTCGTGCCAAAACTGGAACACCCACGTTGTCCACGATCGAAAACATACCACCGAAGTCAACCCATGCTGTCTACGACCGATACTCACTCCAACTGAAGTCTATCCATGTTGACTACACTCGCAGATGAAAAACCCAACGTTTTTCTTACTAGATTTGCAAAATCTCACTTTCTCTCTCCAGGTGTGTTATTCGGGTGCCTATCAATTTTTTCACTCTATCTTTTTTACGATTTTGAGTCTTTAGAAGATGAAAATAATTACAAAAATGCCACTGAGACAAAAATTACAATTTTGCCCACAACTACAGTGGACTCCAACTGTGCTTGAAAAACAATAATTGTGTATCTTAGATAAACATAGATAAATTGATACTTTTTTTCTATCTCGAATAGACACGGATGAATTTTTATTGGTGTCTATATGAGATTGACATAGATAGAATTCGAGATAGACAGAAATAGAATTTTACCTATGTCTATCCAAGATAGACATAGATATAATTTTACGCATGTTTATCTTGAATAAACACATGTAGCCGTCTATCTATGTCTATCTCAAATATATTTATCTATACTTCAAATAGGCAAGGTAAAATTGTGTGATCATCTTGTATTCTAGCTTCTTCCTTTTCTTTTTCTTGTTCTTCTTGTATTCAAACTTTTTCCTTTTCTTTTTCTTGTTCTTCTATTTCAACTTTTCTATCTTCTAGAACTTCAACTTGGGAAAGTAGAAATTTTAAAACTTGTATATATATATACTAGTTTAGGGTAAAAAAGTGATAGGGGACTATCATTTTTTATCCTGAATAGACAGTTATAGACTACTATCTCTTTGTCTATCCTAGATAGAAACATCCGTCTATCCTAAACAGAGATCATAGACCACTATTTATCACAAATAAATCGTGATAGATATATATATAGTTTGAAAGAAAAAAATCAATACATCGATCAGCCAACAATTGTATTGTTTTAGATGTTTCTAAGTTTGTGGTTTCAATATTTCCAAATCATCTTCCTCTATCTGCAATCATATAATGGAAAGTAAGGATTTTGAAAAACTTGATATATATTGTGATAATTTTGAGATAAAAAATTAAAAGATCACTACTACTTTCTATCCCATCCCAGATAGATAGAGATAATGATATATCTTTTATCTATCTTAGATAGAGATAGACAGAAATAGATCTATAGTTTGGTTAATGATCCATTTTTTTTTTCATTTAAAGAAGAATGCCAATTTTGTAATTATAACTTTGCTAAAAATATTTTCTACCATGTAAATATTTTAGGAAAAAAAAAAACATATTCTCATGGAAAGATGAAGGATTTGAGTAATTAAAATTGGAGTTAAAATGAAAATAAAAGGAAAAAAGCAAATTTAAAAAACGAAAAAAGCATTTAGATTGAATGTACCGACAAAGACAAAACCCTCCTCAATTTCAATTCCATGGGTTCTTCTCTCTGACGAAGCTTTGGTACTAACCTTTATTTAATATATATGAATTCAACAACATCCATACCCCACGTGTCCTCTCACTATTTCTCCTCTTTTTACATTACAATAATCATCTCACCTCCATGCTTCCTCTTTCTTATTACTTTATCATCTACTTCTAACCTTCTATATATATATGTATGTGTATATATATATATAATATGAAATGAAAACCACACACCAAACAATTTTAATTCATTTCAAATCAAACCATTTTCTCTCAGGCAGAGCAACATATTATATGTT

**>CsIAA14**

ACTTTTATAAAGCGGATAAATCTAGACACTTCATTAGATAGTCACTCCATAAATGAAAAGATTAATCTGATTAATAGTTAACAAAAACAAATTCATACACCAGTTGTGCTATGTTATAGTAACAAATGGTATTGTTACTACTATTACTGAAACTTATTTTTATAAGGTAAGTACCACAAAAAGAACCACCTCTAAACATATTTAAAAGTGGAAGAATACCTAATTTAAAAGAATGAACAAGAAAACAATAACCCACAAGCTCCTCTCTTGGTATTCTCAAGTGTTGAAGGTCCACATTCCCACTAAGCAAACATATCTTTAAGCTTCACTTTCTACAACCAAGGTGTCTATGCCAATTGTCTTGCAAGTGGAAGACAACCACAAAAGAAATAAAGAGAATGGTATTAAGAATAGTATAATTTTAATGTCTTTGGTCTACAAAAGCCACAACCTTGAGAATATTAAAAACCCTAAACCCCCATTTTTTCTCCTTCCCACTACTAAAGATAGAGACACCCCATTTCACCCTCAAATCTTTCTTTAATTTTACTATTATTTCTTTTCACTAACTTTGCCTTTAACCATTTGTCTTTTAGGGTATTATATATTAAGATATAGGGTACTATATCTACCCTAAATCCATCTATGTCTCACTAATCCTCTTGTACTAGATTGAGTTATATATATATTAAACTCGTAATTACTTTTGATAATAATATTCTCCATGTCATCGAAATTAGAGTATCATCGAATGTGGATGCAATAATATCTTACTTGAAAATATTATATGTTAGGAAACTAAGGACAATGTAATTGAAAAAGTTTCAATATTCCCTGTGTTGAAAAGTTTAGTTTCTCTAGTCCACATATGTTGGTCTCACCATATGGAATTTTGTTTTGATTCCTTTGATAAAATTTTAGGTTTATCCCTTTTAGTTTTTTTTTTTTTCATTATTTGTTCAAACTTGTTCGAGTGAACTTATATCTCGATCTCTAAACTTTTATATGATTATGTTTGCCATTGGAATGTTTAAGATTCACTCATTTATCAATAGTTATAATTTTAACCAATAGTACACGTAATATATTTTTAATGCAATACATTAGATCCCACGTATTGGAACTCTTTTATCTAATGTTTAAACAACAAATTACTAACAAAAGTACACTGTAAACATAATTCAACTGATAAAAATACATACTTAAAGTAAGATCGAAAGAAATGCGAAAAGCAAATAGATAAATGAACATGTAAGAGAGAATAACAAACACATGTGACTATAATGTTTAACGGATCAAAATTAAAAATATAAAATTAAACTGTGTGTCAACTATTTCAATATAGAGAGTTCCCTCGTCGATATCTTTATGAAAAAAAACATCAATCATTCTTAATTATAAAATTTTGAAACTTTTTTTTTTTTGATAAGAATGTTATTACTACTTTATTAATTTTGATTAAAATTAAGTTTGATAATAAAATTTGAGATATTTAATATGACTAATATTGTACTTGAGAACAAGAGTTTGTGGAATTCAAATTTTTAACTAAAATAGTATTTTTAATCAAATAATGAACGTAGTGGACAAAAAGGTAATTTCGTTTGTTCAAATTATGTCCTCGAGTTTGAAATAAAAAATAATATTATCTTCTGTTTTTTTAAAAAAAGGATTGAATGTGTAGAATTCCAATGGCAGTACGGTCTACAGACTACAGAGAGGAGACAGAGAGAAAGAGAAAGAGAGAGAGAGAGAGAGATCCGTGAAGGAGACATGAATCGTGTTGAGATTCAGCTCAAAAGGCAAATTCTTGTCGACAACAATGGAGAACCATGGTGGGTAACTGCTATCACTGGTAGCCGCACTACCAGACATCTGGGTCCCATCATCATCACACCCACCTCCTCTTCATCTTCCTCATACTCATACTCATACTCATTCCATTTTCTTCCTTTCAATCCAACCATTTTTCTGTTTTACTCCATCTCCATTCCCCCTCC

**>CsIAA15**

TGCATTTGGGGGAGGGAATGAGTTACGAAGAAAAAAGATTATGATAATCTAGAATAATCCTAGTGTTATGATAATATATGTTTGAGGTTATGGTTATTATATTGGTAGTGTTTGGGAGAAGGAATACAAATGGTAGTGTTATGATAATATGTGCTTGGAAAAAAATATTATTATTGTAGTATTACGATAATATGTGTTTGAGGGAAGAGTTATAATTGTGGTGTTATTATAACATGTGTTTTGGAAAAGAGTTACAAGTGTAGTATTATGATAAAGATGAAGTAGGAAAATTTGTAAGGTTATGAAAAGGTGAAGTAGAAAAATTTAGAGGGTTATGAAAAGATGAAATAGGTAGTTAGTAGGGTTATTTAACCCTAATTCTAAAAAATGGAAACCCTTTGCTCAAACGAGGTATGAGCCATAATCCTTTATAATGAGAAAACCGCGAGAATAGCAAAATAATAGATAACAGTTGGGGAAATAGCAACTTTTGCAAATAATTATATTTTTTCAAAATATAATTATTTTCTTATTCTTTCTAAACAAATATTCTCACCAAATAAGTATCCTCCCACAAATAATTTATTTTTCCTTCTCTACAAAATACAATATATTTTTCTAATAATTTCAAGATAACATGCTACTATTTTTAAAATATATTCTTTAAAAAAAAAATTAAAATGCCTATTTCTTTATCAAATTAGTTCATGATGTTTAATAGTCTTTTACGGTAATTTGACATGTGTATAAACATTGTTAATATAAGACAAACCAAACACAGTTTTGCATATAAATATTTATAAAGAGATTAAAGCAAACATTTATAAACATGAAAAGTCGAAGACGAAATGGATCTATTTGTATAAAATTAAGAGTTAAAAAGATTTATTTATAAAAGTCGGACAAGAAAAACAGATATTGATAAAATTTTAAATTTTGTTGAGTAAAATTGTAGTTTTCCTTTATATAAATTATACAAATTAAGAATTTTCAAAATTAGGTTGAAATTGAGAATAAAATTTGGAGAGTTTGAGAAATGGAATTAGTTAATGTAATGAAAAGTTTAGAAAGTATAATTGAAATAGAAAAAAGAGTTATAATTTGAAAAATGGTAGTTGATAGGAGTGAAAGGTGAAAGGCAATAATGGGATGAAGTGGAGGGTCCAAGAAGTAAAGTATGTGAAGGTGAGGGGGCAAACAAAAAATAACCATGTCCAGTGGGGTCCAGGGGTGGATTTCGTCCTTTGAGGAATCGTGGGTCCCCATGCGTCTCAACTTCTGACGTGTCATGCAATGTGATGGGATGCCGGCCCCACCTTGTCGGCCTCTCTTTTGGAAACTCATCACCCCGCCTTCCACCCTTTTTCTCTTTCTCGCCAATATTTCTTTCATTTTACCATTTCCATAGACCTCTCTTTGTACACCTTTGAATAATTACAACAAAGAAGAACATGGTTAAATCATAATATTTGTATGGTACTGATGCTAGAAGGATACAACACCTAATAGTGTTGAGTAGAAGCACCACCTAATATTGAATAATTGTGAGAAGAAAATGAAAAAGGTAAAAGATTCTTCCATAACAACGTGAGCATCTTTGAAATAAATATAAGAGAAATTGAAAGTGTCGAGAAAAGAGAAAAGAGAAGAAACAATTTAGAAATGAAGGGGAAAAGAAAAGAAGAGAAAAGAAGCATTAGAAGAGAAGGGAAAACAGTAGAGTAGAGAAAAAGACAATGATGGAGATGGAGGCGCATAACCCAGACAGGTAGGGTCCAATTCCTCCATAAAATTCAATTCCCATTCCATTTCTATCTCCATCTTCCTCCCTACTCCACCTCCATTAAAACCCACCATTGCTTTTCCTTTCCTCCTCCTCCTCCTCCTCCTCCTTCTTCTTCTTCTTCCAATTACTTCTTTTCTAATCAAACCCAACCACCCTCTTTCCTTAAATCCCCCCTTCTCTATCTTCCTTCTATTTCATGCTTTCTCAACATTCC

**>CsIAA16**

TGATAGGGTTGCATGATCCTTTTTTAGAGGCATTATGCATGAGGTAGGGGTATGACAAAAAGTGGGCGAGCTTAATCATCGACCGTGTATCATGATTCATCGGTGACAACTAATTTTGTAATGAACAACCGAGGAAAATGTTGCAACTAAAGTCTGGTTTGAGCTAAAGGGATTATTTTTCTCCATATCTATTTTTGTTTTGCGTTGAAGGCTCCTGTCAAGGGGAGGTTTCTCCGGTGCAATCAAGAAAGCAATGTCCAATAACTTATATATATACTCTCATACGTACTTGCAGGTGGTAGTCTATTGTTTTGTAAAACTACCGTGCAAGAGGGGCATCTTAATGATTATGTAAGGATATTTGAAGGTGTGTGTGGCAAAATTCTGGTGGAAGATCATCTCATACTAACTCTAAAATTTAACGGGTATTATTAAGATAATTTTTAGGTTGGTTGGGTTTAAGGAGTTTGAAAACTTTTTAGTCAAGCATAGTGGCTAAATAGGTGTGGTGCCTATTAATAGACCCTAGTTCTTTATCTGAGATATGATGTTTAAAAAAAATACTATATCTTGATCGTTCTTTAGCTGTGACAATCTTGGGTAGAACAATCCATCTAAGCTTCATATATATGGGAGAAATCACTTTTGTTAGGTATTTGCATGTATATGTCTCTCCATAGATTCCTAAGATTTATCTCTTCAGACCTATTATGTCCCTTAAACAAGGAAACAAGGTTAGAGTTCGCTGAGATTTTTACATGAAATTACGTTTTTACACCCTAGTTTACCATTTTTAACTTTATTTAAAATTTTTAAAAATATTTAAGTATTTGGCAAAATATTTACAGTATATAGCAAAAATTTTAGATTATATCAATAGTAGATATTGATATTTTTCCTCCTAGTAGTCGTAATAGAATCCAAACTTTTGATATATTTTGTAAATATTTTAATTTATTTTATTAATTTTAAAAATATCCCTTTTGCATGTGATTTTAAATTAATATTTTAATACATTTGTAAGAGATTTCATTTTTATTTTTCATATTCCTCCCTCAAACCAACCACTTTTCCATTGAGATCCAGAGCATATGTTGAAGATATACTAACAATTATATAATGACAACAAAATATAGATCATACCATGATTTAATTTTTTGGCATGTTTACAAATATATATTGGAAATGTAAATATTAGTATATATATATAACAATATATAAATAAATTGTCTGGTTCATGCACTAACAAATATATTTATTTTTGGAAAATTCAAATATATGTTTGATAATATGTTTTATATATCAAATATATGTCCTTCTTTTTGCTTTGAAACTTCTTCCTTATTCCTGCTTTCGTTTATCAACATTTCTTTACAAACTTTTCTTACTTGTGTAAGTTTTAAAGAGAGTTTTTTTAAAAGTTCACAACCGTGAGAAATTCTTTTGATATATATGTATGCTGTATGCATATATATATATGAAAAAGTAAATTTGGTAGAAGTCTGCATAAATTAATAGTTCATTAAAATAATGTTAATGTAAAAAATAGGAAAATTTAGGCAATATTTATATAACTATATATTGAAATAAAATATAATTATATCAGATAATTTATTTATGTTTGAGAAGAATAGATATCAGCATTATTAATTTACACATTACAAAATGAAAGTAGAAGAAAAAAAACTGAAATTAAAATGATTATAATATAAACAAAAAGTTTAATTATTTTTTGCATAAATCAATTAATATGTTTGTTTATTAGATTAGAGGAATATGATTGTTGTCGGGAAAAAAGAGGAAGCATGGATTACTGGTATGAGTGGTATGCGCACCACCAGACATCCTGACCCCATTTCTCTTCTCTCTCTCTCTCTCTGCTTCAATCCATGGCGTAAACACTTCCCTTACTTCTTCAATACCCTCTTAATCCCCTTCTCTTCCACTCCCATTTCCCATTCTCTAAACCTTCCCTTCTTTTCTAATCCCCATTTTCCTCC

**>CsIAA17**

TGACTAGGCTATTTTGTTAAGTAACATATTACATGTTGATATATAAATCATAGTGGTTAGTTCAACCAAACAAAATTTAACATTTAATTTAAAGAGTGTATTATCTCCTTTTACACACTTTTATTCATGTTAATCAATCTACTTTTAAATTACTTTTTCTTTAAAAAGAATTGGCTCTTTATTGTCGTAAAATGAAAACAAATTAGTCAATCAAAATCATTATTTTGTAAGTATAAAAACTAAAGTTAATGAGAAATAATGAATAAAAACTCTGAAAAGAAAAATAAAGATGGTAAATAGTTTGTTTTTGGGTTAAAAGTAAAGAGTTTTTGTATAATTATATCACATAATTAAATAGTAGAGCAGCAACACTAACTTAAATTCGTGGAAAACATGACATTATTAAAGTTAGTGAATCTACATCAATTCTTATTATGGTATTTGATGTTTTATTCAATCGAAATTCAATATAAGCCAATAATTGAAATGATGTCATAATATTAATTCTTTTTTTTTTATATGATTAAACAATTAAGTAATATAACAGTATATTAAACAACACTAAATTAAATTTAATGGCTTCATTATGTTTTTGTTAAATATAATTAAATTAGCAACAAATCTGACTTCATCCTCTACTTTTTGCAAATATGTTAGAAAACTGAACCCAATTTAAAAAAATAAAAGAATAATTTTTAAAAACATGTTTTTCATTTTGAAATTATGATAAGAACTTGTCTTTTTAAAAGATGCAAAATGTGTAAGTAAATGAGAGGAAATATATTTAATTTTAAAAATATAAAACAAAAAAAACTGATAAAAAGTTTAGTTGTTTAAAACAAAGGGTAGTAAAAAAATAGAAAGAAAACTAGAAATAAAAGAGGTACTATAAACTTAATTATTTAACACAACACACACACACAAATATATAAATATATATATATTAAAGTAAAGACAATATTTAAGTTTGATGCATATATTTAAAATAATAAAAAAACAAAACATTAATTTATGTGCATGAATAAAATGTGAAAGATTGAATAGCCAAACTCATAATTGAATATCGGAAGGCAAAACAACGTAAGAGTTCCGAAAGAAAATAAAAAAAATGTAAATCTGTATATTTAAAGAGATGTTAAGAAGAAGGAAAGAATGTCAAAAGAAAAAGAATAAAAATGAATGAGAGTGAAAATAAAAAATGAAGTTTCTTCCCCACAAATGCAGGCATGTTGGATGTTTGGGTTGGTGTGCAAGTTAGGCTAAATCTGTTCTTGCTTACCACTGCCATTTTCACTCAGTCCTCAAAAAAGTTCAACAAACAAAAAAAAAAAGAGAGAAAGAAACTTTTTGAGAGAGAGTATCTTTTTTTAGCACGCTTTGCTTCCTCGTCTGATTAATCCCTCCCTCCCTCCATTTCTTCTTTTCCTTTGTTGCGTGAATAATCCTCTAAATACCCTTCTTCTTCTTCAGAATCTCTTCTTTCTATTTCTCATTTTCTCCTTCTTCTTCTTCTTCTTCTTCTTCTTCTTCTTCTTCTTCTTCTTCTTCTTCTTCTTCTTCTTCTTCTTCTTCTTCTTCTTCTTCTTCTTCTTCTTCTTCTTCTTCTTCTTCTTCTTCTTCTTCTTCTTCTTCTTCTTCTTCTTCTTCTTCTTCTTCTTCTTTTACTTTCTTTTTAACCTTTTAATACCAATTTTTCTCCTCAACCCTTCCAATCCCATCTCCCTTTCTTTCTCTTCTCTCTGGTTTATGCTTTACTAACCCTTTTCTTTACTTCTTTCTTCAATCCCCCTTTTTGTTTTTTTCCCTTTTTGATCGTTTTTGTGTCACTTTGCGATTTGGGTTTTGTTTCTTTTGATCTCTTCCTCTAAATTTTCGCATTACTACCTCTGTTTCCTGTTTTAAATCTGTTCTCTCCCTTTTCAACTCAGTTTCTTTCCTAATTTCTCCATACTTATCATTTTTAATCGTTTGTCTCATTTGGGTTATCTCAAAATCTTTCA

**>CsIAA18**

CAACTCTTACTTTATTCTTTATCTCACTTAATCAATTGTTTATTTTCTATATAGATATTCCAATACATTGACCCATTCATCGTTTTTATTATTTAAAATATAAATTTGTACGTCAACTCGATTTAATGTCCATTATTAATATTATTATTGAGATAAAAATGTTGGTATGATATGTTATTAAATAGCTGTCCATTTTAGGCACAATTTTAGAGTTTCCTTTTAAGAAAAATGATAAATAAGTAGTACAAATAGTAGTTTATATGAATGTGAGTAAATTTGAGACAAACATGGATATGCTTTTAATTAATTTCTTTTAGGAGTATAGTTGTGTGTTTTGTGTTAAGCATGCTTAATCATGAAGTTTGAAGTGAATAGGGACCACTCAAAAAACAATTTTAAAATAAATAAAATACTATTTAATCTATTTCTTATAATTAAATGCTGAGATTCCTTTTAATCTCTTTTTCCTTTCTTTAAACACTGCCTTTTTATTTCTCTCAAAACCTCTCTTCTTTTTACTTCATTTATTAATGCTACTTACAATTTCACTTTAATTTTCTCATTTAAGACATTAGAATCAACTCTGGAATTGGAACACCCAAATTACATAATATTTCCATGTTTTATAATTTATCCATCTAATAGACGCAGTTACCACACCAAATCTATCATCCTAAATTTACTTCCAATGGATACAATTAATTTCAACAAAATATACACAAAATTCAATCTATAAAACATAAAATTAAATTTAAGAACTTAGGTGGACGATTGTTCCATTTTTCTTTTATGAAAAATTTAAATTTAGAAGAATAAGTGTGTAAATTTAATAAATGTATCTTTTTAATCTCTTACTTTTTTAAAATAGTTTTAAACCGACCTAATGAAAATTATTTAATTTAAAATCGCATATAATTTTTTAAAAGTAAAAAATTATGTTTTAAGAATTAAAAGTATAAATTTAGCAAAATGAGCTTACCCAACTTTGCACATATGTTGTAATGAAAATAGAAGAGATGAAAGAAGGAATAGTTAGGGTTTTTGACGATGAAAGGGAAAGAAAATGGAAGATATATTCAATTCAGATATCAAAAAGTAGTTGGGAAGAAGAAATTAATTTTAATTGTCCCCTAAAACTCTGTGATTTTGGTCTCAGAATGAAAATTCAGAACCACATCTCTGCGATGATTTATATGTTCATGTCAGCTTTCTCAAAATTCCCTCACTTTCCACGACTTCTCGTTTTCTTTCGCCAAACCCCCCATTTTCACTTTTTTTTTTTTTCTAACCCACAAAGAAGAAAACCCTAATCCCTTCTTCTTCTTCTTCATTTTTCTTATTTCAATTGAATTTTCCTTCAAAATCTGACCACTTCTTACAATTTCAGACTCTGCTTTCGTCCTATCCTTTTCCGCATTCCCGTGTTACGCTGCTTTCAATTTTTTACACTGTTTAATGCTGTCGCTTGGCGCTCTCTGAAAGAGAAGATAAAAGAAAAGGGGAAATCAAAAAACCCCATAGACCAAAGCTACAGAGAAAGCATTTTTCTTTTTTGATTAAAGAAAAAGGGCAATTGAGGGAAAAGGTAGACGGAGACTTTCTGTGACTTGTCGGGGTGGTTGCCCACAAAAAGGTACATTCAATTTTAAAATAACTATAACAAATCCTGAAAAGAAGCAGAGATACAGAATTTTTTTCCTTTTTTCCTTTTTTATTTATTAATATTCAAACAATTCCATGTTTTCTCTTTTATGATGCCTTTCCTCTTCTCCGTTTCTTTCATTTCTCTTCTTCAAAAGGGTCTTTCACTTACTCACAGTAGTTGAGATTTCAGATTTTCCTTTTCTATTTTGTTCAACCAAAGTGAGGAACCAACAGGGCTTTGTCAATCTTAAAGAAGCTTCTCTTCCTATTACCATTGATTCTCTTTCTCTTCTTCTTTTATTGTGATTTAATCCCATGCTTTTTGTGTATTCATCTTGAGTTTCTAATTTTTTCTG

**>CsIAA19**

CAGTTAATTGATCTAAATTGTTCTCATTCTATGTATATGTTTGACACCATGATTGCATGGTATTGTTCATCACAAGTTGGAGTTAATAGTACTGTTGCCATTTAATCTTTTATTTCGTTTCCTTTTTTTCCTCCTTTTTTCAAGTTCTAAATTTGGCTAACATCCAAATTCTCTATCGATCAACATCCATAACTAATATACTTTATCATTCTCTAACTACTTCTTTAGAAGTGTTTTGAGATAAGAGTTGTTGTTTGAGATAATGATCCTATTAAACCAAGTTTTTAATGCTGATTTTATTTATTTTTGTCTTCTAGTCCTATGGGTGTTTCCTTTCTATGCTTATTGTTTCCTTGGCTTAGAAAAGGAAGGACAAAACAATAGAAAACTATTTATGAAGAAACATTCTATTTCTTAAATGTGTCAAGTTAACATTTAAGAAGATATTACATTATTCCAATATAGAAAAGCAGTCAAATAAGTATTTCCTATTAACTAAATTAAAAATGGTAAGTAATAGCGGTAAAAAAAACTAGTTTGAATATAGTTTAACTAGTTAAGAGTATATGGTATAGAGATAATTTTGAAACAATAAAAAAGTAAAAATGAGATAATTAAATTATGAAGGTAGAAAACATGCCTAGAAGAGGGATCTATGGAGGGAAGAACGAGTGGGGCAATTATCCCACCTAGCTAAATTGTCTGTTTTTGTACTTTGAAAGACTTCTTAAACTTAATTGTGTATATATGTTTGGTCCAATATTTTCAAACGAAAGAAATCACAAGTTTAATTTAGCAACTGTAGGTCATGAGAATTATACAAAACTAGGTTGACTTTATGATGAAAGTTTACCTAAATACATTATTTGGATTTGGATTTGGATTGATTCAAAAGTGATCTCTTATTTCAAATTAAGAAAAGATGTACGGTGGAAAAGAAGTAAATGTGAGATGAGTTTGGTTGGCCAAAATTTGGGCAAAACATAATTGGTGTTAAATATTGGATCCCACTTGCGAGGAAGGAAAAGACGTGATGTAATTAAAAAGCAGTAAGGAGGAGGGTAATGTTTTGAATGGGCCAAGGCCCAAGGGTGATGAAGGTAGGCCCAAATGTCAATAATAGAGCGGCAAGGGAAGGCTACAGAAGAGAAGAGAAGACAGTGATTTGATCTGTTATTGGTTCGGGAAAGCAAAGCCTGTGTGTGTGTGTGTGGGCTCCCTTACTCCCTTAAGTTTGAAAGTCTAAACCTACCAACCAACCAAAAAAAGCCAATTACCCCCCAAACCCTTCTATTTCTCCCTTTTATGATTTCCCATTGGTGCTCACGTGGACTTCACAAGCATGGGTCTCTTTTCATTTTCATTTTCAATAATTAAATTATCCCCTTTCTTTCTGCCACGTGACATACCCCTTCCCCTTGTCCATTCCTTTTAGTTTTTTAACCCTACCTCCCATATGCACAATTCCACATTCAACTTCCCAACACCACAAATCAAACACTTACTCATCCTATTCCTCACTTGTTTGTTTAGAATAAATGGAAAAACTAATAAACTAGAAATTTAATACTTCCTATGAATATAAACAAACTTTGAGCTTATTATCTTATAGCATTTTAGATTAATATTTCCACAGAACTGTTCCCCTTTTAAGGTAGAGTAGTAAGAAAGTAGGAATGAATTTAAGACAACCCATTGTCTGCAATTTACTTGTTTTTGTTGAATACTACAAGGTAGAGGACAGGAAAAGGGATTATTAATACCAAAAGAGTGGTATGAGATAAAAGAGAGACAAAGGAGGTCCAACCCACACGCCCCCTCTATCTCTATCCTCATCCACTCTCATTTTCCCATCCCTTTTCCCACACTCAAACTATTCTCTTTCTCTTTTCTATAAATGCCCATATTTCTTTTCCTATATATATACCCACTCTCTCCATTCTCCCTCTTTATTCCCTTCAAAACCTTCTCTCTTTTCTCTTTTAAAATCCCTATCCAAA

**>CsIAA20**

TTACGCTGCTTTCAATTTTTTACACTGTTTAATGCTGTCGCTTGGCGCTCTCTGAAAGAGAAGATAAAAGAAAAGGGGAAATCAAAAAACCCCATAGACCAAAGCTACAGAGAAAGCATTTTTCTTTTTTGATTAAAGAAAAAGGGCAATTGAGGGAAAAGGTAGACGGAGACTTTCTGTGACTTGTCGGGGTGGTTGCCCACAAAAAGGTACATTCAATTTTAAAATAACTATAACAAATCCTGAAAAGAAGCAGAGATACAGAATTTTTTTCCTTTTTTCCTTTTTTATTTATTAATATTCAAACAATTCCATGTTTTCTCTTTTATGATGCCTTTCCTCTTCTCCGTTTCTTTCATTTCTCTTCTTCAAAAGGGTCTTTCACTTACTCACAGTAGTTGAGATTTCAGATTTTCCTTTTCTATTTTGTTCAACCAAAGTGAGGAACCAACAGGGCTTTGTCAATCTTAAAGAAGCTTCTCTTCCTATTACCATTGATTCTCTTTCTCTTCTTCTTTTATTGTGATTTAATCCCATGCTTTTTGTGTATTCATCTTGAGTTTCTAATTTTTTCTGATGTCTATACCGTTAGAACATGATTACATAGGCTTAACAGAGTCTGTTCCTTCTCTGGAAAACTCTGAAAAGTCTTCTGATAAACGCAACAGTGCTGGTTTGAACCTTAAGGCTACTGAACTGAGGTTGGGTCTGCCGGGATCGGAGTCACCGGGAAGGGATGATGGGTTTGAGGATAAGAATGGGTTCCTTCACAAGAGCTCTGTTTCTGGGGCTAAGAGGGGTTTCTCCATAGCCATTGATAGAGCTTCTGCCAAGTGGGTTCTACCAGCTTCTGCCGGATCTGAGGCAGACTCCTCCACAAATGGGGGTTTGTTTTCTCCTAGAGGTGTTAATGAGAATAAGACTCAACCACCTGCTTCCGCTGTCTCCGGTGTCAAAGACGGTATTTCTCCGTCAGCCAAGCCACTTCATGAGGAAAAGCCTCAGCTTTCTCCCCCTGCAGCTAAGTATGTGTTTTTCCTTTGATTTTTCCATCTGAATAAATTGTGCTTTGGAACTCTGCTTGTTCAACATCAGCTTTTTCCTTTCCTTCTTCTTGACTTTGTTTTTCTTATCTTCTTCTCCTATTTGAACGAAAAATTTGGGCTGTGATTCATTCATGTAGTTTTAATCGTTCGTAGAAAAACCTTGACCCTTTTGATAGACAACGTTATTTTCTGTCACTATAAGCTGGAAGATCCATAATTTTGGTCTTTACTTTTTCCCTTTGTATTACAAACTATGTTGGCTGCATATAATTCTTTCGGTTCTGGTCGGTCCGAGCTTGTTAACCTTCTCTGCGAGGTCTCGTCTCTCTGACCCTTCTTCCATCACCTGTAAGATTGTGTAGGAGTGAATTTGAACAGATATCTGTCAAGCTTACTAGCTTGGCTGTTTTCTTAGATTTGGAATGTTTTTACAGGCAAATCTTTTTTTAAATTTTCCACCTTGATATCAGTTATTTGTGTGGATTTCATGTTTCTATCGAAGTTCCTAATTCCACAAAAATCTCAAAGTGCTTTATCATTTTTGTTACAGGGCACAAGTTGTAGGATGGCCACCCATTCGTTCTTTCCGTAAGAATTCAATGGCAACACAGCCCCCTAAAAATACAGATGATGCGGATGGTAAGTTGGGATCAGGCTGCCTTTATGTCAAGGTAAGTATGGATGGCGCACCGTACCTCAGGAAGGTTGATCTGAAAACCTATGTAAGCTATGTGGACCTCTCATCAGCCCTGGAGAAAATGTTCAGCAGCTTCACAATTGGTAGTCTTGCAGATCTTGGCTGATTTAGTGTCTTGTGTAACTCCTGCCCGTGTCCTGTATTGTAATCATATTGCATCACTTCCATGGTTCAGCCACAAACACCTTCAGGAATGTAAATGTTTAGGTTTGGCTCGTAAGAAACTCATGTGGCAAAAGTATCTAAAGACAAAATAA

**>CsIAA21**

ACTACGTGAAATTCACATCCGTATTAGAAAATTTGAAAAGAAATTTAATGTAATAATTGTGATGACTTCATGACTAAGAAAAAACAAGGTTAAATTTTCATAATAATAATTTATTTGCTGGATTGGAACAATTTGGTCCTTGTTTTATATTATTAGAGGTATGAGAAGAAATAAAGTAATATTTTAAACAATGCATGTACCTCATTTAAGTGATATATTTGTAATTACGTAACTCACAAGATTTGTCGTAAAATTCCCCCCTACTCACAAGAATCATTGTAATATTACGAGAAAAGCACTTTGGAAGTTCTGGTAAATATATAATATCCTCCCAAAGGATGATCTCATGGTTAAAAAATAGTTTTATTTCAATTTGAATTTTGAAAAGAAAATTAAGTTAATTTATATCAACATCGAAAATGCACAACAAGAAATAAAAACATGTAATTCGACTTTTAGACAGTATTAGAAAACTAGAGTAGAAAAGAAAAAGAAAAAAAAAAGGGAAAAGAATAAACTATTTAAATCAATTGACAAGTCATATACTTTATTGCAATGAATATATAGAGAAATTTTTTTAAAAAATAATAAAAACTTTATAGTAGGGACCAATAATTAATGGATTGATATAAAAATACAAACAATTTAAATATATATAAATAAAATAAAAAAGAATGGAGGTAAAATGATTTTACATGCAATCCAATAAAAGAGAAGTGAATTTTGGTTGCTTATATTATTGGGTTGCAGTCTCTATTAAAAACCAAAGGCCATATAATATATAATACACACCAAAGAACTAGAAGGCGACACTACGAGAAGAAGAAAAAGACGACGACAGTTTAATAAAAAGCTTTCTTTTGATTCTATATGGAAGCAGTTGGCCTGAATATGCCCAACAATCAGTTCGTTAGCTTGAAGGAGACTGAGCTCTGCCTCGGTTTGCCCGGTGGCGGTGGCAGTGGTGGAGATCAGACCTCTCTCAAAGCTTCTGGCAAGAGGGGGTTCTCTGAAACTGTTGATCTCAAACTTAACCTTCAGAGCAAGGACGGTGGAGGAGGTGTCGGTGTTGGTGTCGGGGTGGATCTCAATGAGAATATTAAGAATGTTTCGACGAACGTGGATGGTGAGAAGAGCCTCTGCTCCAAGGATCCTGCCAAGCCACCCGCCAAGTAAATTTCTTCCTCCAAACTGAACTAAATTCAATTTAGGGTTTATTTAGTTGTAACTCATGGCTTTATATCGTGGGATATATCTCAGAACTAATTAACATGTCCCTTAATATGGTCTCTTTTGAGTTCACCAATCATCATAGAATTTTCATTTCTTTTATTGGATTGAATATTTGTTTGTGTTTAATGAGTCTCCTTACTTCCCAGCATGTCAATGTATGAGAGAGAGAAATATCGTAAGAATGATTTTATTTGTTAACTTGGTTAGAAATTTGTATCCTTCTCCCACCCCACCTTCAACTGGACTTGAAGGAACTATAGAAATTAATTAAGCAAATTTAAACATGCATCTATCTGATAGTTTGTTGATTTCTTAACAATAGTTTGTTGGGTTTTCTTTAGAGCACAAGTTGTGGGATGGCCACCAGTTCGATCGTACAGGAAGAACGTGATGGCACAGAAGAACACAAGTGGCGGCGAAGGAACGGAGAAAGGAAACGGTGGCAGCTCAGCTGCCTTTGTGAAGGTTTGCATGGATGGTGCTCCATATCTCCGCAAGGTTGACCTGAAAATGTATCAAAGCTACCAAGAACTTTCTGATGCCTTGGCCAAGATGTTCAGCTCCTTCACTATGGGTAAAAATAATAATAAAAAAAAAAACTTGGTTTTTTTCTTTAATTACAAAAGATATTTCTTCTTAGTTCTTGCACATGCAATAATGAAAAAAAAAAAAAGAAAAAAAGAAAAAAAATTGATTTGCTTTATTTGTTTTAAAAAGGTGAATATGGTACACAAGGAATGATAGACTTCATGAATGAAAGGAAGTTG

**>CsIAA22**

NNNNNNNNNNNNNNNNATATATATAATATAATGTCTAAGTACGATTATATTTTTAAACATTTGCTATTCTTGCATTTATCAATGAAAGCATGCTTTTATTTTGTTATTAATAAAAAAAAATTGGGACAAAATCCTCAACTAGGTAAATTATGCTACAAAGTTTTAAAAAAAGTTTGACATATTATATACATATCTATCTATCTATATATTAAATGATGAACATGGGACAAACAGTGTAAAGAGGAAGAATTTCATAAATCTAGTACAGAGAATAGTATAAAAGCAATTATGGAACCAAGTCAACTATCTATTTTTCCTTTTCTTATAATTCTATTTCAAATATTTACTAAAATATTAAACCTATTTACAAAATATAGCAAAATTCATCCAACTTTTCACCGTTGATTTGAAATTTTTGCTATAAATAATTCCATTTTTTAATGTTATTCGTGACAATTCTCCAAGCTTTATGGTCTTTATAAATAATTTGGCTAAATTTTGTTAATCATCTCTAAATTGTCTTAAACTCAGGTTGGTAACATTGTTTAGCAACACATAATTAGGCAATACAAACCATTAATCTATTGCCATGTAAAAAATGAAAGAAAAAAGTACCAATTAATTAAATGGTAGGTTGCTATGCTAAATTATATTGATTAAATTGATCAATATATGTTCTTATGTTTGATTTAGTGAATTGCTATAGCAAGAAGCTTTGAGGTTAGCAGTACTAACTGAAGTGATATTGGAGTTTTGTGATGAACTAACAATAAAATTAAGAATCAACTGCCCTTGGAAGGCATTCAAGTTTTGCGGTATATGAATAGTTCAAAGGAATTAATTGATTTAAACTTTAGTTACAAAACTTCCATGCATATAATTAAGATAGATTAATCTCTATTAGAATTACTCATTTTATGTATAGTTATATGATGTGTTTATCATTCCAATATTTTTTCAAAAAACATATTAAAATACAAGGAAGGGAAAAATACATTTTCTAACAAAATTTCCAATCGAAAGAGCGCTTTTGACTCCTCGTTTAAGGAAATGATACTACAAAAAAAAAAGTTGCGGTGAAGCTAGACTTGCAAGAGCATGAACAACTTTATTGCAATCTCTATTATAAAAAGAAAAGCTTAGAACATTTACTCGGGAGCGAGAAAAAGTGTTACCCTTAACAACATTGGACGCCTTTAACTAAATGGGAGAGTAAATACTCAGAAAACTTGTCTTTGATACACATAAAAGCTTACAACCTCACTTGGTGTTTTTAAAAGGAAAAAAAAGAAAAGATTTTGACACGTTTTGATGAAGGTCAAAACATGGGTTTGGGTTTGAGTTATGTAAATGTTCAATTCAACTAGTTCTCTGCATTTGAACGAATCTGCCATATGAAAGCTCAGCAGTTTCTATCTTTAATGCTTATTCATCCAAAATAAGTCATCTTTCTTGAGTTTCTCCTATTCGTCTACCGACTCAAACACACTCCACCACGACTTCCACTTACAAACCTCTTGCTTATCACCATCATTTCAATATGATAAAGCATTCTCAATATTTTTTGGGATCTTTTCCCCCTTTTACTTTGATCTTTTGCTCCTAAAGATGGTGGGGTATGTGGCTGAAGCATTTTCTTAGTGGTTTATGGGGTTTTTTTTTTTTTTTTTTTAGCTGGGAATTTTAAGTTGAGGTAGTGGAAAGCAAGAGTGATCTAATCTAGTTTATCCCCTTTTACCCGGACCCCCCATAGTTTCATCTGTCGTTTTGTGGTTGAAGTCAGAACCTTTTCTATTTGCTTTAACTAATGCACACCACCTGTTGGTTATTTGTTCTCATCCAAAATTTCTCTCTGTTTTGCTTCTTGACATATATATGTATTATATTATATTTATATATATATGTATGATTTTTGAGAAGATTGTTTGAAGCATAACAAAAGCATTTTTTTTTTTTTTTGCATTCAAGCTCAATTTATAACATTTTCTTAGAAGAGAAAA

**>CsIAA23**

ACCTAAATCCAATAAAGTAACATCATAAGTTGTCTTATGAGTCTTGAGCTGTGTGNNNNNNNNNNNNNNNNNNNNNNNNNNNNNNNNNNNNNNNNNNNNNNNNNNNAAATATTTATTTAAAGGATTAAATTGATAGATTGGTTAGTTAAATAATAATGGAAAATTTTAATCTAAAGCTAAGATTACCAACTAATTTGAAGTATATATTAATTATAAAAAATGTTAATATTTTTCTTACATAAGATGGAATTTAATAGTACATTTTGGAAATTAAGTGTGTGATTAATGTATAACAAAGTTGATTAGTGTGTTGCATGTGATGGTTGACTCTAACTATTAGGCACTCTCTTTTAAGTTCCAATTTTCACATTGCCAATATATATGAAAAAATTTGTTTGATATTGATCCCTCACATGCCTAACTTTACAATTAGTTTCCTAATTTTCAGTTGACGTGTCTCATTAATATTCATTGAAAAATTATGGAAAAAATTATCTCAAATATATCATTTATATCAAATCTTACTTGTTCAAATAAACTAATACTTAAGAGTTTTTTTTTTTCTTTTTTTTATAAAAAACTTACCTAATTAATTTGAAATGGAGAAATGAGAGTGTGAGAGAGTCAAGTTAATGAAAAATTTTGGTCGCTTGCCATGCATATACAAATTAATAACAAACAACGGAACAATTATTCAATGAGTCTACCACTTATAGCGTAATATACGGTGAGATGTTAGATATAAAAAAAACTCGTGATTTCTATTAAATACATATTCAAAGTATTTTTTTTAGCTTCTTTCATAAATCCAATTGTGTAAAACTTTCAATTATATTGTGACTGATGAGATATAAAATGTCGATATTGATAGAAATATTTCGATGGTTCAATCTTATAAAATAACAAGTAAAAAATATCGATAAAATATCAACTTATATTTAGAAAAATTATGAATACAAAAGTTAAAAAGGTTTATTTAAAATAATAAATAAGTATTTTGACTCTTAAACAAGCTATTTTATATTTATCTTTTATATCAAATTCACGAAAGCAACATTTTTGTTGCTTATATTTTCTTTTTATGTTTTTATAGATTTTGCTACAATAATAAGGAAAATATTGGTTCATCCACTACGTGAAATTCACATCCGTATTAGAAAATTTGAAAAGAAATTTAATGTAATAATTGTGATGACTTCATGACTAAGAAAAAACAAGGTTAAATTTTCATAATAATAATTTATTTGCTGGATTGGAACAATTTGGTCCTTGTTTTATATTATTAGAGGTATGAGAAGAAATAAAGTAATATTTTAAACAATGCATGTACCTCATTTAAGTGATATATTTGTAATTACGTAACTCACAAGATTTGTCGTAAAATTCCCCCCTACTCACAAGAATCATTGTAATATTACGAGAAAAGCACTTTGGAAGTTCTGGTAAATATATAATATCCTCCCAAAGGATGATCTCATGGTTAAAAAATAGTTTTATTTCAATTTGAATTTTGAAAAGAAAATTAAGTTAATTTATATCAACATCGAAAATGCACAACAAGAAATAAAAACATGTAATTCGACTTTTAGACAGTATTAGAAAACTAGAGTAGAAAAGAAAAAGAAAAAAAAAAGGGAAAAGAATAAACTATTTAAATCAATTGACAAGTCATATACTTTATTGCAATGAATATATAGAGAAATTTTTTTAAAAAATAATAAAAACTTTATAGTAGGGACCAATAATTAATGGATTGATATAAAAATACAAACAATTTAAATATATATAAATAAAATAAAAAAGAATGGAGGTAAAATGATTTTACATGCAATCCAATAAAAGAGAAGTGAATTTTGGTTGCTTATATTATTGGGTTGCAGTCTCTATTAAAAACCAAAGGCCATATAATATATAATACACACCAAAGAACTAGAAGGCGACACTACGAGAAGAAGAAAAAGACGACGACAGTTTAATAAAAAGCTTTCTTTTGATTCTAT

**>CsIAA24**

CTATCCTAACCACATCTCTCACGCCCCCCACGAATGGCGCGTCTCTACACCCTCCACACAAAATACTCTTTCTCTATTTCTTTCCTTCTTTCTTTCTTTTCATTTTTAAATCAATTTATACTATAATCTTCACATTACATTCTCTCATCATATGGTTATTCGATATGAGTGATTAAATTTATTAAATATTACATTGGATATGAGATATGATTAGAGTAAGTTTGAGAACTAAATAGACCAAAATTTTATCACTTTTTAACTCTCTTTTAAGTATTTGTGTTTAATTCACTCCCTACTCCCTTAAAACATTTTTGTGCTTAATTCACTTTTTTTAAAAAAAAAAATTGTGTAATTGTTAACTTCATCAACCATTCATCAAGTTTGAAGGGATACTTGTAAAAGTTTAAGAATTTAAGTTCTCGTTCATGTGTATTTAATTTATGTTGATATGTGGTTATATTTCTATCAATATTTTTACATATCTACGAATACAACATCAACTTATACGATGAGTTGATGTTTGTTAATAAGAAGATAACTCGACTGACATAAAATATGAACTATCAATCAAGAAATCTGAAATTCAAATCTCCATCTTGTATATTATATGTAGAACTTAACAAATTAACGTTTTTATTATATCATAAAGAATTCACAAAACATAGGGAAAAAAAGCAACAACGCATTATGTAACTAATATAAATATAATATAAATGATAAATATTTTAGGTTGCTATTTATTATTGATACATTAATTTGTTTTTTCTTTATACTTTGTCTACAAATATCCATTGGACTTTTTTAAAATTGAGACTCAGGGACATTTTAGATCTTGTTTTGTAACATATATCAACTCGTAATCATCAACTCGACAATAAAAGTACGAATTTTAATTTCTTTTTATTAGTTCAACAACTTCAAACCTACCAATGGAAAGTGTATACAAATGTAAACTAAAACCAACTGAGATAGAGATGGTTTAATTAAAAGAGAAGCAAAGAAAAGAAGTCCACATGAAATGAACACATGGAATAGGAATGAAAAAGAGAAAGGGACAAAGTTCAAGAATAATATATATATTTTTTTAACTATTGTACAAAATAGAGTACCTAAAAAAATAAGACACAAACGAAGGTAAAACTAAAATTAATGTAAAAGATATGGTGATTTTTGTGCCGAAGAAAGTTATCTACCAAACTTTCTCTAATCGAGTTACACCTACGTAGCTGACGACTAGGTCAAATGGTGTGATTGGATTAAAGGGAGTCGTGTAATAAATGTTGGGACGGTGACGACCACGAAGAGGAGACAGTGGCTGCAACGACGGAGGAAGAAGGGAAAAAATAAGGTAGAAAGGTTTTGCCGAAAGAGATGTGAGAATGTGGTGTACCTCGTCCATGTACAACTTCTATTTGAAGTTCTGGATGAACGAGACATTGAAGTCATAGACTTAGTCCACGACTCTGTGCATGCGTGGACTACTAGAGAGATTGGAGAACTTAGTGGAGTCATGTATTGAGTACATGATTTCAACGCCTTATTATGTAACAGGTCTAGGATTTGATCTAGTCGTAGATGGAGTACACATCTTGAATACCCTATTTTTTATGATCCTCTTTTCCGGTCTTTATTTTTGAAATTATTTTTAAAAATAATATTAAAAGGAAAAATAAGAGAAAGGGAAATGGTGTGCGAGTGGGATTGAATAATAAGGTAAAAAGAAGTTTGGCTAAGTGTTGGAGGTTCAGAAAACAATGTGGGTAAGAGAAAAGGAACATGAGAGCAATTGGGTCGGCCAAAGGCAGAGCATTGATACGGACATCTTAGACGTTTTTCCGTTCTCTTTCCAACACTTTGTCCCCACCATCCTCATTCTTTGTCCCACACCCGCACGCACAACCCCTCTTCTCCTTTTAAACCCACATCCTCTTTCCAATTTCCATAATTCAAATTCATCTTTTGTTCTCACATTTTCTTCAATCAATCTCTTCCCCTACCTA

**>CsIAA25**

TTGAAGTATGGGTTGATTTTGTTTGAAAAGCTTCATTTTAACTTTTGAAGTTTAATATTAAAAAACGGAAAAAACAATGCACAAAGTAATTGAGGGGCGTTTTGCCACTAAATTGATGGACACAAAAAAGATTTATATCCGCTTCTGTGTTGCTAACAAAGTGAACAAATCTAAATACATGAATTTGCAATTAAAGAAAAGTCAAATAAACGAAAGAATAAAAAATTGGGAGGTGGGGTTGAAAGAAGAAAGACAACAACGACGTCTATTTGATGTTCATATGATGCATTGCACCATGTGTCTTTATATATATATATATATACACACATAATATTAACTTCAAACATCAAAACTTAAAATGAAATATTTAAACGTTTAGTGATATAATTGAATTAAATTATAATCATATTATTGCACTGGTTGATTGATTTTGTTAAACGCAAAATTTGAAGAACATGATTGGAACCATTATTTCAACAACATATTTTTTTCATATATGAAAGATTCATTTGCAGATTCGCGAAGCGTTTATTCACGGCCAACATAACTTTAGCTTTTATTCTCCTGCAGATCATGTTTATTCTCTCTACATTGAATGTTTTAAGTTTACGGTTTGCGTAAGCCTCAATCTTCATGTATTGCTGTCAAGTGTTGCTGTGTTCTTTGTTAAGGTTGAGATTTTGATAGTCATTGTAGTAGGTCTCAAAAGAGGTACAAAAAATCTTGTACTTGTCATTGAGACTGTTTGATGACATGGTAATCATTGTAAAACTCAAAAGGAGTTTATTTTGATAGAGTATTTAGGGAGAGCCTAATATGGTGAATGTGAGAAGATCTCACTCTTAGGGGAAGCGTAAACATATTGTATTCAGAGAATCTTATATAAAATTTGAAGAAACAATTTTTAGAATTAAACTCAAACTTAGAGAAAGTCTAAGTTGAAACTTTGAAAAACAAGGGATACATGAAGTTGTAGAAGAGAGAATTTCTTAGATAAGTATTTCGATTGTAGTTGCTAAAATCTTTATATAGTAAAGTTTCTTCCACCAGATACATTGTGTGCTTGACATTATATCCTCTATTAAATCAAACACTTTTTCCAATATATATTTATATTTATATAATCTTACTATATTTTTCAAAAAAACAATGTTTTGTCCCCCATCACTGTTGAATTTTAAAAAACGCGCCCACATTCTCTTAACCTAACCAAAACAAATATTTATAATACAAGGGAAGAAATTAAAATAAGTGGTAGAAGTTCAAATAATTAAGTATATATACCACTCTATCCAAGAACTGAAATGCTATACTACTTTGTTATAGAATATCAAACAAAAGTAAAGCAACCGAAAATGGATATTGAGATTTAGGGTGGACTACTAACAACATAGGTTAGATATGTTTACGAACAGGAGTAGGAAGAGAGAAATTTATTATTAGAGAAGAAATTAGTAAAATAGAAATGTGTTATTGGGAAAAGCTTATAATAAGAAAAAGCAAAAATAATTGAAAATTTGAAGATGTTGCCCTCTAACCTCAATCAGTTGAGTTGAGTTGAGTAGCATAATATATGATAATGGTTTAGAGAAGAGGCAGAAGATGATAAGAAGAGAAGGAAGTAAAGTATAAAAGAAATAAAGAGTGTTTAGAATGAAATAAATTGGTGTAAAAGAACCATAATCCAAAGGTGGACAGAGAAAATGGGGATTCACATGTTGGTGTTGGAGAATCCCACCAAATCAGAAACCCGACCATCTCTATCTCTCTCTTTTTTTTAATTAAATCAAACACAAAGTCCCACTTTTGTTCCTCTTGTCTCCGACCGGCCGGCGACCACAATGCTATTCATTTTCCATATCTCATTATTATCCCCATTCCCCCATATAATTTCTCTTCCATCTATTTATATATATATATAAATATAACCTCTCATTTTGGTTCTTACACCATTAACAAAAACTCTCACATACACTTAATCTCATCTTCACAGAACAAAA

**>CsIAA26**

TATATTTTTAATTCTATGCAAATAATAATCTCAACTTTCGGTTTCTTAACTTTTATATATGTTCTATCTTAGTTTTAATCTACCAACGATTTATTTTAGAAAAATCATCTATTTATGTCGTTCTTATACCATAAATATAGTCATTTTAGCACTTATATTTATTTTGCTTTGAGCATGTGTATTAGTGTGTTTATACGAGCATAATTTTTAGTAATATAGAAAATTTGATAGTTCATAAATCAAACAACTCAAATTAGTTATAACGAATTTAGGTTATTGATAATTGACATGACCATTTTGTGTAAGTTGAAGGTTTGATCATCACGGTGTAAACAAAATACAAATTAAAATTTGAAATTAGACCTATTTATATTAATGGAAAACTCTAACCTTTCAATTTTGAATAACATTTGTTAGGTTTTTTTGTTTTGAAAATAATTAATGCATTGTTGATTTTGATTTTGTATGCGAAATCATTATAAATCAATTTTGGTGGTATGAAAGACATCCAATCGGATCTGACCTGATTAAAGATCACCGAGAAGAGAAAGGCAAACCAATAATGATTCGATTGTAAAAGATAAAGGAAAAGAATTTGATGTTCATGTGATCTCTTCAGAATATTTGCTGAAATTCTGATTACATTTTACATAATATTCGTTTTCTTTGAAGGCTTTCAAAATGTGTAAGAAAATCAATGGCGGATTGCGATTGAGCTGTACAATATGCTTCCCCCTGCAAATGCCAGCTTTTCATGTCAGTTACATCTCTTTTGGGGTCTACTAGGCTTTATCCATGCAGGCTTCGACATTGACACGACCAATTCAATCGATTTCGTCCCTCCAATGGCCGCTCGCTGCCGAGAACATCAGTTTCATTGCCCTAATGTTCGATGTCACGTGGACTTCAATTGAACTTGCCCTCTCGGTTCTAGACAACTCCTTGCCCTGATGGCCTACGCTTTTCGCTTGGATCCAGCTGCAAGGTAAAGAAATCTGAACATATACCAAACGTATGCATGGTGTTTGTTAATTTGTCTAAGACAAATCTTTTCCCCCCCTTTTTTTTGTTCTCTCTTTCTTCTATTATATGTACTAGTATACTCCGACAAAGGGAATCGTTTTTATTTTATTTGGGCAAGACAGAAAGGAACTCAAGTACGAAAATTATTGAGGTGGAAAGAACTGATGAAGGGAATGTTTTAGTACCCCCTATTATCATGGAACTTTGAATTATCAGAGTTCATGCATCATGATTCATTTTGACAACATTTAGCCGACAATTTGGACTTGTTTAACGTTTAATTGCGACAGGGAGCTTATGTGTTAACCCAAAATCCACTTAAGAAAATGACCCATATTCAGAAATGGAAGGGTTTAGATATTTAGCTTTTGGGATTGATTAGTTTTTTTAATGTAAATCTGCTCTTAGTCCTTCCTTTCATGATCTGATTGCAAAAGTTGATGATAGCTTAAGCATATCACGCGTACCGTGATGAGATTATTTTGGGAACTATTTAATTGATCTCTTCTGTCAATGGAATATCTGATATGTGTCAATATATCAAAATCATAGTACATAAGATGCTGTATTACCGTAATTAATGTTTATTTTATTTGGAATTTAATGGTGTTTTGAAGTGTTAAGCATTGGGAATTGACGATCAAGGACTTTGTTGAATCAAGTTAGGTGCTTGGCCTACTTAATTTATGATATGTTTCTACAGAAATGACTACATGACCAAACTTGTATTCATTTAGTAGTATTTTTCTGTGAACGTGTCCATATCACTGCCCTTCTTTCAGCAGACCCAACTTCCATTGTTGTCTCTCTCTCTCTCTCCTTACCCAGTTGTCTTCTCCTTCACTCTCTTATCCACACTGATACACGTATAAATAGACCAAAGTTGCTAACAAAACTACCCATTATTCGATCCACTGCTACTGCTTGCCAACATCATCTCATTGCTTTTGAATTATTTGAAAACAGGAAAGATTCTA

**>CsIAA33**

AAGAATCCATTGGATACATATGTTTTGTTTAGTTCAAATTATAAGTTGTAACCTATAATTTCATTTCCATCTACTTCACCTTCATGGTCCTTATGATAAACTAATAGCCAACATAAGTTCAGCTCAACTAACAGGTATGTATTTGAACACACGAGGTTTTGAGTTTAAATCTCCACTCCCATGTTGTACAAAGAAAAAGTCACATCAATTATAAATATTTTAAAAAAATGTTGGCCACATTAATGCTCTTTCGATAAGTTTTGTTTTATTATTGTAGTTTTTTTTTTCAAAGGTATTGTTTAAGAATGTAGGATGAGAGAAATTTAAACTTCTAACGTTGATAGTACAAACACTATGCAAAATGAGCGTAACTCATGTTGACTTCGATAAACTTTGTATTATATAAAAAAATGTTTTGTGTTAGACTACACATAGATACCAAGTTGATGGTCAGTATGTTGTGTTAACTTTCATCTATCCAACACTGTGTGATATATAATGAAATGATTAATAAATAAGAATTATTTTAATACAACTTTAGCAAAAAAAAAAAAAAAAATTGTTTTTATAACTGTGTTTTTCTATTTTATTTTATATATTCGTGACTGGTGAGTGTCCAAGTCAATTTATGCAAACTTCGATTAACTTCATGGAACAATTCGTCTAATTAATTATATATAGACCTAACACTTAACTATTCATTCAAAATATGTTTACAATCGAATATTTATTGACATCACAACTAGATATAATAAAGATGAAAGATATAGATGTAGATAGATCTTTTCTACCTCCACTCCTACTAGTGGCTTTCTTTAATTAGTTTTTTTTAATCTTCTGATTTAAACTTAGGGTATGTTAAAAAATATTATTTTTAAGTAAATTTTTTAGAATTTTTTTTTTAAAATGAGTTTAAAATCTAAACACTTATACGTTTGTTTAATCTATTTTATATGCAAAATTATATTAACGATGTTTATACTTAGATTAAGTTACCGTAAAAAACTATTAAATATTATAAACTAATTTCAAAAAGAANNNNNNNNNNNNNNNNNNNNNNNNNNNNNNNNNNNNNNNNNNNNNNNNNNNNNNNNNNNNNNNNNNNNNNNNNNNNNNNNNNNNNNNNNNNNNNNNNNNNNNNNNNNNNNNNNNNNNNNNNNNNNNNNNNNNNNNNNNNNNNNNNNNNNNNNCTAATTTCAAAAAGAAATACACGCATTTCGAATTTTTTAAAAAGATATATTTTTAAAACAATTAATCGTAGTTACTTTGAAATTATTAATTTTTTAATTACTTGAAACCAAAGGTTGATTTTATTTTTGTTGTATTAACATTTAGGACAATTCAAATTTAAAAACACAAATATGTTGAAAGGTAAATACATAACCCTACAAAAATTAAATATTTGACTTCCGACAACAAAATATAAATTAAATCAGATAAATAGTTTTTTTAAAACAATATAATATCAACTAATTATTTCAGGTGAGGGATGATGTTAATTAGTTTCTTGTTGTATGTTACCAGTAAGAAAATAATGTTAGAGATTAGTAAAAAAAAAATTCAACTAAATTCTTTTTAATTCGTTAAATTTAAATAAACATTAATACCATATTTTATCCTGAAGTCAAATTTTGGTTAGTGACTTCACTCACGTGTTTATTTATTTTGTTTTTGAGAAAAAAGTTTCCTCACATATATTTAATTAAAAATACTCAATCACATAAGTATGTTCTATAGCTGATTAAATGGTAGTAAGATTTTTTTTTCATTTCAAAATGTTCAAAAGTAAAATATTTAAAAGGACAAAAATGTAAATGTAGCAAATTTTCATAATATATTCACTGCTTGGATTTTGGGACACTTCTCTAACTTTTGTCTTATTTCTCTCATTATTATTTTTATGCCATCTCATATATATATATATAATTTTTCTTCCCAAAACAAAACAAATTAAACCCAATACTTGCTTCTTTTATTTCTCTCTCTCTCTCTCAAATCTG

**>CsGH3.1**

ATTCTCCATTAACTTTTAATTAAAAATTCCAAAATAACAATGGCCATGTGTACAAAACAATAAATATCAACCTATCCAAAATCATCCTAAAAAAAATTATATTCTACTCTATTCATCATTGTCTCTTATCTATATTATTCTCCTATGCAGAAGATAACGAGAGTGTTTGAATTTTTTTTTTCTTTTTTCCTTTTGTTGTCTAAAGCTAGATTCCTTTTTACAAAAAGAGAGAAGAAAACAAAAGGTTAGATTATTTTTCTTTTATATATATATCAACCCGTTTGTACTTCTTTTTTTTATATATATTTTTCTTATTTAATGTTAGGATAGAAATTAAACTAAAAAAAATGTCTTTTGGAGTGAGCCCACTTATTTATTGATATAAGTATTTATCGAGAATAAATTAGTATCGACTCCTTCCGACGATAAATATCTATTTGGTTATATTATAGTCTTATTGATAAAAAAAATACTTTTATTATAATTATAAATGTTTTCAACTGTTTTATCAATAAAAAATTTCTAAAAAACTTTGTTTTTTAATACGTATTTATTAAATATTTAAATCTAGTTAAACTCATGTCATACTCAAAATAACATGACTAAGATAACACTTCATAGTCATTTCAAGCCTAACTAAGCAGTTAAACATTTATACTTTTTCTAGAGATTCAAAATGGTTAGTATTTAAAAAAGAAATTTAATTATGATTTTTTTTAAAGATAACATACTGAAACAATATATTTATAAATCAAATCTATCACTTATTATAAATAGAAAAAGATATTTGGTTAGATTTAGGAAAATTGTTCTATATAATTTAAAACAATTGTGGTAATTGAATTATTATTGGGATATTTTTTAAAATAACAAAATAAATTAAAATATAGAATTTTTTTAAAAAATATAATAAGGTGAAAAAATATTTAGCCTATAGAGGATGTCCATAACATATTTGGAAAGCAATCGTTTAGATTTGACTATTATTTCCTGTTTCCAAATCTAAACAATTTTTTTGAAGAGTTTTTGTACAATTGCTTATATTTGGTAAATAGTCGTTTGGATTTAATCATTTAAAGTTGGTACTCAAATCTCAACAATTTTTTTCAAAATTATTTACTAGGCGACTGTTTGGATTTGGTACATCGTTTACTTTTGACAATCCAATATCCTCATTTTTATACAATCTTTTATATTTGGGAGACAATCTTAAACCACAGTATAAACGATTGGAAAAAAATTAAAAAAAATGTAAGAAAAAGACGATGAAAAGAAAAAGAAAAATGTCAGAAGAAAAAATATGGAAAGAAAAGAAAATGGAGAACGAAAGAAAAGCACGATAGAAAGAAGAGAAAAGCACGATGAAAAGAGAAAAAATTGCAAGTAAAAAAAAATAGTAGTAGAAAATGGTCAATTTCTTTTAAGGAATAAAGACCATGACGAGGAAGACAAACCTGATTCTTTTTTTTAAGTTCCATTAGAGTTTTTTCATTTTGTTACGGAGCATAAATATTTAGTAGGTTTGTTAGATTTATGAAAATTTCAGTTAAAATATTTACAAACAAACATTTTGAATTGTATGTGAGTAATATTTTCTACTACTGGCTATTAACACGGTCTAAAAGAATATCACTAGCTAAAATAATAAAATCTCACATTTTGGTATAACTCCCTAAATATTTTGTTAAACTTTTTTATTTTTGACAATTTTGCTTATTATTATTTGAAGATGTTGACAAATTAGAATAAACTCACTATATAAAAGTAGTGCAAAAGTAAAGATTTACGATTATACCCTTGAAGCATCTAGTTTACTCCGCCCATGCCCGTGACCTTGAGTTTGAGTTTGAGTTTGAAGAACCACATTTTCGTGTCTATATAAACCCACTTTGCTTCTTTTTTTCCTCCCCACATCCAAATTCCAACCATCGTTTCTAAACACACATACTCACACACACCCTTCTCTTAATCACTCCTCTTCTCTTCTTTTCTACAACATC

**>CsGH3.2**

CATGAATTTAGTACATATTTCAACAAATCCAATTAAAACTTATCTTATTTTTGCAGAGCTTCAATTTTAATCTTAAAAATTTAAAACTATAATTACACCTACCAATCATATCATATTTTAGAAAATGGTTTTTGTAATTTGATTTTTTTTTTTTTTTTTTTTATATATAAATTTGGACTACTACTAATTACCTAATGATTTGGTTTAATATGATAAAAATTACAGAAGGAACAAGTCATTTTAAAAAAAAAGGAAAAAAGGAAAATCAATAAAGTAGAGTGATACATTTGATAAAAATAAATTTTATAAGTTGCTTTGAAGAGATATAAGTTTGCATTTATCATTTTCATTTAGTCTTTTTTAGTACTCTTTTTCTTTAGTTCATTTTTATTTTATCATTTGTTTACCATGTCAAATCAAAATCAAGCAATAATATTTATTTGAATTCATCTACCAACTGTTGCTAGCATATTCGATAATTAGAAAGACTCTAGTTGGCAGAATGTTTTGAGTTGTCTTGTGTTATGGTCTTTTTTTTTATTATTCATTGGCCTTAATTTTATATGTTAAATATTCTCACTGTGGTTGAGATTTTCTTTCAATATCATATTAACTGAAGTCACAATTTTGTTGCACAACTGTTAGTGAGTACGTAGGATATTGATCATTTAGTGTTATAGATTAAATTTTAAACATTTGAAAAATCTATCTTGACAAGATTATTTGTTCTTCATATATTATAAAATCTCAACGATAATGATAAATTGACATCGATATAAACAATACACTTTTTAGATACGATTGCATATTGTTCTCACTGTTTTGTTTAAGCATACTTTAGGATTAAAAAATAAGATAAATGCTAAATATGAATGTAAGTTGAGAGAAAGTTGTAGTGTAAATTTAAAAAAAATTACACTTTTTATTCACTTAGAAAATTATGAAGGTTTTCTTCTCATTTCTCACCAAAATTTAGGGAAGAAACTAAAAGGAAGAAAAAAGAAAATTATTTCTCTCAGCATCTTTCTTCCTCTCTTGATTAGGGTGGAAATCCAAAATTACCCTTTTGCTTTAATAACAAAATGAACAATTTTAATTACTTTAGGTAGATATTAATATTCATTTGATGTCTATATGTGTCTTTTTTCTTTCAAAATTTTCAATATAGTTTTTATGATTAGTCAGTCGACAATCTCTCAATAAAATTTGGTCTTATTTTTTATAAATTAACGTCAACATTTTTCTCGCATAAAACCTTCAAAGATGTGGTAAAGTTGGCGGTAGATTTATTTTGAGTGTGTAGAGAGAAGTGATAATATTGCATATTGCAAATACAATACCAAATTCTGATTCAATAGTTTATTTTTTATTCTAATTAAGTTTGAGTAGCGCATAGAATATTTTTAGTATGTAGTGTGGAACTATTTTAATTATTTGAATATAGATAGAAAAGAGTAAACATAATACTGAATAGTTAGAAGGTGTAACAAAGATTATAAAAAGTTTAAAACGGGAGGGTAGTAGCAGAATGGTTTTTGTTGTAATGGTTGGAAATACAAATTGTTATAATTGGAATTGGAATGGGTAAGATCCAAAAATCAGTAGACAATTGAAAAGAAATTTCAGTAATTGAAGCATGCACACGTGTGAGGAAGCAAATCATAAAGAGAATATTAACAAAATTCTTCACAAAATGAGAGAAAGAGACCCACAAAAATTAGATAGAAAAGCACCAAAGAAGACTGGCCTAAACAGTAATTTGATTTAATAATAATAATAATAAAAAACAAATAAATAGATAAGAAAAAGAAAATGGGAATATGGTTTTACAGATGTGCCCTTCATTTAATGCTTGTTTAGTTTCTAACTCAAAGCTGCCATCTAATTTTCTCTATAAATATCCATTTCTCCCTTTTGTTTTTCCCCCAACACGCAGAACCCTCATCCTTTCTATCTCCTCTCTTTCTCCTAACAAACTCTCTCATTCATTCATTTGAAAA

**>CsGH3.3**

GTTTGCCTTCATCTTCTTTGTCTATCTCTTTATAATAAAATATGAATGGAGAAAGTGTACTTCCATTTAAATTTTTTATTATTCATTAATGGGTCTTATCTAAATTCATTTTTTAAAGAGTGTCAAAATCAATGGTGATGGAGCCCAAATAGAAGTAGTATAGTAGTAGTTATAGAAGGAAACAAACATCAAAATTTATAACTTTATATTGTTTTTTCATTAAGGGAAAATGGACAAAAGAAAAACTAATAATTAAAACCTATTTGTTTGTCGAGTGGAAAGAGCAAAACGGGCTGTTATTTAATTCAAATAATGGAGCAACATATTCCTAACACTAACCAATCAAAATATATTCATTTACGATCAAATTTTTTAGACGGAATTGTAAAATAATCAACCACCTAGGTTGGTTGCTAAATAATGGGCATGTGATTAATAAAAAGCTGGGAGGAAATAATTGAAATTCATGCTACCTATTTATTTAGAATTCAATATTTCATTACTTTTTTTAGCATTCAAATATTATAAGGTTAGATATGTTGTCCGGTGAAATAAATTGAGATACGCGTGCACTAGCTTAGACACCAAAATGTATATATATATTTATTTTGACAAGAGTGGCATATGGGAAACAAGTTACTGAAAATTGCAATGTGTGATCTCACAACCCACAATACGATAAAATTTATAGTACTACAGTGTTATGAAAAAGATCTCCAACATTATCTTTCATTCATTAATTGTCATGAAAATAATATCTAAGAAATTATAGAGTCTATAAAATAGCTAAATAATAAATTAATAGATCATTTGATGACATCTTAAGTAATGGATGAGGAGCATGCTTAGTATTAGAAAATTTGCATCCAAACAATTACTTTTTCATTAATAATTAACTATGTCCTTATTTTGCATAACAAATGATTTAGGTCAAAAGCTTAACTATGTCCTTAGTTTGCATAACAGTTTATTTAGGTCAAAGTAAAGCTTTTGACCCAAGTATTATTTTTCATTCAAGATAATGATTTAACTTTTAAAATTTAGTATTATAATCATTTAATTTTTTTAAAGTTAAGTATATAATCATTTGAAGATAGTTTTAGTGATGTGTTTGAAATTGAGAGAATTTTATCTAAAATATAACGATATATATTTGAATGATTGTTTAGATAAGAAAGATGAGATTTTGAAAAGTGTAAACATGACTATATAAATTAAAAATGAGATGGTAAGTCGGAAAATAAGAAAAGATAGTTTAAATTGAAAACTATTATAAAAAATGAGTTTTTTTTTAAAAAAAAGTATTACAAATAGAGTTTAAAAGGTTTAAATCTAAACATGAATTTTAGATTATATTAGGATACAAATTTATTTGACTGAGAATGATGGAATATGTTAAAAAATACATAGGGTGGATGGATGGAATATGTTAAAAAATACATAGGATGGATGGATGGAGTAGAATAGGCAGTGATATGATTAGAAGTAGTAGTTAAAGTAACGAATTGAATTATGGGTTTAAAGTTGTTGTATAATTAAGTGTATTAGAGGCCAAACTTAGAAGCTAAAATGTGTTTTAGTAAGTGAAAATGCAAAATCAAGAATGGAGGGAAAAGAAAAGACCTAACCTTCACATGCTCATCTCACTTGCTGCAATGTCTACGTGTTGTGGGTCATCCTTTGTCTCCATTTCCCCCTACATTTCCTTTCCACAACACAGAATTCCTCTCTCTTCTCCCCCCCTCTCTCTCTCTTTACGCTCCGTATAATTCCGTAAGCTTCCTCCCAACTCCCTCTCTCTCTCTCCCTCTCTCTTCTTCATTTCTTTTTAAATATTTAAGTAAAACAACTCTTACCCCACCCAGTCAACCTTCCTACGTGGCACTTCTCCCCCACTATATATATCCTTCCCTTCTCCCCTTTCCTCCACCGCCCCCCCCCCCCCCTTCTTACTCATCACCCTCCTTCCTCTTCTTCATCTTCTTCCTCCCCTTCTTTCA

**>CsGH3.4**

CAAAACAAGTTCTTATTTAAATGTCAAATGAAAAAATAATTTTCTTTTGTGAAACATATATTAGCAATTTTATATGTTTATAGAATAACATATTGTTAATTATAAGACAAAAATCGAATGCAATATAATGGTTTTTTTTTTTCTTTCATTTGATTTTTTAAAATGTAGTGTTTTGATTATCTTTTTTTTCTTTTGAGAAATTTCAATCTAAAAAGTCAAAGTTTTGCAACAAGTGAGATATGGTAAAAGAAGAAAAGAAAACTAGAATTAAGATATTTAAACACTTTAAATTAACTAATCAATTAAAATTAATAGTCAAAATTAATGGTCAAAATTTAGATATTATACACTTAAAATTTTTGGTGCATGGTTCGCACTTATAATTTTTTATAAGATATAATATTTTCTTAATTATTTGTTAGTGGACAAGCTGACTGAGTGTTCAAAAAAAAAAAAAAAAAAAACCAACCAAGGGAAAACTCTACCTCACGTAATCAACTGGGCTTTGAAAAATATAATAAACTAGTTCCTTAAATTGTATTTTAAACGAAAAAGTAATTAAAGTAAAAGTTTTGAAAGTTTAGAGTAAAGTTATATATTATATAAGTTTAGCATTAATTATATTAATTATTGAAGGAAAAGGAAACACATTAAGTTGGCGAAGAAAACTATGTAGAATTGCAATGCAACTGTCATAAGTTTAATTTAAAAATAAACAAAAACAAAAACAAAGTGAATTTTACCTACCCTCTTCATTTTTAAATGCCGAGACAGCTTCAAAAACTAAAATCCTAATCAACTGGAAAAGGAAAAAATAAAATTAATAATCAACGTCATGTTCACGTGATACAGCACCAAATAATAATTCAATAATTCCCACAATGATCTTATCCTCAATTAATCTATTGCCCTTCTATTCTAAATCATAATCTCATTACCTTTCTTTTTTCTCTTTAAATTAAATCTCATTGTTTTCTGTATAATTTAAATAATATCATGATTAATTAAATATTTTCATGTCTCCGACCAATTTCTATTAAAAGGAAAGTTTTTTTTTTTATCGAATATTTTGTTTAACCTCTTCTTTCCATCTTTCATTACCCTATGCTTTCCATATTTGCTTATACAGTGCCTGAATCAAAATATGTGTGTTAACAAAATATCATATGAGTTAACTCGTTTGAACATAGTTTAAATTCATAGAAATTTGAAAATGAAGAACAATTATTTTTTAACAACCATTGTAATTTTTGAAACTTGGTTTAATTTTTGAGTAAATAATGAAACATATAAATTAATGTCTAACGAAAAAAACCATTGGTAAAATCCAGACTTCATATTTGTTGGATAATATCCTTAAAAACCATAATGATAATTTAATTAATTAAGATACCATTCATGGTGATCAACCTCCAGCTCATAATTGATACAGTTATAAAGTTAAAATCTAAGAATATAAAAGGTAAATTTGGAAATGGTTTGATTGGAAAATGATTAAATTAATGAGCGTAAAATGATTAGGATGGGATTTAGTCGTTAAGATGAAGATACTGAAATTAAAAAGGAAAATATGAAGGAAGGAGAAAAGAAAAGAAAAACATAACCACAGTTTTGGCCGCCTTCCTCCCGTTCCGTTCCCTTCAGAAATTGGCTGCTTCCGCCGGCAGCCGTCGTTATCTCCAATAAACACGGTCCTCACTCCAAGACAAACCCTTATTTGACCTGGCACAACATGTGGGCCTCACCTTGTCTTCCCCTTCTTCAGCACACCTATGTGTCTTGGCCCCACATGTTCCTTCTCCCCCACACCACTTTCCCTCTCCCAAATTTTCCTTTCTTTCCTTTCTTTACATGACGTGGATATTGACGTGTCATCCACACCATGCATTTCCATTTCCCTATATAAATCCCACACTCACAACTTCTCTACTCATCTCTTTTCTTCCTCTCATTCCTTCTCTCCTCGCATCCTTTCAAACTCGTTTTAATTTTATCAAATA

**>CsGH3.5**

AGTTATTATTTTTTCTTTTTTTTCTAAGATCGAGTTGAATTTTAGAAAGGATTTAGGTAAGTGGATTTCTTGTTTTCTTCACGTTGCACCTTTGTCTATCATCTCTCGACTCACCCTCCAGGCCTCTCATTTCACCTTCATGACATCGCCGAAGTCTCATCTCCCATTGAACCTTCGCCTCCCATCTCCGCTTAGCTCTCCCTCTCAGTTTGTTATGTTTAATAACTTTTTTGTTACTTTATTATTAAAGTTGTAGTTCATTGACGTTTATAGATAATTTGGAATGTTGTACATAGTTTATGGATGATTGAATTTATGGATACAACTAGTACAACAGTTTTAAATTTAATAAACACTAGAAATCAGAAAAAAAAAAATATACAAACATGAACTCAACCCATATTTTAAGGATTGAGTTGGGTCGTGAATATTAGTTCAGATTATTCGAGTTGTCAATCCAACCAATTTGAAAATTCGTGTTGTTTTTAAAATGTCCTGCAACTCAATCCAACCAAATTTAGATATCTGTACCGTGTTGGCTTATGAGTTTAATTTGATATTGAGTTAAAAAGTAAAAGAATGTAAGAACTAATGAATTAAAAATGTATGATTTTAGTTATGTGACATAAAACTATACTAATGTATTGAGACGAGCATCAGACGGAAAAAAGACTTTCCAAGTTTGTGTTGTTGGGGTGTGGTCACATTGGATATGTTGCAATATACTTAAAAAGTAGATTCACGTAAGCATATATTTGACCCCACCTATCCAAGCTTCTAGAACTCACATTAACTAATCATTCAAAGCCCAATGAATTATAACTTTAGTCAAAGACGCGTGTGGAGCCTTTTTTTTCTCTTATCACCAAACAAACACGTGTGTTTTTCATGTCAACTTCGTTACTTTTATATTTTTCATTTTATTTTTGAGCAAAAGCCCCAAAAGACTATTCAACTTCAAATTTAAATCATACTATAAATTTAAACACCTAGCTGTGACATTTAATTGAAGCACAAAAAATACCTAAACAAATCAAAATATAACACAGATTATAAGTTAAATATACATACATATTGTTAAAAAAAAGTTTGCTTAAAGAAATGGATGATTTTACGTGTGTACAAATAAGATGGATGCAGCTTGTCTCTTTATAAACCTGAAGACTATTATTTTTGAAAAAACTAAAGATAGTACATTTTAAACTTTCAAGTAATTTCAATATGTATTTTAAGGATTTTTATCAAATGAATTAACATGAATCTAAATTTGCTCAAAAATAATTTTTCTATAGTCAAATCTAAACATATCCTAAATGATGAAACATGAATAAAATAAAAATATATGTTGAGTGAGATTGAAAATAACGAAAATTAATAATAAAAAATATAAACATACCAGATCATAAACTCATACAGAAAGCAATGAAGGTTGCAGTATAATTTGAAACTTAAAAGATGATGTAGTTTGAAGAAAAGAAGAATGGACAACGGTGAGGTACACTCATTGTCCTTATTCACCAATCAGTAATCACAAGAACCATATAACTCACCCTTGACGTATTTCACTACACGTCAGCCATGGCGTTTGACACTAAAAATAGCACTGAAAACAAACATGAATGATTGGTTAATACATCACACCGTTGGCCGTCCTTCCCTTGCTTACGAGTTGTCTACTCCAGTCGGCACCCGTCTTTGTCTCCGCTGGACAAATTCAGCTGTAAAGTTCCCCCAATCTGATCTGGCACCCATTGTGGGCTCCCCTTGCCCTCCTCTCCTCACGACACGGCTACGCGTCCGCCCCATCTGTTACCTTTTCCACGTAGCACTCTTACTCAAATGTTTTTCCCTTACTTGCATGATGTCAACTTTGACGTGGCAACACACTTGCTTAAGTCTATATATAATACCCAAACCCTTCTTCTTTCTCACAATTCAACTCACAGCGCCACTCCAACTTTACAATCAAACATTACTTTTTTTCTTTGACAATAATTATA

**>CsGH3.6**

AAGAGAAACTTATATTTGAATATGATTCAAATTTAATTTAAGTGAAATATAAGATATTTAATTTAACTATTAATTAATTGAAGAATTAATTAATAATTTAGTTTTATTTAACTAAACTTGATTTAATTAAATCTATAAAATATTGGGGTGATATTCATTCAAATCTTATTTATGATATTCGTTACATTTATTGAGGAATTTGTCACCCATTCCATTTTTGGTTTGCCAAATCTACAATATCTCGTTAGTCTAAGTAAAATAAGGTGTTGTATTCTTTTTATGATATTCGTTGAGGAATTTGTTTTTGGACAATTTGGTTAATGCCTCCTCTTTTACTATGCCTCTCGTCTCTCGCAAGGAGAACAAAGTAGCTCGCTCTTTACACGTTATGAAATATCCTTTCATTGTTTTACTTTAGTTTCAAAATATATATTCTTTTGGAGAATTGTCTTCCAACTATTCAGAATAAGAAGGTTAGGTTGTAACTGTAGTTCCTCTTTGCCAGGAAAATTTACATATATATCACTAACATCACCATGTACCTCAATATATTTAAGAGCTCTCGAACCACAACATACAAAAGAAACATTTGTGATAACATCAAACTTTTACTTTGTTTTACAAAAACGAGAGAATGGGAGAGGAGAGAGAAAGTAGCTTTTATTTTTTAAGTTTAAAATATCATTCAAATATTATTTTTTTTAAAAAAATTAATTAATTAAGATAAATAAAATGAAAAATTATTTCAAATGACAAAAGTCCTAAAAATAGTACAAATATCACAGACTATAATGTATATGTGATACACCACGATAGAATAAGATAAACTACCTTCTTTGTGTTCTATCATGATGATATATGATAGATATAAATAATAGTTTATCTTAATTTTTGTGGTCCATCGTAAGTAGACTATAATATTTTTTTATATTTTTTAAATAGTTTGTTTCATTATAATATATTTAAAAGAGACGCTTGCAAAAATAGCAAAAAAATTTATGATAGTAGGGTCCATGTTATTATATTTTTTAAATTGTAAAAATAGCAAATTTTTGTGATCATTTGATAGCCCTTTGATATCAGTGATTACTAATTATATTTGTCATATTCGCAATATGTAAAAAAATTGGTGTCATGGACTATTTTTTTCTAAATGTTTTTGTGTTATCATGTAATTTTTCTATTTAAAATAATCCATAAAAATCAAAATTGCTTACAAACCTATTTTGCCAAAAGTTAATATAAAAAAAGAACCTAAAACAACAAAAATTAGGATACAAGCTTCACTTAATAATTGGAGAAATTAAAGATTTTTGGGTGTATTTTATGTTGCATTCATTTTCAAGCTAACACTTCTAATTACACGTAAAAATATTTTTTAAAAGGAAAATTGTCAAATATAGAATAGTTTTTTTCTGTGTTTTAGTGGTTTTGTTTCAAGAGTGCACAAAATTTGTTAAATTTTTCTATTATTGAAAAAATCCATTTAAAAATAGGGGTATTTTTTAAAATAACAAAATAAATTAAAATATTTACAAGCTATAGCAAAATTTTGGATTCTATGACTGATAGCCTTCATCACTATGATATATAATGAATATTAGTTATAGAATTTTTGCTATCTCCTATCTCCTTTATTTTTAATAACAAATAATTTTTAATAACATCCCTTTCTTCCAAATTTTTTTTTTTTTTTGAAAACACCAATATTTTCCAAATAACCCAAAATATAATTTAAAATAACATGAAAAACAAAATATGATTGGAAAATGGAGGAGTCAACAAAGGAGGAAGGTATAGATGCCGAATGTATCTATTCCATTCTCACACGCTCTATTTAATCTTATTTTTCAATTTGATTTCAAATCACAAATCTCTCTCTCTCTCTCTCTGTGGATTTCATCTATTTCTCCTCTTGATCTCTCTCCTCTCCCAATTAAGGTCTTCTTCCTTGGCTTGCTACATCTCTGTAACCCGAATCTTAAAAATAATCTACAAAG

**>CsGH3.7**

TAAAAAATCTAAAATGTTCATATATGTGAAACTTTAGATGGTATAAAGATATGTTTTTATTTAGGATTTATATCTTCAAGGATTCAAATGCCATACATTGAAAAGAAATGTTTCAAATAGAACGGTTATCTACTAGACTTTGGTCAAAGAAAGAACGCTGAGTTTCTAAATACAAAACTTCTTGCAGCACTTTCTTAGGCCACAACTGATGTAGATTTTCAAAGGTTATTATCTACTAACTAATCACACACCATTACAAAATAAGAGATAGTTGCAAATTTAGCAATTAGGTTCAAAATAATTAAGTATATAGCAACATATTAAAAAATTTGTAAATATAACAAAATTTGTCAAATTTTATCAATGATAGAAGTCTATCATCGATAGACCATGTTGCAAATATTATTTTATCACTTATAAATTATACGAGTTTATCAACGATAATTTTGCTATATTTTCCATTATTTTAAAATATTGATATATCCTTAATTATTATTCCTAAAATAACCATCAATTACAATTTCTCAAAAAAAAAAAAAAAATCGAGCTGACTCGCTTCGGCCCAACCCAAATGTGGGGCAGTTGGGTCAGACATTGGTGGTGAATCGTACAATTCAGGATCAGGTCAAGTCGGTTTTGACGTCAACTCGACTCAATCCATTTGCACCCCTACAATTTATTTATTATATACTAGTTGGTAGCACGTGCTATGCACGCTAATTTTCACACAAATAGAACGAAACTATATTTATTTACTCATGATTTTTTAAAATAATTTTTGGAAATACATTTTTGAACTTTATTTCTAGAGAGAGTTTTTCCTCAAGACTTGTTATATGCATGTATAAATAGAATGAGCTAAAAGATATAAAGAATTTGATAGTATGACTATGAAGCAATCCATTCAATCAAGCTTTAGATTGATAAGTAGAACTTTTTTCATATATCAATTACATATCGAATGTTTAAAATAACAAAAATAATTTTGGGATAACTTCAATGCTGCTTAAGGAAAATAACATTTAAAATTTAAATTTTGAATAATGCCCTCTAGAAGAAAAATATGTTTTTCACTTAAATCAAGATCATTTTAACACATTAAGATGACCAAACCAATAGATAAAAAGAAAATAATCAGCTAACAAAAAATAAATTTTCTTATTATTCAATTTTCATGAGGCCTAAACAACAACATTTTATTTCCCTTCTCGCTTATCTAATATTTTCTTTTTTTTCTTTCTCTATTTATAATGTTAGACAAAAACAAAAGTGACTAAGAAACAACATAGGAATCTCTCTTCAAGCTTGCAAAGCAGCACCAAAATGATGACATAAACTCATAAGATAGCCCCTCACCCTCAAATTGTGTGTAACCTCAACAACAATCATAAAACAAAGGGTTTTTGTAGCCTTGACTCACAAATCTTCATTTGAACTTGTTGGAAGACTAATTTCTTTACACACAACTTTTTGTAGTGACTTGAACATGAAAAAAATAAAAAAAAAAATGGAATATTTATACCTAAAAATTTGAAAAGGGAAAGAATTTGAAAGGTTGAAATAATTACAAAACATTTAATGTGAGATAAAGGGATGTGAATAGGTGGAAGATAAGGAGATGTGAATAGGTGGAAGATAAAGAGATGTGAATGGTATTAGAAAATTATTAAAGTTAAAATTATTAATATATATGTAGTTATTAATAATTAATTTTAAATTAAAAATAAATAAAAAAGTAAGTTAGAATATAAGAAAAAACAAATCTACTAAGAAATATGATTTGACGAAGTATCCTTTTTGGGATAGAAATACTGATAAATAGTATATAATATTAGAAATTGGTAAATCCATTCCATTATTTAAATTCTTCCTCTCTCGGTTTCCAGCATCCCATTTCTCATTTTCTTTCAATTTCACATCACAAATCAGTCTCTTCTTTCCCAAGACCCTTCTTTCCTTTGTTACATCTCTGTAACCCAATTCTTCAAAACTTCAAAAAA

**>CsGH3.10**

CACTTTTTAGGAAATTTATATGATTTTTTCACAGTAAAAGGACACTTAGCTATACTTTTAACTTTTAATAAGATGGTTTTGAGAGTCTTTTTTAATTTTTTTTTTTTTGTGATCTTGTGTTTTAATTAATTATATTATATCATTGTGAACTCTAATTGAAAGTTGAATACAATTGGAGACTTTGATTTTCTTTCAACTTAATTGACACAATTATTTACAAGTTTTCACCAAAGATGCCGTGAATAGTACGTCTTGTTTCTGTTGATATTATCAATTACTTAGACTGTGAGTGCTTGAATCATCTAGTAGAGTTTTGCACTTTTTTTGTTATTTGTTCACTCAACCATGATCTTATCTTCCCTTGTACAATTGTTGGCTATTGTGGCAAGGTGGTATTGACCAATTAGTTATCTTGGATGAAGTTCTTATTAAAAATATTACGTGTATTAGATTCTACAAAATAATAGAGAAGTCGAAAACGTTGAAATGTGCATGATTAAGCTAGATCTTAACACTATTTTATCTTGGCTACTTTTCAAAAGTTCATTTGTGTGTGCTTATCAAGAAGATGAAATCTGATTGGCATATACTTAAAGAATGTTAATTAATGCTCATTTTCAACAAATTTGTAGATATTTGAAGAATGCGTGGACAATGAGATCATGTCAATTCATTTATGTGTTAGTTTTCACTATTTTAGGTGACCCATACATATATTTTATTATATTACAACTTATGTATTGGATTCTTGTAAAATTTTTATGGAAGACAATGTAATTTAAAATTTCATTACCAATATAAAGATGTTTATAAACATATATGGATAACGGTAAATGCATCTGGTTGAATCCCAAACATTAGTTTGTTTTAAGAATAGAGAAATGTTGAAATAAGTAATTATATGATAGTAGAAGATGGGTTGTGATCTAAAAAAATAACAGCGCTGAAGAAGTATCAATATATAAGCATTTTTTAGATTGGAACAAAAAATAAATATGGTGGATAAGGAAATGAGGAAAAAGTGAGGTAAATAAAGGAATAGGCACATAAAGAAGAGCATTTGTAGTGTATTGTTGACAATAAACAATTTTGATATGAAAGATTTTGGAAAGTGTGATTAAATGATACAATTTGTTAACAGTGGAGGATTGAAGGAGAAAAAAAAAGTGTGAGGGATAAAAAATTGAGGTTGTTAATTCCGAAATTTGTAGCAGTAAATTGTAAAGCAATATAGGTGGAAATGAGAAATGTAATTTGTTTTGGTGTGGATGAAGTAAGTTATTTGTTAGTGTTGTTGGAAGAGAGAAATTGTAGAGTGCAGACTACGTGCAGAGATAAGAAAATTAAGAGGTCCCCACAAGGCCATGCCTGTTTCAATTTGATTTGCCCATACTCTTAATATCCTTACCCGCTCTCAAAACTCACCCTTTCAATGCATTTCCCTTTTACTTTAATTTTGTTTTGGAATACTCAAATTTATTCAATTTGGGTTATTACATTTCCCTTTCATCCTTCTTCATCTTCTTTTTTACTTTCATTCCAACCTCCATTTTGACAATTGACTATATATACTTTGGAAGGTCTAAAACATTAATTAAATTCATATTCTTTATTTTTTATTTCATAGGCTTTGAGGCTTTGAGGCTTTTTGGATTGAAACAAAAGATAATAGCTTATAGGTGGGTGTCTTTATTGTTTGGATGTGGCTCCATTCTGAAACACATATTCTCTCTCTTCCCACCCCCATTGTTACACACACACTAATCTCTTTCTCTCTCTTTCTTAAGATGATAACACAATGATGTTGTTTTGTGGTGTGTATTCTGTCGTACATTCAATTCGCCCGGTGCAACTTTTAGCAGTAGTAGATACTTTATTTTTTAGTGATCCTTCAAAGTTCGAACAAGTAATCTGGCCCCCTTCACCCCCTTTACTAGAAAAACATATAAACTCTTCGTGAAAGAGATCGATAAATGTAAAAAAAAAAACAGAGAGAGTAA

**>CsGH3.11**

TATTATTATTATTTTTTTTTACAAATAAATTAGTTTAAAAATGGAATAATTTGTTCACAATATTTTTAACTACCCATGAGTAAAAGAAAAGTTTAGGAAGGTTAAGGGGATAAATTCTTTTTTTTTCCCCCCTCAAGTGGCATGCTTTACCATTTTTTTATTTCTATTTATATAAAAAAAAACATGATTTTAGCCTGGGAATTAATTGTTATTTATGTTTTGGAAAAATGAGTTTAAAATTTGAAAAGTATTGTTTTCTAATAAAAGTGATTAAAATATTAAAGTATTTGATTATATTTATATATGTATTTTGTCAAAGAATTTAAACCTGGTCCTATAATTTGATTCCTTAAAGTTTGAAATTAATTTCTTAACTATACTTCAATATTAAAAAATCCAAACTCATCACATAAGTTATTAGTTTTTATATTTTCTAAAAGAAAAATATATTTTGGTTAATTGAAAATTTCCTAATTAATTTATTATTTAGAGAATAAAGAGTTGATAAATGAATGAAATTGATATATATGTTTTTTGTTATAGGTTATTGAAAATTGCTAAATAAGGGAAAATGAATAAAGAGGTGGAGGGTAAGGGTTGGAAGAAAAAGAGAAAAAGGAAAAACCAAATCTAATCTTCCAAAGTGGGGTAAAAAGGAGGCCAACCTTCAAAATGCTCCCATTAGGCAGATGCTGTTTTTGTAAATAAAATTAAAAACCATGTCCTTTTTCCAAATCCGGATTCGGAAAAATAACCCGACCCGCATCCACCGGCCATTAAGGGGTTCGACGGTCATTCCACTTCAATTCCTAAGGACGTAACGGTCATTTCATGCCCTTTTTTATTGCGAGGCCGTACTTGGACGTCGTCGGCCACCGGTGGATACGCCACGCCTATATATATATTTGACCGCCTGTTAGAATCTTCCATCGCATCCACATCCGTGTCTCTCTCTAGTCTCCATTGAAGAACACAAGTAGAAACCTCTATAACTCTCTCTCTTTCTCTCTCTCGAACCTTTTTAAGGAGGACAGAGAAGAGAAACACCATCTTCCTTTACTAACTCAACTTAATTAGCTTGCTGCGGACTGTGTGCGGAAACGTTTCTTGCATCACTCTCCAGTTCTTTCAAATTTCGTTTCAGGTAATTTCCATGCCTGGACTTTGATTTCGTGTTTCTCTTTCTTAACGCCAACGTGTGAGGGCACGAGAGTGGTTTCAGCTGGAATTGATGCCAATACGGCACTGTTTATTGATTGCCCATCAGTTTTCATTATAATTTGGAAATGTTTGTTTTCATTCTGATTTCATTATTTGATTGCCTTGTGTTGGAATTCATCGTCATAATTTGTTCTTAATCCGTTATTCAATCGTCGTTGACGAACTCCATAAATTTTCTTCTTCTTCTTCTTTTTTTTTTTTTTTTTTTTTAATTTTTAAATTTTTAATTTTGAATTGTGGGCTTTTTTTTTAATTTGTAAAAAACCATTTGTTCGGTTCTGTAAACCGGTTTAATACTACGAACCCGACTGGGTTATCGTCTTTGTTTCTGGGAATCGGAAAATTATATCGATAGTTGACTTGGTAGAATTCCGTCGGATAATTTGGATCTTCGGCCGCCGTGTTGGGAGGGCAGTTCAATCGAATCAATCAGTTCTTCTAGCTGTAATTGCTTTTCACTTGGAAGTTGCTGTTCAATTGTTGACTTATCAATGAGTTTTGCCATATTCTCTCTTACTTTTTAATTTTTCTTTTCGGGTTTTGCAAAAAAGATCAATCCACAAATCAAATTTACTGGACACTTGTCCTTCATCTTATCTAATGGACCCCTGCCGTTATTCACGTTTTGGGTTTTGCCTTTTTGCTTGCCTTATCGAGCAGTTTCCAGCCCCTTTACTGTCTTATAGATTTTGATGGGTTTGATTTTAAGCTAATCTCCGTCAATACTCCGGTGGTTTTTGTTTTGTAACAGTTTGTGACCGACGAGAATGTTGGAGAAA

**>CsGH3.17**

TTATCAAACTTACAATGTTGTCAACGTTTTTTTTAATGATATCAAACTTAAATAGTTAATAAAAAGAACCCATTGATTTAGTTTTGGAATAATTATTGAATTATACAATCATATATATTGTTATAATTATATTATATATATCTTTAATTACAAGGTTCCAAAAAATTGGTATGACTTGAAGAAGAACAAGTTTTTATTAATTAATGAAAACCAGAGGTTGTCTGTCTGATGGTTTGAATATTTTGGTCCAAACATTATTATGATCTTAATCATAATTGAATATAGTTCTCAGTTTTTGGATTTAAATATATATGTGGAACATACTGAATGAATAAGACACTGTTATTGTGATTACAACCCAACCTAACCTAGTATATATTCATCATACATTAATTCTAATTATAATCAAGCAGTTCATCTTTATCTCACTTACTTTTCAATATATACTACTAATTAAGCAGTGTTCATATGTAAAATAATTAATCTCTCTAATTTCCAGACAACAACTCCTTTTTGTGACTGTGTAAACCTTTCCACATGATCAAACCCCAAAAATAATTCTCCTTTTTTCCTTCCCTAAAGTCGATTTCCTCGTCATTTCTCGATTAGAACCCAAAAAGAGGGGTATAGCTTAAGAAACACCAAAAGTTTTTTTTTTTTTTTTTTATTTTATTGTATAGGAAATGGGAGTTGTATATTATATTTATTGATATGAATATCCAAGATGAAAGAAGAAATAATGGGAAAAAGATAAATAGAAAAGTGGTATCTAATTTTATTGAAGGCAGGAGAGAGCTGTAAAGTGATATGTCCATGATTGCCAATATGCATACGTTTTTTCTTCTTGGATTATTTGGTTGAAAAGACAGTTCTTAATCATGTCCTTTTCCTCTTCTTCTTCTCTCTCTCTTTATTCATACATATTATAAATTCAAGTATTCTAAGAACTAACACCTTTAACCCAACTCTCACATTTATTGTTCAAATTATACTTAATAATTAATTCTTCTCATTCAATCAAACATTTATTTTCCCAACATCTTAAGCTACGAGATCCAACCCCCTACTTCTAAGGAGAAAAGTCACATCAATTACTCTTGTAAACCCACTTTGACACTCTTAAGTTTTGAGTTTAACTTCTTTTTATGTAGAAGCATTTGTGAACCACACAAACACCTTATGCGCATTAGTACATTGGATGCATGTCAATTGATCTCTACGTTTTAAAAGGTTACGTTATGAGTTTGATTTTAAATTTGAATAGTAAATATTAATTAAGTGATAAATTTTGTGGTAACGTTTGAAAATCTTAAAAGATTAAATTTAGATAATTAAACTTAAAAAAAGAGTTTATCTATAGTAAAATTGTGATTAACACAATTGAGTGAATGAACTTACAAAAACTGTTTTACATACACACATACATATTTTTCTTTAAATCATTGAAACATGAGGCTATCTGCTAATATATATAGACACTTAATAGGTAGTTTTGACCTGTAGTTATATATATATATGTGTGTGTGTATATGTATATATATAATTATTCTTGTATTTCTAAACCATATTTTTAAAAATAATTCAGAGTTTAGCAGACAAGGCCACATGTGTAGGACCTCCATAGCCTCTTCTTCCCTTACATTTCCTTTTTGTTTTTCGTTTTTGTAAATTTTTCTCACCTCTTAATTTTATTTATATAAAAAAACACCCCCTTCAGAATCTGTTATTTAATCCTTTCCCCTTCTGCCTCCCTCCCATTACAGATACCTGGAAAACAGAATCCCCTTCAAAAAAAAAAAAAAAGAAAAAAGAAAAAAAGAAAAGAAAAAATTTGCATAGTGAAAATTGTCAGTTTATAGTTATACTTATGAACTGATTGAGAAAAAGGAAAAAGCTACCTTTCTTTTGCTGTTTCATTCATTGCAATGTTCAAAATTTAAAAGTCAATTATCCTTTTTGTTCATCTTCTTCTTCTTCAAAAATTTCTTCCTGCTGTAAAG

**>CsSAUR1**

AAGTACGTGGTTCTTATTTTGCTACAATAATTTAAACCAAAATTTTAAAAATAAATTCAATATTTGTTTGAACGCTTGTAATAGATACATTGAGAAGATATCGATGCACCAACAAATTTACTATAGGGTCGAGAGGCTCGAGCCTCTTTCAACTTTATGCTCGTTATATTACATATACATATATACATATACATACACATACCTATATAAAACGATACTGAAATATAATCAATTTTAGAAGTTAGTTTAGTGGTAAAATTGTCATCTAACTCTCTTCTAATCAAGATCCATTGTCGCTGTATGTATGCATATATTTATGATAATTAATTTTTTAATTTCAATCACATGGGACATTAGATTTTAAATAATGTTGTAATACTTTTAACTTCTAGAATTTCAATTGAAGAAAAGAACTATTTGAATATGCTTATTGTTGAACTAGATTAGAAGAGAACTAGACCTAGCTAGGTTTGGATTGATGTGACTTGGAGAAATTAAAAAAAAAAAAAAAAAAAGCTGAAATTTATATTGAAATATAATGGATGGATTTTGATAGATTCATTGTATTTTGTTCTTGTTTGGATTGCTTTATTCTTACAAATTTCATCTAACCAAATGTATAGGTGATCAATGAAACACAAAGTGTTAATTTTGAAACTAAAAATCCATGCCCTTTGATCAATTTAATGAAAACAGGTAGTGTTTTAATAAATTATGTTAAGAGTTTGTTTGGCTGGATTAGATTCAGAAAAAAAAAAAGGGTTAAAATACATTCTAAAACTACTCAAAAAATTTAAAAATTAAAAAAAAAACTTATTTTTTAAATTAAACACTTCTAAATGCATTTCAAACACACCCAACAACTCATATTTATATAGTAATTATTTGTTTCATCTCAACTTCAATTGACAATAAAAGAAATAGTTCACATTAAAATTGAATATAATGTTGACTTGATCTTTGTATAAACATTTATAAAATTAATTCAAACATAAACTTATCACCCATCCATTGCCCATCCTCAATTGTTGATTGTCATTAATTATGAAAATTTGTGTCCTTTCACTAAACTCTATAAAGAATATATTACATGCATGGAGCAATTCAAGTGCACATATAATATTTCATCAGTACCCTAACCCAACAGAGAAGCTAGCAATTAATATTTTGACAAATAATTTTATCTATATATAATAATAAAAAGACTACAGATTTGAAGATCATTCTCAAACGGATTATATTCGAATTCATCTTGAAATAGATACACTTATAAAAAGAATATTTGAAAAGAAGATATATTCAATGAATTATTTGTAAAGTAGCAAGTTATAATAATTATATAATTGAATAAATGACTTTTTTTACCCCAAGAATATACCAAGAGTAAAAATCCAAATTTTCCTCTTCCCTTTTTCCATGTGTTTTATTAGAAACCATTTGTTAATGTTTTTGAGTGAAAAAAAGAAGAGGAAGGAACATTCCAATGACAATCCAAATATGATGGGTAGCTGTTCTCTCTTTTTCTTTCCATCTTCCAACAATTATTATTAATACCCTTTTTTAGCATTACATTTTGTCTACTCTCAACTTGTAGAGACAACGAGAGTTCGTTTATAGACAAAACTAAGTGGATTAACCATAATTTTGTCATTTGTCAACACCCAACACCCTTCTTTTTTTTTCTTTTAATTTTATTTTACCTATATTATAATTAAACCCATTCATAATTATTCAAAATAAACCAACTTCTCTCCGTTGACAATAAATCCCACCTCAAATTTTCCATCCTAAATAATGTAACCATCTGGAAATGGCTGCTTAGATTAAATATTGAAAATCAAAACTTTATCCTTAAAAAAAAAAAGGGAAAAAAAATCCCTCCCTCCCCCTCTATATAAATCCAACCTTCTTTACAACTTTTCTTTCGGACCAAAACAAACTTGATCAAAGAATTCCTCCCTTTTCTCTTCTTTGCTAATCTTGGGGTTTTTATAATTTAC

**>CsSAUR2**

GCAACCTGATTTACTTAATTGTTACCCGTTTGGATATTGAATATGCAGTTCATATTGTTATTCAATTCATGGCCGCTCCTCATACTGTTCACTTTAGTGTTGGCCTTCGTATTTTGGGCTATATTAAAGGTACTTTGTGTCTTGGTCTACAGTTCTTCTCAGAGCCCTCTCTACTGGTCTTCTTTGGATTCTCTAATGCTGATTGAGCAGGTGATCCTATTGATAGAAGGTCTACCACTGGTTACTACTTCTATTTAGGTGATGCTTTCATCTCCTAGCGCAGCAAGAAACAAACTATTATTTCTTGTTCCAGCACAAAGTCTGAATATCATGCTATGACTAATGCCACTTCAGAATTACTATGGCTTTACTAGCTTTTTATTGATATGGGGGCCCCATAAAGATCACCCACTATTCTTCATTGTGATAACTATAATGACATTCAAATTGTACACAATGATGTATTTCATGAACAAACAAAGCACATTGAAAATTGCACCATATCATCTCCAAAACAACACTCTCCATCTCCAATCCATCTCCATCATTGACCAATCAACATATATTTTCACCAAAGCTCTCCATTTTCCTCGTTTCACTTAGTTAATTCACAAACTCAAGATGGTCTTACTCTACCAACTTGAGTTTGAGGGAGGGCGTTGGTGTAATTTTTGTGATTATATGTAATAATGATTCACAATCTTGTATTTATTTCGTATATCCTTTCCTTATTTATATACCTCACTGTGTATTATAAACATTGTATTATTATGAAAATAGATTAAATAGCTATTATCCCAAATAAAACCACAAAGATAAATTCATTGAAATAAACTTCAAAAGTACAAGATTGCACATAATTAGGTTTCCAAAATAACAACAAAACACTCAAACAAGAAACAAAAATGAATATGTAAAATGTAGAACGTGGTAACTATGCTCTTGAGATATAATCACTTATCTAATGTTCCAATGAATCTAACAAAAAGGTGGTGAATGATGATTATTAAGACTTTGTTTAATAATGACTTCATTCTTAGTCTTCTATTTTTGAAATTTACGCTTATTTCATCCCATTTTCTAAATTATATTTTCTTTTATTTACAAAAAAAATTGAATTCCAAGTAAAATCTTAGGGAAAAAAAGTAAATTTGTACATTTTGTTTTTAACCATAACCAAAAATTAGGAACTTGTGAGTAAATATTTATAAGGTTAATTTTAAAAAACGAAAGCAAACAATCAAATAGATAAGAAACAACGTCCAAAATTATATTGTAATGATAAATTTACCTAACACAAATGAAAATCCAATATTTTCTTTCATCAACGAACCCCTATCACTCATAAGATTCTTATTGGCATGTTCAAATGATTACCAAATAAAACCTTAAGGGTATTTATTGTCTCTAATCTTATTTTATCAAATTGAAGAACCAAGACATTGCCACGATGAATGTATATTAAAAGGTTATCGATTGGGTCAGAGATGGGGAGCTCATACTCGACTTGCTGAACTCGGGTGCCGACTCTTAGATTGGACAACATAATTCATAGATGAACTTTCTATTTTTGGTAACCATTTTAATAATCCAGAACTCTCAAGAAGTAAGCATAACATATTATTGTAAAGTGATTATTAGGATTTGGAATTGCTTCATTTCATGAGAACTACTTCAATAATTTTGGAGGCAGAAATGACAGTTCTAAAAGTGACCTTTCATAGAACTTTGAATGCATGGACTCAATTACTCACATGCTATGGTCTCCTCTAACTTAATTTCACATACTTCAGTCCAAATTTTCTTGCATGCCCTTTCCTCTCTCTTTTTTTACCTTTTACACCCTCCAAATCTTATCTCTCACACCCCATTAAACACTATAAATACACCTAAACTTAGAAAATTTCAAACACAATCCAATCAAACTCCAAAAAAAGATATATTATTGTCTATCTCCATCTCTTTCCCTTTGTCTCTCCCTCATTGAAAACTTTTATCAT

**>CsSAUR3**

TTCATCACATCTAGCTTTATACTAAACCAGTTTACTTAATGTTTAGTATCTTTCAAAACTTACTAATTTCAACAATCTCGCTCTTATATATTGTTTTATTATTCAAAAACAACTTTTATTAATTTCAAAGTAATGTGTTTTATATTTGTTAAGGATAAACAAGAATACTTTTATCAACATTTTCTTTAATATAAATAAGTCAAGATGAAATAAGTCGATCAATCTATAATAATGACTACCTAATTATGATTTAATATCTTATGAACTTTCGTAACATCACATGTCAGAGTCAAAGTTTATATAGATTAATTGTAATCGACATGGACTTTAATTTTATGATTGAGATGAAATGTTTGATCTTTCTATTTTAACGTCTTCTAGACAACTTTTAATTTAAGATATAAAATGTGTAATATTATTTAATTCATATTTTATAATAGGGAAAATAAAAATAAAAGCATGCTTAATAATGTGACATTAAACTACATGTTTATAATTTAAATAATACAAAAAATATAATATGCCAGCATGAACTACTTGGAAACCCACATGAAAGAACTTATTCGCTTCAATATTTATTTATCAGTTTGATAGTTATTTTGTTTACACATTTTTTATGATTCTAATTTTCATCTTTTTATTAACTATACTTTATACGTACATATAATTTTCAAAACTTTGGAAGGACTTTTGAAATAGGATTAAAAACGCAAACAACAAAACACAAAGAGATAGTCGTTGGTGATAAATAATTTGATGTAGTATTATCACATTAGAACTCGAGTGTTGTAATAAAATAAAACTTTTACTAGAAGAAAAATTATACGATGGAGTTTTGCACAATTTTAACCATTAGAAAAATTATATTACAATAAAGTTTTACACATTGTAATTGTCTCGTAATATTTGTTGGAGGTAGATTCCGTAACATGCATGAATCTTAATTACTATTCAATACAAATTTGAGATATTGTTACATTTTTTTTTCTTTCGTTTTTCGAATAATACATAACTAGCTACTAATTGCATTGTGATGATGCCTTTGGATTAATTTTTTTTGACATTTTTTAATATCTAGGGTAGTAGGCAAGTACAAAAATATATTGCAAGAGTGTATAATAAATTTTTAATTCTTTTAGATGTTAGATTTGACAAATTTAAAAAATTTAACGAGTTGAGCATTTAACGTTTGTGATTTAATTATACTAATTTAATAAGAATCTATTTGATCCAATTCTAAAACTAAGACACTAAAAATTTAATTTAATAAAAAAATCAATCACAACCTTAGTCGTTTATCTTTTCAAGCTTTTAACCACGTTTGATAGGTAATTGAATTTTTATTTTTAAAACAACTCTAAGAATTTTTAAAAAAACATCTAAAACTTTAAAGTTTTTGGTTTTTCAAAATTTTAAATTAGATCATATATTACAAAAAAATCCGTCAACTCTTGTAAAGATATACTCAAAGAAAAAAAAATGAAATTTCTAACCTATTTAACAAAAAATTTAAAATTTTAAAACAATCCTTATGTTAAAATACCATTTGGTTCTTAATTTTTTATTAGTATATCTACCTTTCTATTATGTTGTAAAAGAATTGTGAAACATAAGACATAAACAATAAAAAGTTACTAATGTAAATATAACACTTTAAAAAATCATTTAGTCTTTCTAATTTGAATAGAATATAATAAAATAAACATTAGAGTCTATAAATCGAAATGATTATATGAATTATAGATATGAAGAGATATATTTTTTTTTTTTGATAGAATTTGAAATTTAGAAATATGATATATTATCGTAAAATTGAGATATCAACCAAAAGAAAAAACAAAGAAAGTAAATATGTTGAATATGAAGGGCAATTTGGTACAGTTGTACTTATTAAGGCTCCTTCAAATCCCCAAAACGAAACAACCCCGCAAACGCACTACAAGAAAAAGCGCTTTTATCGTTTCTCCTTTTTGCAATCACCTTCACCGGAGAAAACCAAT

**>CsSAUR4**

TCTAATAATAAATATATATGGCTAGATGGTCAACCCCGAGTTGACCTCTATATTTAGCCAAATAACTCTAATTCTAGACGGTCGATTTTATGGGTTGGTCTCCATACTTAATTAAAAAATAAGTATTCATTTTCTCTTCCACAACATATTATTTATTAAATATTTGATAGACCTAGATCTACTATCCATATGAATTTATAAATACACTTGATTTATGGAAGAAAGAATTTAATAAGGAAAAATACAAATTCATATTATTGAACTCGTTTTCTTTTTATTTTTTCATATTTTTTTAATAAAAAAGTTTTTATGATCTCACATTAAAACCCCACAACCATGAGAATTTAGATGGAATGTTACTTTATATATTTATCAAATGTGTTAAGCCAATCCCAAAATCTTTTTTCTAAAAAAACTATGGCCCCTCTCCCCCTCAAGAACGACTAGGGGCCACTCTCACTCTCAACTCTCACTCTCACTCTCCTTCAAAACAAACCCTTTCAATATGGCCGCCATGTAATGCAACTAACTCTTGATGATAATTATGGTTTTGGTGCAAGATCAAAATCTATCCATTTCTTCACTTTAATAAAAATTCAAAAAGTATAAAATATCAATCTCATGTGATATACGTTGGAATGTCTTTAAATCTGTTGGATGGCTCTTCGAACGACCAAAATAAAATTCATATTTGACTATTTATAGCTATCTACGGTTATGACTCTAGGGCTAAGAGAAGTACTATACTATATAAGTTGATCTCTAACCCTAAAGGCACCACTATTTAGTATTCTTTAACATTCTCTTTAATAAAATTATTCTCTTTGTTTTATGGATGTAGTTAATACGTTACTAATCAACCACATAAATATCAAATGTGTGTGTTGATTCTTCTCTTTTTTATAACTAAACATTATTTGAATAATTGAATCAAATTTCAAAAACAATATAGTAATTAGTTGATGAGTTTTAATTTAAAAATTACTTTCATTTTCGACTTTAAAATCTTGATTAAGTTTTTAGAATAGCTCTCCAAGTTGTGTTTATAAACTTGAAAATTAAATGGGTCAATTCAATTGTTTGATTAGGAAAGACGTAGGCTATGATAAATAACGATTAAGAAAACAAACCTAATTTTCAAGACTAAAAACCAAAATGCTAAATTTAGTTTTGTAGGTCAATATATATACAAGCCACACTGATCCAAGTCTTCAATTATATTACTAACTTTCCCTCTACCTAGCTCTCTTATATATTTGGTTTAATTGATAAAATATTTAAAAAGTTGGCAGTGATAAAAATTATAAAAGAAAACATTTACTCTTGATTAATTTCAAATCGTCTTAATTAGAAACACGTTTAATTAGTTTGATAAGTGTACCAACTCTAAGTAATTTTTTTTTATATCCTCTAATATTTACGAGTGGAGAAAAAAGTGTGAGTGATACAAATATCTTTCACGAGTGTTTTCGAAATTGGAATTCGATAAGAAAATTTTGTATGATAGTAACAATTTAGATCAAAGTTAATTTCTTTCTAGCTAGGTAGCGATTTTTATTTTCCATGTTTGCAATTTTTCATACTTGAAGAAAATTCATGAAAACCATTGATGCAGAGATCAAAATCTATTCTACCTATAGTAACCCCCCCCTCTCTCTCTCAATGGGTCCAAGTTGCCATTGGGATAGCCAACAAACAAAGCAAAAGTAGACCCATTTGGTTTTTGCCCCTCCTCTCTCTCTCTCTCTCTCTCTCTCCCTAAGGACACATTACCCAACACTCTCATGGCTTGTCCTTCACTCCTTTCTCTTCTCCATATATCATTCTCAATCTCACCCCCTTTCTTCTTAAATACCCCTTTCTCCTCCCCTTCATATCCATACCCTCCTTTTTTTTTCTCCTCTAATCTTCAAGCTGCCCTAAGAGGAGATTTATTCCATTCCCTATTTCCCACAGATCTCTTTCAAATTACCACAAAAACACTTATACATTCTTCACAA

**>CsSAUR5**

TCAATGAAGGTCTCTTCTCTGCAGGGAATTGTCAGGCCACCCATTGGATGGTTGAATCCAAACTCCTCCTCGGCCCTTTTCAGTAGGTTTAGAAAAGAAGGATGGTTTAAGAATGAAATTGGAACCACAAATCTCTTTGTTTGGATTTCCCCTACATAGACTGCAATATGTCCTTTGGGAATATCAGATTTAACTTTAGTAGAAACACCCTGTATTTTGAGAATTTGTTTAGTGTGAAGAAGAATTGAAGGCAAACGGATTCCCATCTTTAAATCTTCCTGAAGTGAAAGAAGAGCAAGCAACTGTTGGTCGTTGTGCCCTCAGTGTCGATCTTGAAGTAGAAATCTGCAATACTTGGATGAAATCTCTCTGCAGTTTGAGTTGGAAGATAGTTGTTCATTTAATTTGAATGTATATATAGGTGAGGAAAAGGAGGTGTGAACAAAAGAATTTAGATGTAGGTGTCTGCATGGTCCTACATCATCAAGGCTCACATGGGGTTGCAAAATTGGTTGAAACAGACTCGTTTTCTCATCTGGCCCAACTTCTGATGGTACACATTCACTTGTTTCAGTGGGAAGATATTAAAGAAGAAAACAAGTGGTCAAGGAAAACCAAAAATCAAATGCTGTGACCAGGAAAATAAATAGACCAAACGTATTCAATGTAATGAGAAACTTCCAATCACAAGTTGCTGTATTGTTTGCCCAATGCAATAGTAACTAAGAAATAAGACAACCACAAAGTACACTTCATATTGTACACAAGGTACTCACAATTGGACAATAAATTGCTTTGGCTCTTGTATAGGCACGTAGGTAGGTTCCTCCTTGTTAACTTCTTCTTTAGTGCTATTATGGTAACTTTTCTGGTTTGTTGTATCCATCAATGCCCCCGAGTAACAGGAAACTGAATTTTTCCACATGATCTACAAACACTGTCTTCATCCTCAAGAGTTATTGGAGTAAGCCTGCAGCATGTTTTATGTTTTCATATTGTTTAGTTAGTCCAGAACTTAAGAATGATAATCTTTTGAAAAGTACTAGTTTATGAAATGGGTAACCTATATTTTTAGTTATCAACTAAAAGTTACTTATTTTTTCCTTATAAGGATCTCATTAAATGTTCCGAACTCCACATCCACTCATTGTGAGAGAACGTTGTTGGTCAGCTTTCATGTGAACTTTCACATAGGCTAGTTTGGAATCCAAAATGAAAAATTTAAGAAAAAAAGGCTAACCTCTCAATCTGCATCAGGTAAATTCTCTCCATATCTAGCTATTTTATAACCATCTTAAGTCTCATCATATTTTTGGCTTGTTGATTGGTTTGTGAGACTCCAAGAGCTGTAATAATGTCATCAGAGGTTAGCTGCCAACTTTGGTTTTGTTCATATCTTTGAGTCACTTGGTTTAGATTTTTTAATTAGATAGGCTTTCCTTCTGTTTAGACATAAAAATGTATTCCATTAAATGAATGTTTCCCTGCTATAAGTTGGCATGTCAAAGAATTAGGGGAACGGTTTGGATGTCTGATGCATTTCTCTAACAAATGTAAATGACCGATTCCTTGTGCAACCTGCTTAACAAAACTAATGAGTTTTCACTAAGTCACACTTTTCAGCTCAGATGGGTCCTCTATTGGCTGGGTTTCCCAAGTAGTTAGGTGACTTTGTTTTCCCGATATTATTTTCCAAGTAGTTGAAGATTATTATCTAGAGTACACTACCATGTGATACAGTATACTTCACAAGTACATGTCTCCTAATTGAGCCCATTCATAGATGTCTCACCTGCCACCACTCATTTCTTAGATCAAGCTAGTGGCCACGTCTGCTTTTTAATCTATATATACATGCTTATTCAATTTCCAAGCTACTCAAATCTCAAACTTCCAATTCACCACCAAATATTTTCCTCTGTTTCTCTCAAGCGATATTGTGCAACTTCTAGATTCTAAAATTCAAGACTCCAACTTCTTCTTGAGAGGGAAAAAAAACC

**>CsSAUR6**

TCCATGAATGTATCATTGCTGCAAGGAATAGTGAGACCTCCCATGGGATGATTGTAACCAAATTCTTCTCCAACTTGACTTAGCAGGTCCTTGAAAAATGGCTCGTTCAAGTAAGCGATAGGAATCACAAACCGCTTCTTCTGGTTTTCCCCTACATAGACTGCACAATAACCTTTGCGGATTGCCGACGATCCATGATGTGTTGTTAGGGAAGAAAGTCTAATATTTTGCATGACATTTACCATTCTTCCCAAACGGAAACCCATGGTTTTTTTTCCCTCTCAAGAAGAAGTTGGAGTCTTGAATTTTAGAATCTAGAAGTTGCACAATATCGCTTGAGAGAAACAGAGGAAAATATTTGGTGGTGAATTGGAAGTTTGAGATTTGAGTAGCTTGGAAATTGAATAAGCATGTATATATAGATTAAAAAGCAGACGTGGCCACTAGCTTGATCTAAGAAATGAGTGGTGGCAGGTGAGACATCTATGAATGGGCTCAATTAGGAGACATGTACTTGTGAAGTATACTGTATCACATGGTAGTGTACTCTAGATAATAATCTTCAACTACTTGGAAAATAATATCGGGAAAACAAAGTCACCTAACTACTTGGGAAACCCAGCCAATAGAGGACCCATCTGAGCTGAAAAGTGTGACTTAGTGAAAACTCATTAGTTTTGTTAAGCAGGTTGCACAAGGAATCGGTCATTTACATTTGTTAGAGAAATGCATCAGACATCCAAACCGTTCCCCTAATTCTTTGACATGCCAACTTATAGCAGGGAAACATTCATTTAATGGAATACATTTTTATGTCTAAACAGAAGGAAAGCCTATCTAATTAAAAAATCTAAACCAAGTGACTCAAAGATATGAACAAAACCAAAGTTGGCAGCTAACCTCTGATGACATTATTACAGCTCTTGGAGTCTCACAAACCAATCAACAAGCCAAAAATATGATGAGACTTAAGATGGTTATAAAATAGCTAGATATGGAGAGAATTTACCTGATGCAGATTGAGAGGTTAGCCTTTTTTTCTTAAATTTTTCATTTTGGATTCCAAACTAGCCTATGTGAAAGTTCACATGAAAGCTGACCAACAACGTTCTCTCACAATGAGTGGATGTGGAGTTCGGAACATTTAATGAGATCCTTATAAGGAAAAAATAAGTAACTTTTAGTTGATAACTAAAAATATAGGTTACCCATTTCATAAACTAGTACTTTTCAAAAGATTATCATTCTTAAGTTCTGGACTAACTAAACAATATGAAAACATAAAACATGCTGCAGGCTTACTCCAATAACTCTTGAGGATGAAGACAGTGTTTGTAGATCATGTGGAAAAATTCAGTTTCCTGTTACTCGGGGGCATTGATGGATACAACAAACCAGAAAAGTTACCATAATAGCACTAAAGAAGAAGTTAACAAGGAGGAACCTACCTACGTGCCTATACAAGAGCCAAAGCAATTTATTGTCCAATTGTGAGTACCTTGTGTACAATATGAAGTGTACTTTGTGGTTGTCTTATTTCTTAGTTACTATTGCATTGGGCAAACAATACAGCAACTTGTGATTGGAAGTTTCTCATTACATTGAATACGTTTGGTCTATTTATTTTCCTGGTCACAGCATTTGATTTTTGGTTTTCCTTGACCACTTGTTTTCTTCTTTAATATCTTCCCACTGAAACAAGTGAATGTGTACCATCAGAAGTTGGGCCAGATGAGAAAACGAGTCTGTTTCAACCAATTTTGCAACCCCATGTGAGCCTTGATGATGTAGGACCATGCAGACACCTACATCTAAATTCTTTTGTTCACACCTCCTTTTCCTCACCTATATATACATTCAAATTAAATGAACAACTATCTTCCAACTCAAACTGCAGAGAGATTTCATCCAAGTATTGCAGATTTCTACTTCAAGATCGACACTGAGGGCACAACGACCAACAGTTGCTTGCTCTTCTTTCACTTCAGGAAGATTTAAAG

**>CsSAUR7**

CCAAAGCTTTGTTCGAAACTGTGGATTGGAAGACTTTATTGAGTTCCTCTGAGCAAAGGTTTGCATTGGCAAGATAGTCTTGAAGTAGCTGGTTGCCTCTTCTTGGTGTTCGCTTTCTCCTTTCATTTTGAAGATTTTGGTGTGCAAGACTTCAGTTTCAAGTGGGCTCTTTTTGAGAAAGCGTTTAAGTTTTTCTTTGTATCTAGCTTTCTTCGAATTTCGTTTGCTTTGATTAACTCATTGTTTTTATGCTTGAGATTGTTATTTTTGTTCTAGCTAGTTATTTTTCCGTAGTCTTTTTTGTTCTTAGTATAATACTCTAGCATTAGTCTCTATTATTATTATTAATAAAGAGGGTCGTTTCCGTTTAAAGAAAAAGAAAGTGAGGATTAGCGAAGGAATATCAATATAGGAGTTCGAGGGAACCTACTTGATTAAAGGATCACATGGGGTTGCCAAACTGGTTGTAACATACTCGTTTTTCTCAGCCCAACTTCTGATCGGATAAGCCCTGCTTGGTTCAATTGAAGGACGGACATTAGAGTTGACCAAGTGTTCAAGGAGAACCGAAAACCAAATACTGTGACCGAGAAAATAGACCAAAGGAAATAACTACCATGTGATGAGATACCTCCAAACACAGGTTAATGCATAATTGATTCAGTGAAATAACTACCAAAAAAACTGAATTTCGGTTCGGGCATAATGTACTCGACAACGTACAACAAATTGTTCTAGGTCGTAAAGGCATTTCAAATCCTCATTGATTTTTCCATTAGTAACATTATCACCGCTTTACTGGTTCGTATCTATTGATGCTCCACGAGACAGGAAGTTATGTCTTCTACACAAACTGTCTACCATAAAGCAACAATTTATCTTTGGTTTATCAAGACTGTACCAAACCCTGAGAGCTAAACCTCGAGAGTTATCTAGCACTATGTTGATGTAATTATATTGTTAAGTTAGTCCAGAGCTGGTGAATGGTATGGTATTCTTTCAAAAAGCATTAGTCTTGTAAATTAGTAGTCTGCATCTTAGTTAATTGTTGAAGAAAAGATGTTTCATTCCTACGTAGATGCTTAATTGATTCTAAAAGTATCTTGTATAATATGCCAATAGGCAATATGGGCTCATTTTGAGAAAACAATGTAGGTCACCTTTCACGTGAACTTCACAGACTTGTTTGGAACACACTATAAATAAACAAACAAAAAAAAAAAAAGAAGGGGAAAAAAGGAGGTAGGATCTCACAATTTGGATCAAGAACAGTTGATTTTTCTCTATTTATGACTGTGATCTCACCTTATTGTCTCATTTAGGTTCATCATTTGTGGGACTCCAATAGCTGTACTGATATATATATATATTATCACAAAGGCTTAGCTGCCAAGTTTGGTCTTGTCTATTTTTACCCAGATGGTTTAGGTTCTTAAATTAGATTGATATTCAATACATTCAGTCAGAAAAAATCTATGCCATTAAATGGCGTGTTCATGTTGTATGTTGACTCGGTAAAAAATAAGGGGAAGCGTTTAGATGTTTTAGGCATGTCTCTAACAAAAGTGATCAACTACTTGCTTATGCTCTGTGCTTAGCAAGATTAAAAAGTTTTTAACTGGCCACAATTTTCAGCTCAAATGGGTGGCTGATTTTCCCAAAGAGTTAGGTGACTTTGTTTACCCAATACTATATCCCGAGTATCTGAAGATTATGGTCTAGGAGACAGGACCATGTGAAACAGTAGACATCACTAGCAACTGCTTTCTAGTAGGACCCATCCAGAGATTTCTATCCACCTATTTCTTAGAGAACTATTGGCTACATCATCACTTTTCTTCTATATATACATAATTACATACTTGTTTCAAGTCCCAAGCAACTCGGATCTCAAAAATTCAGCCACCCGCCAAGCTTTCTCATTCTCTCAATTGCAACTGTAGGACTTCAAGTTTCTTCAGTTCTTAATTTATTGATTTCTTGTTCTCAATTTCAAAACC

**>CsSAUR8**

AAAAAAATATAATAAAATTTTATATTTTATCACTAAAAGACACATATAGACACATTGATAGACTTTTATCAATATCTATCGATGTACTTATAAATTTTGATATAATTCTTTCAATATCTATCACTATTTACTATTTTGTAAACATTTTCATCAGTACTATTGTTTACAATAATTTTAAAACTAAACTTGTAAGAAGAACCTTTCTTTTCAAATTATTTTTAGGTATTTGAAAATACAATTCAAACAAACTTGATTCATATGAACGTTAGAATTCAAACAACTATCTTCTAGTGCAAAATTATGGTACCATAGCTAATTAAAGTATAGGTAAGCAAATTTATTTTAATATTATTTGATGATAGCTAGATAAAATAATATTTTAAAATGTCACATCCACATTACATTATTCAAACCAATATTTCCTTTAATTAAATAAAAGATTTAAAAACTTCAAAAATCAAATTAAAATATTTGTTTAATCCTTCATCTCCAAATTGGGCAGTTGGAGCCATGTGATTTGTTCACATTCAAAGTATATGCTTTAGCATTACTAATTAATAACATTACCATTAATACTTTGTGTCAGTCTTAAAATTAAAATATTCCCATTAACTAATCATTATTAGTAGTAAAAAAAAAACTGATAATTTCCCAAAATACTCATAATTATTAACTATATAGGTTTATTAGCATACCATGTGCAATAATCTATATGCTTTACCATTTTTAAAAAGATACCTGGCAAGTATTATCTATAAAATATGACACAATATCAATAATCTCTAATTAGTTTCATTTTAATATTTAACGTTCATTGCACTTCAAGACAAAACACGACTGAGTCTAAAACGTAATCATACTTCTCAACCTAACTTGATATTTTATATTTTAAACTAACTCGAGTTGACAGACAATACGTTTTTGCTAAAAAAATATTTAAATATGGGATTGACAGCTTATTCTAATACATAATACGTCGTATCAAAACGCAATTATACTTTTCAACCTAGTGCATAGTCGGTGCCTTTGATTTGGAAAATTATTCAACGGAGTTGACAAATGTATTTTAATACAATATAGTGTGTTGTATCACAATATAATTAATTAGACTTTCCAAAGCTAACTTGATTTTTGAAATTAAATTGAGAATACATTTGGCTTGGATCGTTATTCAAGGGCATAATTATACTTCTCATTCTCACATGACTTCTTAAACTAACTTTTGATAATGGACACTGCATTTATTCAATGGACTTGACTTATTTTAGCATCCTAATCATACCATAATGTAATTATCATTTGATTTTGGAAGCTAAATTGGAAAATTTATTTAATATGTATTATTAGACATGTTATTCTAGTACATACATGTGTTATACCAAGATGTAATCATACTTCTTAACTCACCTCATTTTTTAAAAACTAGTATGTGTTCACTAACAATGTATTTGATTCATATTATTCTAGGAGCTTGACAATGTCTCACATCATAGTATATGTCGTACCAAAATATAATCACACTTCTCAACCTAACTATATTTTTGTATCTAACTCAAGTTCAATCACGAGGATGCATTTCATTCAAAAAATGTCATTCATTAGAGTTGACAACATAAGCTATCATACACAGTGTATGTACATAGAAAACGGTAATTATGTTTTCAACCTCACTTAACTTTTGGATCTAACTCGAGCTCATCAACAATAATATTTGATTCAAAATATTATTTTATTAAAGTTGACAGACTATCGTAACGCGGTGTGTCGTATCAAACTTCAAAATCACTTTAATAATGAATAATGAAGTGGATATAGTACAATCATATATTAAATTAACTTCCTCAAGGACCCATCAATAGCCAACTACCATTCTACAACCAATCCCTTCCTATAAATTCAAAACCCTAATCTCCCCAATTCCCAAATCACATTTTCCTTCTTTTTTTTTCATTTTCCTCTTCAATCAGTTTCATCCATTTCTCCATTTTTTTTTCTCCTCA

**>CsSAUR9**

TAAAAAAATAGATTTGGAAAGATTTATATACTTCTTTCAAAACTTAATATATTACTCTTAATCTTCTTAATATTTAGAACTATCCGTTAATTTTGACGGTGTGATATTCTCAAGACTCTCAACTATAATTGTATGTACGTGTCTCATTATTTATGGCCTAAACTTATGTTGTCCACTAAAACGATCCTATACTGTATAATACTTTCGAAATGTTTATGAATAGTGCCTTCAAAGAACCAATTAAATTTTTCTAAATGTACAGTAAGAGCAAGAACATTAGAACTCCAAGTGCTTTATATATTAAAAAGACCTTTCCAACTGTGGTACATTTAAACCCAACTGCCTTCTTGAATAAATATAAAAAGTTATAATTCTATACCATACTCATTCATAGTTGAATGTTATGGTACAGTTTTGAGGTACAGTAGCATATTTTTAGGCCTGTTTTCAATAGCACAACTCCATTTTTAACCATTCGCCATTGTATTACTGGGTCCTCTTGTCTAAAGCAGAAACCTTCTCACATGGAGATGTTGCTCCTTATTTAAAGGATTGGATAGACATGGTGACCGATTGCCGACTAAATATTAAGTTCTTGTTTGGTAAACAATTTGATTTTTAGGTTCTTGTTTCTGAAGTTTGAGTCTACAAACACTACTTTGGTTTATTAGTTGTAGGCTTAGTTTTCAAAAACAAAAAATGAAATGGTTACCAAACTGTCTATCATTGTTATGTGATAAAACGTGGTTTCATTTGGCTTTGGCCATGCGTCTATAAGTTTTTGAGAGTGTCTTTGCAGCACTACGTTTAGTTTTTAAGTCTTAACTAATGTTAGAGTTGAAAAATCGGTGTTTAGGATATAGAGTTACGTATCTGGAGTTTGAAAATAGGAAACATTAGAGAGGAGAAGATTAAGTTTATCGATAAATGTACAAAGTTTAATAATGAGCACTATTAAAGTTGGTGAAGGTGAATTATTTAAGGACGACTTTATAAAGTTGATAAGTGAAACATCCCTTGAGAGAGAGGTTAACGCTTTGCTCACCGGCACAGTCGCTGCTGGCAAAGCAGAGGTGAAAACCACCCTCACCAAGGGAAGGAGACTAGATTTCAGAGTGGTGGTAAAACACTTGATAGGGAGATGGATGACCAAAGGAGAATTGAGGTTGTGCACAAGAAACGGAAGGCGAGGAGGAGAAAGCAAGCGGATGAGAGTAAAAAAGAAGTTTGAAAATAGTTAGAAAAACTTGTTTTGAAAAACCATATCAAACTTTAGTAGACATAACTTTTAGATCAGTCTGTCAAAATAGTCATCTCCTACGCGATTTGATTCATGAAGTAACTAAACGAAACGAAACTTCGACACACTACTGTTTTAAGAGACCCACGAACCACAATGTATAATAAGATTCAATCCATCTCATAGTAATTAAAGCTGTGAAAATCCAGCTATGCCAACTACTCGAATAGACACAACTACATACTCAACTCCCTTAGACAGTGAAAAGCATAACTCATCCTTGTTCCAATGTCTGTCTTAAAAACGCAACCAGCTGGTCTTTTTGAAGGACTAAGTCGTACCAAAACATGATATAATTTTCGATTTGAGGAATACATTATCCATCCCTTCCTTCATTCAACCTGAAAATTAGGTTCAATGATGTAACCATTGGCACTTTCCCAGAAAGTCAATCACGTGGATATGCAATTTCCTTGTACAGTCTTTGTTTTGAATGTAGAGTTCTTTTATATGTTGATCAAAGCCATGTGAAAGGGTCTTCCCAACAACAGTCTTTAATACCCCACATCTATTAGTAATGCAGCAAGAACAGATCAGCAATCAGAAAAACAGCTATTATTTTTTCCTTTTTTTTTTTAAAAAAGTCTATATATACAAACTTCAAACGAGGCTTCAAACATATCCATCAACCAAAGAAAGTTTTTAGAGGGTGTTCGCCTCCTCTTTGTACGTGTTCTTCAGCCGATAACACAATTTAGCA

**>CsSAUR10**

GTTCCCAAAGGTCACATTCCAGTGTACGTGGGAGAAACCGACAGAAAGCGGTTTTTCGTTCCGATATCGTACTTGAGTCATCCTTCATTCGTAGAGTTGCTCAACAAGGCTGAAGAAGAGTTTGGATTCAGCCACCCAACTGGAGGTTTGAGAATTCCCTGCAAGGAAGAAGCCTTCATTGATGTTACTTCTAAATTGCAAAGTTCATGAATGATTTGAGAACAAGGAGCAATTCTCCTCACAGTTTTGACAATTTTTTTCTCTGTTCATTTTAGTGTACTACTTCGTATGTAAATGGTTAGATTAGAAAAACTGGAGAGTTACAAAGCCATTTGTACATCAATGACAAATATCAGTTGCAATGAAACATTTCTTGCTAACAAAGCCTTATTCATTACTAATTGTCTTCTGATTCTTTTCTGAGATAAAATCTACTGGAAGTTATATTTGATCTGTATGCAATTAAGTCCTTATACACATGTGAACATTCCCTTTGTATTTATTCATATTGAACCAAGTAAAAGTTTTACCTTAGAATCAGATGCTGTTTTTGGGTGTCTTTTAGAAGAAAATGGCTTTGCAGCTGCGACCGCAAATCTAACATACTTTTACGTGAAATAGTGAACTGTCATGAAAGAGTTTAGCTCAACTAGTATTTGTATATACTTGAAAATCAAAAGATCTCGAGTTAAAATTCTCACTTCTGTTTGTACTTCGAAAAAAACGATCTTGATTTGCATTAATGAAGTGCTCAGTGATCGAATGCTATGACAAAAGTCCTACGATATGGAGCTACGTGGACCCACGGCAAGGGTCAGGAAGTGCCCGAGCTGAAGAGTGTATCATCTTCTATGAAGCAAATCTCGTTCCAGTTTGTAGGCTTCACTGTTAATCAATGCTCAATGTATTAGGCAAGGACCAAGGAATTTTAATAATTAGTATTAACATTTAATTATATAGACATAATTTGATCTAAATCATCGGTTAACCCAAAAAGCTTTTGTTGATAGCATAACTATCAAATTAATAAATTGACACACAGATTTACGTGGTTCACTGACAAAGTATTAGCTATATCCACGAGGTAAAGGGAAAACATTTTTACTAGAGAGAAATTCATAATACAAAATAAAGGCACCTATATAAGATGGAAGAGTTTACTGATTTGCGTGGTTCATTAACTATATCCACGAGATAAAGGGAAAACATTTTTACTAGAGAGAAATTCATAATACAAAATAGAAGCGCCTCTGCTCTAAACTCTAGGACAAAAAATTAAATGTAAATAACTTAGACATTTTAATGTATACTTGGTTATTCAAAGTGTCAGATAACAAGTTCAAGTCATTCCACCACTACTTCAATGATAGCACTAATTGGCTCCTCCATACAGGTTGCCAAACATGGATATTTTGTACTTCTGTTTTCAATTTGATACATTCTATCTTGATTCATTTAATCTGCTGCTACAACTTTACTTGCACAATGTTGTTTGTGATGAATGGAACAACTAGGCCAGCAGAACAGAATCTTGTGATACAAACAAATATAAACCACAGAATATGTGTGGAGTGTAGAATAGACAAAGCAATGTTTTTTTTTCTTTAGAAAAAGACGAAACTTTTCATTAAATGAATGTAAATAGACTAATGCTCTGAGATACAAAAGTATACTACAAAATATAGCGTGGCTTAAGACATAAACTGAAGGACAAAGTCATGTGGAAGGGTCCACTCAAATGTCTTCCTTAGCTTTATTTGGATAAATTTTTATTAGTTTGGCAAAACCATGTGGAAGGTTTCACTCAAATACCCAACCACCAAACTCCAACACCTTACTGATAAGCACCATTCACCTGCTAGAGCTTTCTTCAAAACTTTATAAATATTAACATCATATGAGAATTCAAATACCATTTTCACTTGACTTTCTCTTCTAGTTGGTTTGGAACCAATCCAATCGAAATCTAAGATATTTTCATAGTCTTTCATCTTAAACAA

**>CsSAUR11**

CATCATATGAGAATTCAAATACCATTTTCACTTGACTTTCTCTTCTAGTTGGTTTGGAACCAATCCAATCGAAATCTAAGATATTTTCATAGTCTTTCATCTTAAACAAATGGGGATTCGCTTGCTATCTTTGGTTCCCCATGCCAAGCAAATCCTGAAGATTCAGTCAGGACTTACCAAAAACCAGTTGGATGTTCCAAAAGGGCATGTAGCAGTTTATGTGGGAGAGATTCAAAGGAAACGGTTCGTGGTTCCAATATCTTACTTAAACCATCCGTCATTCAAACAACTGCTCTGCCACGCAGAAGAAGAGTTCGGTTTCCATCATCCTCAAGGGGGCCTAACAATTCCTTGCAAAGAAGATGCCTTTACTGAAATCACTTCTAAATTGCAAGCATCTTGAAGGATGAAGAAAAAATGGCAATATTTCATTTTTTGACCATTTGTTTTTCAAAATTTTCTCTATTCACTTGCCTTTTGACTTGCAAGAGAATATACTCCATTGTTAGCCATAGCCTATAAATGAGTAGCATTGTAAAGTACTGTTTACCAACAAAAGTAGATGTATGCACATACAGCAACAATTCAGAATCAATTCCCATGTAGCTTATTCTTGTCTATACTCTATAGATCATCAAAATTTTACTTTCATCACATAAGATCTGTCTTTCTTTCATATTGTTGAGAAAGTGAAATAGTAGAAAAATAAAGATGCCAATGAGACTAGAGGAGGATATCAGAAATCAGATCATAAGATATAGAGTTGTCAATGGAGTTATAGGGTTCATATATAACACTACATTAAACCCTAGGCCCAATGCTTATAAAACTCAACAAATAACTTATTCAAGTCATTGTAACATATATTTTAACAACTTGAAAACAAGAGCCTTTGAAGGCTTTCTACGAATGGGAAAAATTAATAGGCAATTAATAGAGATCGATGAGGTACTTGATATCTTCTACATTGGCTTCTAAGCTGAAAAGATGCCAATCTTGCTGGAATTGTTTGGGCTTCATATAGAACAAACACATAATTGTTATGAAGAAGAAGCGTTTTAGTCTGCTAAGCTAAGCTAAGGTGACTAGAACATAGAATTCTATAAGCCAACTGAAAGTCTCTAAAACCACACATGGATTTGGCTAATGGGTCACTGGTGCTGGGTCCTTCTGCGCTTCTTTTGATGCCTTATGTGAAGTAATTCTAATGAGCAGTGACTCTTGGATTGAGATGAGATGAGTGGAGCTTTTGTATGTTCCATTTTGCCCGGCACCTGGGTTGAATTCATATTTGCTATATCTGTGTTGTTTGTTATTTTGATCTTAGGATTTAGAGGAGTGCATTATATAAACTCTCTAACTCCTAGAGGCACATCACATTTGTATTATAAGTTTTCTCTCTAATAAAATTGTTGTTCCATCTGCCCGTGGAAGCAACAAATACAGAACTAAGTAAATTTGTGTGTCAGATTCTCTATTGCTTTACGTTTGTTGTTTTCTTTATTTGTCAATTCCATAACAAATTAAACCAACTAAATGACAAAACTTTCAGATTATAAACATCCTTCAGACATATATGTCATTTTCCGCTGATGATGCAATATCCATCCTGATATACACCACATATTTTTGAAGTGTTCAATAGTTCAACTTAATTCCAGCTCAATTGTCCTATACTCAATACCTACTAATATTTTTCAGCCAGATGAAAATGTTTGAATATGACTTCATTTATTGGGACAAGTAGGTCCAGAGTAGCAGAATTTGGATCAAATTTGAAATTGAGTGTACTTCAATCACTCAAACAACCCATGTGAATATTTGATATAGCAGGTTGTCCTAGACACCTATCTTCATTCTCTGTTCATATCTCCTCCTATATATACATATCAAATATGCAACTTATCTCGAACTCAAATCTTCTCACACATTCTCATTCAAGCAGTTCAATTTACCATTCAAAGTTCTTCATTGATCCCTCAATTCAATCCAATTTAAAC

**>CsSAUR12**

AGGATTTGGGTTTGAGGGAAAGCTTTCTTTTTCCAATTTTAACAAGAAATGAGACTTTTCATAAAGTAAACAAATAGATTTTATTAAATGGGAATTTTGTTATTAGAAACTGGAGATTTGGCATTGTTTGAGACATCAGACATTGGTGAATTGCTAAATATTGCACCCACAACAATGAATACAATATTTTCTTAAAAGATTGCAAAACACTTCAAGAAAATAGTGTTTCAATATTCTTTATGACTAGCAAGCTACAATGAACTTATCTTCATTGATGAAGAGTGATGGAGTACTCAATATCTTCTCCAAAAGTAGCAATCTTGCAGTGATCACTTAGCTTTCAAATGGAACAGAGGGACAAATCTTGTTCCAATGCAAAAATTTCAATCCACCAAAGTGAATATGAAACATTGCTTTATTCTCTAAGCACAACTTTAATAAGCAGTTAACAACCTCCTAAATCCAAAGATGGACCAATGGCCTCTGAGTCTATCTTTTTAGGAGATTGCACTGTTGATTGTTTGGTCATCAACTTAACGTTTTGAGTTGATAGGTGATTTAACGTAATATCATAGTAGAAGGCTTTGTCTTTGAACCCGATAAATGTCATTTCTTTTCACTAAACATTGATTTTTACTTATTGGATCTTCTATAAATTTTCAATCTCTCATGCGAGATGGGAAGACTTTTTTAGTATATAGAAAGAGTGTTAAAGTGTTAATATAATTAAATTTATCATCATTTACCAATTTAAGCTTTTGGGTCAATTAGTGATTGATCATATCAAAGTATTTTATTTATAAAAAACATTAACATATTAAAGTGTTGATATATAATATTAAATGTATCTTTATCGATCAACTTAAACTTTTGAGTCAATTGGTAATTTAACATGGTATTAAAGTAGGAGGTCCTATAGATCAATTGACGCCAATTGAATGCATCTATTTAGGGTGGAGAAGGGTTTTGCACAAATTGGTGCAACAATTCTTCCATTGTATGTTGTGAGATGCATGTATAAAAAAACAATAAGACAATAACTAATCCATATGTTTACATTTAAAGAAAAACAATTTCATGTGATATAAGTTTAAAGAAGTTAATCTAATGAAAGTGGCTATTACACTATGAACTGATATACTATTGATATATAAATGATATACTTGAAATGCATTTTGAATTAGTATTGATATGCAATTGAAGTATCAATGCTACACTTGAAGTATATTATTCGAATGATATATTTCATATTTGATACATGATAACGTTTTTTATATGTATTTTTCTCTCTATAAATTTGGTATTGATATACAAAATATGAATATGAAGTATATTCATTATACAATCGACATATCAAATTTATTGTAACTTATATGCTATTAAAACATTTAAAAGTTTAAAAAATAAAACAAATTATATTATTATTTGGAGTGCTTGTCCTGGACTCGGATTCGAAACCCCTATATTTGGTTATTTGAAACGTCAAATACACATGGTCAAGGTATTCTAACAAAGTACAAAAATGAATGGGTCATCCATTGCAAGGCAATCCAGTCACACTAATATTCTTTTTATCCAATTTTATTGTTCTGTTCACTCCATCTTGAGCCATTTAATTTGGTGTAACAACTAAGGTGCTAGGACAGTGGCTTGTGATGAATGGAACAACTTGGGCAACAAAGCAAGAAAAATTTCTACAAACAAATATAAACCATATAATATCTGTGGAGAGAGGAAGATATAATAGAATCAGCTTTGATTAGTTAGGCAAAGCCATGTGGAAGGGTCTACTCAAACACCCAACCACCAAACTCCAATACCCCACTTAGCAACTGGATAAGCACCAATAACTTTCATAGCTCTTTCATCATGACTCTATAAATATAAACATACTATGAGGCTCAAACAACCAAATTCAATTGTTAACTTCCTCTGCTACTTGGTTCTGTACCAATCTGATCAAAACTTAAAATATTTCTCAACCTTTCATCTTAACACAA

**>CsSAUR13**

CAATCAAATTTAGACATGGGAATTCGTTTGCCTTCAGTCCTCCTCAGCGCCAAGCAAATTCTCAAGATGAAAACTGTTTCTACGAGATGTCAGTCTGATGTTCCCAAAGGCCATATTCCGGTATATGTTGGCGAAAACCAAAGAAAGAGGTTTTTGGTTCCCATTTCTTACTTGAATCATCCTTCATTTGTGAATTTGCTTAGTAGAGCTGAAGAAGAGTTTGGATTCAGCCATCCAACAGGAGGTTTGACCATTCCATGCAAAGAAGAAGCCTTCATTGATGTCACTTCGAGATTGCATATTTCATGAAGGATTGAGAACACAGAGAATATACTCTCGACAGTTTTGACAACTTTTCTTTTCATTTTTTGTCTATAGGAGATAGAGTTGAATATCACTGTAAAGGGAACTTCAATGTGTAAAGTGACACACATGAGTTGCAATGGAGTTGAATTCTTTTGTCCACAAAAACTTGTGTTTCTTCTCTTGCGTTAAGTTTTTCTGATAACGTAATCAAGGTTACAACATGTTTTCTTGAAATGTGGAGCACCAATTTTCATATGTTAAGTTATGAGTTGTTAACATTACAAGATTTAAGTAAACTTTTCGAAAACATTCGAATAAATCTCTCCGAAAATCTCTGATGCAGTAAAGTAAAACAATGACAAACATCGGCTACAATGGAAGTAAAGTCTTATTTTTGCATTCAAAACCTTAGTGATGAATTATCATATCAACTTCCGAGATAGAACTTAACAATATTATTACACAAATTCGAGTTGTGATCCTGCTCATGAGTAATGAAGTGCATGTTTGAAAGTGATTTAAAAATTTTCTAGCCACATATGAGAGGAATGTTAGAACGTTGATATAAATATTAAATTTATCCATGATCCATTTGCTTAAACTTTTAAGTCACTCAATAATTTAATACAAATACATTTGGTGCACTTTTGAGGTCTATGCAATGTAACTGACCTCCTCACTGCAATTCGCATTGATTAAAATCTCATTCCAACTCGCATGGTTCACGGCACTCAATCTACTAGACAAGGACTGCTAAATCTGTTAATGAGAGCAAACATATTCTTTAATATATAATTTGATGCTTATGCCTTTTTTGGTTATTTCTCAACCGAACATCAAATTTGTTACAAAGTTGATAAATAAAGAAAGGGAGAAACATAGACAACAAAGATCAACATGAAGATTTACGTGGGTCAATAAGTGTGTTAACTACGTCCATAGATAAAAGAAGAGATCAATTTATTATTAACAAGAAATATAAGATAACAAAAATTGTAGAGTCGTCTCTAATATTAGAGGGTTTATATCTATCACCTTTTTTAAATCTTAGGGCTAAACACATAAATAACAAAATGCATATACAACTTAGGTTTCGGTTTGTTGTCCTTGACATCCTATAAGAGCGTGTCATCCTTGAATCCTGACGTGAGATCCTTGTTCATGGACACTTTAAGCATCAGATAACAAATCGGAGACATTTTAGCAAATTACAACCATTAATGGGGTCATCCATTCCAATTCAGATACAAGAATATTCTATTCTTCCATTTTCATTGTCTTTCACTCTATATTGAATCATTTAATTTGGTGTAACAACTAAGGTGCTTGGACAGTGGCTTGTGATGAATGGAACAATTTGGCCAACAAAACAAGAAAATCTTATATAAAAACAAACATAAGCCATAGAATATCTGCGGAGAGAGAAGGATAATAGGATCACAGGATCGGTTTTGATTAGTTAGGCAAAGCCATGTGGAAGTGTCCACTCAAATACCCAACCACCAAACTCCAATACCACACTTTGAAATTAGATAAGCACCAATGCTTTCAAAGCTCTTTCTTCATATCTCTATAAATATAAACATACTATGAGGCTGAAACACCAAATTCAACTGTTAACTTCCTCTTCTACTTGCTTCTGTACCAATCTGAACAAAACTTCAAATCTTTCTTAGTCTTTCATCATAACACAA

**>CsSAUR14**

CTTTTTCTGTCATTTTCACAGTGGATATAAATTAAATTTATGACTGGCATTGGGATTTTAATGAACAAAGAACCTCAACTTTTCTTTCTTCAGATACCAGAGCTTACCTTTGACATCACCCGATCCCCAGGTAGACGCATCTTTTCAGCTCCATGGTCACTTATCTTGTTTATTGCGAGCATAAGCCGAACATCCAAATTCTGTCAGATATAATATATCACGTTTTACACCAGACAACATATCTTGTGAAGTTTTTGAACCTACGGATGGGAATGGACAAGAAGATATTAGATAACAAATTGGTCATTTTCATATGCGATCAGTAGTTTGCTCTTGTTTCTTCTTAATAAGCAACTGCATTGAATTATGTTCCGTTCCTTTGAGAATTGTATAATATAATATCAAGATCACATTCCTTTTTAGATAGAAAATTATTTTGATGCTTCATCTAGAATCCTACAATCATTTGCCACAACTTAATCTTGCCTTTTTGTCACTGAATTTGTTGAAATCAAGCTCTTGCGATAAAGATTTCCTAGTAGTGTTTGCTACTATTTAAGACTTGAGAATGCTCAGTAAACATGTCCTCCATACTGATCGTTAGGAGCTCAAAATGAAAACCATCCAACTTCAGTTTTTTCTCAATTCAAGAATGTCTACCTATCTCGCATACAGTTTTTCGGCTTTATCCATCAACTATGAATACTGACCTCTGAACAAATTGACAAAATCTCAGAAAAAGCATATGTAACTTTTCAGCGTTAAAGCAAATAAAACAGTAAGCCGATCTCTGTTTCCCTGGACCCAAGTAATTGTGTCAACCTTCAAGAGCAGGTCACTGTCAAAATCAACCTTAATCCATGGTAGAATCATGCTCCACTTTCAAGTCTGTTGTTCCTAGTTTGAAACTTTGAGATTCCTGCTATGTAAATTTCTAAATCATTCCATATCTTGGCTTAATTCCTGAATGAGCGACCATATCCTAAAAGAGCAGCTGGTTCAAGAATGAAAAACTGCCTCCCATCTTCCCCAACAACTCTTGCTCACTTCCATAGACTTGAAGCACTACTCCTTCTGAAGAATGGAAGTCGCATTTAAAGTCTGCATTCAAAGTAACATAAACTGGAGAAGCCACCCGATCTTCTGGTATGCCATTAGGAGGAAAAACGTAAAATACAAGAATTTATCAGAAAATATGCAGCTTGCATATAAAATCTTATTGGCTCTTTATATAGTATCAGCACAAGTGCTTCTAACAAAAGATATCAAGAAAACGAAATGAGTGGAGAAAAGGGACATGCATAGACCAATTTAGACAAAGATAGTTAATGAGTAAGGAAAGGAATTTGACTGCATGTAATAGACGCGTCAGACCTATGGACAAACATTACACGCATCTCATATCACACATAAGCGAAAATGTATTAGACAATCCTAAATGGTAAACATCTAAGATTATAGCATGCTGGAGACCAATATTATTCTTGTCTGAAAGAAAATTTTACAAGTGAGCTGGAATCCATTTATTGATTAGTCTTTTTCAACGATAATTTATTCCAGTTTGGTACCCTCCAGTATACAATTCTTCTCCAGTCAGATTAGAAATGCAGGCAGGGACGGATGATGGAAGTTTGTCACATGACTTTGTCATCAGTTGATCTTCCACGACATCAAATTTGGTTTAGAAGTTTTATTGGCCTCTTATCTTGGCCTATAACTGTACAAAATTGGAACGAATGGACTGCTCACCAGTAGTTAGAGCACATGATGAAGTGTGTGTATTCAAACCGAATAAGCATCCCATGTGAGCATTGATGAAGTAGGCCCTCTTAGACACCCAATTACACTGTTTTCTTCCCATCACTTCTCAATTCTGAACCCATAAATACAGATCCAATAAACTTCAATCTCCAACTCAAATTTCAAACACATCCTCATTCAAGCACAACACTTCCCAACTACTAGATCTGCATCAAGGAGAAACCCTTTCAAAAGAAATC

**>CsSAUR15**

AAAGCTGAAAAGGAAGGATGGTTTAAGTATGATATTGGAACCACAAACCGTTTTCTTTCAATATCTCCCACATAGACTGCAATATGGCCTTTGGGAACACCACATTGATTTCTAGAAGAAACATTATGCATTTTGACAAATTGCTTAGCGTTGAGGAGAAGTGAAGGTAGTCTGATTCCCATGATTTCTTTTGAAAGGGTTTCTCCTTGATGCAGATCTAGTAGTTGGGAAGTGTTGTGCTTGAATGAGGATGTGTTTGAAATTTGAGTTGGAGATTGAAGTTTATTGGATCTGTATTTATGGGTTCAGAATTGAGAAGTGATGGGAAGAAAACAGTGTAATTGGGTGTCTAAGAGGGCCTACTTCATCAATGCTCACATGGGATGCTTATTCGGTTTGAATACACACACTTCATCATGTGCTCTAACTACTGGTGAGCAGTCCATTCGTTCCAATTTTGTACAGTTATAGGCCAAGATAAGAGGCCAATAAAACTTCTAAACCAAATTTGATGTCGTGGAAGATCAACTGATGACAAAGTCATGTGACAAACTTCCATCATCCGTCCCTGCCTGCATTTCTAATCTGACTGGAGAAGAATTGTATACTGGAGGGTACCAAACTGGAATAAATTATCGTTGAAAAAGACTAATCAATAAATGGATTCCAGCTCACTTGTAAAATTTTCTTTCAGACAAGAATAATATTGGTCTCCAGCATGCTATAATCTTAGATGTTTACCATTTAGGATTGTCTAATACATTTTCGCTTATGTGTGATATGAGATGCGTGTAATGTTTGTCCATAGGTCTGACGCGTCTATTACATGCAGTCAAATTCCTTTCCTTACTCATTAACTATCTTTGTCTAAATTGGTCTATGCATGTCCCTTTTCTCCACTCATTTCGTTTTCTTGATATCTTTTGTTAGAAGCACTTGTGCTGATACTATATAAAGAGCCAATAAGATTTTATATGCAAGCTGCATATTTTCTGATAAATTCTTGTATTTTACGTTTTTCCTCCTAATGGCATACCAGAAGATCGGGTGGCTTCTCCAGTTTATGTTACTTTGAATGCAGACTTTAAATGCGACTTCCATTCTTCAGAAGGAGTAGTGCTTCAAGTCTATGGAAGTGAGCAAGAGTTGTTGGGGAAGATGGGAGGCAGTTTTTCATTCTTGAACCAGCTGCTCTTTTAGGATATGGTCGCTCATTCAGGAATTAAGCCAAGATATGGAATGATTTAGAAATTTACATAGCAGGAATCTCAAAGTTTCAAACTAGGAACAACAGACTTGAAAGTGGAGCATGATTCTACCATGGATTAAGGTTGATTTTGACAGTGACCTGCTCTTGAAGGTTGACACAATTACTTGGGTCCAGGGAAACAGAGATCGGCTTACTGTTTTATTTGCTTTAACGCTGAAAAGTTACATATGCTTTTTCTGAGATTTTGTCAATTTGTTCAGAGGTCAGTATTCATAGTTGATGGATAAAGCCGAAAAACTGTATGCGAGATAGGTAGACATTCTTGAATTGAGAAAAAACTGAAGTTGGATGGTTTTCATTTTGAGCTCCTAACGATCAGTATGGAGGACATGTTTACTGAGCATTCTCAAGTCTTAAATAGTAGCAAACACTACTAGGAAATCTTTATCGCAAGAGCTTGATTTCAACAAATTCAGTGACAAAAAGGCAAGATTAAGTTGTGGCAAATGATTGTAGGATTCTAGATGAAGCATCAAAATAATTTTCTATCTAAAAAGGAATGTGATCTTGATATTATATTATACAATTCTCAAAGGAACGGAACATAATTCAATGCAGTTGCTTATTAAGAAGAAACAAGAGCAAACTACTGATCGCATATGAAAATGACCAATTTGTTATCTAATATCTTCTTGTCCATTCCCATCCGTAGGTTCAAAAACTTCACAAGATATGTTGTCTGGTGTAAAACGTGATATATTATATCTGACAGAATTTGGATGTTCGGCTT

**>CsSAUR16**

AATCCAAGAAAATTTGATTGTAATGCTAATATGTGAACAATACTACTCTTAGTTGGTTTAATAAACACCAAATATGGTGAAAGAAACATTCGACTTTACCCTATGAGGAAAATTAGCTTTATCGGTTCTTAGATCTTCAGCAATTCTTAGATTTAACAGATTACATTATGCTTTAAGCTTCATGATTTTGATCAAATTGTAAAGAGGAAATTGTGACAAAATCTTCTTTCGATGACTCCATTTTTCCGATTCTATATCCATATTAAGCTGAACCTATCTGGGAGTCTGTTCGCCTTCAGCTGTCCTCAATGCCAATCAAATTCTCATAGTGCAGGTTGTTACAACTAAAAATAAAGCTCATGTCCCCAACGCTCACATTGCAATTTGTGCTGGAGAAATTCAAAGGAAGTGCATTTTGGTTCCCTTATAATATTTGAATGAATTATCCAACATACGTAATCAAATATTATTAATGTTGAGGAGTGTAGATATGACCAGTCCGTGGGCGCTTTGATAGTTCCTAGCAGAGGATTTGCTTTGATCTTACTTCAAGGCTGCACAGTTCTTGAAAGATTATGAAGAAGAGAATTCCCTTAGCTACTTTCTCACCCAGTTTTAGTAAGTCAGAGTAGAATATGGTTAGTTAGTCAGTCAGCTTACGCAAGATAATGTCTAGGTTGTTTTAACTGGACTTTACATCGTAATAAAAGTTAAACTTTATCATAAATGAAGTTTGTTCCGATCATGGATTTTGGGATATCTAGTTTCCCTATGCTTTGTTATCATGTAATTAAATAAGTGTAGGTCGTAAATAAGATAATCAGCTGTAAAATCTTCAATGATTTCATTTTATTCTCATTAAATCAGCTTTTATGTAATGAGTCTTTCTCCTATATAAGGCTATGTATCTTGCAATTGAATCATCGGAATGCAAAATATTCTATTCCTTTCAAGTCTCGGTGGTATCAAAGTAAGTGATCTAGGAAGGTTCCATGTTCAAACCCCTGCTATGTTATCCTTCCCAATTAATATTGACTCCCACTTTTTGTTGGGTTCATATTTCAAGCTCACAAGTGAAGGGAAGAGTTAGGTATTAAATTTTCCTTCACTTATTAGTTTCTTTTGGTCCTACGAATAAACTGACGTGAAAGAAGCAAGATTGCACCTTCTACCTATCGTTCAGGAAAAGTGAGATTTGTAGATATCCCATTGAAAATGGTGTGCAGAAGCTTCCTTGAAATTCCTACAACAGTAACTATATCCGCAAGATCAAACAGGATTTGACACACGAAGGTGTAAGAAAATAGACAAATGACGATTCAAAGAAAGTAGTAGTATTAACATCAGAATTCCATCCATCATCATTCCCTCATTAATATGCAGCTGCTTCCAGCTATAAAATTTGTTTTAAGTGGATGAAACTATTAGATTTAACAAATAAAGACATGATTAGATGCTTATTAAATGGTTTGTCTTTATGTATTAGGCGTACGTGTACTTGGCTTCAGCCTCTGGTTTACAATATGATGCATTCGGTACCTCTAATATGATCCATTTGGTGTCTCTTTTCCAAATTAAATTAGGATATTGTACCAATGATGTAACCATTTCTCTTACTATGAAAGGTGATCTTGTCTCTGTAACTTGATGCCGTGTTTCTTTTACCTAACACACTCTTTGTTTAAGCGTTCAATTCACCGAACCAAGTTTGGTTGCTGGAATGGACAATCACTTGAACCAAACAACTGGGCTGACATTTGATACAACTAAGAGAGATGTGAAAATCATTTCTAAGTTGAGCAGATCTCTAAAATAGCTCTCTTAGATACCTAACCACCTGTCTTTAAGTGGCCATTAGAAAAGCACCAGTCACCAACAAAACTATTTTCTCTAATACTATAAATAAAGAACAAACAACAAACTGAGCTTAAAACACATTCATTCCCTAGTTTCACTTTTAATTTTGGTTCTCTACACCAATAGAACAAGCAAGCTTTCCA

**>CsSAUR17**

AATAAATCACCTTACTTTGCCAACAAAATATCTTGTTAAAAAATACATAATCTTCATCAAAGTGTGACATTTGACGATTTTCAAGGAGAAAACATTGCTATTGATGTTTCAAATGACGTTCCAAGTACTTTTAAGTGAAAAACGTGTTTTTAAGCACTTGGTCATTCCAAATAGAACTTTACTTGCATTTGTCAAATAATCAAAGTTAGTTGTTGACACCATCATCATTACCAAGAAGATGCATTATTTACACTCAAATTTACATTGCCTAGTTTGAACACGGGACATCCAGTCCACTCCTCTTGAAAGGTTTTCAAGTCCATGAATGAGGATGGGTTAAAGTTAATGAGATAACAAACTATCAATCTCCTAATCTCCTTATGCCATAGAATAGTTTGCTCTTAAATCTTCTTACTTCACAATGCATATCTTTGTGAAATGTATGATCTCTTCGTCCAAAACCTTTTGATAGAAATCATTTATGAACCTTTTGGCTTAATTTTCAACATTTCTGTAGGATGCACAATCCCCTTAATCATTTACCACTACCCAGTCTGATCTTTGGTCAATGGCTTGCTTAAACTCGAGCTCTTGTAAAGAAGACTAAATAGAGTAAACACCATTTTTAAACCGATCATTAGGACCAGCAAATGGGAACTATTTGACAAACGGGAAAACTTGTTCCTACCTTAGCTTTTTCCTTGACTTAAGAATTGAGATAGGTAAGCTTTTTCCTACTTTACCTATCTCGTATACAATTCTTCAGCTTTTCCCTTCAACTTTGAATACTTGCCAGCTCTCAACAATTTGAGAGAATTTGGACAAAATATAAGTAGCTACTCCGTTTTCAAGCAAATCTAACAAAAAGTTAATAACTCTTTTTCTGAACTCGAATAATGATTCTACCTATGGAATGCAGGACGTCCCTGACACGATCAATCTAAATCCATGGCATCACAATGCAACATCTTTTTACTATGTTGATTCTCAGTTGAAAGTACGAGATTGCCGCTCTTTGATTTTCCAATTCATTCCATTTCTCAGCCAAATTCCTTTTAGAGAGACCAAATAGCTATCTTTCCAGATACTTTATACAATATCACAAGGGGATAAGACAAGAAACAGACGAGAATTCTCAAGAAATGAAGCATGAAAGAGAAACTATGCAGCTTGTAAATCTGATCTTTCAACGACGCTATATATAATATCAACATGAGATGCCATTAACATATCAGGAAAGCGAAATGAGTGGAGAGAACTGACATGGATATACCGATCTAGACAGAGATGGCTGATGAAAGTAGAAAAGTCAGACCTACAGAGAAACATTATGCATACCCCTTATTATAGACAAGCAAACACATATTAAACAATCCTGAATGATGAACCTCTAAGATTATAGCATACCTGAGACAATTATTTTCATTATGTCCGGAAAAATGGTTTACAAATGAACTGGCATTTCATTTAATGTTTGATTAGTTTTTATCCAGATGACTTCATTTCAGTTTTAGTACCCACTGTGTATGTTTTATCTCTGCATTAGATTAGAGAGTTAGGACAAGACCTATAATAAAACCTTCTCACATGGGTCTGTCATCAGTTGATCTTCGCATGGCGTTTGGTTTTGAAGTTTTCTTGACCTCTTATTTTGGACGATAACTTCACCTCCTTTGAACGAGTGGGACTGCTCACCAGTAGGTAGGGTACATGAGGAAGTGAATGTATTCAAAGCCAAAAAGCAACCCATGTGAGCATTCATGAAGTAGGCCCTCTTAAACACCTACCTGACACTATTTTCCTCTCTCCACTTCTGTATTCCTGTATTTATAAATACAGATGAAATAAACTTCAATCTTCAACTCAAATTTCAAACACATCCTCATTTCAAGTACAACATCTCCCAACTTCTAATCTCCATTCCAGCAGAAAGCCTTTCAAAATTCGTCATGGGAATCAGACTACCTTCACTTCTCCTCAATGCCAAGCAAGTTTTCAAA

**>CsSAUR18**

CATAGTTTCTCTTTCATGCTTCATTTCTTGAGAATTCTCGTCTGTTTCTTGTCTTATCCCCTTGTGATATTGTATAAAGTATCTGGAAAGATAGCTATTTGGTCTCTCTAAAAGGAATTTGGCTGAGAAATGGAATGAATTGGAAAATCAAAGAGCGGCAATCTCGTACTTTCAACTGAGAATCAACATAGTAAAAAGATGTTGCATTGTGATGCCATGGATTTAGATTGATCGTGTCAGGGACGTCCTGCATTCCATAGGTAGAATCATTATTCGAGTTCAGAAAAAGAGTTATTAACTTTTTGTTAGATTTGCTTGAAAACGGAGTAGCTACTTATATTTTGTCCAAATTCTCTCAAATTGTTGAGAGCTGGCAAGTATTCAAAGTTGAAGGGAAAAGCTGAAGAATTGTATACGAGATAGGTAAAGTAGGAAAAAGCTTACCTATCTCAATTCTTAAGTCAAGGAAAAAGCTAAGGTAGGAACAAGTTTTCCCGTTTGTCAAATAGTTCCCATTTGCTGGTCCTAATGATCGGTTTAAAAATGGTGTTTACTCTATTTAGTCTTCTTTACAAGAGCTCGAGTTTAAGCAAGCCATTGACCAAAGATCAGACTGGGTAGTGGTAAATGATTAAGGGGATTGTGCATCCTACAGAAATGTTGAAAATTAAGCCAAAAGGTTCATAAATGATTTCTATCAAAAGGTTTTGGACGAAGAGATCATACATTTCACAAAGATATGCATTGTGAAGTAAGAAGATTTAAGAGCAAACTATTCTATGGCATAAGGAGATTAGGAGATTGATAGTTTGTTATCTCATTAACTTTAACCCATCCTCATTCATGGACTTGAAAACCTTTCAAGAGGAGTGGACTGGATGTCCCGTGTTCAAACTAGGCAATGTAAATTTGAGTGTAAATAATGCATCTTCTTGGTAATGATGATGGTGTCAACAACTAACTTTGATTATTTGACAAATGCAAGTAAAGTTCTATTTGGAATGACCAAGTGCTTAAAAACACGTTTTTCACTTAAAAGTACTTGGAACGTCATTTGAAACATCAATAGCAATGTTTTCTCCTTGAAAATCGTCAAATGTCACACTTTGATGAAGATTATGTATTTTTTAACAAGATATTTTGTTGGCAAAGTAAGGTGATTTATTAGCAATGAGATAGATGAACTCCTTTAACTTGCATATCAAGGTGCAACAATTGGCAATCACATTGGACATATTTATCGTCCTCTGCTGAAATGCTCAATCTGACTCGCATAAACAATTTTTAAAGTGGTATATCTTTTTCCAACACTATTTTGATACTTACCAAACATGTTCTAGACACTTGAACTTGAATAGTATTCATCTAAAAAAATTAGAAACATGATTACATAAAAAGAGAAGGAGGAAAATGGTATATTTTGACAACAAAAACTACACAACTTGTTATATTGCAAGAACATAACTCAACTTTTACTTTCATAAATATGGAATTATATTGTTGAGTTTTATTTGATCTGAAGTTTTCTTTGAATTCATTTTCAAACCACCATATAACAAAAAACATGTATATTTTCTCTTCAACTCCAAGAATCAGCATTTTCTATTTCCAATGGAATGGACTTTGTCTTGAAGTATCCATGACTTGACTTGTGATATATGGAAAAAACTGACGAATGAACCTGAATTGTGGTATAGAGTACAGAGAATATGAATGAGTGGGATAAGAAAATTTGTGGCCCTTTCAAGAGTTGGGCAAAGCCATGTGAACTGGTATACTCAAATATACCCAACCACCAGGCATCAATACCCCAACTAGCCACTAGAAAGGCCACAGTCACCAGCATAGCACTTCCTTCAGAAACTTTATAAATATAAACATCAAAATGAGTCTTCAAGCAGAAAATTCAACTCATTTTAAGTACTATTTCCTCTTCTAGATTGCTCTTACCAATCAGATCAGAACTTTAGATCTTTTTGACAGCCTTTCATCTCAAGAAA

**>CsSAUR19**

ATATCCTACTTGAACCATCCTACATTCATGGCTTTACTTAAGAGAGCTGAAGAAGAGTTTGGATACAATCATCCAATGGGAGGTTTGACAATTCCCTGCAGAGAAGATGCTTTCATGGATCTTACTTCAAGGCTACATAGTTCTTGAAAGATTTTTGTTCAAATGAAATCCCTTCCTCAACCTCCCAAGTTTTGCCAAATATAGTTTTGTTTATTTTGAGTTAGACAAAGTAGAAGGCCGCTGAACAGTTTTTTTCTTTCTTTTTTTTGTAAATTAGGGGATAGAAAAGGTTGTTAATTTGCCTTTCTTCCTGATCTTCGTAATGTGTATAAAGTTGTATCGACACGGTTACAACAGGAGTAATAGAAAATTACACTTGTTTACAGATAAACTTTATCCCAAAACTCATGAACTATGATACATATGTTATTTGTATTTTCTCATTTTCTTTTACAAGACATTTGACAAAACTCAAGCAAAGAGCATTTATTACCAAAATGAAGAAGTGAGCTTTCTTTTTCCATCTAATGTTGACAAAAACTGTAATTAGCCAAGTTAGAGAACTAGAACTAATCCCATCTTAATACAAGCTCTTTTGCTCATCTCAATGACTCAAAGAAAATGGTGAACTTAAGTGACATAATTGTCCAACCTTTTTTACCACACCAATATGTACATGATGTTTTAGTAGTACATGTACTTTACTATTGATCTGGACGTCGAAATCAATGGTGCAACAAATTCGCCTTTTAGTAGGATTCCCCTCTTGATCTTTCTTCTGAAGTACACTGCTTAACTATTCTGTTGAAATAAGCAAGGTAGTTGACCTTTGTGCCTCCTATTTCATCCGTATTTCATCCTACTCGTTCAGAATCTCAGTCTCAAACTTCTTGAAAGAGAAAGAGCTGCGCTTCTGGTTTTTAATAAGAAGAAGTAACATAAGCAAGGTTCATAATAGTTTGTTCTGATTGTTAAATGGATGTCATCTTGTTGGTGCTTTTACCGATCAGTATGGAGATCACAGTTGGCATATATTTGTGGAGGGCCTCCCAACGGCTATGCGAGAAAGAGCTCCATTTGTACAACAGGGTCAAGGATTTACTCTATACAACACCTGCATTTTCTTCTATGAACAGAATGTGGTCGTGTTAAAGGAACTTTGACCTTGTTGTATGTAAAGGTTGTCTTTCTGTTTAATTTTAAATATGTACACCTAAGAGAGCAACGAACAGACAGAATATCGAATAGAGAAATATGAAATGATACTGAAAAGTCTATTTGTGTTATGCTTTGGTAGGAAATTCACCAATGAAGAAACTCATTTACATTCCAGAAGGCTTGAGATTATATGTCTATACAACATTCAAACAGGAGTTAGGAACAGAAGGAAAAAATGCGTAAGAAATTGGTTGATGCCCATTCCATTATCTTCATCCTTCAAGACTGTTGCAGTTTAACCAAAGTTCATGTCCAGATTGATGAAGGCGCCCTCTTTCCATGGAATCGTTAGACCTTCATGTGGATGATGGTAACCGAACTCTTCTACTCGGTTGAGTAGTTGTTAGAATGAAGGATGATTCAAGTATGAAACAGGAACCACAAATTACTTCTTATGGGTATCTCACTTCTTGTGTGTATCTCCCACATATACTGCAGGGATGAAGGACTCTCCATTGTTAAATCTATTTGCAATGATCAAGGGACTATAACTAAACATGGAGTATATCCTTGATTGAATTTGAAGTTGGAAGTAGAATCATGATATATTAGAGTTCAAAATAGTACTCGAAACTCATTTAGCATCAATATTTATAAAGTTAAAGAGGAAAAAAGTTGCCACGAGCTTACTGTTTAGAGGACCCGCATACCATGACCATGTCAGCTCAAAAGAATTTTCTATTTTCATCTCATCCTTTTTCTTGCCTCCCATGTGTGTTTGATATGTATGGTATAACATCTAAATTATAGGCCAATTTGCAATTCTATTTCCAAACAACACC

**>CsSAUR20**

TTTTTTGAAACATCCTTTGGAGCCTCATTTGTCTGGACTAGAGTAATAATCTAGATTCCTTCAGTACAAATCCCCTTCCCTCATTTGTAATATAATACTAAAAACAAAGTTCAAAGAAGCATTTGACATCCATCTAACAAGGAATCATATCTTTCACATTGCATTTGACATGCTACCTCCTCTTTTTCATTGCAGCTTTTGCAAAGAAGGGAAATCTTTTTTGTTGTAGGAACGATGAGCATCACAACCCAACTCAGAAGTTCTTGGTCAGATTTTGTTATAAGTGAATTATTTTGAAGGTTTGTGATAAAGCACTGGCAGGAATAAAAAGATTAACTGATGATATAGCACCCAGAGAAAAAGAAGTAATCAGCAAAAAAACAAAATTCATGAGCTGAAGAGGAAAACTTTTCATATGAGAAGAATGTAATTTCAATTGCTCAAACAACGAAGTTACTGTACAGTATTATCCTACTACTTCCTAAAATTATAGGCGACAAAACTGTGATCAAGAACATTTCTTTTCTTTTTTGTTCTCTTTCATGACGTGTGCAGCCTGGCAGTGAGATTTATGAAAGCATCCTCTCGGCATGGAATCGTCAAGCCGCCCATTGGGTGGCAAAATCCAAATTCTTCTTCTGATCTATTGAGCAGATCTACAAAAGAAGGATTCTTCAAGTATGATATAGGGACGACAAATCTCTTCCTTTGAATCTCTCCCACGTAAACTGCAATATGGCCTTTGGGAACATCAGATTGATTTCTGGCTGACATAGCTTGCATTTTCAAAATCTGCTTGGCGTTGAGAAGAATCGATTGTAGACGAATTCCCATATCGAAAATTTACTCGAAACAAGAGAAAAGTGATTGAGTTTGAGCGGTGTCTTGCTTGATATGGAACTTGATTTAGAAAATAGAGTACTCGTTGGATGAGAATGGCTTCGTTCTTATGTGTATTTATAGCAGATATGAAGGAACTACTTCTTGAAAGGCTCACATGGGTTTGCTCTATCCATTGAAATTTCATTTGCTTCCTAACTGGTCCCACTTTTTCTAGTGAAGAGAGGCCAAGAAAACCAAATGTGTCAAATGAGTAGGAACATACCATGTGATACTCTCCAAATCTCATGAAGTGCTCCACCAGTGGACCTAACTAATCTGCGAATCAGATGAAATATGGTGATATGATATGATCTAATGCATCGGTTTGTGTGAAGTCGCTTCCCTTACCTAGAAGCTGGGTCCAATGATGAAGCAATTTCCTCTTGCTAGGAGGTCCGATCACAAGGATATGTCGGCTTGTTTTTACAATCCATCTGTTTTGATCTATTATTCCACAGGAACAAGCATAGTTATGAGCATACATATCCAACACACAAGGGGCCAAGAGAAAGGATGAGTGGGAATGGAAAATTCTTGTGAGCTGACAAAGTCATGGTAAGTGGGTCTTCTAAGTTGTCTAGGGAGCCCGCTTTTAAAAGCACATTAGCAGTGCTCAGTCAACATTTTTCCTCACAATTCTATAAATATAGATCGTAGATGAGTTTCTAATACCTGAAATTTCGCAATTCTACATCCAACTACATCGACACCGACCTAGATCAAAGTTGAGATCCGTTTGTTGCTTTTCGACTGTTGCATATGGACTTGAGAATGGGGATTCGTTTACCATCATTCTTGATTTCTTGTGCAAAATAGATTGTCAAGATGCTAATGGGTTTTAACATGAAGCATCAGATGTTCCTAGAGGTTATTTTGTAGTATATCTGGGAAATCTTCAAGCGAAGCTGTTTGTAGTTCCCATTTCATTCCTAGACCTTTTTCTAACAATTACTCAACCAGAGTAGAAGAAGGTTTCAGTTTCCATCATTAACAAGAATGTCTTACAATTCCTTGCAAGGACTGCTTCATTGATCCCGCTTCTCGATTAAAACACTCAAAGGATGAACATGACAAAATAACCGTTAATGAATTCTTGACATATTCTTTCTTCTATGTTC

**>CsSAUR21**

CAACCTATGTATGATGTCATTTACGTGTCACATAGTCGACTTTTTTGGCCCATAATAGAGTGCTCTACTACTTTATATTCTAACTTATTCCACATATTTTTTTTTTCACTTTTTCACCAAGCTAATAAAAAAGATCACTTATCTTGAACATTATACAATATTATGTATTATGATTATGATCTTATGTAATGTAATCACTTGTCACTATCATTTATTTTTGGGAATAAATTACATTAATTAAATTGGATTTCTTTATTTATTTTTGTGTTTGATCGATGGGAATTAAGATTGTGGACAGCTTGATTCATGAACGTATAAAAACCCCCCGTAACCGAAGAAAATGGAAGGATTCATTAAAAGACAATTTGAAGGATGATGTTTTTAGTTTGTGGGCATGAGAAAGGAACAGTCACTTTCATGTACCTCTTCAGAATATTTTTTGTTTCCATAACCCTAAAATAGTGAATTTCTAGGCTCCACTATATTCCAAATTCTTCAATTTCTATTAAGTATTTTACTACTTATAACTTTACTTTATAATGGTACAATTAATAAAATTTGTCATGATAGTAACTATATGACTGTATTTAATATAGGATTCAACGATTACTTTGTTAAATTGTAAAATTTAATAACTAAACGTGACTTTTTACGTATAAAAAAGGATTGATGTTTCGTACCTAGTTTAGTTTTAGGAGTTAAAGACCTTAATTATTGTCATTATATATAATTATTCTAATTGACATGCAATTTAAAATTACAAATTTCAGAAGAACAAATAATTAAATAATAGAATGAATAATATTCATGATCATTACCTTTCAAGCATGATACAAAATTTTCATTGACAAAGTTGAACATCGACAAAATATTTAAACATAATATAAATAACAAATATTTTGATTAACTTACTTAGAAAATCTTAGACATATTTACATACTCATTTAAAATTGTTACATAATACTTTTCATCTAAAATAAATGGACATGGAGATTTTAAACTTTAATTTGAAGGATATAGAAAATTGGAGGGGCTCACACATAAAGTTTGAAAAGGACAAAGGGATAAACGAAACTTGGAAGTGTGAATCCATGTTAAAGAGAGGGGACCCAACTGTCCTTCCTAAGGGTAGTAACACCTACTAACTTTGAAATAAATTCAAACCATAACTTATATGTATTACATAGTACTTATTTCTTTTGCTACTCATATTTGTTCTCTTCTCCAAAACAAAACTACGTGTATTTATATAATTTTGAATTTTTTTCCTCAAAATTTTATTGAATTATACATCAAATGCCCATATCCATATAGTGATATTGTTGTTACGAGTCATGTGACACATGTAGTTGATGAATAGAGAAATACATGATTTTCAAATAAACTAGACATAACATTTATTTAGTAGGCCCATCTCTATAATTTAATAAAGCTTTGGAGTAGCATTAAAAATTGAAATTTACAAATTAAGGTAGATTTTTTTAGTACAAATTCAGAGTAGAAAAGTTAAACTCGAGTTCTGTTAAACTAAAAAAAGAGTATAAAATTTCCTTTCGTGAATTTAACTATGCAAAGAATATTATATAAATTATGATAATGTTAAATTATATTATTTTTTGAATTATTATAATGCATTATGGAATAATTTATAATTAAGAAAAAACAAAACACAAAATTGGAATCTATAGGATTATTAAACAATTGAATATACGGTTGTAAGTTGTATCATAAATAATAAAATAAGAAAGAGGAATACGTGTTGGACGATATGATAAATATTATTGTTATTTCTTCTTCGTTTTCTTTTGTTTTTAAGAAAAAAAGTGGGTCCGTCATCGTACAGATATAGTCTGTCCTTTGCTTCCCCTTCCACAACCCCACATGATTTTGTTACCTCTTCTCTTCCCCCATAAATAACGAACCCCATTTCCTCATTTTTCTTCAAATTCTCATTCTCCAAAACAAGAAACCAAATCAAAATCATTTTATTTTTCTCATCA

**>CsSAUR22**

TCGATCATTTTTCTAAAATAGAAATTGTCCGTTTTTGTTATTACCTAACTCTAAGATACCAAATCGATCGAACATCAATATGATTGTCCAAAAATCAATTGTATTTAAATACCAATTACTTGAACTATCATACTCAAACATACACAAAATAAATACATGAAAATAAACGAATAATTTGTGTCGACTTTAATATTTTTAAGTATAAGAACTAAAAGGCGAGAAGAATATGAAATATCCAACCTTTAAAAGAACGTTCATGTCAATTATGATTGAATTGAAGATGATGATTTTAATATTTAGGTGCTTACAATAATGAAATGATGAGCCAATGACTTAGACCTATTCCTAAATCTAAACGATACATCTATATAGATTATTATAGAATATATTAGTTTTGATTTTAATGACTTGATTTTATATTTATTATCCAATTAATTAACAGATAATTTGGACCTTAGGAGGTATGTTAAAGGCAATGCTATTAAATTAAGGGTCAAGGTTGGAATTAATTAGTTTAAATAACTCTAATGTGTATTAAAATGAAAGAGATTAATTAGATTAATCTAACCAAAATCAATTCTAATCCACTTAACTTTACCCTCTTTTGATAACACCATTATTTCCCCTTCAAATTTTGGCTACAACTACAACATATAGTTTTCTCAATGACTCATATTTAAAGTTTTAATTTGATATAATAACTAACATATTCCTACTAACTCATGCATAAAAAAATAAAAAGAAAACAATTGTGCATTTTATGACTCATGCCTCGTTAGCAATAGGTTGTATTTGTAAAATATAAAAACAAAAATATGGCGTGGACAAATTAACCACTCCCTTCAAATGGTTGTGCTTTTCGTTAATCTTCACACGACAAAATAATAAAATATTTTCATAAATTTCAAGTTTATTATTGATATTGTGAACTATTCGTCGCTTTTTCTTTCGTTTTCTTTGTGATTTATTCTTTTTTTTTTTTCAGATTCTTCAGCTCCTCTTCCAAGTTTTCATTTTTCTATTTTTAAATAATTTATTCTTATGTTTAAATCTCCTATTTCATCTTCATTATTAATTTTTTGGCAATATTCTTTAAAGCTACTTCATCTTCCTCATATTCAAGAATATCAGCAATATCGTTTAAATATCATGATCTAAACGATCACTTACCATTATCAACACGATCATTTAGCATGATCAACATGATCATTTAAATTTGAAGTCATCTTTCCATCGTTTAAAATTATCTAAACGATCTTATTGAAATAGGTAACTATAGTTAACGCAATCATTTAGATTTGAAATATTTTTTCCATTGTTAAAAAGAACTATATGTTCATGTGGATACTACCTACACAATCGTGTAACATGATCGTTTAGAAGAAAAATTACACACGTGCATGTAACCGATTAATCGCTTGTTGATTTTGTCATTTTTTGTATTTCCTATTATGAGTTTTTTTTTCTTTTTTTTTGAATTGTTGTAAATAGTAAATATTTTACTTTCTTATATATATATTTTTTAAAGAAAACCCTTTTGCTATTAATAATATAATTACTTTTATAGGATTAGTGTGACCAAAATATTTGTGATTTTTGAATATGTCAAAATTACATTAATCATACAACTTTTGGATTTACTTAAAAAAAATATTTTGTTTTAAAAATTAGACTCACAATTTAGATTTAAAATTATAAAGACAAAATTAGGTTTTTAAATGACACATATATATAGAGTTGGAGCAAATTTATAATTCATTAAAATATAAAATTTGAACCAACTTGAAAATAGCAAAATAGTATTTAAACCAACAAAATAGCCTTCTTGGAATTGGAACTTAAAAGATATAAAAAGACAAAAAAGCAGGCCAAAAGGGCAAATTTCTAAATTCCCCAACTGGGACTCCAAACTAATTTCACTTTCTTTTTAAATACCCCCAAGTGTTTTCTTCTTTCCCAAATCTCCCCTTTCACACCGTTCATTTTCACAAAAACCAATC

**>CsSAUR23**

TAATTTAAACATAAAAACATATATACACATAATGCCCATTGCATATAATATATATAATTTCATAATAATGAGTTGTATTTGTTATAATGGCAAAGGTATATTTTGAAGTCAATCTCTCCCACTTTGTGTGGTTCTTTCCCCTTCTTACTTCTTTTCCCCTTATTCTAATCTCACTTTGCATCTTTTTTCAAATATAATGCTTCTTTAACCTATGTAATCATGCCTTCCGATACAAAATTTTTTAATCAATGTATTGGGTTTAGCTTTTGAATAATTAACATATCAAAGCGTAATAATATCATATGTTTGTAGTTAAACAATCCTTGTAACTCTTTTATTAAAGCTAAGATTAATTAGTGTTCTTTCCATCTATATCTTATTTCAATCTAAGTAAGTTGTTTAGTTGATTCTTCCTGGTGTAAATACTATTGAAGAAAGTGGTATTATTAACTATGATCAACATAGGATAGAGTACCTAATGATTAAATTTGACCCTAATTAATTTCATTAACCTTTCTATTGCTTGTAAAGGTGAATTAAACCATTGACTTAAATTTAGTTATGATATTAAATTTAATTAATTCGAAGTTTTCAATCCATACATTTCCTAAGTAAAACACAACGTCTCCATTGGTACTATAAAGGACATTGTCCCTATTTATTATGGGTTTATTTCCGTCCTTCAATTTTAGTTTTTCTTTTTTCTTTTTTTATTTTATTGATTATTCATCCCCTCTTAAATGTTGATTTTTTTTATAAAAAAATAGAATAATCTATATTAGCATTTTCTTTATAAATCCTAAAAATACATTAACAAACGTAGAGCCTTTCTTTCATAAAAATATTACTATGAGATAAAGTAGAAATATTATAATTAAATTTGAGGTCGAAGAAAACTCTTAAACCTTATCTTTCATTTGAATCACATTTTCTTCGATACTTTTCGTAAACATTTCAAAATTATACCCAAGTGAGACAAATAAAAAAAAAAGTTGGAATCTAACATCATCACAAACTATCGTTCAAAATGACCAATGTTAATTTGATTTTCTTAGTTTAATCTATTGGAAAAAGAAAAAAAAAAAGAAGAAGAAGGTAAATGAATGAATGAATTGGGGAAGCAATTAATGTTTTTGTGTTAAATGTTAGACCACTGAGCTCCCCTCTTTCTTCCCTCTTTCTCCTGTCGACTTGTCGTTCTCCTCTTCCCTTCATTGAACGACACCTCAGTCTCATGTCGGCACTTCCCTTTCTCTTCGTGTCCTCCTTCCTTCCACACGGGCGCTCTCTTTTCACGTGTCTTCTCATTTCTTAATCACCCTCATACGTCGCCGTATTACGGATGTTTTAGAATCAAACGAATAATTTAAATTTACTCCCACACGCACCTTCATTTTATATATTCTGCCTTCTCTCTTATTCAGTTTCACCTTTTACTTTTACCTTTCTTTTGAATAACATGTTTGAACAGTTTTATAAATTACAATTAAACGAAATTTAGAGTTTGAATAAATTGGAGAATTATTTGGTGTAAGGAATTGAATGAATTGGAAAAGGAGAAAAGAAAAGGAAAGAGGAAAGGGGAAAGGGGAAAGGGGAAGAGTATCTATAAATGAAATGAAAGAAGTGGATGCTTTTTGATGGGAAGGCATAGACAAAGAGGGAGGGGGAGACCCACTTCCACTTTCTATGGTTTTTAATTCACTCCCAAATTCCTATATAACCAAACCCTAATTGGAACCCTTTCTCTCTCAACAAATCTAATTCTAATTAAAACCAAACACAGTCTCATGGCCATTCCCAATTACTCCCCCAATCCCACCTCGTTTTACGCTCTCACACTTCCTCCCTTCACTCCATCAGATCCCTGGCCTTTTCCCCATCTCTGAATTCAACTTCAAATTCCCCTCCAAATCCCTCTCTCTCCTTTTAATTCTCATATATCCTTTCAAATCACACGTACATATATACTTTATATATTTTATCGAGTCGTCGTAGAA

**>CsSAUR24**

ATGTCTAAACATTACCAACCCCCAAGGAGTTATGATTAATGGCATTAGCTTGATGTTTGGTTGAGTTTAGGTATACCTAGTAAATGTAGACTTAGGTTACACAAAATAAATAAAAAAATGTGTTGTAGTTTTAGCGGCCTCTAGTGGGCTTTTGAATTGAAGTCGTCTAGCTCAAGTGGGGTTATATATTAACTGACTAGGATGAAACCTAAAGTCATATAGTTCAACTAATGAGTCGCAATCGAACAAAGTTATGATTAGAGAGCTTTATGAAGCAAGTAGGTTGGTTGTACGTAACTCGACCAAAAAAGTAAGATTAAAAGTGACCCAACTCGAATTTAATTATGATATAATCGACAACATTCATGATGTGATCACGTTTTTCTCTTTTATATTATATGACATGATATTAAACAAAAAGTAAAAAATTTATCTTGTGTTCTTTACGATACTATAATTCAATTGGTCAAGATAATATATTTACCTTTCAAAAGGTTCAGGTTGAAATTTCTCTTATTGTTGAACAGTTTTTTCTTTGAAAGAAAAAAGATTTGTGAGAGTTTAAAGTGTTTGATAGACACCACACATCAATGCACAACATTTTAACCAATCAAACTTAGGACCACTCTTAAGGACAACAAAGTCTTCCCATATCTTTACAAACGATACTAATATCGACTTTTAAAACGAGCATCTATGTACCAATGCAGTTTTTGTTAGTCCAACAAATCCAGGAGTAAAAGATTGAGTCATCAATATATCTTTGAGATGATAGTGTTACATACTCGATCGATTCTATTCCAATTTATCAGCTCAAATACCTATACATAGTTTCATTCTCTACCTCCTCAATTGTTTAATTTTTTTTTTAAAAAAATGAAGTACTTATATAATTTTAATGTTTATTGACGTGAGACTATATAATATAAATTAAAATTGAGTTATTAATCTTAATTCAATACAATTTTAACATATTAGCATATTACTTAATTTTAAACCAAATATATAAACAAATAACCTAAGGAACCAAACCCCAATTTTTTTTTTGTTGGATTGAGTTAATTCATTATTTAATAAAGGTTATTTGGATTGAATAAATTTACAACTCAGCTGTTGTGGTGATCAGTTCAACTAATTTTCTAACGAAAACAACTATAATCAATTTAGTAGATGTTTAAACCGTTCTCAGTTGTCGAGAAGTACTTATTAGTTTAATTTAGATTTGTCACAAACCAACGTCAATCGAACCTCCTCAGATTCTCCAATAACCATCTACGATTGAGTTGCTCTATTTTTCGATCTTTTATACCTAAAAGTATCTTAGTCCAATCCAACTCAAAAGCATCCCTAGTCAACAAAATGAATCCAATGCAAAGTATAGGGATGAAGTAGCAAATATACAAAGAAGAGTAGTACTTTAGATTCCAACAACCACCCCAAATCCATTTTTTTAAAATAATAATAATAATAAATAAATAAATAAATGTTTATTGTTATTATTTAGAAAAATGAGGAAAATAATTTTCAAATATAAATTCCACAATTTACAATCGTCTCCATGTTCACATTTCCAAATCACAACAAAACAATAAAAACTCCCACAAAATACTTCCATTTCCACTCTCCAATGGCCTCCTCTTCATCTTCAAACCCCTTCCCCCTCCTTTGACCTCTCTCCATCTCCACTTCTCTCCGCCGGACAACTTATGATCAATCTCTTTCTTTTCAGTTTCCTCTAATTTCTTCTTCTTATTCTCTACGTGTTTAATCAATTCCTAACCTGCTTGGATGATTCCTTTCTCCTTCAATTCCAATCGCTATGTCCAACATTCCCACTTCCCTTTCTGAAATCTGCCTCACTTTCTTCCGCCTCACCATGTCCACCGCCGATCCTTGGCCCTTTTCCTAATCTACACAGTCCACGCACACCGCCGCTAATCCTTTTTTCTCTCCTTTCCCCTCTCTTTTTAATTAATTTCTTCCATTTTTCCCTACATACG

**>CsSAUR25**

AACTTGCATCTATCGTTTTGAGTACCAGAAGTTCGGGTTCCAATCTCTCACTCCAGCTTGTACTTATTATGGTATTTAATAAAATAATAATTATATGGATATATCAAATATTTGGTTAGAATAATCATTTTTTATGTACTTTTTGTTAAATGATTAGAAATATGAGATTATTCTGACTTTATTACTAAAAATATTGTAATTTTATTTGGAGGCAAATTGCAAAAACATTGGAGTTGCAATTGCACTATTACATTTTACCTATAAATGATAAAATTTAAAACATTCAAAATCATACTATTATTAAGTCTTTTATAAATTTAGTGTATAATTTTAACGTTTCTATAAATTTTGAAATTGAATGAACAAATTTTGAATTGAATTTTCAAGGATATGATTGTAACTATCACTATAACTTCAAACGGTGATTTTTTACGTTTATATTATTTATTATTATCTATTTTCATCGATTACCATATTTTGAAATTTTGGATTAGACTTAGGATGAAACAAATAAATACTGTGATGAATGGAGACATTATTTAGTAATTTGGAAAGCTTGAGTTCGACTCCCAATTTTTTTTTTTTTTGGGCTATATCAATTTAAACTTTAGATTTTTCATAAATTCATCAATTTAAACTATAGACTTTCATATGTGTATCAATTTTAACTCTCAACTTACATAAGATTGTTTAAATAAAACGTAATTGAATACTTAAATTGATACATGTACAAAAGTTTATAGTCTAAATTGATATACTTGTAACATTTAGGATCTAGATTGATACAATTATTATATTGCAATGAAATTTAAGACAACTCTAACCGTTAAATATTTAATTTGATATAATTATTAATTTAGAGTTTAAATTTATATAAATCTCAAAATTTAAAGATATAATTCGAAACTTTCACTTGTTGTTAGGCACAACTTTCAAGTTTGTTTATATTCGGGATAAATACAAATAAACTAAAAGACTACCTAGGTCAACAGAACCCTCGCATTATTTTTAATCGAACTCTTTTTTTATAAAAGCTATTTTAAAATAGATTTTGAAATTATTTCAAAAAATATTTTAAGTGAGAATTGATTTATTTTTCAAATTTAAACGCATATACCTAAAATTGGTAATTTACCAATAATTTAGAGAGAAAAAACCAGAGGTTTCGAACTCTATTTTATTTTATATTTGTTTTCTACATTTTCTTATAACAACACCATTTTATTTGTAAGCTAATTAGATATTATAGTCTATATTAATAATTGAAATATATATATTCTATTGCATTCATTTCATTATTATGAAGAAACTTAAATTCAATTAAATATCTATAGTTGTTACCACATTACACATTTAGTCTATCAATATTATTCATGCTTTTGTTTCAATTTGAAACGAATATATGTAAACCTAAATACTTAATTAAATTTATATTTATTTTTTAAAAAAATGCAATAAAATATATATTTGTGAATTGTGATTCTTATTTGAAGATGTAAAAACAACAAATAAAAAAAGAAACGAAAATCTAAAAAGTAAAACAACAAATGAAAGAGAAAGAAAATGAAAAATGATATAAAAGAGAGAGAAAGAAATATAATCATCATCCTCGTTCGAAAAGATGATAACAAATAAACTATATAATATCTCAATAATAATTAGTCTCAAAACAATTCAATATTTTAGTTGGGCTGTATCGTTTTTGAGTCTCTCGTCATAATTCTTGAAGCTCCAAATTATTATCCTCTAGCTCCAATAAAACTACATGAAATCTCTATAATTATTTAGTTGATCGTATTTATCATTTTGTAATCTTTCCTTTTCACCATTTTTAAACTCAAACTAAAAGAAAGTAGACATCCTGAAAGAAACCAATATATACAGAGATTTAGAGGTATTTTAACCAAAGTGGAAATTGCCAAAATCATATAAAGTATACAATTGATATTTTTTCTATTTCCACTCAAACACTAACAAAAAAGAACCCCATA

**>CsSAUR26**

ATTTATTATTTTATTTACTGTTTCCAAGTGTTGTTTATTACATTTTAGGGTTTTAAAATTAAAAAGAGTAACTTTTAAATACTTGTTTTCGTTTTTAGAATTTCACTTATATTAAATATAATCGGTAAAAATAAATAGTTATAAAATGGAGTTTTAAAAAGTATTTTTAATTTATTTTTGGGGATTGGGCTAAGAATACATTTTTTTGAACTTGACTAAAATGCAAATGGTTGTAAGTAGTTGAGAGGAAATAGAAGCCAGAAAACCGAAATGAGTTAGTAGTTTAGTTTTGGTTTTTAAAAATAACCCTATAAACTACCTTTTAATTTTTTTTTGTGGAAAATTAGTATGTAACTTTTTTCCAACATTTTCAACCAACGATTAAGTTTTGAAAAATTTGGTAAGAACTCAACTCTTCCGCTAAAAGAAAAAACAAATTACTGAAAAAAAAGGCTTAATTTTAGAAACAAAATATAAAAAAATTATATAGTTATTGATGAACCTCACCCAAATTACTTATAAGTTACATAACCGGTTTATTATTATACTTCTGACGAGTAAGAGTGGACTTGTTGTGATTGGTTTTCTAAAAGATTGGGTTATATATTTTAGAAGTAAAAAAACAACTTTGGAAATGAGGCATCTAGAACATCATACATAAATAATAATATATATGTATTCTGAAATGAAAAGGAAAAACAGTAAAGTATGATTTGAATTGTAAAATTTTGGAGGGATTGCTATAGGGTAGGATAGATAGAACATGGGGTGTTTATTTATATATATATATATACACCATCTTGTTCTTGGCAGTGATATACATTAGAAGCCCACTGTTTTTCTTCATATCAACGTGACACAACTTACATTTATTCTAAAATAAAATACACCAATATTGTACATCCAATAAATTACTTCTATTCAAAGTACAATAATGTCCAAATCTCAACACTTCGTGTTAAAAAAAAAAAGAAAAAAGAACAGAACAGAACATTATTCATCCGCATGGTTTTGATAGTTATAAGAAAATTAATCAATATTGTTTTCAATATGCACAAGGTTAATTGATATACTAATAAACAATAATACAAATTTGTAACTTGTTTACGTACTGTAAAGTACTTTATTAGTTTTTTAATTACTTACTTGTGAATCACTAAGGACAACATTATTATTATTAATGTATATGTTCATAATATCTTGGAGACCTCCTAATTATATTTTCCATATCAATATCTAATTCCTTATAGTTTATTTTTTATTTTTCTTTTAGTAATTTCCATTAATCAAAATTTCTTTTATTCAAAGGGTCCTTTTACTAGTGGAAGAATGTTTTCTATGTATAACAAACTCATGGTCTTCTCTTTAATAAAATAAAATATAGGAACGAGTGACTTTAGATTGTTAATTTTTATATGGATTTAAGTTTGAAAACAAAATATTCAAATGTGAATTGCCAAATCAACGTTAATATATAAATATAAAAGAGTAATGAGGGAAATGACATTTAATTTTAGTTTACTTATGAAATATGAGCTGGGTGAGCAGGCCAGGCTTTGGAAATCCAAGTTTAGTCAACTTAAAATTGATATTACCCCAAATTACACAATTTTGTTTCAAAGGGGGCTAAAATTGGTTGTTTATTATTATTTAAATACAATAAAAGAAAATAGATGAAAAAACAAAATTGATTACATTAATTTTTGGATGAAGTATAAGTATTTTATCAAAATAACTATTTTTGACAATTTTGTAATTAATTTACTTGGAATTGAAAAGACTTAAAAAAAGAAGAATAATATAATTAAGGCAGCAACGTAGGTATAAATAAATAGAAGCGGGGGCGTAGTAAATATAAAAGAAAAGGAGGGGTATTATCGTCAACCGAATGTCTATAAAACCCTCCGCCATTGCCATTGCAAAAACTCCCCAACTTCCAAGCACATCCTCCAAATCTCACCTTCAACCTTCTCTTTTTACTTTTCCCCCACAATCGGAAA

**>CsSAUR27**

TCACCAAACAAATGGATGGCATTGATGAGGCCTCATTTTGTAACCAAAAACATATGCATATATAAGGTAATAATAAATTATATTAATCAATAACAATGTATTAAAGTTTTTATTTCCGTATCATATTATTGTACAGCTTAAACATACACCAATAATAACAATAATAAACACTAGAACACTGTTATCTCAATCATAACAAAACAATAATAGTCTTTTCTTTAAGATTTATCATTATTGAAAGTAGAAAACTTTAATATTAATTGTAGAAAAAGAAGAAGAAGAGAAAGAAAAAGAAGATATGGTTTTTATGGCCACAGATGGTGGAAAAATGAGATGAGATGATTAAGGAATATAATAGGATATTTGTGATTGAGAGTTTACAAAGATTGTAATATATTTGTGTTTTGTTTTTAGATTCATTGGTATTGGGAAGTACAACCACTAGTTGTGAGTACAGTCATCCTTTGACTTCAACAGTGCTTTTCTTTTACCAAAATTAAGTCAAACCAAAACCCACTCTATATTTTCTTCCAACCATATACTATTTTCATAAAGACTGCTTCACTATCAGTTTCTCAAAAACAAACACTATTTTATTACATACAACATTTTTAATACTGCCTCTATCAGAGATTAAAGAATTATAGTGTATTTCTTTAAACTCTACAACAGTCATATCCTAAATGACTATATATATATAGTTAATTATATTAGATACGATTTCTAATATAACATATGCATTCTAATTCCAACCCCCTAACAAAGATTCTAAATCAAATCAACAAATTTGTTGACTTTTTTTTTCTTTTCCGTGGACGACAACTTTCAAAATTAATATTAGGCCAAAAGGTATGGATTTGAATGGTTGTAATTCAATTTGGGGGCAGCCCAAGAATTAAAGCAATTGAATAAAATAAGGTTTCTTAAGAATCTCTAGAATTAATTAATCAGCCAAAAAAAAAATATCTTGAGAATAGTTAAATTATAATTTGGGAAAGTTAAAATTCTATCTAACTAAGTCATTGTATTTTTAAGAAGTATTTAAGAACAATAGAAATTGATGTAGTTAGATTATTTACCCACACACACAATAAAATAAAATACTTTGCTTTAAGGCATGATTAATGCTGAATCTTCGTGAATTTATTAGAAAATTTATTTTTGAAAAATAAATTATAAGAATATTAAATTAAAAATAACATATTTAGAAATATAAGATAATGAATGTAATCACGATTTTTAAATCTAATCTTAGTAAATTGATAGTTTATTTATCGACGTATAACTTAATAAGTCAGGGAAAATTTTAAAAATGTGTTTCACTTGGATTTGTTTTGTTGTTGTTTGTGTGTATTTAGTAATTGTAATGAGACGTAGACTCCACATTAAATTATGTTATGAAAGAACGTAATTTGATTATGTTAGAAATTTACGTAAACACATTAAGGGTTATTGTTAGAATTAGTATTACTTGTATAGGTATTAGAATTAGAGTTGGTAGTATGGAGTGTTTATTGTTATTATTAATTGGACCATTATTGGTGTGGTTAGACCACATAATTGTATAAGGGTAATAATAAGCATTGGTAAAATGTTATTAAAATTGGAGCAATTTAGAATATGAAATAGGGGGGAGGGTCTAAATTAATTAGGAATAAAGAAAAGAGAAGAGGGAAAGAAAGAAGAGGGTAGGGGAAGGAGGGGCCAAGTTGTGGGAAAGAGTAAAGGGTCATTTTGGGCTCAAAACAAGTAATTAATTCACTTCTCCCAAGAGAAAAAGAAAAAAACACCAAATGGTCTCTTATTAATATTTACCCTAAGCTAAACCCAACCCAAAAACTCAAAAGGCCCTGTTTCTCTCTGTTTCTTTTTTTTTTTTTTTTATCACCATAAATACCCCCCCTTCTCCTTTCTCACTTCTCTCGCTCTTCACTTTCCTCACTTTCCTCACTTTCCACTCTCTTTTTCTCTTTCTTTCTTTCTGCCTAGCTAAACCCAAA

**>CsSAUR28**

CCCTGGGGTAAATAGTTGGATTATTTTTCTCTCTTTAGAAGAGAAAAATACTCTTATTTTAATATAAAAATGTTTCTTTATTTTCATTTTAAGAATTAAATAATTAATTAGTATAACTACATCAATAACCCCTATGTATTTAACTAAGTAATTATTAAATATTATTTTCTCCAATAAATTAAAATTCTCGTGGGCTAAATTGAACCCATACTCTTAATCAATATTAAGATTTACTTAAAACCACATATAACTAACACCTAAACATATTCAATAGACTGACTCACACACCCTTTTTTTTTCTTTTTTTACTTAAAAAGAACACATATATTTAACATTTAAACATTAATTATCTCACAAATTTAATTTTTTATCTATACTTGTGATCCAAACAAAGAAAAGACAATTCAGAGAAGATGAAGATCCAACAAACTGATATATTAGAATTAAAACAGACTGTCAAACAACTAACGTTGAATAATAAAGTAATTTAATTTCCCTTGGTATAAAATGATTATAATTTATATATAAAAATAAAATTTGACTTAGAGAAAAAATACAAAATTAAAATATTATTGATATTAACATAATCAAGTAGGATTTATTTGAGATTAAAATAATAAAACGTAACAGGTGTACGGCGATTATAAAAAGGATTGCTGAGGGGAAGGTTAAGCAAAGAAATGAGTGATGAGGTTCGACTTTCGAGTCATTATTGATACATATACTAATGGCTTTAATATGCTAACCTCACATGGGTTATCTTTACCGAAAATAGGAGCCAAGAGTGATTAAATGCTATAACTGTCAAAAGTGTCACACTTTATAGTTTAGTCCTTTTATTTATTTTAAACCTTGGGTTACGCACCCCAACGTATTATTAATAATAATAATGTTCATGAACTTTGTCATAATACTCCCTTTTAAAAAACAAGTTCATAATACTCTTTTTTAAAAAAATTATTTGTATTTTTGTAATTTACATGTTCGTTGAAGTAACAATTGTGACACGAAAAGTATATATTACGTGTGATTTAATATGAATATAAGTAATACCTAATATATTGAATTTTACTCACATTTTCTTATGTAGTATGGAGAGAAAAATATTGACATATAATAAGTTGATAATTGAATTCCATTTGTTTGCAATAAAAAAATGTTTTTTTTACTAATTTTGAAAATAGGTGATAACTAATAATTGATGTTTTGTTTTAAGTTTATATTGAGATGAAATTGTAGAATTTGAATAGTGATAATAAATAAAATGTTTAAAGTTAAAAAAATAAGAATAAGATTTAATAAAAAATATTTACGATGGTTGATTAGATAAAAATTAGGAGTTAAATAAAAAGTTGATATGGGCATAGTTTAAATAATAAAAAATACCTTGATCACGAGATTTAATAGTGGAGTAACTTATTATTTCAATTATTAGGGTAATAATTGAAAAATTATTATAATTTTGTATCTTCAATTTTATTATTTTATTTAATCTTAATCGAATCCATTCAAATTTGAAAACATGCACAACAAAATGTATTAAAAAAATACAACAAACTTTTAAGAAATGAGCTAAATCCAAAAGCTTCTATATATAACCTTTTATACGTTAGTTATTAGTTAAAATTTAAAAGTTGTAAGCGGTTAAGATTTATCCTTAATCACTTTTTACGTATAATCACTTATTCTTTTTCCTAAAATCAACAGCAAAACAAAAGCTTAATATAAATAAATTAATTAATGGAGAGGACATAATGGTAATTACCACCATAAAGAATTTAAAATATTATTATTATCACAGACGATTTAGGCAATCTCAACAGAAAATACGGATAAAATTACTCTAAAATCAATGAAAATTTTACGTTTTTTGAAATCCGAATTTTCCAAACTCAAACTCACTATAAATTCAAACCATCAATAACAGAAACCACCCCTCTCCATCTCCATCGCCATCTTCTTCCTCACTTTCCTCAAAACAAAAAAGAAGAAAATCAGCA

**>CsSAUR29**

GGTTCTTCCTCTTAACTAATCAACATAATAAAATGACAAACTAATATATGTTATGAACATTATGGTTTCAAGGTGGAAAATTCAATTAACATGCCCTTCCAAAAATCTCTAGATCACCTTAATTAATTTGAAAAGTATATGACTTCAATAATAAGCATTGATATAAATAAATAAAAAAAAAAATCCCTCCCACCCCACCAATCCTAAATCTATTTCTATCTCCATTCCACTTTTTTCGTTTTTTTTGTGCATAAAATTACCTCATCTCAATCATTTCTCAGATCTATCCATGGCAATATATCACCTTGCACATTAATGAGAAGAACGTTAAAGGGTTTTGGTCCTTAATCCCAACAAAAAAGAGTGCAGAATCATATATAAAAAGGAAAAAAGATTAGCATGAAGGATAAGATGCACAATAGGCATATACTTGATTACAAGTGCCAAGTTTCACAAGAGAGAGAGATTAGAGAAAGTGTTATGCTCGAGATTTTGAGTGGAGTTTGGGGTCCAATTCACAGACCAACTTTTTTTGTCCACTAACTTTGAAATCATGGAGAAAGAGAGTGTCCTCATCAATATCATCAACCAAAGAAGAAAAAAGATCACTTAGACTGTACGGGATATAGAATTTGTGGACCCTAAATCCTTTCATTTTAAAAAAAAATAAATGAAATACCTTCCTCCCTACACCACACAACTCTTATGCTTTTCCTTTCTTTTCCTTTTTTCATATTATATTTGTCTAATCATAATATGATTTTAATTTATTGATTAAGACATATACAGTATTTTCAAATCTTAAGAACAAATATCTTCTGTTAAATTTCTATTCTACTCCTTCAGTGAATTTTGGTTATGGAGTATGTCCCTCAAGGATGATAACGTCTACTACTTATAAGTAGTTTATGTTTCTCATTGTAATTAAGGTTACATTTATTTACATGCCATCCTAATCAATCAATAGTAATATGAAAAAGTGAGGGCTAATGACTTACGATTTTCTGATAATTCTATCACTATATAAATGAATTAATGTTTGGTTGCACTATTAAAATCATATTCTCGACCTGTAATATAATCACACTGTGTGAGAAGGCAATTAAGCAATTCAATTATTATTGGGTGAAGCTTTGAGAATTTTTTATATGTTTCTCTCACGTCATCCCTCCATCCGTTCTAGTGTTTAATTAATTTCAATATATATATATATATTGATATTGGTATGATTGAGAAATACATCTTACTAAATAGTCATATCGTTTTTAATCATGTTGTTTCGATAGTTGTTGATGTCATTATATAAATATTGATTACCGATATTTCGATATTTGTATTGGTTGAGTGTCGATATTGTATATATAGATGTAATCTAAGCTAAGTATTGATTTGTACACCCTAACTAACCTACCAAAATAATAGTATATAATAAGCTAAGCTAAGTATTGATTTGTACACCGTAACTAACCTACCAAAATAATAATATATAATAAGAAAATTTACTTTTAAAACGATATATACTACACTACCCACCAACAGTAGCTACTTTCTGGGGTGGCATATTTTCATGGCCTATTTTTCTAATTAAGATTCACACATTATTAAATAACATATTACTTTATTTTCAAAATTCTTAAATATTTCCAATACCAAAAACAATGAATCAAAGATAGCATGAAAAAATGTTGAGTTCCACAATAATACTAAAACTTTCCAACTTTTCTGTTCTTGAAATGCAATAGAAACAAGGCAATAAAAGAAAAAAAAGAAAGAAAGAAATAAAGAAGTGGAGTAGTACACGCTCTATCTCATTCTAGTTCCACAAAATAATAAATGAGAAAAACAAAAATGGACAAGTCAAAAAGCAATCAATACATGATTCGATTTGACAGTCTTAAAAGAAAACGAAATTCCTTTACACCCACCACTCTCTAACCAAGTATGTGTGTATATATATATATGTCCATTACAAACAATGAAACTGTGAGAGGAAAAAAAAG

**>CsSAUR30**

TTAAGTAAATGAAGGATATTTAGATGGTGAATTGTATTTGGAGAAAATGTATAATTGAAGCCACTTGCTAAGAAGAGATAATGTAAAGCACAAAAAGTTAAACAAGTTTTTAAAGAGCTTATGATTAATGATTATTGTGTTCTTTTGTTTTTGGCAAAGCACACAGAGAACCAAACCCTACAACCTTCATGTTAGATAGATAATTCAAATGATATAAAGTATTCGGTTTGCCATTTGGATTTGTATTACTTTGAGACGTGAGATGCAAACAAATTTAGTAATGAATTTGTTTTATTTTCAAGGATTCTTTGTTAATTTAATTTTTTTTTAAAAGATTCTGACTCAATTAATATAAATAAATAAATTTGTTTTTATCCTCGTCCAACAAATAATTGAGATTTTTTTAATTAAAAGATAAATATACGAAATTATATTTTGGGTTACATAAATTTATAATTTTAAAATACGAAGAGTTTGCAAATTTAGCAAAGAAGGGAGCAATTAGATTTAAAATAATTAAGTATATAACAACATTTTAAAAAAAATTGTAAATATAGCAAGCTTTCGATTGATAAGAAGAAAATTTTAAATCGTCTCAAATCAAGTCAATCACGTATATCAACCTTGCTTGCGTATCAAGATTGGTTTGTTAGGGGATTCCAAAGAATTAAGAAAATATGTTTTACCCCACAACCGACGATGAATATCATACCCATCTCCAACGCTTGGAAGATACATTCATGGAGAGTTATTGCTAATTTTGCAAGTTCGAGACAAATCTCAACTATGCTTGAAATCTCATGATTTTTTGTCATTTGTTCATATCACGAACAATGACACTAACGTTCACTTCATCGTCATTACAAATAAATCATTTGCTTTTTTAGTAAAACAGAAGTGTATGGATATTGAAACTACATATGTCGTGATCAATAATACTCTCGTATACCAATTGAGCTATGATCGAGTTGACATGTATAAAATTTAAAACAGATCATATTAAATCCTTGCTAAGTATCATTGTATTTATTATCGTGTACACATTCCACCTTATTAGGAGCTTTTGTAATAAATATGTGTGTAACTTTCATTAATTTATGGCACCTTACTTATATTGTTTAGCTTCTACTCAACAATCCTAACCTTTAAATAAATATATTTTTTATGAAAATCTCTCATGAATAAATAAGTGGGGCATAATTTCAAACCAATTATATGACATATTATTCAAACATATACTTAGAGTAAACGTTTTTCTTTTAATCGAATTATTCAAATGTAAATATAAGAGTATAATGATGCATAATATGAATCATCATGGTAGAGTCATTTTCTTTTTTTAATAAATGTTAGATGTATAAAAGAAACAATTTAAAAAATACATAGGTAATATCATTTTATGTAACATGTATTATTCAAAAGTCGGTAAAAACTTCGCTATAAATTTTGTAAACTTTAAACTATTTTGTCAAAATTTACTCGTAACAATAATTTTATTACAAAAAAATAATAATAAAAGTGAAATTATCGTAAATAGCAAAAATATATTTACAAAAATATAACAAAAATCCATATTCTACCCTACTATATTTTAGTTTATTAATTTTGTTATATTGTGAGAAGTAAGACTCAAAATCTCTATATATGTATATATGGATGTTTATATTTAGCTTCTTATGATTCCCGAGCCTAATTCGTAATTCATATAATATATTTTTACATTTAATTTTTTAAATTGAAGAAAAAAGAATGAAAATATATAATATATCCTTTATTTTTTTATAACACAAAACAAATGGGTGATAATCTAGTTGGCCAAAAAGTGGAAATGATGTATTGATATATATATCACCAAAAAATAGGGACTTTCCACCAAAAACCCAACCCCCCCATCTCTCTTTTCTCTCTCTCTTTTTTCCTCAATTTCAGATTCTCCTTCAAGTCTCCTATATAAATCTCTCTCAAATCAAATCAAGAAAAGAAAACTTAAAAAGTAAATA

**>CsSAUR31**

ATTTTATGAACGTGTAAAAGAACCTTAAACGTTGGTGTAGATTTTGGATAAATTATAGTTTTTTAATATTTTAAAACTCAATCGTATTTATGAAGCATTTAAATAGTTCATAAGGATTTTCGTAAGAAAACTTCAATAATTTTCATAAAAAACTACTAGTAACAGTATTTTTTAGGAAAATTTTTAAAAATAATAATTTTACAAAATATTTACAACCTATAGCAAATTCTATCGTTGATATGTTATAGTGATAGAAACTATTAGAGATACAATCCAAAATTTTGCTATAACCGGTAAATATTTTAATTTATTTTGCTATTTTTAAAAACGTCTCTATTTTTTAACCATTTTCTAAATTTCAAGACCATTTTAAAAAACTTTTAAAAGCTCATAGGTATTTAATACAAAATACAAAAATTGATTGAAAATTTCTCTTTGAATTTGGACTTAGTGTATTTATTTTTTTTCGAAATAAAAATGATCATAATAATTGATGTTGACTAGTAATTTAATTCCCATTTTTATAATCATATCCCTAAACTTAGAAAGTCCACGAAAAAGAAATAAATAACTATTTACAACATGGAAACAACTAACAACTAAATGCTCTCTTCTCCTAAAGTTGTGAACCTCTACTCTCATATTCAAAAGTACATAAAATGAGTTAGGTTTCATCATGCATATCCATCGTTTCACCTTTTGTTTACAATATTAATCATCGTTAAACTTATATTTGATTAACTTACATGTTTCAAAAAAGTAACAAGAGATAAAAGGAAAGTTCCCACGATTACAACTCTATATATTTGTTCAACTCAATGAGAAAAAAAATGTGATAAAAGATGTCTAAAGTACTAGAGACAATTTCTTTTTTTCCTTCTCAATAATCATTATAAAGAAGTTGAAAATTAGGAAAGTGCCATTCCATATTTTAGGAAAAAAACAACATTGTATTTATTTACGAGGAGATGAAAATGTAAATGAGTTGAAGATTTTAGAATTGACTACGAAAATGGTAAAAAGGGAACATTATTAAAATGGAGAAAGGAAACACGTATAAAAGAAGAGTAGATATGGTGTCCGTGTGATATTAAATGTTTGACCATCTAGAAGGAACGAATAAGGACTTGAAAAATCTCTTAAAAGGAAAATAAAAATAAACCCTAAATTCCGACTAATAAAAATAATAATAAATCATCTTCATGGGTATAAAAAAAGTAATTATAATGAATGACCATTTGAAAAATAATAATTAAAGATATAATAATATCTTTAAAAAATTGCAAATATAACAAAATTACGTTGATCGACTTGTATAGCTCATAGACTCGTATGGTCTATTTATAACATGATGATAGACTTCTATATTGATAGAATTTGACAAATTTTGTTATATTTACAAATTATTATATATCTAATTATTTTGAGTTTAATGGCTAAATTTGCAATAGAATAAACGTTAGTAAAAGTAAAATTGAAAGGTTGAAGACTTAAATAGGGAAAAATCAAGGTATTAAAGAGGGAAAGTTAAAATGAAATTGAAAAGAATAAATACCCCGTAAAAGAAGGAATATAATAAATAAATACATAAATAAAGGAAGGTTTGATGTCATTTATTTGGTAAAAAAGAGAGAAGAAAAAGAAGAAGAGTAGAAGTAGAAGATTGTAGGGGCAGGGCAACCGACTCTGTGTTGGAGTGGAAGCTAAGGAAACGCAAAGCGAGGGCGTGCCTGAGGCTGAGGCTGACCCTTTTTCACACGGACTTGGAATTAAAATTCAATTGCCTTAAGTTTCCTTTTCGGGATATAAATACCACGCTCCGGCTCTCCCAAATTCTCAGTTCCTCTTGCTTTGTAATATCGATCGTATATGTTGAAGAAGAAGCGTCAATGACCAATATCCCTCCCTCCTTCACTCATTCCTCTTTCACTCTCTTCACCCTCGCTATCTCCTCTTACCATCCTTGGCCCTTTTCTCTTTAACACTTTCACCTTTTTCCG

**>CsSAUR32**

TCTAATTAATTTAATTTAAGTTTATTTGTTAAAAAGTTTAATTAAACCCTATTCACCCACCTTTAAGGTTGCCATACCGATGCATCCAATAAGTGATATCAATCAGCCCAAGATCCACCAAGATTTACTCCTTTCTTGGTCAAACAAGCACCAAGATCTTGCGCAAGAACCCAAATTGTCCTTCTTTGCTTCCAGTCCCAGGCGAACAAAGCCCCGAGATGGCTTGAGTTTTTCTTACCTTTCCCTTCGAACTTCCAATCTCGGCTGGACACGGCCCCAAGATTGCTCGAGATCTTAACCTCTTAACCTTGTGCCCTTCATCTCGTGCTGACAGAGCCCCAAGAGCATCGAGATTTCACCGCTATCTCTACTTTGCCTCTTATCTCGGTCAAACAGGACCGAGTCCTGAAGCCTCTATCCTGAGATTCCTTTCTCAAGGCACTTACTTTGGCTAACAACCTACTTTTCCTTAACTTTTCTTCCTATCAACTCAACACAACTTCCACTTCAATAGTTAACTTGAATCAAATCTTAAAGTTTCTCTTTCTTTACGAAAGCTTAACAAGTCCTAAAGGTTATGAAAATTCAGAATTTCACAATCACTCTTAGTGCTCAAGTAATCTATCTTATACTTGTCAAATTGCTTGCCTATAAATAGGAACATGTGATGGATTCGTAAGACACTCATATTGGAGAGAAATTCATAAAATTTGGAGAAAGTGAATAAATTTAAATTTTTGAGTTTTTTTTCATAATTTTTAAATTTTAATATTATATTTTTTCGATATGTGGAGTCGGATTGTGACACGTTTTTACAACAAAATCTATTATTTTGAGAATTGTTTGTAAATTTACTATAATTATCTATTTTGTGAAAAAACAGCCTTGGAAGAAGTTTACACCCACTTGCAAATCAAACCATGCATAATGAGAAGTTGTCAATTGACGTAGGAAAAATAAAAAAGAATTGATACAACGAGAGTGAGAGGGATTGGTAAAGGGAAGCGTTGAGGAGTTGTGGACAATAAGAAAATAGAGAAACAGAAAAGAAAAAGGAAAGGCAGTCCGAAAGGATCGGGAGAGAGGAGAGTGGTTTTTGTAACGCTCATTCCTCTCTTCCACATAAATATCCATTCTCTTCTTTCTTCTCTCTTCGGAATTTTCTCCTTCTGCTTTTCCCCCATTCATTCATTCATCTTCCTCCTTCCAATGCCTTTTCTTCCTCACCTTTTCACCTAAATGCTCTTCTTCTTCGCCGGTATTCTTCTTCTTCTTCTTTTTTCTTTCTTTTTACGCCTAATCTATATATGTTTTTTCATCTGATTTAGCCATTTCCTTCCTTTTTCAATGTCAATATCACTTTTCATCACGCCCAGTTCCTATTTTCTCAAAGGCCTTCTATTCTCTTACTTTGTTTTTCATATCTGATTTTCATGTTTGCGTTTTTTTCTTCTTTCTTCTTGTGTTTCAGACATTACTATCCAACAAATTGGGTTTTCTCCCCTTTTCTTTTCAATGAGTTGTAGGTTTAAAGCGTAACTAGATGACGTTTTAAGCGGATGACTCTTTTTTATCTTTTTGGATTAGAGGTGGTCCTCTGGAATTTTTCGATCCATTGTTTTGACTCATTCTCATAACGGAACTTGTATCTCCTGTTCTTGATGTTGTATGTACTTTTGATCTTTTTGCTGTTTCTTGCTTTACTTTACCATTTACACAACTGTTCTATTGCCAACAATTTTTAGCTGTACATTTTGTTATCTGAAACTAGGATTGCTGGATATGATTTTAATCCTAACCTTTGAAATGGATCATTTTGGGAATTGGTGATATGTTGTAGCATTAAAATGGGAAGTTCTTTTCCTTGTTTGTTCAATCATTTGTTTGGATTTAAGTGTGTAAATGATGAGAAGTAGTGAGCTGATGAGAATTGTGTTTGTGGAATTAGACACTGTGGATTCTGTGCTGGGGTTCTGTTGAATTGTGGTGAACGTTTAGAA

**>CsSAUR33**

ATTCATTCTTCACGCTGCTTTGATTTCTTTACCAAATATTCACTTTAGTTTTTGGAGTTGTTTATTAATTAATTTACAAGAATTAACAACAATTCTACTAGAATTTTGTCACTTTTTATTGATCCATGCACATAAAGAAATCATACATATATCTTTTGGCCATTTAAGAAAAAAATATATATATAATAAAATACCACCACGATAGATTACTCTATAGTCTAGTTACTCTATATAGTCTCTTGTAGATAAGATTATGTGATATATATTTTGCTATATTTATAAATAATTTTAAAAAATTCATGTCGGTTGAAATAATTTTTAAATATTTGATTATATATACATATATTCCAAAATTGAGGTTCATCGTGAGATATAAAGAAATAAAAATAAATATAATGTTTAAATCTTGTTGAATTCAATATTTTGCAGCCAATAGAGTCAACTACGTACTAACTTTGGTCTTCATATAAAAGTTAATTAAACATCACTTTTAGTGGGGAATCCTTTGCATAGTGTACATAAATGGACTTCAAATAATTTTTAATTTGTTCGAATCATATATGATTTGGTATTTGTTTTCTTTTCTAATTTCTAAACTTTATAATGTTGTCTTCCAGTTTTTCTCTAAATTCCAAACATTTCAGTAAAATACTACATTGTCTTTTGCCAATTAGATTTTGACACAAATTTATTTTCTAAATCCATGAAGTTAAATATAATTTTCTTTTTAATGCTATGATCTAATACTACACATTGTTGCATTGAAAAAAATAAAATGTTAAACTTGAAAATTTAGAAACTAAACTCTAACTTTAATTAAATATGTATTTATTTATTATACTTTACGTACAAAATTTATAATATGGAAAATGGACTAATTAGGTTTATTTTTCAAAGTTTCTATTAGATTGTTTATTAAGAAGTTGAATAAAGAGCTAACATGAATCTTTCAACATAAAGGAAGCAAAAGAATGATTACTACTTGCATATATGTAAATTAAAGATACATCTTGGATTTTTTTAGGAGAATTATATAAAATAGTCTCCAAAGGAATTTCCTTTTGAAACATTTCTGTTTAGTTATTAATCTTATAAAATTATCAATATTACTCCTACATTAAAATTTCTTTTAGATTATTTTGTATAAATTTCTCAAATTTTGTTAAAAAAATCTACAATGATTAAGTTGATTGTTTTATTGACATAATTGAACAAGTGTAATTCTTATCCTACTTATCACATACATAAAATTTTATACGAGTAAAATCAAATGAATTTATATATAAAAAAATTCATAAGTTCCAAAAATGACAAGATATCACATCTCTTAAACATGTCTACAAAATTCATGGGTTACGTAGCAAATGATATACGTGTAGTAAAAGAAATTTAAGCGAGCCTAACTCAATTCATAAAGTTTCACTAATTTGACTTAATTCAATCTATATAAATTGATAACACAAATTAAATTGTAGATAGATTGAATTTGAGTGTGGGAGTCATCAATTATTTTTCTTTTTTGATTTGGGAAATATTCAACCCTAAAATAAGATTACATATTTATAAAATTTGGTGCCCCTCTTGCCAAGCCTCCTCGACCACCCCTAGACAGAACACTAAACTCTTTAATTAACTTTTCTTCATATTTGACTCTTAATTTGGAAGAATAATAACCTAACAAAAATGCCATACATTAAAAATTATTTGCCACGTCATTGGCTGCCCAAAATGTAAATATTAAAGAAATAAATTCCGTAAACAATAAGTACTACGGTGTTTTGAAGATTACTATATAATATATATATATATAGTACTATACACACATACTATATACTAATATACTGTGAACAATAAAATCAATTAAAGAAGAAAAGAAAACATACCTATCGCTACATGACATAATCAACTAATTTATATTTATTCAACTTCCATTGTAGTCTCTGTTATCCTTACTCTCTCACAAATCTCCATCTCTTTCCTCTTTCCTCTCCATTTCTCTT

**>CsSAUR34**

GTATGAAAAAAATGCTACTACTATATTTCCCCCTATTGAAGGAGTAACAATCTCACATAAAAGTCACATTAAGGGCTATTATGAAAGCAACTCTTCAAAATATTTGTTATTTATTTTTGCATGAATCTATAAGAAAAATATTGTCTAAAAATCAAGAATGGCATCTTTTATAAATACCATCCTTTAATGGCACATTCATTGAATTACAATAATTAATAAATATCACAATTTTTTTTGTTTTTTTTTTTCATCACCCATTTTAAATTTTAGTCTACCTACTCAATCTATCAATCTCATCCCCTATCCAAGTGGCAATCTTTCCTCAAGCCCACATAATTATATGTCATATTGGGAAATAGTTTGTCTAATCAACATGTCAGGTTAGTATTCGAAACGTAAATTGATATATTTAAAAGTTCAAAAACTTGAACACGTCGCACTTTTTTAAATTCAAGGATATGAGGTAGCTACGGATTTGAAGCTCTTTAACTTTATCATCGTAAGTATATTAGTGTTATATGTTATTTGAGTTATGTTCATATTGGTTATTTAATCGTAAAATATATATATTTATCGGATTGTCTAGATTTTCATCTCACCCCTAAAACAATAAACCCTATCAACTTCAAATGCAAGCTCTCTCCACCTACCATAATAATTTTATAAATTCAACTATTTCCACGTTATGGTATAACTTAGAAGATTGTAATACTTTAACTAAACAAAAATATATTTTGGATTGCCTTCCTACCATTTGTTTTATTTGAACTTGATTAAATTAGACAAATTAAATGATCTGTTAGTGCTAAGTCACGATCAATTTACTAGGATTGTAATTGAATGGATCATTAAACAAATTTAAAGGACTAAATAAATACGATTATTGAAGAAATTTAATTTAACAATTAATTTAATTAAATTGTATTTTAACCTTAAAATGAGTAAAATTTGGGTGTGGTTGAGCTGCACAGTCTAAAAACGTGTCCCAACCTTATGGCTACACGTGTCAGCTTTTTTTGTTGGTGTCTTTTTTCTTCTTTTTTTTAATACAAAGCTTTTCTTTCCTTTTTATTCTTTTTTTGTGTGTGTTTTTCATTTTCTAAACTTTTCAATTATAAAAGACAAATGAAAAGGAAAAAAGAAAAACAACATAAAAAATCAAAGTAAATGTCCAAATGGAAAATCTTCTAATACTACAACATAGATTTTGAACTCAAACAAACATCAACCATGATTATAGTTAGTTTTATTTGATTTTAATTAGCCAACACTATCTTCCTATTAACTAAAAAGTAAATAAAAAGAGTGTTTAATATCTCCTATAGTAATAAAAATTTTCTTCAATACCATCATCGATTCGTTGACTCATCTAGTCATTGTAAAATACAATAAATAAATAAATATTAAAAACCCTATTCTAAAATATTAATAATTTATTTTTCTTAATTTTCCATTTTAAAATTTTGCACTAAGAGACATAGCAGTTGAGAAGTGAGTGATGTCATGGAAAATAAATATCAATAATTTAAAATGAAAAAAAAAATACAAAGGAGAGAAATGGAAAAAAAATGTATTAAAAATTAAAATTCCACATGGGGTTGGCTTTATATATAAACCTCTTCTTCCTCCCTCCATTTTCATACAGACGCCATTTTCAATCAATCTAAGAAAATTCCTTCCCCCTTTTCTCTCAATTCTCTTTCATTTTTCTTCGTAAGTCTTCAAGAAATTTTCTTATTTTATTTATTTATTTATTTTTTTCATAATCATCTTCAATTAATTTTCTCCCCAAATTCATCGAATTGGTTTTTCGTTAATCACGATGAGTAACACAATGAGACCTAGGGTTAAGATCTTGTTTTATTTAGTATACGATCGAGGTTAATTTCCATCAAATTCAACAAAATCAAACCCAAATTTCCGTTTAAATCATTCTCGCTAAGTTCCGATCCGAAGGATTTTCGTTTCTGAAATTCGCTAGTTTCGTTTACATTTGATCC

**>CsSAUR35**

ATATATATATATATATATATATATATATATATATATATATATATATATATATATATATATATATATATATATATATATATATATATATATATATATATATATATATATATATATATATATATATATATATATATATATATATATATATATATATATATATATATATATATATATATATATATATATATATATATATATATATATATATATATATATATATATATATATATATATATATATATATATATATATATATATATATATATATAACTTTGTATAAGTCGGAAGTTCTTGGCACATCAAAAGAAAATTAAATTAAAACCCACAAAAAAAAGTATTCTAAAGTCAAATAAGACAAATGCTTAATGCTGGTAACCCTAATATATATAGCTAAGTTCGATCAACGTATATTTCTTCCATCCAAATAGCTAAATATTCAAAATTTGTATGATACCGTATATTTTTTAACTAAAGAAATGGGAGATTAAAAATATTAGTCCTTATCCGTTAATTCACGGTTAAATTTATTCTCAAATTTTAAAATTTTAGTTTTTGAATTTTGATATATAACGTATTAGATTCTTATACTTCAAAATTTAAGGTTACAATTAATTAGTTTCTTAGTTTGTATAACTTTGCTTTTGGTTTCATTACATACAACGAAGAGATGAATTTTTTCACTTACATATTACGAAGATAACGTGTGATTCTCACTAAGTGGAATGATATGTTTTCTACTTTTCTAACATAATATTGTTTTAGTATTAAATTGTTCTAAATTTATTGTTAATATTACGCACACACCAAAGAGCAATATACATATAATGAAGTTGAAGTTAATTAGGTGTGTGCATATATATGAAAATGAGTTCAAATGGTGATGAAAACAAGTAAAGGTGGGGACAATATGAAATAAAGGTTTTAATTGACAACCACACACACATAACAAGTTGAAGGACACATCTTGATATGTGTATGGACATGTTCATGATATGCTATTGGCTGTTCCCCAAATTTGGCCTTACCATGGACATACCTACAAATTTTTTTATATATATGCTTCATAACTTTAGGGGTTGTTCCAATTATTTATTATATATATTTAACTAGCTAGCCTTGTACATAATAATTGGTTTCCATCTGTTTTAGAGTCCTAGTGATCATATACATCTTAATTTTGTGTATTGAATTTTGAGGAAAATGTTTTGACTAAATATGAAATATTAATCTAAAATTCATAATGTATACTTTTTTTTTTTATGAATTTATTTGAATTAGTGAAACCTTTGAACTGTAACTTGGAAAATAATAATTTTTTTTTGATGTATTATATAATAATTCAAAATCTAGTTATAAGAAATAGTGATGAGAAATTATGAATGAAAAATAGGTCCGATGTATTTAAATAATGAATAATTGTTTGATAGTTCTTTTTCAAAACTCATTTCAACCCTTTGTGTGAAAAAAAAGGAAGAAAAAATGTTAGTTTTTTATATTTGAAAAACAATTTTAAATGGTCTCTTACCTAACTTTTTAATTACCAAATAGAACCATTTATTAAATATGCTATTTTGATATTTTTGAGTTTTTAACTTTTGATTAATATCTATAAAATGTGATTTTTATAATTTTGCTATATTTATAAATACTTTTAGTAATTTTGCATTTAAAACTTTTTTTTTTGGAGAATTGACTTAATTACATATTATAAAAATAAAAAAGAAGAAGAAAACTTTCTTGGGACTTTCTCTCTCCGATCCTCTATATATATATTCAAATCCTGCTTGGATTGCTTTACGTTTCAAAGCTTCAAAAAGAATTAAAAGTGATGGCAAAAAACTCACCAACCTCCTCCTCCTCTCCACACAAACACTTCAAAGTTCTTTCAATTTCTCATCCCATTTTTCCCTTTATTTAATTATTTTTTCTCTCTAACTTTCAATTTATAACTTGATCAGAATTCTTCTTCTTTACCAAATTATTCAAAGAAAAAAAAAAAG

**>CsSAUR36**

TCATTTGGACTATTTTTAAATAGAAATAAATAAATAAATATATTTATAAACTATAATAAAATATCACACTTTATTTATATGACCAACGTTAATAGACTATTATTATCTATATATATTTATATCGTGATACACAAATTATAAATTTATCTTGGTCTATTGTGGTCTATCACACTTACATTACGGTATTTTATTATATTTGAAAATATTTTTAAAAGTTTGGTCATTCTTTTATTTATTATTATCCTTTTAAATTCTTATAAGCTTTAATTATAAATGATGTATTTAATTTAAAATATATATGTTCTTAAAAGTGTCACGCTAAAAATTACTAAATTATAAATTTAATTCTTAAATTTGTTGTTATGGTATTTATTTACTTAATACGCTTACCTAGCTATAAGGTTAAATGAAACCTTGCTTAAAGTTTAATATTTGATTATTTTGTCCAACATACATTTTTTTTAAAAAGTCTATTAGCGTTTATAGATTTATATGGTTTATCACTTATATACCACCAACCATATAGGTTATTAATGGCAGACTGTCAAAATAGAAAATGGTGACTATTTGTAAATGATATATATAATAACTACAAGTTTGACAATATATAAGTTACTTAATGGTAGTTAGTTAATTTTTTCCTTTTATAAATTCAAATTATCACCCCTCTGCTCATACTCCAACCCTATACTACCAATAATCTCTTTTAGCATAGTAAAGAGGTGATTTTCTTTTAAATTCAAACAAGTGCAATTAATGTTTTGACTTCGATCGTGAGGATACAATTCTTCTACTAGTTAGATTATGCTTTAAATCTATATAAAAATAACTAAAACGAGTTTAACTCCACTATAATTTGGTATGCTATTTGTTGTGGGTAAAACAAGAAGTTTGATATCATCCCCCAATTATCGTATTAAATAAATCTATAAAAAATTAATAGACCAAAGTTGTCAAGATTTTGGATATTAGCTTGTAGTTAAAGGTAAAAAAAAGATATTGTTTAAAGCATTTTCAAAGTTAAGAATTCTATCACGTACATGATTTATTGACTTGATTACCTTGATTAATGGTTAAGAGATAGATGGTTTCAATCCATATTTATTCCCTATTTAGAATTCAATATCCTATGGAAATTCTTGATACAAAAATGTTATAGGATCAAATATATTGTGGTGTACGATTAGTTGAACATTCGTATAGGTATAAAACTAGTAATGATTTCAATTTAGTTTATGTAGGTTTGAAAATGTTAAAAGTTATATAATTGATATTTAAGTTTTATTTAAATTTGATGTATAGATTTTAAAATAGTATACTTGTAACGTTAAATCTTTATTAAATAATTGATTTAAATAAAATAAAATAAGAAACATCTTGGAAACAGAGGTTAAATTTTTTTAAAAAAAGAAAAAGAAGAAGAAGAAATTGAGAAGCATAAACATATAAGCAATTGGAAGCAGGGAAAGAGAAGGTAAAGGTAAAAGATGGAAAGAGTAAAAAAGAAAAAAAAAAAAAAAGTTAGTAGAAGTATAAGAAAAAGAAAAAGAAAAAGAATAGAAAAAGATAAGAAAAGAAAAACAAAATAAGCAAAGGGATTCCAACCTGAAAACCCAAATATGGTAAGAAAAAGTGGGTTAGCAAAATAAAAAAGAATTAAAAAAGAAAAAAGAAAAAAGAAAAAAAAAAGAGTGGGTAAAGGAAGAAAAAAAGGAGGAGGTTTGATCTGTACCTTCAAAGTACGCTTTACCTGTACCTCTCACAAAGTCTCAACCTTCCTTCCTCCCTCCTCTTCCTTATTTCATCATCATAATCATAAATTATTCATTTTTCCTTTTTTTTCTTTTTTCTTTTTTCCTTTTTTCTTTTCTATTTCAATTCTTTCTATTACAAATTAAAACCAATTTCCCCATTCTCCCACTTTATATTCTCATTCCTTCCCTTCCTCTGTTCCACAAACCCCCAAATTCCTCTGTTTCTCTCCTCATCTCTTTTCCGTT

**>CsSAUR37**

TTGTTTCACTTTATATAACAATTAAATTTACAATTTATTTCCAAAAGAAAAAAATGGAGTTATCTATAGGGTTTTAATATATGAAGAAAAATATAATAATGTGACAGCTATGTTAGATCCCCCCATGCAACTGCATGCATGTGCAAAAGGGTTAGAGAGTTTATTTTTGTTGGCTTTTCTGCCTTTTTGTTGGAATACTTTCACTGATGAAAGAAACATTTGAAAAAGTTTAAAATTTAACAAGCTAGGGCCCCTTAAATTCCCCACATATATATTTCTACATATGCATGTCTCACACCCCAAAAAAAGAAATTAAAAAGATTTTGATACTACCTAAATATTTTTAAATTATATATACACAGTTCACTATTTTTTACTATATTATATTGAAATTTACTCATTAATTCTATATTTGGAGAACACATCTAGTTGCACCTATCTATCTATATATAAATATATATAGTCGAGGCAGCTGTCAAATTAAAAGTGTCATTTGCTCATGTAAAATTAGGACAGTATGTTGTGTTAAGTACTGCAGCCTTGTACTTAAATATTACTCTTCTATATTTATTTATTCATAATATTACAAAATACAAAGGACAAAATAGACATTAAATTTATCATAATGGTGAAGTGGATTTATTTTAAGATCTTTTTTTAGGGTACAACAACTATATATAGATGATATTTTCATAATGTACTGTTTTCATTTTAGCAAAAGGAATTGTTTTTTTCTATATTGTACTCCATATTTCAGTACTACATCTTGTAATTTTATTTTTGAATAATATAATTGGATATATAATTTAATCCCTTTCTTGTTTATATCCACCTTAATTGATTACTTATAAGTTGGATGTTTATATGAGACAGGATGAGATAATGATAGAAATTAGAGGCTAGGGAATGGCTGATCATCACCATATATATATATATATAGTTTTCCCCTTGTACTTTGAATGTGTGTGTGTGTGTTCATGTTTCTAATCAATAATTAAACAAGAAAAAAACTGTTAAATTAGTTAAAGACCAAATCAGATAATAATTGACCCTTATTGGACAGGGAGACAATTAAAAAACATACCACAACACAAGCACAAGCATATAATCAACATTAGTGATCAAATGGGTCATCTCTTTAATTATTTTATATAACAAATGCCTAGTAATGCCTAGTACTAGTACAAAACATTACTAAACATTATGTACCTTCTTTTCAACCATAATTTTTGACAAGGAGAGATGAAAACTCAAGATTCAAAATTCAAAATTCAGATTAGGTATTTTCTTGCATCTCTTACAAGAGTATTGTTACATTATCTTTACTTATCTTAAATAAACTGCTTGTTAAGAGTGAAATATATTATATCTCAACAAGCATAAATCAACAAAGATCTTGTCAATACATAGACAATTGCTTTCATTTCTTTTAAGCTTTTCTTCATGATGTTGTTATATATTTTGGATCCTTGCATATATATAGATATATTTTTTGACATCGATGTTTCAAACCTTACATGTATCCCTAGTGAACTCTTGAAACAATGTTCATTGATTATTCTCAATCTAATCTACGGTTGAGGAGAAAGACCATTTATACATTCAAATGGAGATAGATTATTAATACAAAAATAGATAAAATAAAAGTAAATTGTGAGAGGAAAAATGGATCTTGAATTATTTAGAGACAGACATGACTAACAATTGAGACATTTCATGGAATGCTGAAAGTGACGTACTCACATGCTATGGTCTTCGAACTCAACTCCCAAACTTGTATCTCTGTCCAATCACAATTTTCCTTTTTTGCTCTTTTTTTCCTTTGAGAAATATAAAAATTAAATATCCTATCAACTTCCATTCAATTTCACTATTTTGATCGATCTTTTTAAATTATTTGACCAACCCACAAACACCATAATATAAATTCATTGAACACCCACATAACAAGGACCAAACTTTTTGTAGAATTATTCCACACATTGCCATTAACTTCCAAT

**>CsSAUR38**

CTATGTTCGGACTTTTGCAAGTCACATGTAGAACGTTTATTGGAATATTTTTGCATCCCCCATTTATCATATCATTTGTAGAGTTTAGACCATGAGGGCACACGTAGTTGTACTTTCCTTTCGATACTGAAGCTGACTTCCTTTTCTTTCGATTTGTGATGTCTTCATTCTTGCAACAGGAGATGCGTTTGTTGGGGGGTTCTTATCTCAATTGGTTCAAGAGAAGCCCATTGAAGACTGTGTGAAAGCCGGTTGCTATGCGTCTAATGTGATAATCCAAAGGTCCGGCTGCACATACCCTGAGAAACCCAACTTCAATTAGACAGCTTTGGTATGATGCAGGATTATCAAATCCTTTCCTTCCATGCTAGAGCAGCAGCAATTTTTCACCTCACATCTCCTCTATTACTTTATTATGAAAAGTCTTTATATATTCACTTCTAAATCCATCCATCGAGACTCGGGAGTCGATTCTATTTTTTGAATTGTCCAATTTTTCTCAGAGTATTTCATCTCATGATGGAAAATTTTCATTTGAGCTAGACTCTGAATTTGAATTGATATCGTTTAATCTCGAACCATGTGTATCTCAAGTTATGAAGTTTGTAATTTTGGATGCATCTTTGAACTGATCGGAAGAAATTTTTTGACTCTATAATTTATGCCATGCTCATTTTTCGTAAGGAGTTGATAGGGAAAAGAATTAACCTAAAGGCCTAAAATACAGTGTGGATTTGATCGACTTCATAACTAAAAATATGAGCGACAGCAACTTTAAACTATTTTGTGTTTTGTTAGTTTAGCGGAGGGATTCCAACTTTTGATTTTAAAATTGATAGCAATACAACTGACCCAAAAGGACTTTCTTGTTTTTACTAATTATAAACTACTTTTCAAGCTTGTCTTCTAATGAGAAGTGCTTGTGAAAATATAGAATATGCTTTAATAGTGTCCTAAAAATCCTTAACTATTTAAAAATATTTTAGGCAAAAATAACATTTCAAATGATCTGTGGGTTGATTGAGCTAAGGTTTAAAATTGGGATACTAATTATTTTAAGTTAAAATTGAAATTTCAGAGTATGACTGAGAACAACTAAAACAAGTTAATGGTCAAATTTGATTTTCTCCAATAAAGTTTAGAACCTAAATTGTTTTTATCCTTTCTTGTTATACTCTTAATAAATTCTCATTTTTTGTGTATGAAATTTTAAATCAACAAGCAGTGAGGCAAATGTACCTTAAAAATTTAGGTAGAAAAGTAAGATTCCACACTAACCAAATTAATGCCTTTTAAATAAGTTTAGATCTAGCGAACCAATACCATATGGTCACGAGGCCTTATATTTTTGGTAAAACAAAACACAATGTTGAGTTTACAATATTTGCAAGGAAACGAAATGAACATTGTGTAAAATAAAGAACTTCGCACTAGTCCCCTACTTGACCACCAATACTTTGTCGATCAAGCTAACAATAGTTTAGAGAAAAAGCTAGAGATAAGAGAAGTTAAGATATTATTTTAGTGTTCCTCAAATGACTCGAACCCTTTTCATGATATAGAAAAATGTTTTCGAGATGGTCATTAGGCCTAATTAATTTGCCCGTTACTAATGTTTCTGGGCGTTACAAATGAATCCAAAGACATGTAGTTGGCTTCATAGATTGACTTTGGCTCATAATTTGTCTAGGGTCGACCTCTATACTTGGGGATTATTTTTCTCTCAGGGCAGATCCTATGAATTGATCTCTTAAACTCTAAAAATCGGGTTTGCCACGATACTTAACTAAATAAAACAAATCACTTATTTGAATTGCTTCATCCGATTATCCTTATGGAGTCAAATACTTAACCATATCTTTCACCCACACCACATTTAATTTTCATTGTCACGAGTATGGAGTGAAGCTACACATTTACCAACCAAGAATTCAAAAGAGAGAAAAAAAAAACAAAATATTGGTTACATTTCAAATCCACTAGTGCCATATTCTTTATGC

**>CsSAUR39**

AGAACAAATTAATTTTTTTTTTAATGAAATAACGAACAAAGGAAACGATTGTAGGTTCAACTTGAAGGAAATTATAGTTTAAAGCTCTATATAATTATTTGAAGCTACACTACTAGAAGGATCCTTCTGATCAAACTTAGAACTCTATATCATAGGTCTTATCCACTTTAATAAATTAAGAGTACAAAGGACTGAGTCAAAGTACAAAATTATATTATGAAATGGGACTACATTTTCCTCGTAAGGAGAATATTTTCAGCTTTCATTCTTTATATACGAACAGACTTGAGAAGAAAATTTAAAAAAAGGGTCCAGATACGAGGAACTGATTTCTGAAAGGAACCAAATCTAGACTTCAATATGAAAACAAACAAAAATATATGTAAAATTTATTGAAGTTCAAGAAGAAAGAGGTTTGAAAAACTGAAATAAAAGTTATGTATGAGTGAGAGTGGCCCATCACCTGCACCCAGATGTAATGCTGCAGAGAAGCTTATCCCAAAACAAAACCTTCTGCGGCAGATGAAAGAAAAATGGATGCAGCTGCTTTCTCTGCATAATATTAAGATAAGAATCCCATAGGCCATAAGGTACTGTTTCAAATAAACATAACTGTACGGCTATAGTACACCAAAACCAAATTCTTCATAATAAAATAGATATAAAGATTCTGATGAAGTACTTAACATCTCATGGTGCGCTCAAATCAATCCAAATATGTAAAAAAAAAAAAAAGATAAGTCATAAGAGCAACAATTTAAATATGACTCAAGAGCTATGATGAATAAGACAAGTGATGATGTATTTTTTTTCATAAGATATACACACTTTGCTTTATGCTGATTCTGACGTATCAATATTGTCAAATTAACTACGTTGAAATGGTTATGCAAAAGAATGAAACTTGGCTAAGGCTATTTACTAAGTATGGTATAGGAGCGTGTCTACTTTCTTATGGGGTTTATTTCTAAGCAAATGTATATTATTTTGTAGTTTGTTTCTACTGTAGAATAATAGATCCCACTAATTTTTTAGATTTGAAAGAACAGTGATATCTTTTTTCAAAAAGTTAGTTGCAGTTATTACTATGTAGTACATTGTCATTTCCTTTTTCTATTTCAGACAAAAAAATTTGTAGGAAGTGCTCCCAACACCAAGATAATTTGAATAAACATCAATATCCATAATTCCCTTGTTTTTCAACGAGAAACATAGTTTTTGGGGCCTAAAATCAAAGCAACTACCAAATAAAGATGAGCACTCATAGTGGTCAATAACAATGATCATACAGTTATCCAAAAAGTTATCTACCATGAAAATTCATCTTCCATGAGCAATAATGCATGAAGTTTATCAATAAAAAGTGGCTTAGAAGAGATGAAATCACCTCTATGAGTGTGAACAAATTGATATTCTACAATATCCAAGCTTAAAACTTAAAAGTCATAGAAATAAGTGGGATCGCCACTAAGCCCAAGAAAGTTATCTCCATCCCCTCTTTCAAATCTTATCTCTTGTCCACTCCAATCATGTGGGTCCATTACAAATTTAACTACAAGAACTTTGGGGAAAAAAATCAGTCAACCATTACACCATGCTTTGCCAAAAGAAGAAAGAGATTTCATATGGCAGATCCATCTATCTCTCTCTTCCCCATATGGAAAGTATAATATTTTTCTATATTTGAATATATAAAATCTCTCATCTCAAAACTATGCCATGAATGTTCGTACAATTCGTATCATTTAGAGATGCAATAATATAATAAAGTAAATCGGTACAGCACGGCAAAGGTGCATCTACTATAATATAAAATGCACTGTCAAAGAGAAAATGCCATACAGAAACGAAGCCCACTGCTTAGCTATATCTATCATGTCAACCATTTAATAGTCTTCTCCATCCACTCTTATATATACCTATCAGCTTCTCTTTAATACCATACCTCTCTCTCTATCTCTCTTTCTCTCAAATTCACTAGCTACAGCTACATACTGAAT

**>CsSAUR40**

ATCAGTGAAGCTTAATTTAAGAGGTAGCTATTCATGCATAATTAAACTCTTGTACAACTTCTTTTAAAAAGAAAAAGAAGAACCATTGAGATCCGTCAAATGCAATACCCAATCTGTATTAATGGATACATTTTGGCACTGACAGAGAACACAAATGGGCTCTTCAAATATCAATACAAAAAATACTTTTGAGATAAATGAACTAACAACGAAATAACTAACATGGTTATAGATTTAATTCGTAGTAATTTATGCAATTTATTCCATAATGATGCTTTAGTTATGTCAATGGTAAACAATAGTTCAAAAAAGAAAAAGAAGCTCTAATTACAACAATCAAATCTGACAAAAAAAAGAAAAAAAACTGATGGGTATAATCATGAACATAGGACAAAAAAATCTTTCGAAACTCACAAATTTACTGTTTAATATTCGATAAATGTTGTTGATGATAAGTTTACAAAATGGTAAGGAGTGAAACTTGAATTTAGATTTATAGGAGACTACGAGACATCGTGGACTATACATATATATATAAGGAGCATAATGTTGAGAGAGAATCCTCATAGATCAAGTCGGTGTTTGAAGCCTCAATAATATCAGACATATATTTTCACATTCATTAAATATACAAGAAAATATCTTCCTTCACGAATCGTCAAATGAATGGTTATAGAAACCGGAAAAATTGCAGCACTTAATCCAATTTAAGGTAAAAATTACAATAATATAAAGCAGAATTTGATGAAGTCAAGTTCAAGTAAAGAAAAAAACACACGCATACGACAAGATTAGTTTGATGATTTAGCAGTTCAGAATTTAAAAGAAAACTGAAGGAAGTCCTGAAGTTTCTTTCTAAAACTGCACTTGGCAATTGTACCTTTCCTTACGCGACTAGGCGTCGGCATAGTCCAATATTCAACATCTCTAAGTTAAACCCAAAGACTTAAAAGATATTGCAAGAAAATTTCTTGGTCAACAAAAGGGTGTCGGTTGTTCAAAAGATTGGACTCTTCAAAACCTCCTCCACACTGCATTAAATGCAGTGATGGCAATGGAAAAACCAAGCACTAGCATCTACCTAATTCTTATACAGAAGTTCTAATCATTTAAAAAGATGAAAAGATAACGATTCTAAACATTTTGATCTCATTTATCCGACCTTCCCATACTTGTTAAGGATTTCACAGAACCGGCCACTTTTTTCATTGTTAATTTGTTGTGAGATGAAACTTTGAAGATGTTATGGGATAAAATTGTAATATAATATAATCCAAAACTCATATTTAGATTGAACGTTTTGGACTCGATTTGTAATATATATAAAACCCTTTCCGTTCTAAGGGTTTTCAAATCAATCCTCTTTCTCACTATTTTCACCCTTTATGCTCTCATTCGTTTTGTCTTTGATTACCCATGCTTAATTTTTAATACTATTATGATTACCAATAATCCTGATTACATTCTAGTTCTCCAAACATAATTTTTTTGTTATCTAATAACAAATAGTAGGGCCTATTCACTTTATAAATAAATGAACGTGTGAAACCAAAGATTTAGCAGGAACAATAATTTACAACTTTACTGAGACAGTGTTTAACGTGTTTTTTTGGGTTGAAATTATGGAAGTTACTGAATTTTCTCCTCTTTTTTTTCCACAGATAGAAGACCAACATCTAAGTGATCAAATCTCATCCCAAATTGTCTAGCTTATGTTTAGTAACCTAAGGCTGACTATGATTTTTCTGAGACAGAAAAGTTGTCTGACCTAGTCAGTATTAAATGGTATGTTGATGATACCCACATGGAATGGTTCAGACATGGCCCTTGAAGAAGCTTCAGAGCAATTTCTGATGTACGTCCCTAGCTTCATATAAATTTGTTCCCGGCTCTCATTTGGCTCTATCCGAATCCTCTTGAGTTCGCATAAGCATTTGTTTATCACGCCAAATAGTGGGAACAAAGGGAAATACTGATACAGGTTTCGGCAGTTCGTCCTG

**>CsSAUR41**

TAGAAGCTTCTAATAATACATGTTAATGAATTGAAAGGAAGAAGAATAGGAAGAAGTGGTTCTTGATTTGATAATGTGAAATGTTTAATTTTGGTAAATATAAAAAAGAAAAAGTTAGGAGTCAAGGGAAAGAGGGGGGTTTTGAATGATAAGTGGTTGCCATTATTTCAAACTAAAACGCCTTCTCATATTCTCTCTTTTTAGTGGTTGAGCACCAAAGTCCATTCTTTTATGCCGCTCCCTTTGAATATCTTTCACATCTCATTCTTTTGTCTCTTTTCCATATCTTATCATATATCATATATCATATATATATATATATATACACTTAGTTACTATAATATTCATCTATATGTATATGTATACCTTCCAAAGTTAGGGGTATAATAGTCATCGTGTTAAATAGAATGTCGATGTTTAACTAAACTTATGTCAACTTGTCGTTGCAAATATACCAAAGTCCAAGAAAACAACATGTTCTCACACTAATCAATTGCATCTTTAAATCGACCGTTGTTGGTCCCAAAATTAGTCGGTAGATTATTTAACTTATATTTTCATCTATAAGACAATAATTAAATACAATGGTTGATTATTGAAAGTAGTTTAAGATTTGTTTGGTGGAAATATTGAGTAGAGATAAAAAGTAAAATAAACAGGAAAATATTAGTAAGTAACAATAATGTATTTATAAAAGTTTTTTTAAAAAATTATTGTAAGTGACAAAACTAATAAACATATTTACAAAACATAGTAAAATTTTAGATTGTATAAATGATGACACCAGTAGATATTCAAAAACTTCTATTAGTGTTTATTAATTTTGTTGATCGATAAAATCTAAGATTTTATTATATTTTTGTTATTTTTTCTAGATAAAAAACAGTAAGAGGAAACACACATGGTAAAGAAATTCAAAAGAAAATTTTGATTTATCAAAGAAAATCATCAACTTTGACATTTTAATGTGATACACAACTACTTGTCAAACATATGACGCAAATTAAATGAGTTTTTTCCAAACCATATGTTAAACTATAATTGAAGTCAATGTGTCAATACTTTTATATTTTCCAATATGTTTTTGAAGTTTCATGGTCATCGTTACTATCATTTTGTTTTAGCATTAAAAACCTCTAATATTAATGAAAAGCAGCAGTCAATAGCATAACTTGATAAAGATAAGGTATAGTATATTTTGATTTCTCACATATATTGTCAACCAACATAAAGTGTATGCATTATTGATAATATTTTTTCTTCCTAAATTACAAATCTTTAAAAGTTTAAAATTTAAGCTTAATAAGTTTTTAAAACATCTAAAAATTCTTAAAATTAATAATGGTAAAATCTATTTCTAAAATACAATCCGTTGTCAATGTACTCTTAAAATTTATTATTTAAAATTGGTTAAGATTAGACTTTAAATTTAACTTACAACCGTAGAAGATTGAAGGTTCAATTTTAAAATCTATATAAAAAAAAATAAAATTTGATATTTATAATTAAAATTTTAAAAGTATATTCAACAATTTAGAAAAAATATTTAGATTTGGATTTGTTTAGATTAATGTGAGATTTTTTCTGAAGATTTTGGTACAGTTTAAATTTGGTTTGTACCAAATGTAAACGAATATTCAACGATTTTTCTTCAATTTGATATATGAAAAGGAAAACAAATCTGTAGTAAAATTAGGTTGATAGAGTTTTTTGATTTGATTTGGTTATATTTATGAAAATTAACGAAAAAACTATGCAATTAGAGTTTAATTTACACTATAAATTAAAGCATAGAATTGGAATAATTGAGACTGAATGAGTAACCGTCAAAGTCAATAGTCCAATCTCTCTCTTTTCAAATCCCTTTTATTCCTCTACCCGCCTCCTTCGTTTTTGTATAAATCCCTTTTTCACTTCCTCCATTTTCTCCATCTCCATGGATTCATAGCTTCACCTTTCAAAACCCTAATCGCCTCTCCACTTTCTTCCTTCACAAGAAA

**>CsSAUR42**

CGGTTCCACTTTTATGAAAATAAAATAAATTTACTTCACCAAGTTTATAAGATATTTATTATTTCTTCAAAACGTCAAAGTAAGCTTATTCATATACGGTATCCACCTATTTTACATGTCACAACATTATTTGTATTGGTGTCATTAAATTTTTATCAATATATCGATGTATTTACATTTTTATGAATTTGATGCAGATATATTAAGATGTGAAACAACATTTTCATCGATCTCAATACTTTAAATCGATTTTTTTTATATAAGTAAATTTAATGATTAGAATAGTGTGATATGTTTGTAGATGAGTTTTTTCGTCATGAAAAATGAAAATTAACTATTTAGATGGGGATTTCTTCATGAAATTAAATATTAATTTATTTTTTAAAATATATTGAGAGAAAAAAACTATGAAATTTAAGAGGGACCAAAAATTTATAAGATCGAGGCAAACCTCACTCTACCACTTTGTGGGGCATTCATAAATACACACTTAACACATATTTGAAACTGATTTTAAAATAGTACAAATCATTTTGGTTGAGTTCATAAGTATTTTAAACAACACACTTAAAATTATTTTAATAATTCTTATATTATAAAATGTAAGTTTATGAATGATTAAAAACTTGTTTTAAGGTAATTTTAAATACTTTGAGAAAATTTGTAGATAAAAAAGTAACTAAAAATCAAATTTACTTCAGAGAAATTTGTGAACTAAAAGGGTAATAACTCTCTATTTTCTTTAATTACAATTCAAATATAGGAAAGAGGATCCAAATTTGTCATCTACAACTTATTGTTCCAATTAAACTTAAACTAATTGCAAAGTCAATATAATCTAATTGACTTAACACTATGTACTTTTTATTGAAAGGTTGAAAGTTTGAATCCCTAACCATTACGTAAGTTATCATTATCAAATTTGAAATAGTGATGGTCCAAATCTCCTATTTTGTAGTAGCTAGAGTTGAATTATAACTCCCAAGTTAGGAGAGTACCAAATGAGTCGAGATCACATATAAATGAAAGTGATTCCAATGATATATATGAAAGTACGATTATGAAACTTCATGCTTAATTTGAAGCAATTTTACGTTGAGTGACCTCTAAAAATAGTTTACTAAAGAACATATGAGTGAACACAAAATATCATAAAATGATTGTTCATGGTTGTTTATGGAAATATAATCGTTCCTATTAGAAGTAGTGAAGTTCACAATCTGACTTTAAATATATATTCAAATGAATGAAACTTTTTGTTTAGTTTGCTCTAAACTGGAAAGAAACCAAAAATAAATTGATAAAACCGTTTAATGATTTAATGAATCAACATTTTCTAAATTTAAAGATCCAATTAGTAATAAACACTTAATTTTGTGTGAGCTAAGCTGATGAATTTTAAGAAACATTGAGAAATTGGTAGACACAAACTTGTTAGATGTAAAATCAAGTCTTTATTATTTATCAACCTTATTATTTATATGTTTAAATTTTGTATTTTTATACTTGAAAAATAGGATTATTATTTAAACAATTTTTTTTGTTAGAAAATTAGTAAGAAGAACTAAGCATAATTAAAATGGAATACATAAACTGAAATTTAGGACAAAATGATATAGTTTAATCTAAAAATAAAATACGTTAGAGAATAATAATAAAGAGAAAGTTTAAGAAAATGGAAAGAGAAAATGAAAATAAAATAAGAAAAGAGAGAGAGAGAGATCAGTGAACTAATTGCACGTTAAATCATTTTGTCTAATGAAGTTTACACCTTCACATGGGATTGACTACAAAATTAAGAACATACCGATCTCGTAATTATCTCCAACTTCATACCCTTTTTCATTTCTCCATCTCAAATCAACAATTCCCTCTCCATATATATATAGTGTATGTGCACACACACATACATGAATAATTAAACCCCCAAGTACTCCAACAAGAACCAAATCAAAACTTCCCAACGTTTTCCCCAGAAAAATTTTCAAGTTCCAGCAAAA

**>CsSAUR43**

CCCTACTAAGATCAATTATTTAATAATTAAATTCACATAAATTTTTAGGCAGAAATGATATTCAAAATAAGGGTTCGGGTTCCCATATTGAAACACAAGTGTTTTTCTCCATTTTAATACCACTACTTATTTTGAAAAGAGAAAGAAGAATAAAGACTAATACTTCATGAGATAAAAGAAGGATAAATGAAGTCCATATATATAGACTACAATAATTACCCAACTTGCAACAATGTGATGTTTGATCAATATCTTTTGTATGCCTTTTCTTCTTCTTCTTCTTCTTTAGCAATTAAAAAAGCCACTTTTAACTTCAATAATAATCATGGAATTAAAAGAGGGTTGAATTTCTATTTTTTTTATTATTATTTATATATATTTGTTTAGTAAATAAGAGTGTAGAGTGAGATAGACATAAAATATGGATAGTGAAAAAGAAGAAAAGAATAATTGAAAAGAAACAAATGGGTAACATGGGTGAAGATGTTGAATTAATGATAGTTTGAAAAGGAAAAGAGAGAAGGTATACTTTTGTCTTTTCAACACATAAATTGATAAATATAAATAAAATCCATTTAGCCTTACAAACTTTGAAGTTTTCTTTGGCCCTACTTTCTTTTAATAACTCACACTCTCTCAATACCCCACTTCTTCTTCAATCAAATGAATCACCCAACAATCTTTTCTTTTCTAATATGTTCTCCACTAATTTTACTTTACAACATGTATTTTTACAGTAAAGAAAGAAATGTAGAATATGATTTCAATTTTGATTGTTTTAGTGAATTCATCGTCATATCCAACTATGTTTGATTTAAACTTAAGCTTTAAGCTTTATTTGTGCAGCTGAATTTTAAATTATGAGTTCTAATAAAGTTCGTGGGATTGTTTATTTGGAGACATGTTAAATGTTGAGGAATAATTAAAAGAGGAAGAGAGAAGAGTAATTTGGAAGGCAATCTACAATTGGTTGTTATAATTTAGTACATTATCGACAATGATCCGTGCATTATTTTAGGTTATACTTTTATTAAATCGTGTCAACATCACAATTTCTTTTTTACTTTTTTGTAATCTTTTGTTGTGTGTGATTAGAGCGAGATGCTCAACCATTTATACACATTTTGAGATGCAATGATAAAAGCTCACAAAAGTAGAAAAAGATGTTTCCTTACCTTTTTCATTTAGAATATTCATTAATACAACCTAATAATGGACCAAAATTAAAATATCATCACAAAAAGACTTAAATAAAAAATATACAAAACATTTATGGAATATATTTACGAATTTTGTTTGAAAATATATCAACTTTTTTTTTTTTTTTTTTGTCAAAAAGTTGATAGTTCTCAATATTACACAACACATCTTCGTTCAAACGTAAAAAAAAACACTGACACATTACTAATATAACATCCAAATAATAAAATATTTATTTACTATTTTATAGAGTTATTTTCTTCAAATATAATAAAATGATATAACTTATTTATAATATAATAAAAATTTATTGTCAAATAACAATTGAAATTGATAGACATTTATGAAGATTGTGACATATAGTTACTATAGTTGAAAATTATTTAAACCATTACTCCAATTTAAATATATTTAATATTTTATATAATTTGACTAAAAATATTGATGAACATTTTGATAGAAATTAAAAATGATAGTTCCATCAACAACCTAATTATTAAAAAAAAAATGACGTTAGAAATAAAAATAAAACTAAAGAAAAAGAAAATATCAAGAGAAGTAATTAAAGAAGAAGCAGAGCCACAGAGAAGAGAAAAAAGAAAAAACAGAGGGGGTCCCCATTTGTCATTTGGAGGTCCAACAATGGCGACTTCACCCCATTGCCATGGCTCCCACCTTCTTCTTCTTCTTCTTTCTCTCTCTCTCTCTCTCTTAGTACAATTTCCGTGCTCTCTCCATAAATACCCAACTCTATATTCCCAATCCCTTTCCAAATCGGACCCGAAAAACCTCCCCCAAAA

**>CsSAUR44**

ATTGTTCAACTCGAGATATATGAACTGTCCAAAGTATTGAGCCTACCACTTCAGTTCAGTGTTAAAAAACAAAATAATATGTTTCACCATTCTTTGTAGAACATTCATTGAATGAGATAGAAGTCAAGACTCTCGGTGCTAACAAGAGAACACTTTCTTCCTTTTTCTCCTAATTTTTTATTATTTAGTTCTCTTCGTTTTTTTATGAGAAATCACAACTTTTATTGAGAAAAAATGAAAGAATACAACGGCGTACAAGAAAAAACCAGCCCACAAAAACACCCCAATTAAAGGAAGGGGGACCTACTGAGAAGGATATCACCTATTTAATTCTCTTCATGATCCAATGTAAAGACATTGCCATAGCTTAAGTGCTGTTCAAAGATTATTTTACTTAAAATAAAATTTTGAGCATCATAGAGAGATCAGCACATTCTCCATTTTACATGGAGGCAAAACTTCTATGGCTTTTTTTTCCCATCCTTTTATGACCTAATTGGGGAGATCAAAAGCGTTTGTTCATACAGAGTAGGCATATGTGTATTCCTGTGCAATCCTATACTAACAAGAACATCTAATTTGAATATGTGGCAGTAAAAATCCAACAAGTAAAAGCCCATTCTTAAGGCCCATTACCCATTGAAAATTGTATAGTGGTATTTACTCTTTTTCTTTTTGCTTTTTCCTTGTATATTCCCCATCCCCTCTATCTTTAATTTATTATTGTAGACAGCTGGCTATTTGTATGGTATGGTATGGTACAATATTGTATTGTTGAGTAGAAGTAAGAAAAGTAAGTCTGCTTTCTAATAAAATCCTACAAATACAATAACAAAGACTTCTAAATTGAATATAAGACCAAAGCCTTTTCCTTCTAATGTAATTATAAGCCAAAAACTGAGTTCAACTGTTCCTAAATTCAAAGCTTAGGCCTTTCATTCATGATCCGGACACTAATAGCTTGAAGTTATTCATTAACAATCTAATCCGAATGTTGTGAGGAACTTTCCTCTGGCTGTACAACTAAATTGTTTTAAATTAGTAGCAAGAATACTAAATCGAAGGAAAAACAAACGCCCTCATTCATATGAACAGAAACACTGATTACATATGCATCCATTAAAGTCAACAATAAAAGCAAAGTTACCTCTTTCCCTTTTCTCGGGTAAATCTTTCCACAATTCTCATGAGGTTCTGACATCAGCAAAGCCATCTTTTTGAGTTTCCTAACATGGAAGTTAGAAAAAAAAAATCCTTGCAATCCAGACACAGTCAAGTTGAACCATCAGAAAGTAGTGGCTTATATTTCATTGTCTTGTAATGTTTTCATTCAATCCATCAAAAACAACGCAATACAAAATGAGGTAACCTTTCCCATTTTCATCTTGTTGTTTTCCCCATTTCCCCTCTTCTTGTTTCCATGGATTATCCTCCCCTATTAATTGCTGAATGAGCCAAATCCACTCCCTTCTTCTCCTGCTTTCTTGTCAAATTAGACACCACATTTTGCCTGAGTTTCTTCTTTGCCTCTGTTTCCATCTACCCTTAGGGTCTACATCCATACCAAACTCTCCACAGACCGCCCTCATTGTTGAATATACCAAACACCGGTGTCCCGACCATGTCCTTAATCAAAAGGCCAGGTATCAAACTGTCCATTTCTAATCTTTAGGCAGCTCTCTATCGCTATTTTCTTGTGTTTGTAGGACATACCAATGTTTGTCACCTAATAGACACTATGATGTTTGCAGTCTCAAATCTTAGCTGCAAGTTGGAAGACAATTCCATGTGCTCTTCATAATCCATATCCCAACTCCATTTGCCTCTTCACCACTCGAAAGACTTCAAGAAAGAAGAACAAAGCAATTCTCACCTATATATACACACACTAATTCTCTTCTCTAAATCACAAACCCAGATCTTTATTCAGGGCTGAAAATCACCCACTAAAGCCAAAAATCAAAACAACCCACAAAATCAAAATCCTCAAGATACATACC

**>CsSAUR45**

TCATTACTAAAGCTAGATGATGATACAACTTGTTGGAAAATTCTTTTTTCATTCCTCTTACCCCTCACATTTGTTCTCTGTTCAGTTTAAAAGTGGAAAAGAGGTCAACAAAGGCATCCAAGCTTTTCCTATTCATATTAAATGGTTTCTGAAGTATCTCCGATGGCAAACATTTTGGGGACAATATTTTACTGACCAAAAATTTTCAGCCAGATGGTTCAATTTAACAAATTTACCGTATGCTTAGATTTGTGGGGCAAGCTAATTTCTAAATTGAAACTGATCTTTCAATGAATTTATACTAATTTAAGCTCTTCCAGAATTTTAACTCAAGCTGTTATTATTTAAGAGATCATTTGCACTAAATATCCAGGTACTGTAATGTCTTATCAAGAAGCTGTACTGATTTCAGATTAAGCACCTGACTCAAATGAGGTTAGCACTCATAAATTTACAATAGCATTAGTGGGATCCATGTAGGTTTCTTTAACAATTAATGGCAGCTGAAATTGTTCACCTACCAGCATACACTACTAATGAAAACCATCCTATTGATGATAAGTCAGAAAACAGAAGCATGTGCTATACATAAATAATAAAAATAGAATGCAACATTTTTCCCTAGCTGTATTTGAAGTGGGGTCCCTTTTAGGATGTTAATTTTGGAATGCCTGTACAGTAGATACTATGTTTGCCAACAAAAATACATTCACAACCACTGTTCAACGTTCATCAATTTGACTACAAATTCATGTAGTAACCTCTTGTCATATGTTTCCCCTGTTCACCTGCAGTGGAATCCACATTTGTAGATTTTGTAGAATCATATTATATGATTCTATATTTCTATGGAATCTTTCGGTGTAAAGTTTAAATTTTCTTGCACAGAATTGAAGATCAAGAGATGCAAATATACCCTGTCATCATGTCGAAGATATCTTTTGCCAAAGCTTCAAATGTTGTTGAATCTTCTTTCCTGTCCAATCATGTTCCAAATGGGTAAATTCCAGTCTATGTTGGAGAGAAGCAAAAAAAGCGGTATTACTCGAATTAGCTGTTGTAACAAAAGTTGTTTGATAAGGCAGAGAAAGGAATTTGGATATGATCGTTCAATGGGAGGCTGCACAATTCTCAGCAAAGATAGTGTCTATGTTAAACGATATAACATTAAATGTCTTTAACTATTAGCTTAAAACTTTTGGGTCTTGAGTGAGATAATGTAGATATGATGGCTCAAGAAGATACAGGTGTGACAGAAAGCAAAGCCTTCCACATTAGAAATTATCCTTCACCAAGTAATCAATACTCCAAATGTCCTGCAATTTGATGTCATACAAATGAAAACATAGCTAGTCAAGTAGAATAGTTAAAAAATTATGGCTATTCAATTCTCTTGTTATGAATGCTTGTTTCTTAGATTCAAACAAATGCTAACAATGGAATCTCTGTTCTATTTCTATCCTGTACTATTTCACATTCTTTGTACCTCCAAGTTTCACCTGTACCGCTATATCCGTGCTTAAGAATTTTATGAATTCTCTACTAACACCAACAACCAACAAATTCTCTTAGCTGGAATCATTCTAATGGAAGAAAAATGGCAGACATAACTATTTAATGCAAATAGAATGGATTCACCACACGGACAACAAAGGCTTGAGGTGCTTCTAGCATGTACATGATGATGATGCTACTAAACTTATCGCAATTTATTCAACTCTGCTTGCTCATTTTCATCTAACTGGCCATTTATCACATGAATATTAAATCGTCATAATGGCTTGGAAAACACAACATGTGATAGGTATGAGCTAATATCTATTTAGCATTTAATCTTATCTTCCTTCTTCTCCAATGTCACTATCCTTCATCTATATAAAGATCATGAGGTTGAGCTCACAAATCAACTCATCTTCCACGTTTCTATTATCAAAAGAAATTTCCAAATTTTGTGTTCCTTCCAAGGTCTCACAGTTCTTTTCGGAAAATACTGTAATACC

**>CsSAUR46**

ATTGTTCAAGCTAAGCAAAGTCTTCGACGTTCTTCATCAACTGGAAATGGAACAACGGCGGTTGATGTTCCAAAGGGGTACTTCACAGTGTATGTTGGTGACGTACAAAAGAAGCGTTTCGTCATTCCTTTATCTTACTTGAACGAGCCTACTTTTCAAGATTTATTGAATCAAGCAGAAGAAGAATTTGGATATGATCATCCAATGGGTGGCATCACAATATCTTGCAGTGAAGAACTTTTCCTTGGTCTCACTCAAAGTTCGAAACACTTGTGAACTAGAGGAATGCAAACTAGAAATTGGTAGTATAGAAAAAGGATTAGAATAGTACAGAATGTAGACTGCATACTGTATATATTTTTGAAATGAGAGAACTTGATTCTTTGAATTGAAACTTTTGTTCAGTGATTCTTATTCTTTATATTATCAGCACTTAATGATGAAACTAATTGAATGATGCTATTGGATAAAAAGACTTGCATAAACTGGATCGTATTTCTTGAAATGGCTACTTGCAGTCACACAATGTTTTTTGCTTATTCATTTCCTCTAATTCAAATTATTCATCAAGTGATAAATACATAATTTATCTTTTCTTCTATTCTTTTATGATTTGATTTAATATTGTGATTCCAAATATAATGCAAACGCTTAACTTTCCAGCTCGTGTTTAAAAATTTCCTGTAATTTAAAAACGGTCTATTAAGCCCTCGAAATTTTAAAATTGTGCCTAATATGGCTAAATAAACATAAATTTCAAAGTTTACTGATCTATTAGATATTTTAAAAAGTTCATAAACCAAGAAGAGACAAACACCAACGTTGAAGAACTAAACTTGTAACTCAACCAAAAATCAATTAGATTTGAATCATTGAATTTTTATAGACTAATCACTATGACTAGCATAGTATTCAGGATCTTTAATCAAGAGGCTGGCACTGTTATTGGATTATGCATGGACTAGATTCAACTGAGGCTAATACTCATAAATCTACAAATTACACTGCCAATGAGAACAATGTTATTGATGATAAGTCAAAAAACAAAACCATGTGCCATCCATTAATGATTATAACAGATTGCCACATTTTTCCATAGCTTAATTTGAAACTGGGGTCCTTTCAATTGATAGAGTGTAAAGGATGTACATTTATATTATAATCATAGAGGAATATTGTGCACATATTCATTAGACCATTACCGTTCTCCTTTTCTAAACATGGTTTCTTTTTCTTTGATTGTTAGGTTGTTAGGGTGTTTATATAATATTAAATTTACCTACGTCTAAGTTTAAGCTTTTGGGACAATCGGTGATTTAACATTTTTAAATCTCTGCAATATTATTTCCTCCCCAATTAATATATAGGTTCGGGGTTTAACAAAGGCAAATACTCATAACCAACCTCAAATAAGAACAAAAAAAACTGATATGCCTCGCTTTTGGTTCGCTCTATTTTCTCACCCAAAGAAAAAAAGCCAGGTTAAGTAGAACACAGTATCCATGCTAAAGATTTTATGAGTTCTTTGTTAACACTAAAAGCCAACAAAGCTTCTTACTCAGACAGCTAAGTAATGCAATTGAACTTTATTTTACCAGAAGGACAAGAAGGGCTTGAATGCCTCAAATATGTCTATAATAATATTAAAATTGTAGAGGCTTATTCAACTTCTCTAGTTGTTATCTATAAACAAAAGTAGGCCATGATGTTTCTTCACAAGGTATAGAGAATGGCCATATATCACATGAATTTGAAATACTCATAAAGGTGTGGAAGACACATCCATGTGGTAGGTAATGAGTTAATGTCACTCTAGCATTGAATATCTTCTTGCCTTAACTAGACCTTTGAGTAACCTAAGCCTTTCATCTATATAAAGGTCATGTTATATGGATTAACAAGCAACAAGAGCCTTTCCATATTCGATATCTCGAAAGAAATTTAGAAGTTTGGTATTCCTTTCAAGGTCTCATATTTTAAATCTTGGAACATATTACAAC

**>CsSAUR47**

AATAAAACACAAATGCTACCATATAAATAGGAAAACCAACAGCCTAACTACTCATAATAATTTTTACTAACCCATTTCCTTTCTCTCTTTTTTCCCTCTTATTAAACTTAATCATCAAGAAAAGAATTCAAGTTTCACCTTCAATCTGTATGTGATGACTTTATTGACCTTATGATTTTCAAACTACTTTTTAGTGAAAGCAATGATAATGATGTAGTATTCAATCAAGATTTTGAATTTTGGATCTCAAAGTTTTGACTTTATAATCATATCCTCTGTCATGATATGGAGTGGGAACTCATTTAGGAATTTCTCAAACTGAAGTTTCAAGAAATGGATATTGATTAGTTTGGGCACAGTCAAGTTCTAAAGTGACTGGTTAATATTAGTGTCTTGATGTCTAGTCAGCAATTTTGAACCGCCAGTGGACTAACAGAAACAATTCTATGTGTGATTTTTGTAGATTGTATACCACGAAAAGGTAGCGGCTGGATTTGTACACCAACCGATGTACATCAATAAACAGAACCAGTTCTTGATGATAAGTCAGCAAACTGAGCCATTGTGATATACTAATTCCCTAGCTTAGACATCATCTTTATTTGATCAGAGCATAATTTACTTAAAGCAGCATGTTATAAAATCTGCATAGCAAAAAGGAGATATATAGATAATTTTTTTAAAACTATATAATCAGTATTGGCAAAGCCTCATTGGAGCAGTACAAGTTTGAAAGCTTTTCTTCAATAGTAATAGACATTTGTGCCTATGAAATTGTATTGTTATAACATTGACAAATAAAAAAAGCACACAAAATGTAAACAATAAAGATTGACAAAGAAAAAATTGCATGATTCACTAATAGTGTATTAGCATATCCACAGGCAGAGAGGTAGATCAATTTATTATTAAAGAGAAATATAAGATAATATAGATTACAGAGGTACCTCCAGGGTTAGATGGTTTATATAAAATGCAACTCTCTAAATTCTAAGGTCAAACTCGAACATACTGAGAGCCTAATGTTTGGTTGTTTGGAGCGTCAGATAACAAATTCGAAAACAATAATTTTGAAAATAGTCTTTCACTATTTTACTTCAATAATGACCTCACTTTTCCCTTTCTGTCTTTTGTCAGCTTCGTGTGAAAAACTATGCAGATCCATCACAATGAAGGCCCTTTCTTGTAGTATCACCACAATATAAGTTATTCTCTTTTAAGTTGCAAAATGCAGCTTCATCACTATTTTAAATTGCCAAATGTAGGTTCTACTCCTTTGCCAATTGTCAGGCAGAGATATATCCCCATTGACTTCACATAAAACAAGCAAAGCCAATTCAATGAGAAAATAGGCACTCGAGACAAGTCGAATAAAAAGTTAATAGTATCTATTCAATTGGTCTTGCTATGGCTAATGTTTTATCATCCAATAGAGTGCTAATAATGGGCTCTCCTCGCTACTTCCATCCTGTACCTCTATATGTTTCAACAGTACCATCCACGCTAAAGATTTTTTAGAGTTCTCTACTAACACTAACAACCAACAAAGTTCCCTAGCTAGAATATGTCCCAGTAAAGAAAACAGTAATAGACAGAACTATTAAATGCAATTAAAATTTTGTTTTAGCAGGACAAGAAGGGTTTTGAGTGCCTCTAGTATGTCCATAATCCAATTGAAAATTTAGAGGTTTACTCAACTCACTTCTCCGGTTGTTCTTTGGAAGCCATAGTAGGCCATGATGTTCTCTATAAAGTATACAGATGTCACCATATATCACATGAATTTGAAAATCTCATAAAGGCTTGGAAGGCCCACCCATGTGATAGGTCATAAACAAATCTCTCTCTATCTTTCTCCCTTTTCAATGTGATCTTCCTCTATATAAAGGTTATGTAATTGGGTTCACAATCAACAAGAGTCTTCCATATTCCAAAAGAAAATTCCAAATTTTGTATTTATCTCAAAGTCTCACTAGTTCTTTCTCAAAAAATACTATAAC

**>CsSAUR48**

AAACAAGGGGAAAAGTAAGAAAAAAGATAATAGAAACAAAGAAATAATTATAAAACAAGTTTTTATCGTTATTTCTTCTTTTCAAGAAACAAGATACGTAAATAGTTATAAAACATATTATTGTTTTTAGATTTTGAAAAACAAAATACAAGATACAAGACACAAGAAACAAAAAACGGGAATGTTATCAAACGAAGTCTAGGTATGTGCGCAAGTATTCTTTTTCTTCATTTGATTTCTTGGTAGAGGCTCTTTTATTTTGTTATAACTTCTGCTTAATATTTGAGCCATTCACCATCCATTTAATATGTATTGCATTGGATTAAGTCATATTTAACTCTATACTTGTTGTTTATTTTAAAAAATGTTTTTGTACAATCCAAGTGATGATTGTATATTCATAATATTTAGAAGTTCGAGATTTATTGTGAAGATTGGGGTCAATTTTATTTAGAAGACTGACTCGGACACTCACATATATCAAAAAAAGAAAAGAAAAAGATTGGGGTTTTCTTTTCTCCATTTTATTATGAAGTTTGGTTTCTGGTTTAATACAACTGGGTTCTTTTCAGTTGCATCATTGTGGTTCTTTCTTAACAACATACAGAATGTTGAAGAAAATAACCTCTCAAAGGAAGAAAAGGAAAAGAAACAAATAACAATCAAAACAGCTGACCAACAAAAAGAAAGGCCAAAACAGAAGCCACGAAGCAAGACTTACACCATAAAGATCCCAACAAAATAATAATTAGTGTAACCAATAAATGATGATTTGCATTTGTTGTGAACTGAAACTGAATAAGATGAATTGAAAGGTATAAAAAACACAAATGAAGATGTCAAAAGGAAAGATGCCTTTGAGGAACAGGTAAACATGTCTTTGAGTTGGTACAATATTCAAGAGGGAATTTTTTTTCTTCCTAAGAATGCCACTAGCCCCCCAGAATTCTTTCGTTCTTTCTTAACAAAAGTTGTTGATTCTATTAAAAAAAAATAGTAAACAATAAATAGCAGTGGGACAACTTGGTTGCCAAGATCGAGCTACTGGTTCAATTAAGAATTTTCCATGGCAAGCTTCTTCCTTAGGTCGATCATTTCAAATCAGCTCCTTTGAAGGCTTCATCATGGTGTTCTTTATCCAAGTTGATCATGAGATTTTCCCGTCAGAATATTTATATTAATTGGGGTACTTTCATAGATCTTTCTTCTTAGAATTTTTTTTTTAACCTATTATTTATAGTGTCTTATTGTTATTGCGTTTGTTATCAGTATTATTCTCTTCGTTACATCATTGAATATTTACGTTTCTTGCTCAGAAAAAGAAATGTTCTCTTCATAATGAATGCTAAACTAACAATTGGGTTTCTTTTTCTTTGTCATCTTGTACTTGAAGTATTTTCTTCCACTTACAAGTTTCTGTTGTACTACGGTACCCGTACATATGAATTTTCCGAGCTTTACAACCGACAAAAAACCCACTTTAGTTAAAATTCTTATACCACGAAAAATTGTGACAGACATGGCTATATGATGCAGTTAAATTTTCTGTCAACATATAGTCTGTCAATTGTTATCTCACCAAGAGGACAACAAAAACTTCAGTGCCTCAAATATGTCTATGAATCTACTGGAACTTGCTTAGTTGTATTGAATTCTCTTCTCAATATTGTCCATAAACAAAAGTAGGCCACCTATGATGTTTCTACGCAAGGATCCAAAATGGCCCCACGTTAACTCATGAACTTGAAGTTCTCATTATGGCTTCAAAGACAAATCCATGTGATTGTTTTTGAGCTGGTTCCTTTCTAGCATTTAATGCTACCTTCTTGCGTTGTCAATATTTCTATCTCCAATAAATCTCACCCTCCATTTATATAAAGGATCATGTAATTGGGTTCACAAACAGCAAGCCTTCCACATTCCCACATCACAAGAAATCTTCTTTCACAATCCTTCAAGTTAAAGTTCCACTAATTCCTTGAAAAGAAAAATACTATACC

**>CsSAUR49**

CAAAAGTTGCCAATAGTTAATCATCATTGAAAATGTTTATTGCAGGACGTAATGAGCGACCCACAATTGATGATGATTTCAGAAAGTTCCAAGAGCCCGTCTAGTATATCATCACAGGTAGAAGAAGAATGAGGTAAGAGTTATTTGTTAACGCTCCAAAGGAGCAGATAAATTTGTAAATGGGATTATGACCAGTAGTAGGATTGGTGTAAAATACCATAGTACTTGAACAAATCTATCACCACCTCTTGAGTTAACCAAAGTGGATACATATAGTACATAAAAGTAAATATTGATTATAAAGTTGATACCTATGTACCTTATTTTGCCACTTTTCGTAGTCCATTCTCCTTGACAACATGATTACACTACATCTGCCCTATGATGTTCCTTCAAATGATTTGTATTCACAAGGAGATTGAGAGCTGTTTTCATATATGAGATTCATTGTACTTTGCCTCCATTGAAAAATTACTGAAAAAAAATGATCGGTGTTTTAATACTGCCAACTTCAACAGAAAGTTCTTTTTTTTTCTTCTTACAAAAGCAAGAGAGCTGACTGATTAAAATCAATACAAAACTTTAACTAACTAGTAAAAATGAGTATTCAAATCACTCAGACTAACACTGCGAAAATATATGAACGTTATGAGACTTACTGATAGAGCAATTAGCAATAAAGGGAACTGAACAGGTCAGGGCTTTGAAGGAAAATATATATGGAAGGAGAAAGCAGGTTAGTAAAATGAGATGTTTGAAATGACTTTCTATGTGCATAAAAAATGGAGTCTAAGTGTTACAAAAGTGGTTTAAGCACTTGGAAAGTCATTTCAAATAGGTCCAACATCAACTGGGCAGATCTTGTCAATTTTTTGATTTGATGTCTCAATTTGTTGGTTTGTTTAGTTTTAGTTTAGTTCTTCTCTTAAAAAATGGGGCTCTCTTTCAAAGTTTTTGGTGCTTCTTAGTAGTAGGGTTTTTGTCTTATTTGACTTGTACTCTTCATCAGTTGTATTTCTCTTCTATTTATCTTTTCAATTGTTTTGTGTTTTGGAGCGTTAGTCTATTTTCAGCTTGGGCAAATGTTGGAAGATGACAAACCACAAACTTTTTCACTGGAGAGAATCATGCTTCAAGAATCAGTAGTAGATTTCATTAACAAAGAGTGTCGTAATATCTGTTGGTAAGAAACCAAGTTTTCATTGAGAGAAAAAGAGCATACGAAAAAACAAGCCTACACTCCAATCTAACAAAATCAAACCAAGTTCATAGTTACAAAAAGGCCTAGCGAGTGTCCACAAGGAGGCGTTAAATCTTGCTACCTCCGAAACTTTATTGCATGACCTCTCCACCTCCCTTAAAATTCTATTGTTTCTCTTGAGCCAAACTTCCCACGAAATAGCTAGCTTGCCACAAAAATTTGTCCTCTCGAAATGACAAACCTTGGAGCACCTCCTCAATACTATTATAACCTTAGCGATTCCAAGCCAAAGTTAAACCAAAATTCTACATCCAACGAACAAAAGCATATCACAACTGTTGTCTGGATGATATGAGAAACTACTAAAGCTTATGGTATATAAAAATCATCATGCAATGGTAAGCCACGGTAAGCCAGCTGTTTACAATAATCAAGTTATTAAACTAACATATTTTCATCAGTCATTGTCATTTAAGAGACAATATGATCTCTAAATCCTTTGCCTTGAAAAGATTACACAATATCCTCTACCAACATCACTACTTCAATGTTCATTATTTGGTATACAAAATCATATGATACTTCATGAGTTCTTGTCTGCTACTAAGCCTTATCCAAAGACTTCTATCATCAATTTCTCAGAAGGTTGGATTTCCCACAACTCTTTTCACCATCTATAAATACATATTTGATTGAGTTCACAAGCAACTCAACTCTTCATTTTTTTTTCATTCAAGGTGTTTTGAGATCTTCTAGTAAAATTTTTTAGGTCTTGCCTTTATTGTATCCAAATATAAAC

**>CsSAUR50**

TTTGTAGAAAGACAATGCATTTACAAACATCAATTTATTGATAATGGTAGTTGGAACTTGGAAGACAAAGCATGTTATACCTCTTTAAGTTCATGTCCCCTAGTAGGCCCTATCCAATAGCTATGAAAATATACCTTCATGTGATCATTTATTTATAAATACATATGTGAGATGGCTTAGACGAAGCAATCACAATTCTTCACATTCCCATTTGAAAGAAACTTTCTTCTCCTGAAAACTTGTGTTCTTACAATCTTCTCATTACTCTAGAAGTACATCAATTCTATTGTACAACATGGGGATTCGTCAACTTCTTAAAAGAAACCAAGGAGTTTCAACAATTCCCAAAGGTTATTGTGCAGTCTATGTTGGAGAGAACCAAAAGAAACGGTTTGTGATCCCAATTACACACTTGAATCAGTCATGCTTTCAAGAGTTGCTTTCTCAAACTGAAGAAAAATTCGGTTACCATCATCCCATGGGTGGTCTTACTATTCATTGTAGAGACGACATCTTCACCGACCTCATCTCTCGTTTGTAGTATATCATTAATCTTGTAGAATAGGGGCATTGAAAATAACCAAATCATTGTACATTTATTTTTTCAATTTTCATCCTTTCTTTTTTGTTTCCTTTGGTGGGGAGTAAAAAGTTTCCCTGCACAAGGGAATTACTTGTGATGAAAATTAAAATGACTATAACTTTATTGGAAATGGAAGAGTATCATTCATTCCTTAAAAAGATATATCGTCTCTTTATTTTATGGTTATGCTTCTCTTCTCTACATGTATGCAAGTCAGTAATATGGATGAATCTTCATTTTTTGAAACTTAGCTGTTACTATAAAGATATTAAACCATTTTCATTGACTTTCTTTTGGTTGGGTTGGATCAATGACAAAAAAAAAAAAAAAAAAGAAATGCTGGTGAGATTTCATATCTCAAGTTAGGGTCAGAACTTTTTCGTAATTTTTGTCTTTACATGAAAATGCAGTTGGCTAAACCATAACCATACACTTTTTTCAAGCCTAAGACTGAAAGAAATTTTGCAAAATAACTCATAACAACGTTTTATTTTAAGGCATGTTTTTTAATTGATTAAAATAAATTTTTTATGTTAAAAAACAGTTAGAGACATATATTATAAACCCATCAAAATTATCAAAATCACTTTAAAACGTGTAAAACTAAATATTAAGTTAGTCGTGAAATAATCAAAGTCTTGTTCGTGATTTTGAATACGTCAACAATGGTTTTAACTATTTCAAACATACTCTTCCCTGACTTCAAACATCGATGTATTTTACCACAAATTCAAAGGTCTCTTTTGTTTAGCCATTTTAGTTGACCGCGAACAGTTTAACTTTGTTCAAGATAATAACATCAGTTTAGTTGCTGTCATTTTTTAGCAAGTTATTGAGCAACATTGAAAATATTTGAATCATGTTGGTGTGTTATAGTCAATTCTATTCGAGAAAATGGGGTCCGTTGGCCTCAACTTCTTTGTTGGTGTAGCAGTACTACACTACTAAGCTTAGGCATATTTCCAAACAGTGGAAGCTAATAGTATTTCTTCCATTTAATGAAAATTTATCTATAAACTGTGTTATACAAGCAAGGTTGAAGAAAAATGTGGGATGTCCAAGGCTTGTCCTTGGCTATAATTGGCCCACATTTCTTCTCACCAGTCTTCTGGGTCTTTTTCTTCAGAGCTAAATGGGGCCTTGCATTGGTTTCTTCCATTAAAAATGCAAGACAATGTATTTACTAACTTCACTAGTAATTAGAAGACAAACCATGTGATGATTCCTAGCTCATGTCTCCTGCTAGGGGCGTTCAATAGTTTTGTAAATGCATCCTTATCATCTATATATACACATTTGATATGGTTTAAACGCAATCACAGTTCTTCACTTTCTCATTCAAAAGAAACTTTCGTCCCATAAAAACGCGTTCGTCAACTTTTGAGTAGTTCTAAGTGTGTTTACTATAGTGTACAAT

**>CsSAUR51**

ATTGAAAATATTTGAATCATGTTGGTGTGTTATAGTCAATTCTATTCGAGAAAATGGGGTCCGTTGGCCTCAACTTCTTTGTTGGTGTAGCAGTACTACACTACTAAGCTTAGGCATATTTCCAAACAGTGGAAGCTAATAGTATTTCTTCCATTTAATGAAAATTTATCTATAAACTGTGTTATACAAGCAAGGTTGAAGAAAAATGTGGGATGTCCAAGGCTTGTCCTTGGCTATAATTGGCCCACATTTCTTCTCACCAGTCTTCTGGGTCTTTTTCTTCAGAGCTAAATGGGGCCTTGCATTGGTTTCTTCCATTAAAAATGCAAGACAATGTATTTACTAACTTCACTAGTAATTAGAAGACAAACCATGTGATGATTCCTAGCTCATGTCTCCTGCTAGGGGCGTTCAATAGTTTTGTAAATGCATCCTTATCATCTATATATACACATTTGATATGGTTTAAACGCAATCACAGTTCTTCACTTTCTCATTCAAAAGAAACTTTCGTCCCATAAAAACGCGTTCGTCAACTTTTGAGTAGTTCTAAGTGTGTTTACTATAGTGTACAATATGAGGTTTCGCCAACTTCTTACAAGAAGCCAAGGAGTTTCAGCAATTCCCAAGGGTTACTGTGCAGTGTATGTTGGCGAGAGCCAAAAGAAGCGGTTCGTGATCCCAATAACGTGCTTGAATCAACCATGTTTTCAAGACTTGCTTCGTAAGACAGACGAAGAATTTGGTTACCATCATCCCATGGGCGGTCTTACTATTCATTGCAGACATGACTTCTTTACTAATCTCATCTCCCATCTAAATGTTCTATGAGAAATTTATTGACACTCTATAGTTTATTATCAATCCATAGACACTAGAAATAACCAACAATATGTACATTTTTTTTTCATTTTCCTCTTTTTTTGTTTCCTTTTTCAGTAGGGAGTTAAAATGTCTCCCTCACCAAGGAAATTGCTAATGATGAAAGTGAAGAGGTTCATTCATTGAAAAAAAAAAAACTTTCTATTTCGGTTATCCCTCTCTTTATATGTGCAAACTAGTAATACAGATGGATACGAATCTTACCATTTTATATTGACTCCTCTTGGTTTGGTTGCAATCAAACAAGCACTATGAAGAGGTTTCATATTTCAAATTTGGATCAAAATTCAATGTCCATTTCGTCTTTGCATAAAGATATAATATCTTTTGTTCATTATCAACCATGGTTGAGTCTCGAAAAGTGTACGCAGTATATCCATATAACCATATGCTTTTTCTCAATTCTAAGATTGGCTAGAATGACAATTTTACAGAGTATACCATTTACAAAACATTAAGTTGTCAAATGTGTAGTCTTCCATCAATTTGAAGACTCTCCTTCGCTTAGCTATCTTAGTCGGTAAACGGTTTAATTCTATTAGTCCTTTTCACTTTTACTAAAGCTATTGAGCAGCATTAGACATCTTTGAATCATGTTGACCTGTCAAAGCAAAAACTGCTGTAGAAAATGGTTCCTTAATTTACTCAAATTTTGTTATGTTTTTGTTGGTTGATGCAGTAATTCCTCCACTTGATGAAATAATGACCATAGACTGTGTAATCTAAATGAAGTGCAAGCAAAATATATATGATGTCCAAGAGTCTCTTGACATAACTGGCCCACAATTTCTAATCTCCATTACCTAGTCTTTTTCTTATGAGCTAAGTGGGGTTTTTTTCATTGAAATATACAAGGCAATGTATTTATTAAATTCACTAGCTGGATTGTACTAATTGGAAGACAAAGCATGTAAGGCCTCATAAGTTCATGTCTCCTACTAGGCCCCATCCATTAGTTTTGAGAAGACCTCTTTATCATCTATATAAGTCCACATTTGATATAGTTTAGAAGCAACCACAAGTCTTCACATTCTAGTTTGAAAGAAAAAAAATTCTTCTCTCGAAAACGTGTTCTACGATTTCTCTTTGCTTTAAATATAGTAATTATACCGTTCAAC

**>CsSAUR52**

TGTTGGAGAGAGCCAAAAGAAGCGTTTCATAATTCCAATTAGTTACTTGAATCAACCATGTTTTCAAGAGTTGCTTAGCCAAACTGAAGAAGAATTCGGGTACCATCATCCAATGGGAGGTCTTACTATTCATTGCAAAGATGCCATCTTCACTAATCTCATCTCTCGTTTGAATGATCTATGAGAGACGAATAACATTTTCAAAATACAAAAACTCTGATAGCTAGAAATAACCAACACTTCATGTACATTTTTTTTCTTTCATTTTCGTCATTTTTTTGTTTCCTTTAGTGGGGAGTAAAATAATGTTTCCCTGGTTAAGGAAATTACCCAAGGATGAAATGAAATGAAATAACTTCAGTTCTACAGAAAATTTAAGAGGTTCATTCCTTAAAGAAGTATTTTCTCTATACCTTAGGTCATGCTTCTATTTTTATTTATGCAATCATGTGGATGAATCTCAATTAGAAAATATGCCAAATTCATTTATGTGTTGGTTCCTTTTAGTTTCAAATTTAAAATTTGAGTAAAAAAACTTAGTGGCAAGATATGATTGACCCTCCATAAAGACATAGCCATAACCACATAAACCCTATGCTTCTTAGTTCTTTGAATTCTAAGATTTTCCATCCTTCCGCCTTGTAACTGTTTTTGTTACCTTCCAGCCATAAATGGAAAACTCAATATTGAGTTCTCAAACAAAACAATGTACTCCCAAGTTCTCATGAAACAAAATATTTCCCTACAACAACTTCTCTTTTCGAGTCCTCGTATAGTTCTTCCTCCTTGAAATTGAAATTTACATTACTGTGATTTTCATTGGTATAAAGTAAAGTCTCAATTGATCTTCATTCACCAGAAACCCAAACAGCTCTGAAGTTTGAAGTTCTTCCAAAATCTAGTTTAAAGTTCATGTTAAAAGGGATAGAAGAAGTTATCAGTTGTTCCTACATCTTTTACAAGAAAACTTGTTTCCCTTTCAAGATTTCTCTACATATTAGTCTGTTAAGAAATCAATATATAAAGAAAACAGAAAAACCTAAAGTAATTGAAAACCGACAAGATATTTGCGTGATATAATAGCGTGTGTTAGCTAAGCTCACAAGGGGAGGAAGAACAATTTATTATTAGAGAGAATAATTCAGCGACACCTCTAAAGTTTAGAGAGTTTAATGCATTGGTCTAAACAATAGGGTCGAAATCCTAAATAATATAAATATGTCAAATATGAATTCAACTTAGGTTTTTGGGTTGTTGTCCCCGAACCCCATCATTGAAAAGATTAAGAGATTTTACTATCTTTATAAAATATATGAGGTACTTCTCTCATTGTCAATTGATTGTTAGATGAAATTTCTCATAAACATATTAGTCGCTATAAATAGTTAATGAACAACATTGGAAATGTTTGAATCACATTGACTTGTCAAAGAAAAATCTACTCTAGAAAATGGGTCCCTTTTAACTTGATATTCATTTATGTTTATGTTGATTGAGCAACAAGCTTCATCTCTTCTTTCTATTCTGCAAGATGTAGTATTTCCTCTATTTATTGAGATTATATCCATAAACTGTGTAGTTTTAATAAAATGGCAGCAGAATGTGGAGTGTCCAAGGCTTGTCCTTGGCTACAACTGTCCCACAGTTTCTAATCACCATTTTCACAAGTCTTTTCTTTAGAGCTAATTGGGATCATGTATTTGTTTCTTCACTGAAAGTGAAACTTTTTTCAATGTCTTTGCCAACATCACTGACTGGATTGTAATAATTGAAAGACAAAGCATGTGATGCTTCATAAGTTCATGTCTCTTACTGCTATGTCCCATCCAATAGCTTCTAAGGCGCTCTTTATAAAGTATATAAATATAACATTGGATATGGTCTAAACGCAATCACAATTCTTTCACATCCTCAACCAAAAGAAGCTTTCTTCTCTCTAAAACTCGTTCTGTGATTTTCTTGTTGCTCTAAGTATACAGATTATATCGTACACTACAAAC

**>CsSAUR53**

CCTTCATTAAGGAAATTACCAGTGATGGAAATGAAATGGCCACAACTTTACTTGAAATTGAAGAGATCTCTTACTTCAAAAAGTATTTTCCTTTATTCATGGCTATGCTTCTTTTCTCTGTTTATGTAAATAAATTATATGGATCACTTTTCTAGAGCCTGAGCATTCACAGAAAAGATGGTAACCCATTTATATTGAGTATTGACTCTTTTTTTTGGCTTGACTGCATCAAACAAGCACAGTGAGGTTTATAATAACAAAAAAACATGATCAATTGATTTTGTTAGTATATAATATCTGTTAATCATAATTAATAATAGGCCATGATTGGCCTTCCATGAAGATGCAGCATGTTCATATAAACAAATGCGTTTTTTCACTTCTGAGAGTAGGTAACAAGACTATATTGTTTATGATAGTTTGATTCCTTCAGCCTTGTAACTCTTTTTCTTGCCTTCCAGCTATAAGTGGTATACATATTATTGGATCTCAGGCAAAGCAAAGTGCTCCCAAGAACTCACAAACAAAAAGGATCTGATTATAATGTACATATATGGTGCTTCACAATCTTCTTAAAAGAAACCAAGAAGTTTCACTGAATCCTAAGGGATTTTCTGCAGTCTATGTTGGAGAAATGGTTGGTGATCCCGATAACTTGCTTAAATCAACCATGTTTTCAAGAATTTCTGTCGAACTGTAGAAGAACTTAGTTACCAACATGCTGTGGGTGGTCTTACTATTCATTGCAGATACGACATTTTCATCCATATCATCTCCCATTTGAATGATTTGTGAGAGATGCACTGACATCTACAATATATACAACCCATTTGTAGAATAGAGATATTAAATGTAACCAAAACTATGTGCATTCTTTCATTTACTCATATTTTTTTATTTCCTTCAGAGGGGAGTAAAGGAATGTTTCCCTACATTAAGGGATTGAATTGGCCATATCTTTCCTGAAAGTGGAAAAAGTTTATTGCTTGAAAAAGTATTCTCTGTGTCTTATATATGGCTTTACTTCTCTTCTTTACTTACGCAAACAATCTACTTGGATGAATATTAATTCTTAGACGATACAAAAAAGAAGCTACATCATTCATATCAGCTGTTTTGGTTGCACAAACAACAATTTTCAATCTACAATCGAACTAATACCTTCACAACTAGTATTTTATTTCAAAAAAATTTAAAACAGTTATCATCAATTCCCTCATTTCCTTAGATTTACCTATTGAGCAATTCAAGAAGCTGCTAGTAGCAAAAATGTTTCAGGGTTCTGATTTATTTCCAATTATGAGCGGATCATGAAGAAGAGAACCAAAGTTCAAATCTGTACAAGAGAACCTGTTTGATAAAACTGCTCAGAGATCCTGCTAGAGAGTACAACTTAGTTTCCTTAAGAGCTAGCTTTCCTTAAGATTCAAAATAGACAGAAAAGTAAGATAATGTTATTGTCTTTCCTCAAGTTACTTGGAGAAGATCATCATTCATTTATGAGATATATTTATTACAATTTCTACTTTGAGGTGCTCCATTCACATATGCAAGGGGAAATCCTGATTTTTCTTTCTCTAAAACCTTCACTGGTCTAGCACCTCCATGTTATATAGTCTAGGAATTATCTGCATGAATGCTATCTCCCTTATGATGGGATTAGGTAGTAGGGTAGAGCTGCAGGCCATGTACATATGACAGAGGCCACTGACTTGTTTAGGTCTGTCTGAACTTGTAACATTCATATTTATTTCATTGAACTTCAGCTGGCAGGCAAAGCCACGTGGTTCACTCTTATCTCTGTCCTTCACTGGGCCCAATTGGCTGATTTCCTCTCCACCACTTGTTGCTCAAGAGTTAAGGAGAACCGAAATATTTCATCTTGTGTATATATAGAAGTGGATCCTAATGGAGTTCTCACCACATTTCCAAATTGTTCTATCTTCCATTCAAACATAACTTGAAACAAGACTTGCAGAATCAGTATACAGTTTTCCATC

**>CsSAUR54**

TCATAGTTGTTCATTCAAGTGAGAGATCAAATCAGCAAAGATATCATCGCTACAGTGGAATGTAAGACCGCCCATTGGATGATAATATTCAAATTCTTCAGTAGTTTGATTGAGCAAGTCTTGAAAACATGGTTGATTCAAGTAAGTAATCGGAATCACGAACCGTTTCTTTTGATTCTCACCAACATAGACTGCACAATAGCCCTTAGGAACCATTGAAACTCTCTGGTTTCCGTTCATGGAAGACCGTCTTAGAAGACGACGAAGTCCCATTGATTAAAAAAAGTGAATGGAATGAGAAGATTTTTGTGTTGTTTGTGAACAACTAATATGTATATGTATATATATAGAGGGTTAACGTTAAAAGGGCTGTGAAACCAATGGCTAGAAATCTTTGAATGGGGCCTAGCAAAAGATGAATGCTTGTGCACATGGTTTTGTCTTCCAAATACTTATCAGTTAAGGGAATGATTGTGGTAAATAATGTTATATTGTCCTGTCTTTTTCAAAGAAAAATGGAAGCACTAACCCCATTGTTCACCTGAGAAATAGATTGGCATATCAGTTTTTGTACCTTAGAACATGTTAAAGAGAAACTGATACCTCAACTTTTGTGGATGATACTATAATTGGTGGTATACGTCTTGAACTATATTTGTCTTTTTAATGTGAATTCACTGAATGAGGAAAAGAGAAACAATCATCTCCCAATTGTTAGTCTTCAAATATTTGTTGTCTTGTTTCAACTTTTATGTGCCCACCAAATCCCCAGTATATATCGTCTTGATTCCCTTAATTGGTTTAGAAAATTTTTGATTGTGGCAAAGGAATGGGAAGGATTTGAGGGAAATCAAGGGATCTCATGATAGAAGATGTATGTGGGCTAGTTTTTTCGTTGTCAATTAGTTTTGAGATGAAACATTATGTTATTTAGTGAGAATAAAATTTAAAATTTAACTTTCTAAATGGCTTGAAAGTGATAATAAGTTTAAGCTAGCGAGTGAAGGCGAATTTAATTATATATCATCTGACATCACTCTATCTATAGACGTAACTAACACTGTTAAACAACCACGTAGATTTGTAAAAGTGAATTTAGCTGTACAGTGTAGTGACAGATGCATTGGTTAATAAACTATTACTATATTGCAAAGAAACAAAAATGGATTTGGAGTTAAAATTTTCAATTAGGTTGCAAGATGTTACCTTCATTTGATGGTTTTCACGAGCATCACAATCTGTATGGCACTTTTCATCATTTGTTGCAGACTCATTAGACGCTTCTTTCGTAGCAGTTTTAGAACCATAATTTTTAGAAAGCTTATGCACTGATTTTGATCTTGTTGATAGTTTCCCTTCATGCTCATGTTTGTTTGCATTTTGCTATAAAGTGACCTAAGGACAGCAATTGATATATTTACCAATGGGCAACACCATTCCAGACCACAAAAAGGAACAAATGAAATATAATTGCATCATAAACTTATTAAACATGCATAGAAGGCCTAATAAACTTACTATACATACATAAAATCACTCTAAATGGCTTAAGAGAGGTAGCAATTTGATTTTGAAACTAGTAGTCAAAGTTTGAAACGTACAAGATAGATGCGAGATGCTGCAAGATATAACATAATGGCCTAATAATCTTACAAAACATGCATAGAATAACTCTAAATGGCTTGGAAGGGGTAGGAATTTTATTTTGAAACTTAGAGTGAAAGTTTGAAATATGATATTGCAAGATATAACATAATGACCTAATAAACTTAGGAAACATGCACATAATCACTCTAAATGACTTGGAAGGAATAACAATGTGATTTTGAAACTCATAATGAATGTTTGAAATGTGTGAGATGGTGTGAGATACGTGCGAGATAAAACATACTAAACTGAACTACTTACGCTCCTAAACATATATAGTAACACCTTGAATGATTCATAAACATATATAAACGTTGTATTTTTCTTTAAAGAATCTCACTCAAGCAAAGACT

**>CsSAUR55**

ATAAAAAAGTTCTTTTTTAAAAAATATTTTACAACTTCTATCGTTTCTCTCTCTTTGGATCCATACATTTTTTTAATTGTTTCACAATTTCAGTCTCTTCATTATGTACTCAATAATTTTATATATATATATATATATTACTTTTAGCTATTCAAAATTTTATATTAAATAATCTATTATTGTTTACTTTGTTAAAAATATAAAATTCTTATCAATATAGTATAACACAAGATTAATCTATGTTTTTTGTTAATAAAATTTCTAATAAAAAAATACATTAACCTTTTAAATTGCTGTAGTTTTATTTCAATTAGTTATTTGTTAATACCAAATAAATGGTTAAAATATAATTCATTAGCATTAAATGTCATGACAATAGAATTTAATAATATTTTATAGTAAAATTTTTATGATTTAATTTGTATTAAGCTTCTTTAGTTGTGTAATTTTGTTTCAATTAAATTTTAGGAATGCTTTTAAACATACCAAATAAACTAATATATTTACAAAATTTTAGAATTTATGCTGATAGAAGTTAAAATAAATAAACTATTAATTCATTTTTGTTGGACTTCATAAATTTTGAAAGATTTATTCAACAATGAATTAATGTTAGGGCTCTAACATTTTGAAACAACACCAAACTTTCTATAACTATAATTCTATCCTCAACAATTGGTCTCTTATTTAGAGCTCTATGTGATTTCTTTTCTAGATTATTTATGATTAGGCTCCACAAGCATGAGTCCATGACATAAAAGTTATTATTAAAGTTTCCCATATCACTTTATTTTCTAAAAAGTTGGGATTTATAGGAAAGACATAAAGCATAGTCATTTCAAGCTACACACAAAAAGAAATGAAGTGAAATTAACTTTGGATAATTAAGATAATGCTTTTGCGTCTTTATATCATCAATAATTAAGATAATGCTTTTCATCAAAAAGTATCATCCATGATTAATCAATGTTTTGTGATAAATTTAACACTCATTTGATATTGGTACAAATACATAAGAATATTGCTTGATCTGCTTGGAATTATTGTTTGTTATGTGCTTGGTTTCCAGTTAGTCTGCATGGAGATGACTAAGGAGTTCCTTCTTCCTCCTCTGTTTCATGAGAAGGGAAGGTCCTTGTGCACGCCACGTTTGTGTTTTTTGGAGTCTTTGGGGGCATTAGAGGTATTGAGAGAGATCCTTCTGACGTTTGGTCTCTTGTTAGATTCTGTGTTTCTGTTTAGGCATCAATGACAGTCATTTTGTAATTACTTACTAGGTCTTATTCTTCTTGATTGGAGTCCTTTTTTCTTTATACCGCCCTAATTTGTAGATGCCCATGTATTCCTAAAGTTCACTTAAAAGAAAATAGAAATAAACTGTTTGGTCTATTTTATTTCAACTACTTGATCTATGACCCCTTCAAATTCAGGACCAAATTCCTGTAATCTTCATGTAGCCAATGGTTTTTGTTTAAATATTATGACATTTTATGATAATGTATTTGTTGGCTAGCTTCCAAAGACCAAAGGTTGCAAGACCTTGTAGAATTCACGTGTATATGGTCAAGAACATCGACAAATCAATCGTTTTGATTTACGTTTTTAAAAAGATTATCTGCCATATGATTTTGCTTATTGGTCTCTTGAAATGATTCTCGTCTTTTCTTCTTTTCAAATGATCCACAATGTATGAATGTGATGATGGCCAAAAAATAATTTGTTTGACTGAGAATTGTAGGTCCTCATGGTTTGTTATAAAAAGAAATTAACAATAGGAAATGAAGTTCACCATGGCTTTTTGTTGGGGGACAAAGCCATGTGACCTTTATAGATTATGGCCCAAAACTCAATCCATTGTGGTTGTTCTTTCACTACAAAAAGGCAATTTGCCGATTTGTTTTCTTTATATATACACACAATTCAAAGAACACCAATTCCCATCACAGCAGCTACCATTTCCAATTCTCTTGAGATTCATTTACCACCACCTATTACCAAAA

**>CsSAUR56**

TTTGATTTATGAAAAGGAATTACTGGGTAAGATGCAACGCCCCAGGTCAAGGACTCATACAAGGATTGAAAACTTGATGGCTCTAGCAGTCTTCTGCATCTCCTGTGACGTGACAATGACGTATTTGTTTGTCTTAAATGGCTTTTAAGAGTGAAAACTATCTCCACAAATCAATATAGATCCTTTCAACATGTTTTGTCCCCACTCACATGCCTAGAATTGTACTAAACTAAGTTTAACTTTAGAGTTCTTATGAATGAGTCACCGAAAAGATAGGCAAACCTTGTTGGTTTAGGTAATAACTTTGAATTCTTTAAAGTCTTTCTTAACCATATTTTCATATCCTATATCCCTCTCATTTGTGATCTTAGTTCGTGTGTGTACCTCTCCTAAATTCGGTGTTATATACGATCATTGTCAATCAGGACCTTCATTATCCAAAAGTTTGTAGTTTACTTTTCTTGGAGTCGACTCATGGACTTTCTGTCTGGCCTACTCTAACATCATGTATTTCTTCCAACTTTCTGAGGTGGAAAGTTGACAGCCGTTGGGAGGGTGATTTAGGGATAAGATTTGATAACCACGAAGAATATTCAAAAGGATCTGGTAATCCTATTGTAAGATTCTCAATCATGAACTCATCCACACCAACAAAGAAAGGAAGCAAGCAATCCCTCCCTAATCAAACACAACTTTGCGCCATCCAGTAGAAATTTAGATCATGATATTGGAATTGGACTTTACATAACAATGCCAAGGACATCTAAAGATTCAAATGAATGAATTATCCAATCCATTCTGGTACTTGACACGGTAAGGTCATACGGATTTTGTAAATGCTTCAATAATTAGAAATTATAAGTTGGAGTTAGTAGATTATACTAAAAAAGTTCACAAAGTTCGCATGGAATGATAACGATATCATTGAATAGGCATTGCCAAGATGCCAACCTGGCTGACTGGACACTGTCCTATCTCTGAAAAAAAGATAAACTGATAGTTAAGATATTTCCTCACCTAGTTCGTCGAAGTTTCTACTTCTAATAAAACCACGTAATGGACACTGCGTTCGGAAAAGTCCACATAGTACCAGAGACGTGTGGACACCAGCATTTATTACATGTCCTGGAAGCATAAATTCTTGAAGAAGCATATGAAAGTTCATTATCAAAGCATCTAGTATCCGAGTTTGAAGTGTTGCAAAAGTGAAGGGTCATTAAACTGATAGAAAGAACAAAATTTCACACTGAGGCGTCAAACTCTAGCAAAAAGTTTAGATATACAATAATGAGTACTATACTATTCATGACTCTTCATTGAGATACTGTCTGCAGATGCATAATTTAGTTTAAAGCAAGAAGAAAATGAAAAGTTAGGTCACTGTTTTCTTCAAATTAAGGGACCCTTGAGTCAACCAAATGAACCCACAGAGGCTGGTTGGCTAGTTGGCAAAGAGGATAAAGAAAAACAAAAACAAACCCAAAAAAGCAAGCAACTTAAAGAAGCAGGCCAAGTTCAGTGCTTAATCAAGTAATCTAAAAATGGAACTTAAATTTTTTCTTTAATTGAAAACATACTACATGTTGATAAGAACATGCCCAACATCAATTAATTTAGTGCTTTATTTGATTAAAAACTCAAGGATTCACAAGTTGTCCTTTCTTTTCCTTTTTCTCTCTTTTTTTTTTCCCTATTTTTCCTAAACACATACACATTTGTGCAAGCAAATGGTAGGGTATTATGTTTTGCACTAAACAAGGCAAACCATGTGAAATATTGCCAATTTCTTCACCTGCTATGTTTTCATTCCTTCTTCTTACTTTTCTACTTATCCATAATTGACAAAAATTCTACACACACACACACACATATATATATGAGCACAAACCCACATACAACACTCACTCACTCAACTCAAAGTACCATAACAAGTCCTTTCAAATCCAGTTCAAAAGGCAATTCAAAACAGGGGATCTTTCTTTGTTGTAAAGCATAGAGAT

**>CsSAUR57**

CTAATGGAGGGAATAAGAAAAGTGAAGAAGAGCGATGAGTGTTGAGAGCATTGAGAGCGACAAGAGAACGTGAATGAGAAGAAAACTAAGCGAATGAGAGGAACAATTAATTGAGATTCAACTTTATTTATATCACAGGGGTAAAATTGACCTTTCACACGATATTTAGTTTGTCAACATGTTTTAATAAACGAAGGTTCCACACGATCGGTGAATTATTTATAAATGATGGCACACTTACTTTTAAGTGATCATGTAAATTATGACTATGCAATCCACACACAAAAACATAACCAATCATATAATAAATGTTAAACGATCGCTTATTAAACCTAAACAATCGTGTAATCCGAAAGCCATTATTAAAAATGTTCAATTGTAATAATATGCATTAAAGCTTTAAAGGTTAGTTCTAATAAAGTTTTTACAAATAGGTCATAGTTTTTACTTTTAAGGTTATGGAATTTTCAGCAAAGAAAAGTAGTGAAGACTAGGAATCTAGGAGAATTTTTAGAGTAGAAATATTATTATACAATTTGTTGATGATACGTAACGGCACATTTAATTAGTAGAGTTTTAATACACAAGTGAAGGTAAAAATAGAGTTAACCCAGAAAGTAACTATTTACTAAAACTAACCTCATTCTTTTTCACTTCTTATTATATATATATATAGACATAGCCTTGTCTATATATATTAGTATTAAACCTTTTGGGTAAGACAAAATAATGAATTGTGCATACTTGAATTTAACGTGAGTATGTAAACTTAATTCAAATTCAACGGTATTTGACATGATTTTTCTAAAGGTCGAAATTTAAACTTCCACAACTCCAACATCTTTTTACTATAAAAAGAAGTGAAAAATATATCATAGTAAACATATACAATAGTTGACTTTATCGAGTATATACGATAAGATTCTTGCAATATCCTTATTGTAGATCTTGTAGAAAAAAGAAAGTCGAAAAATAGTGGATATCTAAATGTGAAGGTTGTGACAAGTCAAATTATATAGTAAGGTCCTCCTTCCACCTAGAAATGCTCTCCTAAAGTGGTGGGGGAAGAAATAATGGAATAAACCTACAACTCAGTCCTCTAAAATGAGTGAATCCTGTTGGAATAATACTACTTGATCCACATTCCTAGAGTGGTGGAAAAGAATGTAGGAGCTACCTCTCTCAAAAGAAGATCATCAAATTAAAAGGCCTTTTTTCTTTGGTTGACATTGAATTTTTTACAATATATTTTCAAATCTTCGAAACCTAAAGTTGGAAGGGATAATAACTAAATAGTGACATGTATTGAGTGGTGCACTAGCTACATAGTTCATGTGTGTCAATGAGTATACTTTTTCTTCTTATTTTTCAACTTACAAGTGGAAGATAGGTAAGCATAAAGTATATTTTGTATATGGAGCTAAGAGTTGTATTTTATATATGTTTTCAACTTTTTTGATGATGAGGATTGCAGTGATGTACAAGATTATGGCATTGGTACATACTTTATTTTCGATTATAATTTTAAGAGCATGGAGAAATATTTACAAAAGTAGTAGCAATCAGTTTTTAATAATTTGTTTCCTTTAATAATACATAAACAAATATGGTGAATGGGGGCAATATTAAAATAATTAAAGACAAAGATAAAAAGATTCATAAGATAAAAAGATGATCAAGATAATGTTTCAAGAAAAGATTAAAAAAGTGAAAAAAATAATGTGAGTGTCTTGCTTTTTTTCATCTCTTCCAACACTATGGAAGCCAAAGGAGATGAACTTGCATGGAAAAAACCAGGGGAGTACCATAGCGGTATTTCACACTCACGTGGGTCCATTCTCGTCCATGTTGAAGCTTTGGATAATACCACTTTTTAGCTAAACCCCACAATCCAAAACCTCCCTAGAGTTTTCCATATAAATTCCCCGATTCTGAGCTCAAAGAACCATCCAATTCTCAACCGCCGAAAAACAACATTTCGTATACAAGTTTTTAGCTAA

**>CsSAUR58**

GTGCTCCAAATGAAAACTCCTTTGCTTTTGCAAAAAGCATCTCATTCAATGAGCCCAACTCTTCAATCTCTTAGATTCTTTTTTCAAGGATTTTTTTTGTGTTCTAATGGTTTTTGTAACACAAAGAAACAAAAATTAGATTTAATTAGATCACTATTAGATCATGACATGGACAAATAAATCATTACTTTTGTAATAAACCACTGGTATAAATAAGGTTTTATATATATATATATATATATGATGTGATTAAATGTTCAAGGTCTCATTTAATTTAATGATGAAATTTAGATGATTAGGGAGTTGATATTATTTAAAAGATCCGGATTAAATTGATAATAAAACTAACAAATCCTATTTTTTATATTTTATGTGACATAAACTTAAAATGATAAAAGGAAGAAAAAAAAAATTAAAGTTCCTGTTGGTAGATAATGGTTTCATTTTTTATTTTGAAGTTGATGTTTGTTACTTTCCAATTATTAATTCTGATTTTTACCTTTGAATTTAAAAAACAAATTGACAGTAACAAGTTTCTCTTTTTCTTTTCAAAATTTGATTTGATTTTTGAAAATATTGGAGAAAATGTGGATAAACAAACATAATAGAATATGGTCATACATTCATCTTTTCTTCTACTCTCATTTATTTTCCTTGTATCTTTTGTTAGGTCATGGAAGATGGACCAACTTTAAAACGTTTAGAGAGATTGTGTTAATTGTGTCATTGAATTAAATTCACTTTTGAATTATCGTCGCTCTAAGAGTAAGGTAAAAATAAATTCAAGTCTCATGAATCATGGCCTCAAATTTCCCAACAACTAAACCTTAATATTTCTTCTCAATTTTATATCAAACCATTGATTTCTCATGAAAAAATGAAGGTTGTCTAACACAATAAAACTTGTTACAACATTTCTAAAGACATCACAAACATACGTAGAAAAATAAAAACCATAACAGAAAACGAAGTCTGTGTAATCCAATTCGGTGCAATACCACCTACGTTTAGGGTGTATGTACGTAGTGTGTGCCCATATATAAAGTCAATCAATTACAACAATATACTTATCTATAAATAAACGACAATTACAGTGACACTCAAATAGCTCATAGATAATGGTCATTTAGGCTCCTCCTAAGTATGAAATCCCTCACCTTCGACACTCTTACACTCTCCGTAAGTATAAGGATAATTGTGATATTTCATAAAATAATGAACATCTTCCCACCAAACTACACCTTGATCTAAATAGTTGTATATCATGATAAAATATTACTTTTCAAAGGATACAAGTGCCAAAACAATTAAGATGATGTAAACAATATTCTCCCCTCTATATACGTCTTCCATCTGTCCTACTATTTTGATTCAAATTTGAAGAGCTTCTATGATTATTATATACATCAAATAGTAAAAAACTAGAAATACACATTGAGATTTGAAGACCTATCGGCCGACAGCTCAAATATTCTAAGTCTAGTAGAACTACTTCTTTCCATTTCTACAGAAGAATAGTGAACACTATTCTTTGAATTGATGAGACAATAAATTAAAGAAGAAGTGCAATTAAATTCTCACTTTTTAAAGGCAGATAGATATTACATTACAATGGAGGATATCATCTAAAGTCATTGTCACTACCCATATATAAGATTTGAAATAATAATTGATGGGTAGAGATTTCAAATATATTCATCCCTCCCCAATATCAAATTATCCCATCTCCTTCCTCTCATGTGGCTGCTATTTCTTGTAGTGAAACAAAATCTATGGACCCAACTTTGTCCTCAATAAGAAAAAATGGATATACACCAAATTAATTGAACCCAATTTTTGAGAACAAAAATTAAAAACATCCCACCTTCATTTCACCTTTCCCCCAACATTCACATGGCCATGTACCCACTACAAAAATCACCTTTTCATTCAAATGTATAAGTATAAATCGACACCAAAGTTTTCTTTCATTGCAACATCCCAAACCAGAAAAAAGAAAC

**>CsSAUR59**

AAAGGAATGGAGAAGAAGAAAGAAAGACGCGTAGTATAGACAAGAATATAATGCAGTTGGGACATACCCTTCCCCTTCCCCTTTCCCACCTTGTTTCCCTCATCTCCAATTAATATATTTACATCCCCCATATTCTTTCCTATTCCTATTCCTATTCCTTTTCCCTTCTTTTTTTTTTTTTCTCTCTTTTCTTTCTCTCCTTCTTTTTAATAATACAATCAGTACTCAAACTAAACATAAACATAGTTTAAGTAACTGCAGCAATTAATTAGCATGTACTCTACTACACACCATCCCACCTTAATGTGGAAGCTTGATCAAATTAAATTCTCATTTTCTCAATTATTACAACTCCTTATTTCTTTATCTTCAATATAATTAATTAATTAGTTTTATGATATGTGAATGTGAGATTCCAAAATTAAAAGAGTTTTAGAAAATAGGTATAGGATTAATAGGGGGAGTGGGGAGGAGGGCATGTGGAAGGAGGGCACCGCACGCGGTAAGGGTAGGAGTGTTGCCGTAGGAAATAAATAGGAATATCAATATTCAATCATATTTAAGTATACTATATAATATATATTACTCCCACCTCCTCCTCTTATTCTTCTTCTTCTTCTTCTTCTTCTTCTTTGTTTTTAATCATATATATTCCCCCACACATTGAATATTCAATATATATCCTATACACCGATCGAAGCTATACTTCCCCACTCATTCTTCTTTTCAAATATTAATTTATTCAAAAACAAAAAGCATACTTACATTTTATCAATATATATATATGATGATGATCCATAATTAATAAACATACAAATCACTCTTAATTTAACCCATAACCCTCTTACCTTATTCCAATCTTTTACTCCTATTGTATAGTTAAATTTAAACTATAACAACAATAATAATAATATAACCTATAACAGTACTTTATATTTATTTAGTATATATGATGAACAAGTGAAAGTAAGGTGTAGTTTTTTTCTAACAAATTAAAAAAAGAGTGTAATTGTTTGGTTAAACTAAACCAATTGAAAGTAGAGAATTATTTCAAAATTTTGAGAAAACAAACTAAAAAAAAAATATCTCTTATGTATTTAACTTTTGTCCTTCTCTTCTCAAAGTATGAATATAATATACTAATTAAAGTACTACAAAAGTACTTTTTAATTTATTTTTTTGAAGAAAGTGTCCTACACACATCCATATCATCATCCACATTGGTATGACCATGGGAAATATGAGTTGGTGAAGATTACTTTCTCCAACCAAAAAGTTGATTGTTTCACCCCATTTTCTTAAGGTAGTCTAAAATCACTACTTAATTAGTTTTTTATATAATAGATATTAAATTATGGTTATTAAGAGGTAAATTTATAACTTAACCTATATATATGTATATTAAAAAAAAGAAGAAAAAAAGGAGAGAATAGGAGCATAACTAAATATGATAAATATAGAAAAGTAAGAGAAGAAATAATAAAGAATAGGGAGAGAGAGGGAGAGAGAGGGAAAGGGGGGAAGGTGGTGTTGAGTGATTGTACCTGCCAAGTACTCTTACCTGTACCTCCCCACCCTCTCTTTTTCTTTTTCTTTTTCTTTTTCTTTTTCTTTTTCTTTTTCTTTTTCTTTTTCTTTTTCTTTTTCTTTTTCTTTTTCTTTTTCTTTTTCTTTTTCTTTTTCTTTTTCTTTTTCTTTTTCTTTTTCTTTTTCTTTTTCTTTTTCTTTTTCTTTTTCTTTTTCTTTTTCTTTTTATATTGTTATTGTTATTATTGTTATTGTAATTTCTTCTCTTTTTTTTATATATAAAAATAGAAAAAAGAAAATTAATCTGGAAATTCTAAATTCCAAATTCCCACCTCCCCCTCCCTTCACTTCTATATATATTCTTCCCTTTCTTTTCCCAATCTTCAAGCTCTCTCTTTCATCTTCTCTTCCAACCAAAAACAACAATCAATATTCACTCTATTTCCATATCTCTACCTATCATATCATTCATC

**>CsSAUR60**

TAACGATATCGATTCCATTCAGGATAACGCCTCTGTTAAAGAGGATGCTATTCCTCCGGTTGCTGGCAGTATCAACCGACCCTCTAAAGCTGCTAAGGGTGCTGTTCACATGGAACACGGAAGAGACTTTGAAAGTAAAAGTGGAAAGAGCGCTGAAAAGAAATTCAACAAGTGCAATACCAGTCGTGGTAAGAGGAAAGTAGAACGTGAAAAGTCGAGTAATTTTGATTATTCTGAACGAGGTAAGGTTGATAATCATGCATCAAAAAGATCAAAAGAGTCGCCCCAGCATGATACATTTTGTACTAATTTGGAATCTTCAGCTCCCTTGGTAGAAGAAAGCTCCAAGGAGGAACGTAACAATAGTGCTGCACCAAGTCGATGTGACACTGAAAAGGAATTGATAGTGAAAAGGAAAAAACAGCGTGAAGCCGTTGATGCCATCCTTTTTTCATCACTAATTCCTTCAAAGAAGTCTGAAATGTCAATGAAACTTACCTCAGACAAAAAGCCACACTCACTTTCAAATGTTCATGGTAGCATGAAACCTCCAAAGGGAAGAAAAGGCTGATGGTGTCTATTCATTTGAGGTTTGGAACATAGCCAATTCAACTATGGCACACTGGTGGTTGAAGATATCATTCTTGCACCTCTTTTTTGGACTGACTTGTAGAATTTTGTCAATATTTTGATCTTTAACAAAAATGAAAATTTTTTGTTCACTCCCACCCCTTGGTTGATTAACTGTAACGAACACTCAAAGGTGCAATGTAATAGATATATATTACAATTTCTAGCACAAGGAAATCTCTCAGTAAATATTTCTTTTATTTTAGTGATTAAAGTATGATATATATTACATTTTCTATGATTTCGAAGGTGCAATCATCGAGGACTAAATTTTATAGTTACTGAAACTTGCATTTATATGCCCTGGTTTGTTTCCAGATGATTTTCTTATATTTTAAAAATAATTTCAAACTTCTTTACTGTCTACTACTACAAATACTTTTTGAAATATTTTTAAAGTTTATTTTAAACGATCTTTGTCAAAAGAGCTAAAATAAAATTGATCTTTTGAGAAATACTTTTTTCTCCCCTTCAGTCCTTAAGTTTTAGAATTTTAATTAGGCATGGTATAACTATGTAACTATCTAAAATAACTATAAGTATGTTTTATCTTAAGCAAACGATCAAATATAAGATTTTGAAAAAATATTGTATTCATCACTATATATTGATTAGGACATAATGTTAAGCTTCTCTTTGAAATTGCTAATTATTGAATTACCTAAAATCGTTGAAACACCAAACTTTGACCATTATAATCACTAACGTACTTGTTGATGGATGAACTTTAATCACCTACTTGTATCCTAATTTTGTATAAGTATTATTTCATGTACATTATATTAGATTGAAAAAAAGTATACCTATTCTTGTGCAGTCCAGATTGTACAAATTTGCTAAAGATGGATGCAAAATGTGACCATATAAGGTAAATTGGTGCCACCTAATAATTGTTTCATTAGATTTTTTTTGGTCGTCCTATTAGGTTGTGCACCAATTTTTTTATGTTAGAATTCATTTTCATAACATACATGCATGTTTTTTCTAATCTGGCAATTCATGTGACTTGTACTATGTCAATTAACCACCTTGTTTTGACCTTAGAATTTAATGTGATTCCTTGATGTGCTTGTTTTTTTTTTTTTTTTGGTGATCCACGTTCCATTTCCTTATTAGTAGCTTATTGGTTTCATTTCTTATATACTTATTAAACAACCAACAAGTTATAAGATCTGAGTACAACATTATTTGATTTGGATTGGCCATTACTCTCTGTTATGGATGTAGGATTGAAATATTTTATTTTTTGCTCACATGGGAAATCATCAAACCAAGCACCTGCAAGATCTAACAACCAAATCATACTCATCAAGACAGAAAGAATTATAAATTGGGTGAAGAAGAGGCTTCAAATATGCTATTATGTAACA

**>CsSAUR61**

TTGTGTAGAGTTAGTGACTGTTAAATGTTGAAGGAAAGGAGCCTATACTACCGTAGCAGTAGTATTTTTCTAGGGTTAAGATTTGGATACTAACAAGTTTGTATGTGATATGGACATTCGTCTTAAGAAAAGAAATTGACTAGCTACTAAATCCAATTAGATTGACCATATACCTCGAAGTTGACAACATTGATGCTAAATAAACATTTCAGAGCGACTAACAAACTTCAAATATACCATTGTTGACTGAAATAGAAAACTCAGACAAGGACCTCAAAACAATTGAACTTAGCATAGAAGGGATTACGCATCGAGAATTCAATTGAATTGATTTGATTCCTCGAAAGGGAGTTGACTGATAAATACTAATAATTGGGAGAAAGATCCCTTAATCATTAAAGTTGAAAGAGAAAACTGATGTGTTACTGAGACAAGACGAGAAAAAGGATATGGATTTAAAAGATGTTTGGTTGGAAAAATGAAAAGCTGAGAAGGCAGATTACAAAAACATAGAGTTGCAACCAAAAAAAACAAAAAAGAAAAAGGTAATTAAATGAAAGGTAATCAAGCTTTTTTTTCTTTCTTTTTTCCTTTTTTGAATCAAAGACGAATTTTTGAAGCTGGATGGCATTGGCAGATTACCAACCAACAAGCTTTCTCTCTTTCTTTTTTAATTTTTGTTTTTGTTTTTGTTTGTAATTAAAATTCCTTCTTTTTTTTTTAATTTCTTTTTTGCACGATTCCTTTTTCTGACACTATTGATATGCTTAAATATTTGTTCATATTGACATAATAATCCCTTCTTTATTTTCATTTTTAATACCCTTTCAAAACGACAACTAGCTCTTCACTGGCATTTTCCACACCTATCCCTTTTTCTTTTCCCAACTCATTTTCTCTCTCTTTAAACAAAACACTTTTTATTCTTATCTATTTGTTCCTATTATCATCTTATTTGATATTTAGCTCCAATCTAGTCCCTTAATTTTAATAAATTTCTCCCTAACACCCTCACTTTCTGTGAAATTTATCAATGCACTAAAGTCTATAATGTGAAGGGAGAAAAAAAAAAGAAGCTAAATTACAAGATGGATTTGTAATTTATCACTCACAATAATTAGAACAATCATAAATTGTAAAAGAGAAAAGAAATCTAATAGAGGTAGTTTATGACAAAGCTTCACACTGATAAGATTGTTAATATTCATTATTTGGTAACACCATCCAACGATGATATATTTGGAATTATTCGATCAAATTTTAGAAATTAATTTGATTAATTAATTTAAGTTTTGAACTATAGCCCATTAAATAGGTTAATATAAGCATAAACCAACTTATCAAAATATTACTCACCTTCTTAAAGGCTAATTTCAAATTTATAAATTGATCTTGTATACATAATACATGTATTAAAATACATACGAGAACTTCATAGTTTCATTTTATTTTCTTAATTCATTCCTTTGTTGAATAAAAAATATATAAATTAAAAATAACGAGTCCCCATAATATAAATATCCAAATTGATTCTTCCAAAATAAAAAATAACCAAATTGATTTGTCAATATAATAAAAAGAAGATTTAAAAAATTGAAAACTGATTAATGATTAATTGATTAATTGATTAAAATTGATTAATGAAATTACACGCGTCAAAACTGGTACGTATATGTGGATGATTATGTACTAATTATAATTTTAATACAAATATTAATAAATTACAATGGAATGTTTTAATTAAATTACAAAACAAATTCATTTCTTTTTATTTTCTTCATATCTAAAATTGAAATAAAATAATAATAATAATAATAATAACAACCAAAGATATATTTATTTATCAATATACGTGTGGGCCATGCACGTATAATTCTTCTTCTTTTATTTTTCTTCTTCCATTTCCCTTTGTTTTATATAACGAAGCTTCCCTTTATCTTTCCCTCACCTTCTTCTAATTCTCTCTCAATTTTCCCCTTTTCTCTTTACAATTTTCCGCC

**>CsLBD1**

GTATAATCCTTAATTGTGAGTTATTAATGACAAAAACAGACAAAGAAATATAAATGATAGGTATATATGTGTGTGTAATAGAGTGCATGAGAAAGAAAAGAAAGAAAGAAAGAAAGAATGGAATAAAAATGGGAGACAATCCCACAACCCTGATATTATTATTGGATGCAGGGTCATAGTCATTCTTTTGTTAGTGTACCCACCATTTATTATCCTTGTCTTTTGCATATCCCCTCTCCATTCCATTCCCCCCCTTACCCCTTTCTCTCTTCCCATCCACTAAATATACTCTCTTAGCTTCTCCCAATCTTTCACTTCCACTCAATACCCTCTCTTCTTTTGTTTCTCTCTCACCCGTCTCTCTCTTTCTTTCTTCTTCCTTCTTCAAAATTAGGGTTCTTCATCAAATTCAGCCCTAAATTACCAAATCTCATAAGACTTCTTTTAGTTTATGATCAAAGGGGAAAGGGAGAAAAAGAGGGTTTAGAGCTTTTCTCACACACTTGAAAGGTAATTTAAAAAAGGAACTTCCTTTTTTGGTGGGTTTTTATTGTTTTAGTTTTTTGAATTGATTCCAAAAGAAAAAAAAAAAAAGGGGAAAGACCCTAGATCACACATGATGAGATGTAAGTTTACACACACACACAGACACATATATATGTAGCTTTCTTTTGTTTTGTTTGTATAAGTAATAAAAAAATTCAAATTAAAGAATAAAGGTTAGTTTTTGTTTGTTCTTATGAATTTATATATATATATATATACATATATTCTGCAATTCAGGTGAAGAAGATGAGACACATGACTACTGGTGAAAAATAAAGTAAAAAAAGAAAAAAAAGTCATCAGAAAAAATATATATATTTTTTCTGATGACTTTTTTTTCTTTTTTTACTTTATTTTTCACCAGTAGTCATGTGTCTCATCTTCTTCACCTGAATTGCAGAATATATGTATATATATATATATATATGAAACCTGTCTTTGTAGCTGCTTTGCACGCAACCTCTACTTTCTTCTCTCAATTTACACTTTCCAAAAAAATAATAATAAAGAAATAAAGTAATAATTGATGGGTTTAATTAATTATCATTAACTTGCAAAAATCAAAATCTATTATATGGCAATGGGAGAGATGAAGAGATCTAACTTCTTTTTTCCTTTTAAAATACCATATCAACAATTCGGTAAGTCTCTAATTCGAGCATAGATTCAATTCTCCATTTTCGTAATTGATCAAGAAATGAAATTAATATATATAATTGAGTATAGGATATGCATGGGAAGAACAATAATAGTGTGTGTGTGTGTGTGTGTACACACACACATATCTGCAATTTATGCAGATGTTTCTGCAAATTTAATTTAGGGTATCGTACGTGTCTGTCGATGGGAGTTTTTTTCTAGTGCGCACGCGTGTGAAAGAAAGAAAGAGATCTTCATTCTGAACAAAAAATAAATTACTTGGGATCAGCTACAAAATTTTAAATATAATGTTCTTCTGAATTTCATCTGACTTTGTTCTAGAATTTCTTTTTCTTTTTTCTTTTGTTAAAAATAAAAGAAATTCCCTTTACTTTTTCTGATTAAACTACACATACACAGAGATGTGTAATGTATATATATAACTTAAAATGAACAAAAATAAATATATAAGAGACAAAAGAAACAAAGAACTAATTGGGTTTAATGTTTTAAGTTATTCATATATAATGGTTGTCTTGTTTAACGAATTAATTCAAACTTTAAGGTGTAAGAGAATGAGGGGAAAAAATATACCTCTTGTACTCTCTTGTACTGTTACCATTTTTTGAATGAATGTTGTTGGCTTTTGAGTCTGGAGGATGATAAAGGAGTTAAGGAAATGTGTTAACATGAACAGTAGAGATGTATTTCAAAGAATATCTTGGTAAGAATAATTAAAGAATATGTATAGTATAGATTAATTAGTTAAAAGAAAATTGATGATAATGTTCTTACTTTGATGGGTTAGGGCAAGG

**>CsLBD2**

CAATAAATAGCTAAGTCATTCATCAACAATTTTTTAAAAAAAGTTTATATGCTTTAAATAAAATTTAATGTCCCTTTCAAACAATGTGGCATAAGTTGTTAAACTTTTGATTTTTGTCTTCTAACAATTTTAGGTGTAATAGAAACTTTGATTTATTTGATATTCTTTATATTCTCTTTATTGAAAAATGACACTACAATTGATTTTGCTGTTGAAATTTTGAGTTTTAGCCTTTCAAATTTTAAAAAGAATAAAGATAACAAAATTTTACTTTTTTAACTCTAACACGTTGTGACTAATAAAGTCTTTTGATAACTTTAGTTTTGAAGTTAAATCATACTCGTTTTAATATTAATTTAACTTTTTAATGAATTTGAAATTTGGTAATGACTTCTTATTTTAATGACATTGTTCAATTACCGTTAATTCTTTATTTTATATTATCTTGATGTGAAAAGAGTAACTTTTTTATATATAAAATTTAGTTTAGGTTATATTTGAGCAATTTTAGCCCTAAAGTTTACTTTTATTATCACTGTTAGTCTTTTCGGTCGAGTTTTCTTACAAAATTATTAATTTTTAATTATTAAATTACTTTTTTTCTTCTCCCTCATTTTTCCAATCTCTCTTAGTCCCACATGGTTGCCACTATTGACCATATTTATCGATTGTTTCGAATCCCAAATTTAAAGGTACATGTTAGGAGATATTTGACTCGACTCATACTCTAAACAAGATTACAAGGTATGTCAAGAAATTATATTGTCTCGTACTATTATGAATTGATATATACAATAGATATGGTCAATAGTGGCAACCATATATATAATTTCAAGAAAATTTTGTTATTCGATCAAGCTTTGTTTTTGTTTTAGATGGTACCTTTTTTCACAACTAAAGTACAATTTACGTAAAAAAAAAATATTTGAAGTATAATAAATTTTTAAAAAATGTTTCAAGTTGGTTTCGTGTATAAGAAAGATGATGGAAAGTTCCTCTTACTTTTACTGCCAAACTCTAGTTGATTTACAACATTGATTGAGATGATACTTATCCTCCATGTGGAACTAATTTCATGAATGACCATATAAAAAGTAAGCTCCAATTAACTATGTTGGTGTGGTTGAAGCATACCTCTAAATTGATAGCGAAAACAGACAATTTAATGGAACACAACACATTCATAATTCAGCATGACTTAATACTCATAACTTTTACCACTAACAATCAAACATAAATTGAATTTAGAACAGTTACGAGTAATAGATGAATGATAAAAGTAAGATTAGAATGAGGCATAATGGGATATGGGATAAGTTAGGAATAGCCGCCGTTGGATAGGGTCCACATAAGGAATTAAAAAGAGAGGGAGAGAGAGAGAGATAGAAATAAAGAAATGTGTGTTTGTGTGTGGGAATCCGTAGATAGTAAAGAAAAGTCCGAGTTGATTGGAACATGGAAAGGCAAGCGCGTGGCTTTGGGGATTTCAATTCCCACTTAATCCCATTCTCCGCTTTCAATCACTATTCTTAATTCTTTTCTTTTTCTTTTTTTTCTTTTTTCCATTCCAAACTCTCACACTGTGGGCGCCCTTTGTACTTCCCTTCCATCCGGTTTTCCCTTACCCCCCCCCCCCGCCTTCATTCATACATACAAATCTTACTCATCCTTTTTATCATATAATTATGTGCAACTATAAACTAATTTTTACTATCATAACCACATCCATTCTTTATTTGTCAAGTGGTAAAATAAACTATTAATTTATGGAAACAAATGACAATTTGTCAACAAAAACTTTCCCCATTGGTTACCAAAACCAGAGAACGACCAAACTGGAAAACTACCCGTTTTCTTGGAAAACCACTTGCGGCCATCATTACAAAAACAAAACCCACCGTCGATTCCTTCCCCACTTTTCTTTTTCTATTTCTCTTCTTCTCTCTCGATTTTTCATCATCTTCTTTTTCTTCTTCTCATACCAATCCAATTCCTTCACT

**>CsLBD3**

GTGATTTATTTTCATTTAAATTTGAATATGTATTTGAACAATACCCACCAAATATTACAAATATGGTAATCATACATACAATATTTTTTTTAAAAAAATTAAAAATCGCATCAACCCTTTAAACTCAAAGCTACTGAGATAACTTTATATATTGGAATGGTCACAGTTGAAAACTTTTAGTACTACAATATTTGAAAATCATCAAATGAATACACATAAACTTTAATGATTTGTACTAAAAATTATAAATATAACTAACGATATACTAGTAATATTCTTGAAAGGGTTCTCTTCTCCTATAATGTGTTAAGGTAATGCCATAATTATGGGTTGGTTAGGCACATGCACTTCCCTAACCTAAACCCTAACTCTCAAACTCAGAAGAGAGAAAAGAATTGTATATGAAAAAGTGAGACCTCTCATTTATATATAATAATTAAGGAAAGTCTCATGACATTTGAGTATATTTATTTGGGTTTCTTTTTGTTATTCTCCATTCAAACTTAGTGTTTATAAATTATATTATATCCAACTTAAAAAAGAAAAAAAATAATTGTATTCCTCATTTGTAAGTGTATAATTAGTTGTGTATGGAGATTTAAGAGATGCATATATGGTGGGTTTTTGGAATTGTTGGAATGTCAAAGTAGGAGACATATCTCTGAAAATAATTATCATTATTTGATTTGATGCTTCCTATTTTACTGTACTTTCCATTCATTTCCCCATCTTTTGCTTCCACTAAATTCTCTATCCCCTTCTCTCTCTCTCTCTCTCTCTCTCTCTCTCTCTCTCTCTCTCTCTCTCTCTCTCTCTCTCTCTCTCTCTCTCTCTCTCTCTCTCTCTCTCTCTCTCTCTCTCTCTCTCTCTCTCTCTCTCTCTCTCTCTCTCTCTCTCTCTCTCTCTCTCTCTCTCTCTCTCTCTCTCTCTCTCTCTCTCTCTCTCTCTCTCTCTCTCCTAAATTAGGGTTTTTTTTTTTTTTCTCTATGAAGAGAGTTCACTTTGATCAGAAAATTGAAGAGACCATATATGAGTTCTTCATTCATCTAATTTTAAGTTATTCTTGAGTGTTTTTTAGTATAAATTAGTTCCTTATAACTCTACTAAGAATATATGTCAAGAAAGCAAGGATCTTGAAAACTGACAGCCCTAAGCAGCAATCCAAAAGGTAGTTTCTTTTGCTTTTTTTTTTTTCCTGATCTATGTGAAGAAAATTAGGATGATCACATATCATATGTACAGTTTTGATTTTGATTTTGTTTTATGTTTCTTAAAATATGTTGAGAAAGATGACGAGTTTTTGGTTGATTTGAGAAACTGTCTCAATTTTGTCAATATTGAACCTGATTTGGTTTTTTGAAAACATGCATATGTCAAATCTAATCCTCTTTTGTTTTGTTTTGTTTTTTTGGGTAAAAATTCAGAATTAAGATATTTTCTGCCATGTGTTCTGAGAGCTTGCTGATGATTCAACTGGAAAACTCAGTTAAAAAGTTGAAACCTTTCATCAGACATGTCAAGAGAAAAAGAAGAAAAAAAACCCTTATTTTTTAGAATGTTTTTAAGAATTTTAAGGGTTCAGAGAGTTCATTGGAAGCATGAAAACACAGAGTATTGAAATAACTTTACAAAATTTACATAATTTCACTCCAAGGGATTCACAAGATATGAAAGAAATAATCTACAAACTTGTTGTGTCTTTCTTTTCTTTTGAAGAAAGTATTTGGATTAGGAATTTGGCAAACTCTAATATAAATAGCTAATTATTTAGATTATTGTATTAGAGGAGTTTTGATTTGACAAGAATTCAATTCCTTGTCACCCAAAATCAAACCATTCAACAAACTCAAATCTCTCAATAAACTGAACAAAAAAAAGTGTAATATAAGATAATAATTTCAACTTCATCTAGTTTATTAAATCGACTTTCTAAGTATTTAAAAACTAAAAAGTCAATCAAAACAGTTTCTAAAGTTCGTTTGTTTGGTGTGTAGATCAAA

**>CsLBD4**

CCGTCCATCATACTTGACTAAAGTTTAAACTAACGTTTCTAAGTATGTTTGGATATAAAAATTTGAAATAAATAAGACAAAACAACAATAATTTATTGTTTGTTTTCTAATAATAAAATTTTGAAAACATGCATGGAATATATACATAGAAGCATAAGGTTTATTGTTATCAAACATATTACTGTAATCCAATTCCACTAATATATGACTCATTGTCTTCCATTTATTCTTCAAATCTTGGTGTTCACACTCAGCGTAGATCTAAAAATTTATTGGTACCAACCTTTATAGCTTAATCAAAATATATATATATATACCATACTTCTTATAAGTATTATTACTTTTCTTCAAGTGAGATATAACGACTTCGGACGATTTTCTTTTAAAATATTTTTCAATTTAAATTTTAAATTAAATCTACAATAACATGATATTGTAATAAGAGTAATGAGAATAAATCAAGAAGATATTAACATATAACAAAAATATAACTTAAACAACTCAAAAAATTAATAAAATAGGTACAAAATAAATAAACTAATGCAAAAAGCTGAAATATCCAATTAGAATTTTAAATAAATGCATCTGACATCTGTATGTTCAATAAGAGAATAAAATAAATTACAAAACTTACCTCATAAATTAAGAGATAAATGGAAGAAAAAGCCATAAACAATAAAATAAATGAAGTTGAATAACTTTAATTTAGGTGGGGAAGAGTAAAAGTAATAAAAGAAATGAAATTGAATAGCTTTAATTTAAGTGGGAAAGAGACTATAATTATTATTTTGGGTGGAAATGGAACATGAGATGACAAGAAGAGTAACAACTCAACTAACTACCGATATTAAAAATAAAATAGTTTTTTTTATATATATTAAAAATTGAATAGTTATATATATATTATATAAAGACAATAAAGTTGAGTGGCTCAAGCCCCTCCTAGACCTATACTAAATTCGTCAGTGTTCACACGAGATTTTCAGACAAATGTAATTCGTGGAATTTAATTATGTATATTAATTGATATTATAGATACAATCTCTAATATTTACTCATTTAATGTTTTCTTTTAAATCCAAATTAAAACTTATAACTTTTTGATCTTCGGTCTTCGAGATGTTGTAGTTGTAAGTCAATTTGAGCTTCGATCTTTTGGAATCCAAGGAAAGTTTAATCTTTCAAGTTTTCAACCTTTTAGAAGATGTTGATCTCAAGCTTGATAAGAACTTGCATGTCTTGAATCTTCAATTCTTCAGAACTTCAGTACTTACTTCAACCAAAGATCTCCAAAATGAATGGAAAGGCTTCTATTTGTAGAAATTTTTCATGGACTTGGATCCATTTGTCTAGAGGGCTTGGGCTCATTTTGCATGTTGAGCTTGAGCTTGGACCGATTTACTTGGTGTACTTGAGTTCATTATCCTCGTTTGATCCAATTTTGTATCTAGAGCCTGAATTAGGTAGGGTATAAAAAAATTTAATTACCTAAAATTCAATTAAATTATAATTATTACCATCTCTACCGTGACAACGTGACATAATTTAATTGACCGAAATTTGTTGCAAATGACTTTTTGTCTTCCAAAAAAATAATTTTAAAAAAACTATATTTAATATTCAAAAATAGTTTTAGTTCTTTTAAAACGCCTACTTTTTGAAAAAATCACAAATGTAAGTTATAAAGTTTGCATATATAATGAATGATTCCTCTCTATTAAAACAATTTCTTATTCCTATCCTATGTAAGAAAATAGGTTAATTTTTTCAAACCCTTAGAATTTTCGTACAAAAAAGCCAAAAGTAATAATAATAATAAGACAATTTTTAGAAGGAAAGAAAAGTACAAAATACAGAATATAAAAGCAGCCAAGCCATAATAAAAATGAAAGAGAAGACGTCGTCGTTTTAATTAATTAGTTATTTCCCTTTGCCTATTTATTCTTAATTATTTGGTTTCGGTTCCCATCAATTTCTCCAATCTCTCCCCCGCCTAAACT

**>CsLBD5**

TCCACCATCCACAAACGGGTAGTTCTAGACACAAAGAAAGATCCTTGCATCATCCTACAAAAGAACAATTAATTATCTCAAGACATTACGCATGGCGATAGTCTCATTAATTGCAACAATGGTTCGTCAAGATTCTATCCTTTGTTTCAAGATAAAGTACTCTGATCTCCTCCTGAAAGTAGAATATGTTTCCAGTTGTGGATTGGAAAAAGAATGAATGAATAAATATTCTACAACTTCAAGAAGTGATTTTGATGATGTTGGTTACGTTGTTTTTATCCTATACACTAAAAAAAGGGTACTACAATGAGTACTTTTCTTTTATGGTTCAAATATAAATTTATGTTTTCCATTTTCTTCAATCTCTTAGTCTAGAAAGAGGGGCAGCATTTTTTTTCAATCCCTAGAATTTTCCTACAAATATTCCAAAATAAAGAAGGATGCCTCATAGATTTAAATTTTATAATTTTAATCTTTAACTTCTTCTTTTGGCTTTAATTTTAGGCTTATTTTTTAATTTCTTAACAAACATTATTAATAAGATTTTTAAAGTATACATTGGTATTAATACAAATTATTATTGTTATTTTATTAGGATTGAGAAATATTAAATTAAAATTAACTAAACGAGATTACTTTTTAAAATTGATTATTGAATTTGAGATTATCAAATTTTGAAATGAGAAAGTATATGAATATAGTGAGATCAAATAGATTGGATTTAAACAACCATGGTGATGATATTATAAAGGACAACATCTAAATTTTAGAGATGGATGATTATGATCATGAATAATTCATCCAAAACTATCAAACATTTCTCTTCAATCTCTTCGTTTCTCTTGATCTTTAATTTAAATCTAACTCATTCCTTTTGGTTATTGTCAAGTTGATTTGAGGTTTTAGAATTTGATATATCACAGATTCACCGATTTGATTCCTATTTATTAAACCCTATAGATTTCATGTCAATAATCGTGACACAGTAGGGAGAATTTCAACTTTCAAACTAAGTTTATTATGAAATTTTATTTTATTTTTATACATGAGACAATTAATACAATAATGCACATATTCAAAATTACATTGTTCGTTTGTATTAATGATTAGTAAAAATGAATATCCTTAGGATGTGTTTTTCTCAGGTTCATAACCTAATTGACTTCGTAACTTTAAAGTTTATATATTTAATAGAATATTTAAACTAGAATCATATTTCGTAAGTGAGCGACTAAAATGAATGAATATTTTGGTGTGGAAAATACAAAAACAAAATTGTAACAAACTCTAAATTAAAAAAAGAAAAAGAAAAATGATATTTTGATATAAATTAAAGTTTAATAGAAAATTAATAATTTATGTTATAAATGAAATTAATAAGGAGGCAGAGTGTATAAGTCTACAAAAATGAACTTAAAAGTCCAATTTTAATTTTTATTTCAACTTACGACGTCATCTCTTCTTCTTCACTTCGTCTCGTCTCGTCTCTCTCTATATATTATATTCTTCGTTCTTCTTCACAGCCAAAACCAACTCCATCTCTCTCTCTCTCTACTGACTCTAAAATGGAGTCTACTGACACAAAATCCAGCCCGACTTCAACTCTTCTCCTTTCTCACTCCCCGTCGCCGTCTCCGTCTCCTTCACCGCCGCCGACTGTTCAGGCCCACGTGGTGGTCACTCCATGTGCGGCCTGCAAGATTCTGCGTCGGAGATGCACACAGAAATGCGTATTGGCGCCTTATTTTCCTCCCTCTGATCCTCTTAAGTTCACCATTGCTCACCGTATCTTTGGCGCTAGCAACATCATCAAATTACTTTTAGTACTTCCTTGATCCCTAATTTTCTGTTTCTCTCTTTCAAATTTCGCTGTCTTTTCTTCTTGTTTCTATGTTTGAAAATTATGAATTCCGTTATTGAAATTGAAAATTGACCTTAACAACTTAACGAAAAACCATCGTTCTGGCTGGAATGAAGTTTGACGAATTCACAATCCTATT

**>CsLBD6**

CTCAAAGAAAAGTATAGATGTTGTAATTGTTGAAAGCTCTTGTGTCGTTTATGGGCATGGATGCTCACGTTATACAGTTAATTAAATGCACTACACGAATACTTTTAAATGCTTTTTGCAAAATAAAACCCAATTGAACGTGGAAGTTACAATAATTATTTGTTTTTCAAATCAAACTTGATACACTAATTTTTCCACATCTTTGGGCTTAGGATCATCAACTTTTTTACCCTACATTTTTATTGCCTCTGTGTAGTAGTCAGCTTAGTCTACCCATTCTATCTAATTTAGACTTAAAACTTTAACTCATTAAAATATATAACATCAATCAAATGTTTGAAGCTTCTTCATACATTAAAAAAAAAAAGAAAAANNNNNNNNNNNNNNNNNNNNNNNNNNNNNNNNNNNNNNNNNNNNNNNNNNNNNNNNNNNNNNNNNNNNNNNNNNNNNNNNNNNNNNNNNNNNNNNNNNNNNNNNNNNNNNNNNNNNNNNNNNNNNNNNNNNNNNNNNNNNNNNNNNNNNNNNNNNNNNNNNNNNNNNNAAAAAGAAAAAGAAAGAAAGAACATAAAAGAAACATTCTCATACATATATATTATTATAGTTTAAAGGCAGATGTAAAACATGCAATATGTACTTTAGTGATGCTTATTTTCAACTTCTTATTTTGAAATAGGAGACATGATAATGCCTTTAGTGTGATATATGACTAATTCCTTTGAAAAATATGCTTTGGTCATATCGTACTTCTAATTTACAATGCTTATTTATGGTTCTGTTAACAAAGAGCTAAAGGTACCATAGTAAGAATACAAACTATATATAATATATATATAACTCCTAGTAGTACTAAGTAGTCACAGCAGTAGTAATGTATTGTTGAGTTGTTAGTAATAGTAGTAGCAGTAGCGGTGTGTATTGGTGGTAGTAGTAGTGTATTGGTAGTTTATTAACCCTCCCTCAAAATCTAATGTATTTTAGTATACTAACATTAATCAGTAATCATTTTTAAGTACAACTTCATATATTAATATTGGTGTATCAATAATATATTAACACTTTTTTAAATTCACATTTAGAATATAACACCGATATTTTAATATATTAGCGTTCGATGTACTTTTGTAATTCAACTATAATAATTTATACTTTGTTTCAAACGCTTCCGAAAAAAGTGTTTTACGTATTTTTTGGAGCGTAAAGTTAATAAATATGATTTTTTTTTAATTATGGAATATTAAACATAAGTATTGGAGGCTGGAACCTCATGTAATATTTTTAATTAAAAAAAAAGAAGAAGAAAGATGTTTTGATGTCAAGTGTCACACTCACACTCATTGGAGGGATTACCTATTTATCAAAATATGGTCACAAAACGATGGGTTATTTCATTACTATTTGGTATAATTATATAAATAAGATATAATGTGAAATAATGATAATAATAATAATAGAAATTAAAAAGTAAAGTAAGTAAAGTAAAGTAATTCACACCGCATGCAAATACATCATAATGTTAATGTAGAAAGAAAAAGAAAGAAAAAAAACAAACAAAATGAATGTGTGTGTGGGTGTGGGTGCCTTAGCTTATAAAGTAATTCAATCTAATCTAATCTAATCTAATCACTTAAATAGGATCCCTCACGCCAACTTCTTTTCTTTCCTCTCTTTTCACATTAATCCACTATTTCACTTCCAAAAAACAAAATACCTCTAAATTCGTTTTTTATGTACTCTTACAAATTAAAACAAACTATATATCTTCCTTTTAATGTTTTTCTTTTCTGAAAAAAGAGAGGTGGGGTCCGTTGAAAAGATCATGTTCTTCTTTTTCTTTTCTGATGTAGGTCTTTGGGGAGTGGTTGGTTATTAGTTTATATAAATAGAGAGGGAGATGAGTGAGAGGGGTCAGTGTCCAAACTCCAAGAGGCAGAGGAGAGTGGCAGAGAATAACAAAGGAGGAGGGGGGGGATACGTGTGTCATAAGACTAGAGGCGGACGAT

**>CsLBD7**

TGTGATTTCTGTTAGAGTGAATTAACTCGATAAAGAGTAATATCATGGTATTAGTATATATATACCGAAGTTCTTAACTTCACTCAATGGTCATTTCTACCATACATAAACCAAATTAATATTTTTAAATGAATTATGTTATAGAATATTTAATATACTTTTATTTATTGATAGGGCTAATTTATATATCATGACATCACGTAAGACGATTTTATTTGAGTTTGAAAAACATAAATCATGTAATTATTCAGTTAATAATAAATATTATATCTTTCTATTTGTTGAAAATGAGTCTACTCAACTAATATACAGAATTATTATTAACTATGAAGTCTCCCCTTCGATCACATTGATGTGTTGGACTAAAAAAAATGATAATTTGTCAGAAGCCTTATATGTACACTTATATCTTGTCTAAAAATTTCCATAGTGAATTTAAATATATATACGAAATTGAATATTAAATAAACATTAAAAGAAAAAGAAATAATGAACTCATCTGTCTTGACTACAATCAATTGATGAACTTTCATCCCTAATAGACTAAATCCTAATCAACATGAATTTTAGCTAATAAGTCAAACCCCTTAATTTTCACTCTATCATCCCGTCAATAAAAAGCTATCTTATTTTTCTAATTTATATATCTAAAATTTCAAGCTTGAAACTTCTTAAATGTGTTCAAAGTTCAAGATTTAGGGTGGGCACAGGTGTCTCCAGACATAGGGGAAGGAAAACTCCAACTCATTCTTATAGAATAACAATAATTAATAATCTAATATATTTTGCTCTAAAACTCATGTGAAAGCAAAAATATTTAAAAAGAAAAAGAAAGAAAAAGGAAAGAATTTTTCATGCATGCATGGATGTAATGTAAGTAATTAGATATTATAAGTTTTTTTTAAAAAGGTGTTTGGAAATCCCGAGACATTGACAAAGAAGAAGAAGAAAAAAAGAGAGAAAAGATTGAGAGATCTCTCCCACAAGTTGAAGTCAAAGAATGATGGAGACAACTCTGTTTAGTCATTCTTATTCTCTTTTGCCTTCTCTCCTTTTTAGGAAAAATCCTTTAAATTTATCCCTTCTTCTCTCTTTCAACCCCCTTTTCTTATTATTATTATTAATTTTTATTTAACATTTACTGGAAAAATGGGACTGTACTTAAATATTTTCAACCAGATCTTCTTCTTCTTCTTCCCCCTCTCTTCCATGTTTTCTTATTATACTATTCATCGATCACAGTGAACCAAAAAAAAAATACTTTTCTCTCTCTCTCTCTCTCTCTCTAAAAACCCATCTTCCTTTTTGCTTCTCTTTTCTTCTTCACGAGATCCAACTTCTCAAAACCCACTTGTAAAAGGGTAAGATTTTGATTTCCCCCTCTTTTTCCTCTTTGGTTTCTGCATTTTTCTTCTCTCTCTCCTCTCACTTTGGTCTAAGGTATACGGTTCCTATCTTTTCCTTTGTTTTCACAAACCCATCTTTGGATTCAACCAAACCTTCTGCTCCTACTTTGTTTCGTCAATGGAAAAACAACCATGAAAATCAAGATTATTTTGTTCTATAGAGATATTCTCTGTATAAAAGAAAAATATTTCCTCACATAATCCTCTTTTGATTCAATTCCAATTCAGAAATAACTTTGCTCTTTAATCCCATGTGTTTTCCATATCCACAATTTATAGGGTAAATCTTTCCTATAGACAGCGTTATATGGAAATTTTCTTCTTTCTCTTGTAAACAGACAGACAAACAAAGCAAACAAGTCAAGTGCATAACATGTATAACAGTTCTCTAATCCAGATTTTTCTTCTCTTTTTTCTTAATGATGATTTTGAGTCTATTGTTTGATTGAAAAGCAAGCAGTCAAGTACATAACATGATAGTTGTTTAGTTCATAGTGATTTTTCCCCTTTTTGTGAACGAGTTTTTTTAGTGTATGTGCTTGATTGGTTATGTTGAAGTCTAATAGAGTGTTCTTACAGAGGAGGAATTGGAAG

**>CsLBD8**

TTTAAAAGTTTCTTAAAAAGTGGTCTATTAAGTTATCGAAAACTCTTATCAATCGTCTTATATGTTACTCTTAATAAGTTTAAAGCTAGCTAGAAAGATTGATGGCTTTAATGTGGTTTAGTTTCGTTGTTGGTTTTCTAATTTTATTTTTAGAAATAATGAATAGTGAATTATTTTTTCTTTAAAAATGAATGAAAAAATCACGATTGACTAGGGCTTTTATATTTTATTTTTAGTATTTTTTTTACAGGAAAAGAAAAACATATACAAGTTATTAAATAAGTCAATAATCTGTTATCTTACACAATATTATATTGTGATTTGTAAATAAATAATAATTTTCTCTTCTCTCTTATCTCCTAGTTGACTCTATCATGTATTAAATTTTTTACTAATAACGTATATTAATTAGGGTTATTTTCTTCTAACACAATATAAATTAGAGATATTAGATGCAAATTCTCATAACATTCATTAAAGCAATCTGCCGTATCCATATTTATTTATAGGGTGAAAACATTTGTACTTTAAATCCTTATTATTATTTTCCAAAAGAAATCATTTTTATTTTCAACAGTTTGTTCAAAATATTGTTCCAATTGGGATTTTATTAAACATATAATTGAAAAGGTTAGGTTTTTCGTGCACAAAGTGATAATTATTTTTTCTATGGTAAATTGAAAAGTTAAAATTATATATTATTATTGGTCATAAAATATAGTATTTGAATAGTTGAAACTGTTTTTTTCTTTTTCCTTTTAAATAAACGTTAGAGTTTGTGGGTTTTCAAGACACCAAAAATTAAAAGTCATCCACCCAATGAGGTTTCTTTGTACTTTCATTTACATAATTTAGAAAAATATTTCAAAATTTAATATCATTTATTAAATATTTTAAAGTACTTTTAGACTGAATTTCCCCCTCCCAAATGACAATTTATGATTAAAAAAACATCAATGAGAACAAAATGTTTAAAATATCAATACCAACAAAACAAAAAAATATATTAAAATCTTAGTTTTAACTCAATTTTAATGATAAAAAAAAAATGTTGATTTATACTATTTTATTTTTTATTTTATGAAATTTTCTACCATTATAGAAGGTAGGTAATGAAATGGAATATGTTTGTAATGCAATGTAATTCAAAATATATGTATAGATAACTCTCTTTTTCAATGTTTTCATTGTTTACGAATAAATTACATTTTAATTGGACCATTGCAAGTTATGAAACGCTAAAGAGAAAGAAATAGGGGTGAGGGGCAATATGGGTATTAAAAATGGACAGTACAGGGACAAAGAGGTAACTCAGAGACGCATTCAATTCCTGCTCAGAATGAGAGGTCCTTCAAAACCCTTTCGTTCTTTCTTTCTTTCTTTCTTTCTTTCTTTCTTTCTTTCTTTCTTTCTTTCTTTCTTTCTTCTTCTTCTTCTTCTTCTTCTACTTCTTTCTTTCTTGAAACCCCAAATCTCCCCATCCAACGGCTCTCATTCACCCCCCGACGACGCCACAGTTACCACCCTTCTCTTTCTTTATACAAATAAATAAATAAATAAATAATAATTTTCCTTTTTTCTCTCTAACAATTTTAATTTCTTTTTTTGCCTTCTTCAATCAATTAAAATAAATTTCCTTTTTCTCTCTTTCTGTTCTCCTTCTGTTTGGTATTGCGAAACTCGAAGATCGGATCCAGCGCTTTCTCTCCAAACCCTAATACCCAAAAACAAAACAGAAAAAAATTAAACATATTCTCCCAATCTCTTCCTTCTTCTAATTTCTTCATTCATGGACTTCAATCGCTCCAACACAATTTAACGCCGTCCGATTCATCCCAAAAGGTCAGTTTCCTCCCCTTTTTCCCTTCCATCTTTTTACGCCATTTTCATTCTTCTAATTTGCTTGGATTCTTGATTCTTCTTCTGTTGGTTCTTGATGAAAAATGTTACATATATATGTATAGAATTTTGTTTTTGCAGTTGGCTTTTCTTTTTAAAAT

**>CsLBD9**

GCTCAAGGGCATGATTCAAATGGACCCAATTGATTAGATCGTATGATATTGAATGAGCCCACACCATTTTGCTATTGAAAACAGCTCCATTGGGATTGTGTTGATAGAAAAAGTGGTAAATTCCCTTGTAGAACATTGGTCCTTTCAATCAACAAATTGCAAAAAAATCATACTTTTTAACGAATAATTAGGCAAATTTATTGTATTTTATTTCATGGTTTCTACTCATTCATCAGATGTTTTTTTTAGTTTCATTATCATAGCCTATACTACTCACCTAATATTGATAACCGTTTCTCTTTGTAAGTTTGAATTTCATATCGAAGGATCAAATGTAATTTGAATCAACACTCTTATTATATTATTGTCGATAAACTTATATTAGAAAGAGATACCTAAAGTTTAATTATTATACATATAGCTATAAAGCTTTAAACAATTCTTACCATTTGGGTCTTTACGATATCGTAAAGCATATTGATGATTCATCCATCAAATGAAATTTCGATCAAGGGAGAAAAAAAAAATACAAATAACAAAGTTAGAATATGTAATAAAGTAGAGATCGATATTCAAACGATTTTTTTTTTGAGAAAAAATAGCGAAACAAATAGGAAGAATATATACATTGTATGTATATATGTATTACCATTCATCCAATTTTTGAGGGGTTGGAAGTGATAAGATGTCCTTTGAACTTGTGGAGTATGAACTTCATTGGCATATTCATGAGAAACCACAAATCCAATTCTATTGCATGTAAGCAAAATAACAAATGATACAACTAAACCATACTTGCCTATTTCTAACCTTTTTGGTGGCATTTTATCAAACAAAAGATTAGTAGTATTCATTTCAAAAAACATGCATACAATTCTAGCTTCCATTACGTTTATATATATACACACACGATAGTTCATCTTGTCTAATCTAAATATAGTTTAATCATTCTAATATCATAATTTCAAATAAAAGACTAGAGATTCAAATCTTCCTTACGGAAAAATTAACATAAAACTTAAGAAAACATTATCATTAACATTTTTTTTTTCTAATTGATTAGATTTTCAAAATATAAAAAAGTTGAAAAAACCTTACTTTTATTTTTAAAATTTGTTTGGTAAGTTAGTTTCGTCGAGAACAATCATCTCAAATACCCAATAAATCTCAACATTTCCGTGAACAATCATATCAAATACCCATATCTAACTTTTGTAATTCAACTATGAATGCGAACAAATAAATATATATACACTCGTGTTAAAAGCAATTAGAGAAAATCATTGAGATAACCTAACATGTTAAGTTTGTTACATTGATACAAAAGGTAATGCAAAAAAGTTGCAACTTTTATCATCCAATACATACTTACATTATATTGCCAACTAAGTCATTTCATAGAATAGGCAAAATTATTGCTCACAAACACTCATCACCCCAACCCACAAAATTGTGACTACAAAATGTCTATGCAAACACATTTATAAGAAAACCTTACTTCTTTTTTTTAATTATATCAACTTTTAACTTTAGCTTGAATTTATTATTATTTTATACTATTTATGTGTATCAATTAATCCTTTAATGATATGTATCATTTAAGTGTGCTCTAATGTTGTATATGAAAAAAATGTTTTTCAAAAATACATTATGTAATTTTACCACTTCCTTAGATCGGTTAATTTATTTGATAAATCTTTATATACACATAATTTTGTTTAAAAGATAAAACGATCAAATATTTGTTATATTATAATCGAATATATTATGGACTCACAATTAATAGCATTTTTTTCTAAATAAATTGTTTTTGGAAAATAGAAAATTAAAATTTAACCTAATTGTTTTATTATATATTATTTTAAAGTTAAGAAGCGGAAAATATTTTAAAAAATACCTAATAAGTTGCGATCGCAAAGGAAAAAATCCACTAAGGAAAGTGGTTCAGTTACGTTCTTTCTCTCCTTCTCAGTTTTTTCTTCTCCGACTTTTCATCTGCC

**>CsLBD10**

TTTTCTCTTCCTTCTTCGTTTTACAGTTGTTGTTCTTAATGAAAAATCGTGTGATAGAGAACAAGAGGAAGAAGTGAGAAGCGAGAGAAGTAATCTAAAAAAGAAAAAAAAAATCAAATAACTAAATTGTGCTTGTGTTTGAAGAGAATTTAAAGTGAGATTAAAAAAAGCGAAGAAAAATTATCGTTGCGTTTGATGAGAAATAAGAAAAATGAGCTAGAAGCTGTAACGAGAAACAATAATTATTTTAAGCTGTAAACCCTCACATGCAGTTTATGGAGAATGTGATACTTTCACACGCTACTGACTATTCAAAATGTCATTTTAAAAAAACATAAAATTGAATATTGGATCAAGTTTTATATGGACTTATATAAATGGGTCATCCTTACAAGAACTCCATTAAATCATTATCTTGTAAAAATCTTATAGAATCAAAATAAAATATTGACATCGATGGATATTTCTAGAAAATGTATTATGATAAACGATAATGATAATAAATAAACATTTTTTAATTTTTAAACAGTTACGTGTGATGTATTATATTATTCTTGCATTCGTTACATATTCTTAATATATATATATTTAAAAAAAAATGTTAAGAACACAACGAGATACTACAAAAACACGTTGACAAACTTAAATATTAAATCTAAGTCATTCCTAATTAGAACTATACAATTTGTTTCTTCTTAAGACAAAACAATTACAACTTGTAAATGGCTCCACCCACTTGTTTATTTCATTTAGGGTCACAATTTTGCTATTTATATTACTCGTTCTTGTTTCTCAACTTTTGAAATACATATTGGGGGCAGAACCTCATCATCAATTGTAACCTTATAGGCATGTGTGATCATGTATATAAAAAAAAATGGGCACATTACTAAGATCAATAATATCTAATAACAGTGGTATATTATTCCATACAACGTCCATGGTACTATCACATGCATAAAACAACACACTATACTGATACAAAATAACTACCCTTCAACCACATTACTATATTTATTTTCTACTCATTTGATTTAAGTTTCGATGAATAACAATTATCTATTTGAAATGTTTACAAATTGAAATACAAGTGTGTGATTTCTCAAGTTAATTACATACGATTCATTGAACTTACAAATCAATCAACTCATTATATACAGATATTAGAATCAACAACTCCAACAACTTATCAATATAACGTACACTCATGAAACTAAACTCAACGGATTAAAATAAACAACAACTAGAGACTAACCAAAGTACGAACACAACACAACAACAAAATTATAAAAATCAAACTTTAACCATGTAATCAAATTGCGTAACAATTTATTAACCACATGAAATTCACATTTCATTAGTCATACATAACTCCTAAATAGTATTAAAACAACACTTCGATTAATATTTAATCACACAAATAAATTCGATTCACTCAACGACATGAGGAAAAACAACTAAGTAAATGAAATATATAAAGGAAACATTTAGGAAGGAAAATATTAAAGCCTCAAATTCTTAATTTACTTATTACCATAACCAACCACCTAAGAATTTCCTTCTAAATCATAATTTTTTTTCAAAAACTAGCCGTTGGACATCTCATTTCAACTCAATCAAAAAAATACAACATGTGTGTGTGTATATATACACACACAACAAAAAGATTTGCAAAATCTACACAAAATCTTCTCTAATATTTTCTTCCAAAACCATTTTTTGTCTCAAGATTTTACTTATGATTCCAAGTCGTTGTGCAGCTTGTAAGTATTTAAGAAGAAGATGTTCATCAAATTGCATTTTCTCTCCTTATTTTCCCTCAAACAATCCTCAAAGATTCGCCATTGTTCATAGAATCTATGGTGCTAGTAATGTTGCTAAATTCCTTCAAGTAAGTACATTGTTTTCATTTATTGAACAATTCATTAAATTATAATCTAAATTAAAAATCTTAGACTCTCTTTTGGGTTAATTTAATTTAATTTAATCAGCAAGTTCCT

**>CsLBD11**

TGTAGGTTGAGTTTAACCGAACCACTATAAAATTACGGTGTCTTCTTCTCTAACTCTTTTCTAATTATTTTTTAATTTAATTTTAGTTACATATTCTTATTGGTTGAGTTTGATTGCATACTTCCATTCATATATTTTTATCAATTTTGTTATTTTACAAAGTTTACAAAGTTATTTCTTTAAATTTAAAAAGTATCTAATTAGTTGATTTTACTTTTTATTTGTCAAAAAGTTTATTTAAACCCTATTCACCCCCCTCTAGGGTTGCCATACCGATCCAACATATTGATTGTATTGTATGTTTTGATTAAGAGTAAGTAAACCATTAGTATCATATTCCTATAAAAAAAAAAAAAAAGCTAACCATTATGTACACATAAGGAGTAGAGGGAACCATGTAGAAAAGAACTGCATGGTAGAGTGAAGTTAGCCAATGCATGAAAGGAGTATTGAGCTTAGTGGCCTAAGCAGCGATAAATGAACCAAGAAAATTTTTGGATGTTGTTCGTGAAAAAAACATCACAATAGTTTAAACGCGAGATGTTGAGTCAGAGAACTTTTCTTGAAATCAAAACTAATGAGAAAAAATTGTTAGTAGACAAGAGAAAGTATTAAAAAGTAGGGAAATTGCATCAAATGACAAATTCTTTTAGAAAAAGCAGCTCATGGTACCATAGGGTTATCGGTTTTCATGAGTGTTAATAAAATGTTTTTCTTAGACTTTGTCAACTTTCCTTTAAAACGATATATTATCATTCTATCTAAAGCTAATGTATCAAACATTTCAGAAACAAAAAATTGTAAATGTAATATGCTTGAAACAAGTTTAAAGTGCGTCCTTAAAATCTCTATAATAGTTAACAACATTCCAAATTATTCATTTGAAAATAGTTAGGCAGTCAAAACTTATATATTTGAAATTTTACTTTATTCATAATGAGTAATTAGACATCAATATAAAATATGATAAAAAGTACTCCCACTTAACATTTATTAATTTTCTTGCCTGTAATTATTTAGTCAAAGTTATGGGGTATAAGTTTATATATCAAACCCTTGTATTAACAAAATTCTAATTAGTCATTTCTCAAACGAACATATATATAAAGTCGTTCTAAATTCTAATTGAGACTTGTTTATTCTGAATTCATGTTGGGTTTTATTGATTCTTGGGGTAATCTAATGTTCTTCCATGCTTAGAAACTACTTTAGTTTGGAAATTAAACCTAGGCTTGAATTGCTACTTGGGGAGACCAAACTGATCAATTTGGTTTCACAAAGAAAATTATGTAAAAAATAATTAGTCTAATTAGTATTGATTATAGCACAAATATACAAATGTCAAATTTACAAAAATATTATAGTTTCAATTAGCAATAATAGCATATTCTTTTGAAAAAATTGACAAGATATGAAATGCTAATTGAGATATTTGAATGTAATGTTTTTTTACCATTATTTTTTTGTTCACAAAAAGAATAGAATTAGTTGGGGTTTCTTTTAATCCTGATGATAAATAACCAGTTAAATAAAATTCACGCATTAATTATCACAATACATATATTAAAATATTGAACTCCACATGATATAGGATGAAGATAGATAACTGATCTTCCTTCCCAATGAGCTACCTAAACCCTAATTTAAACATTAAAAGAATGCCCTTTCCTTTTTCACCATATTATTAAACAAGCAATAGCCATGCATCTTTTCATTTACAAACCAATTTGGTTGTCCTTCTTATCTTGTTTTGTTCATTATTAAAATCATACACTCCCTTTTAATTTCCATTAACCATTAACAACTTCACATATATATTAAGTGTTAAAATTTTAAAAATTTGTAAGAAAACATTCCGAACCCATTTATAATATAAATTCATCAATTCTCCTCCACCATGCATTAATTCTTTTTTTTTTTTTCTATGCATATTCATATATGCTTCTTTCCCCAACAACAAGAACACCAAATTTTCATCTTTGTTTCTTTTTCTCTCAAAG

**>CsLBD12**

TTTGTACATTAAATTTTCATCTAAAATCTATCTTAATCTTTGCCACTAGACTTTCATATTTTATATGGAAACCTTATAGTTGAAGCCCCATATATTAAGGGTTTGTGTTGAAGTAGAAAACAAATGACTTGAAAGCTTGTGTTAAATACAATTAGAGATATTCAATAGGAACTAGCAAGTGTTAAGGACAATATGACACTTTTTCAAATAATAAAAATCAAAACAAACTAAAAGGAGAGTTTCAAAAAACAAATAATACATCACTACAAATATCTTCATTGGATTACTTTCGAGTTAAATTGCAAAAACCATCTTTAAAGTACGTTGGTAGTTACAATCACACCCTCAAACTTTCAACAATAACAATTAAACTCCTAAATTTGTGTTAAAATTGAACTCTCAAATTCACGATGATTCATATAATTACGAAAGTGTAATGGTCAAACTTTAAATTGTGGATAAGTTTTAGTGCTCAGTTTTATAATTACGTAAGGGTGATGGTCTAATTTTAAATTGTGAATAAGTTTGAGTGTTCAATTTTAAAATTTTAAGAGTATAACAGCAATAATTCCTCGTAAGTTTAGGGATACTTTTGCAAGATATTTTTGCAAATTTTGTTAAAAATAATTTAACTAGTAAAGTCTAGAAACTTATTTTCTCATCTCTTCCGATATATATTTAAAATATTTGATGTCAAGTTGTCCTTTGTTATGAAACAACTTGTAACTTTTTTTAAAAAATAGTTTTACTCAAAAGATTGAAAATAATTGCAATGTAATGATAGTCTTAGTCCAAAATTTTGGTGATCTCGAGGGTAAATCTTTTACTAATCGGCTGAAGAAAGCTTCTAAGATATCACACAATTCAAGCAGCAATTTATGTACAATCTTACACTACACACCAAATATAATATACATTAAAAAAATTTCATTATACCAAATATTTTCTTTTTGTCCCCGGCTCTCTCTTTTCGGGTACCATTGCTGAAGTTGTAATTACACTACTTGTAAACTTTCAATCGTAAAAATTAAAACCTTAAAATTTGATTAGTAGTAAAATTAGAACCTCAAACTTAGAAATTATAACAATTATTTAGGCTAGATTGGATAATCAATCAGAGTTTGTAATGAATTGTGATGAAAAGTATTTATAAAGGAATACAATTAATAGCTTTGGATTGAACAGAGAGTCAAGCCAAAAAACAAAAGATGTTTCTCAAATTGAAACAAACAAATAGGTAAATGCAAATAGATGGATCATTATTAGATTAGCTAGATCTAAATTTTGCGTTTAAATTTTGCTATAAACGCAAAACTATTATCTAGTTTTGTAGCTGTCTTATGCATCAATGAAATTTTTTCAAAAATAAAAAAAATGACAAATTATTGAAGAAAACCATTAAAACACAATTTATTAAATATTTTGTCAAAAGTTCTTTATTTGACAAATTCGTATATTTTAATATTAATAATGGAACAATAATTGCATTGCCAAAGAGACCTATCCTATGATACCTATAATCCATCTCAAATTAATTAACAAGTAGGGGCTAACATTTTCTATATTTTCTAAAGTTACAAAATTATCGATAATAAGTTTTTATTTTATTTAAAAAAAAGAAACATTATTCGAGGTCCTCGTACCCCTCACTCCACGCAGATTTAAAAACACAAGCAGTTTGGCGGAATTTCATAATATTACTACAAAAAGAGACCTATGCTATTCCAAAATAATAATAATTATTATTGTTATTTCAATAATAAAACTATGTTTCAATCTTCCAAAACCTACATTTCCTTCTAATTTGCAAATCCCATCAAACTAAGAGAGAGAGAGAGAGAAATGTAAATGGAAAGAAAAGAAGAATTATATACCTTGTGATCCACAAACCTCAAAGTCAGCTCCACACATATATATGTGTGTGTGTATACATATTTGTCTTGTCTTCCAGTAAGCTTTGCTTAGGTTTTCCTGAAAGAACTCAAGCAAGCAAGGTTGAG

**>CsLBD13**

TTCTCATAGCCAACCCAATGCAAACCCTTGATGAAAAATCGAGCCAAACAATGGGCCAGAGCCCGCTAAGAAAATGAGCTCAAGTCCAAGCCCAACAAGTAAATGGGCCCAAGTCCAAGCCTAACAAGCAAATGAGTCCAATCCCATCTAAAGCCCATGAAATTTCTCTATAAATAGAGACTCTTGTCATTCATTTTGAGAGATCTTTGGTTGAAGGAAGAATTGAAACTCTGAAGTACTGAAGCTCTAAAGAATTGAAGATTCAAACTTCTACAAGCTCTCAAGACGTGTAAGTTCGTATCAACCTCGAGATCAACAACTTCTGAAAGATTGAAGACTTGGAAGATTAAACTTTCCTTGGATTTCAGAAGATCGAAGTTCAAATCGACTTGCAACTACAACCCCCTGAAGATCGAATATCAAGAAGTTCTAAGATTTAACTTGTATTCGAGAGAAAGCATCAAATGAGTAAATACTAGAGATTGTATTCACAATATTCATCAATATATCAAAGTTTAATTCCACGAAATACATTTCTTCGAAATCTCATGTGAACACCGAGCGTTGAACTCTCTGGTGTTGTCATGATGAATGTTGCACGAGGGTACTCTAATATCCGGACAACGGTTTAACTCTCTCTTTTGCAAAATTGCTCTGATACCATATCAAGTTGAGAGAGGTATGTGAAGAGATTCTTTTATTAATTTCACTTACTTTATTTACGGCTTTTTATAATAGAACTTTGATACATGGAAGTTTAACTAACAACTGTTATTTACAAACATAATTCAACTAACAAACTGTTATAGCTACAAACTGTTACAACTAATTAACAGTTTTATTTGTTAATAAGAATGTATCATTGTGTTAAGTTAATTTATGAATATCTTCGTGTATTAATCTCTTTATATATGATGTTGTGAACTTCAATCAATAAGATCCATTTTAGTTTTCATAAATTAAACTTATTGGTATTACGGTTTTTTTCTTGTTTATTTTAGATTATTTTAAAATAATTCAAACCCAGCTTTGAAAAAGAAATGAGACATTTTCAAAATATAACAAATTAAACTAAAATATTTATAAAATAGAGTCATTTTAAATAAATAATAAAGAAGAAGTATAGACATCTATTCATGTATATGTATCTATCAATCTAAAGTTATGCTATAATATGAGTGAATTCTTAGATTTATTTTTTTTTTAAAAAAATGCATTTCAAATATAGAAAAAAAGAGGAGAATTTACATAAATAAAAGAAAATGGAAAATATTTATATCACAGGGATAATTTAGACTTGGTTAAAATATATGTGTTTTTATAAAAATTGAAAAAAATATACGTATAGTTTTGGTTAAATCATGTTATTTTAAAAAAAGGTTTGTAGTTTTCAAATAAATTTTTTTATAAAATTGGGCTGCAAATTCAAATATTTAATCATAAAAGCTATGGTAAGAAGTTGTAAAAAAGATAGCAAAATTAAAATAATTAAAATAAAATTGGTAATCAAATGTTGAATGTTCTCCTTATCATTCTCCTTCCAACTCAATCTTGAACTTCCATAATCCATAAAAAATTTGAATACTTCAATTATCAATACTAAAATATTCAGAAATCTGAAAGCTTTGTGTTTCCTTCAAGACCTATATATTTTGTATTAAAAACATTAATTAATTTATGGTTTAAAATATTTTCAGAGAAAACAAATTCCTTTCACAACCCTTATGATTTATGGAAGATAAGAATATTTAACCAAATTGTTTTTTGTTACCATATCTACACAATTACATAATTAAACATTTCCTTGTAAACTATATATCACAATTTGAAAAACTAGCCGTTATCTGTTGTCAATCAAAAATACAACTCTTCATATTTCACCATAAAATGTATATATACACAAAAGATTTGGAGAATCTTCATCCAAATCTTCTTCTCTCTTACTTTCTTCCAAAATCTTGATCTTGTCAAATAATATATATTCAATTTGGATTTTTAGT

**>CsLBD14**

AATGGACATGTTTAATGAACTTTTACATTTAGTTACATTTACGAAAATTAAGTTGGTGCAAAATTGTATGAATAGAGTAGGAAAGGAAAGAGATAGGAAAAATATATAGGTTTATTAATGAATAAAATTTAACATCTAAAATGGGAAGATTCCCTACAAAATTTAGAAAACCCAAAAACAGAAATATGATTGAGAAGAAAAGTAATAAAAAAAGAATTAAAAAAATAGTGTCTTTGTAAAGTGTGTTCACGTGAAGGGCAGAATTAACAAAATCCGAAATAATAGCAAATAGAAATAATGATAATAGTTAGATGATAAAATGGAAATTAATTAAAAAATAGAAATTAGAGAAAGCAACACGACTCTTTTAAAAATGAATTAATTAAAAGAAAGAAAATTTGAAACTGTTGGCTAAGCTTATAAGCGCCCACAGTCCCGCACTATCCCACAAAACCAGCAACCGGTTTTGGTTTCATCCAATCCTGGTTTTCCCTAATTTAAAAAACGAAAAGAAAAAAGAAAATACAAAACCAGTTTTCACCGATCTTCCTGAAAACCAGGAGATTTTTCTAATCTTTAAACATATTCAATTTTACTTTTTTTTTTAATTTTTATTATTATNNNNNNNNNNNNNNNNNNNNNNNNNNNNNNNNNNNNNNNNNNNNNNNNNNNNNNNNNNNNNNNNNNNNNNNNNNNNNNNNNNNNNNNNNNNNNNNNNNNNNNNNNNNATCATTATTATTATGTTTTTGTGGGACCCAATTTTATTCCACGTTATGTGGCAATTCGACCATATTTCTGATTACCTCAACGTGGTTGCCACGCGCCTTCTTTTTTTCTTTGGATTTTTTTGCATGTTGTCTCTCTTTCTCTTTTTATTCCACCTGTCAGCTTCTAGATAATTTTATCCCAATTACTATTTTACCCTCCTGAATTTCCGTGCCACTTGCTTGGTGACTATCCTTGTTTGTGGCGCGTCGGACGAGGGCAATTTAGAGAATCCAACCAAAAGTTCCAAACCAGTTTCTATGTTCTACACCACAAAATTTACTCTACTCTTGTTTTTTTGGACGTTGCTCTCACGCGCCTTGGATCTCTCGTCCTACCATGAGATTTTCTTAGATTTGCATGTTTACCTATTGGATTAACGTTTTTTTAGTATTTATACTTCATAAATTTTAATTTTTGGAGATCTAAAATTGTCTACTTTAATTTCATTTGATTGCTGTGAAAAGTCAATTCAGAGAGAAATGATTTCTAGTGTTGTCGCTAACTATAATATTCATGCTGTTAGAATACCTACGAATTAAAATGTTAAGGGATAAATCTATCAATCTAACGATTCGATATTGATATTAAATGTCGATGAATATTTCAAATATCTTTTTATAAATAATCGGAAAAGAAAATATGTGATCTACTTAGTCCAATATAAATAGAAATACTAATAATTAAATTCATTAAATTATGAAACAATATAATTGTAAATGAATAATAAAAAAATGGAAGTATTGTCTGTTAATTAGAATGAGGATCATAAAAGAAATAAAAATGGAAATGAGATTGAATAAAGCGAGTGGAAGAGAGGGAGAAGAAAAGCGAGTGAAAGTGAAACGGAAGTAAGCGTAGTATTTGGGTGCTGGTAGAGAGCATGGAAAAGTGGGTTAGAAATCCAACCTCCACAACTATGGAAACCCCGGTCTTTAAAATTATTTTAAATTTAAAATCCACAAATTACAATTATTTAATAATGAGAATATTATTATTATTATTATTAATTGGAAACTGCGTGCTCCCATTTTCCCTGAAAAAACCCAATGGCTCCACCTTCCAGATTATATATATACTACTTTCCTTCTTTTTCTCCATTAAATATCCTCTATACCTCTCTACTTTCTCTTTTATATCTTCCAAACCCTTTTCCCCCAATTTTCATTCTCTCTCTCCACACTTGCTTCTTCCCATTTTATGCTTCCCTTCTCCCATTGCTGTTCTTCTTCAAA

**>CsLBD15**

GGTCTACATGTTGGTTAGCAGGGACAAACATAAGAAAGTGACCAAATTAAATTAATTGAAAGTAAAAAAAAACCAAGTTATTATAAATTTAAATTAGATTAGTTAATCATAATTGTGGACTTAGTGGTCGACATGTCGTGCCCTCTCACACTTAAACAATTCAATTTCATTTTTTCTTCTTTTAAACTTTTTTTTGTGAATTAATGTTTCACATAATTTATTAATTAATTATATATCATATGGTTTTTTTAACTGTTGTATTTCAGTGTCACCATTCTTTGTTTTCTTTAGTTTTGGAATATACTTCTCAGCTTTTTTATTCTTTAGAACTTCCATTATCTCAACATGTTTCACCATATAATAATATATATTTATTACAAATTCCCCATCATTTACTCCATATCTTCTACTTAAAAACAACACATTTTCAGTTTATTAGGGACTTTTTTTTTTAATTAGCTTCAATTTGTTTGGTGAATTAATGTTCAAACTCTATGGATTTCTTATTATTCTTAACTTAACCCATCAACATTCACCCTACTACAAATAAAATGCATACTAGATAATGTTTTACATTTTATTATAAGCTTTCTAGTTTGTATGTATAGTAAGTTCTTGAAATTTCAGTTTTATATAGGTTTATAAGTCATTGTATATATATTGACATTATTATTTTAAAAATGAATTGATTTTGATTCACATTTAACAAATTTGGGTGTTTAATAGTTATGAATTTTTTAAAAAAAAAGTAAAGTAAAGGTCAATTAGTACTTAGGCCAATATGCCTAGTAGATACGAGATTGAAGAGTAAATAGTCTATTACAAATAATGATAAAAATTTAGGTTTGTTGTTGATTAATATAATAACTATTTTCTGATGAAAACGTTGGGTTATATTTATTTTCTTATAATTTTCATATTGTTTAGGGTAACATTTGAATTCTTAGTCAAATTTCAAAAGAAAGAAAGTAGTATACAAAACTTGGTTGTACATTCTAAACAATATATTATTATATAACAAATAAATTAAATTTATGGGGTGGTGGAATTAACGTAATGTTTATAAGTTTAAGTTTTAGAAAACAAAAAATGATAAACGCTCAGTATGATCACAATTTGATTTTTTAGTTATATAATATTGAATTATGGGCAGTTATTTTTCCTAAAATTTTAATTTAAATAAGAATAGCGTTTAAATTAAGTTTTTTAAAAAAAATTCAAATTATTATCACACATGCCAAATGATTATCAAATAAGATTTTAAAGTTATACCAGGATAAGATTTTCATGAATTAATTAAACATAAAAGTTATAGTTCATATGTACTCACATATATGTAATCATACAAAAACTAATACACTCAATTTCATTAGAACTCTACCGCTTACAAAAAGAACATACTCATCAGATAGACTGCATAACTCTATTAGATAAACATAATTAGTTTAAGTAGGAGGATGTTTGGCCAAATGACCTTTTGGTTTTCAATAATAAAAAACAATAATTTTATACCTTATTAACTCTTCAAATTTACAAAGAATAGTTCAATTTGCTCCAAACGTCGCAGTGAAGAGAAAGGAAGAAAAAGAAGAGGTGGTAGGAGGGTTTTTGTTTTATATTACATTTATACTAAGGATAAATGAATTAATTAAAGAAATCCAATCATAAAAGAAAGCATATTAAAAAAAGGAGTTTAATCTCTAAACAATGACAACAGCTGATATTTTGTCGTATGGAGATCAATATTAAAGAGAACCCATCTCAAAGATGAATAATAATAAAAAGATATATATATATATATAATATATATATATAATATAATGAGAGACATTTGTTGAGAATATAATATAAAAAATGTTGTCCATTTAGGGAACCGAAACCCATTTATCATAATTTTAAATTCTTTCACTCTTTCTTCCTTCATGTGTTTATAAAGTGCAATAATCATAATATGAATCCCTTAAACTGAAAACTGAGAACCAAAAAGATCAGAAACAACA

**>CsLBD16**

ACTTGTAAACTTGATTGATATACTTAATAATGATTTACTTTACTGATATGTTGATATTACTTTACTAATATATTGTTGATATACTTGATAATAGTACACTGAGTTATATCGTTCATATACTTAAGAATGCATTGGTTTATACACTCGATACAAAATTTATTATATTCTTAACCATAACTACAATGAACTGCAAAAATTTTGAAAAAAAATGAAAAATCATGTAGTGAATATATCAATCAACTAATATATATAATATAAGTATATCAAGTTAAAATGAAAACAATCACAATTGGAGTTAAATTTCTCATATCAACAACCATTATATATCAAGTTACAATTAATGTACCATTAGTATCCTAAAATCAAGATTTAATACAATAGAAAAGAAAAGAATTCTTGAATGCCTAATAGTAGTACATCCATTTCAAAACCAGAAAGTAAAAAGAAGCAAAATAAAAAATAAAACTTAAAAAACTTTTCAAATCAATGAAAAATACAAACAACCGAACACTTAAAACCACAAGGACGAAATCAAAATCAGGACTCCATGTTCATTTAGGTTTCGTATGACTTGACAAACAATTCACAAATGAAGACACCTTTACTAATTTTGAAAATCAATTAAAGTCTATCGATGAGGAAGAACGAATTGCAAAGAACGTAAGAAAGGATAGTTATACTAAAAAATATGGAAGAAAAAATACGTTAAGAAAAGTTTGAGAATAATAACATGTTTAAAATAAAAAAAAATTTAAAAGAAAGAGAATTTTGTCATATTTACAAAATTAATTAAACCATATATTATATTTGCTATATATTATTAAAGAAAAACTACCCACCACCATTTTTGTTTTTAGATAGAGTGATATTGAAACCTTAATAGAACCCATAAAATATATTCGAAAAACATTCACATGATATATTCTTACTTCTAGTTCAACCCATTAAATTTGTAATAAGTTTTCATCCACTAAAAGTAGTATGATTTAGTTAAACTTATGAAATTTGTAATAAGTTTTCATCCACTAAAGTAAGATGGAGCGCCCAATATGATAATGTATTCTTAACATTATTATTTAATTTGATTATGCAGAACGTACCTGATGTTTGGAACATATAAGTATAGTTTTGTTTGAATGGTTGCATATATGTTTGGGTAATAATAATTATTAAGAAACTTAATCGCTAAACTTAATGTGTTTTCAATTATTATGTTTCGAAAACAAAGAAAGAAAAAGTATAATAAAAATAAAAAGAAAAAGGAAAAAACTGGTTTAAATTTAATGGCTGGTGGGGACACATAAATTTCTAATTTATAGTTTAATTAAAAAATTTTAAAAAGCGGAAAGCGACAAAACGCAGCAATTCTTAGGTGCGGCGTTCGGCGGAAAACAAAATTTTAATTACTATTTTATTACAACCCATCAATCAAACCAAAAACTATAACATTTTCTTTTTATTTATTTATTATTATTATTCTTTTTTTAAAAAAGAAAGTTTTGACTCTTCACCCATAACCAAGAAATTTAATAGGAGATCCATTCACCAAATTTTAATAAACATTTTTATAATTTATTAAACTTATTGCGCATATATATATATAGTAGAGTAATTTTATTGTACGTTTAAATCTCATTTTTATCCTAAACTTTAAAAGAAATTTCTATTTTAATTTAGTTTTTGATTTGTTATATAAACAATGCTAAAACACATATACATTTAATAAAATTAATTGGTATAGATTTACACAACAAAAACTAATATACATGTCAAAGAGGGGTAGAAAGAGAAATATGTATTAGGATAGTACTAAAATTAATTGGAGTTTATTTGGAAGAGAGATGTTCCCTAATAACACAACACACAAACCTTCCATGTCTCCCTTTCACACTCCTTTATATAAGAAAAACCACACGCCCACTCCCTTATCCCCTTTACTCTCTTCTTTCTTTGCACTTTTTTCTAGTCTACTCGTGCACGGCCATTTCTTCTATTTTCCC

**>CsLBD17**

TAATTTGTGGTTTGATTTTAATTATCGTATAGAACATACATGAATAATATAGGTTTGAGTTTTCTTAGCTTGTAATAAAGAATCAATTAGGGTTTGAATCTTAATCAAAAGAAATGAGTTTTGAATCAGTTTAATTCTCTATCATATATATAATTATCATTCTAGTAGAAAAAACTCAAGTAGGAGTCTTTAGGAAAGTAGGATATATATAGGAATATATTCATACTATTATAAAAATTATTGATTTTTTCTTCCATCTACTATTTTAGTTTTCTTTAATTAATCATATTTTTTAGTTAACATTGTTTTGTTGAGTGGGAATTAGTGTGGAGTCCACTGGATCAATATTTTCAATTACATGCATGCTCCCATCTTTCTCTCTTCTGCCATTTCAAAGAAATGTCAAACCATATATATATACATATACTCATGTATTGCTTTCACAAATTAAGCCCTCATTTCCAAATCAAGTCATCTACACAAACGCACGTCTACGATCTTTGAAAGATATAAAAATTTCACAAATCTGGATAGTTTCGCAAAACTACTACATTATATTTAGAAAATATTCAAAATAAATAAACCATAGTGTGAAAGGACGGTTTTTTTTCTTTAAATTTTTCGTTTCCCAAGTTGATTTCTATAGAATAATTAATTAACGTATTATTGGTTATCACCATTTGTTTTGCTTCTCAATTAAGATCATCTCACCTTTCAATTATCACTAAAAGCACTAAGTTCATGTTGACTTGAGTGTAGATACGCAAGCTTTCACTCCAACCTTTATATTAACATGTAAAAAAAAATGTTGAGTATCAAAATTTATATAGCTTTCGATCTCATGAAGGGAAGACATTGTTTTGATTATTACTAAGCAATTTGCTCTATGTTTGACCAATTGACTAAGAAATTAGGAGCTTTTTGTACTGAATATATGATCTTAATGAAGTATAATAATCCTTTGTTATATTGTCAGAACACATAAGGACGTGTTAATTATTTCTATATTTCTCTATGAAGTTTTAGTGAAAATGTAAGGTTGTTTGTAAAATAGTTCTCTCACACACGTAGTTCTTCCTCCCTCGAACCCTCTTGTGCTCCTCATCCACTTGTACTATAATGCTCGTGTGTTCAACAATAACTAATCTAATACAATAATGCTCACACACGCCGAGTGCTTTCCTTCACTCTTCCAATGTAATTTTTCACTATGATTTCGTTGCTTTCTTTTTTAGTCTCTCTCATTCACCTTCCCCACTTCTTTGTGCTCGTATTTACCACTCTTTAAACCCCCATTAATATTGGTGCATGATGTGCAATATAGATAATTAACGCAGTATTGATACACTCAAACCAACGATAAACTTCATTATTTTCAAAAACTGTAGGTAGTAAATGACAAACCATATTTTGAAATTTTACTCATGCATTCATACAAAAACTCAAGAGATTAAATGGATTCAAATTCTTTCATCTCTATTACGTTTGATGATCCCACACGTCAAGGACGGCACAAATGTTGTGGTTCCTCATTTCTTCATTATCATCACCATTAATTGCCTTTTAAGAAAACTAAAGATAGCTAGAAAAGAAAGAAAAGAAAGAAAATCACTCCCTTTGCACGTGCTTCTCACACCACCTTTCACCAACCCTTTCGTATAATTCATAACGTCTCTCTCCCCCCTCTAGAAGGTGAACTTATTGTCACTCCCTTTGCACGTGCTTCTCACACCACCTTTCACCAACCCTTTCGTATAATTCATAACGTCTCTCTCCCCCCTCTAGAAGGTGAACTTATTGTTATAATTTCAAAATAATAATAATAATTCCTCATTTTCGCCAACCCTCTCTCACGAGACTATTGCTTCCCCTTCTCTTTCTCTCTTCCGTTTTATTTCCATTACTTTTTCTTTAATCTTAACCTTATTGTTATTAATTCAACTAAATATTAACAATCCCTCTAATACCCTTTTTTTTTTATTTCCCTCCATGCACATTAC

**>CsLBD18**

ATGGCTTCAACTTCATTTTGCTTAAGGTTTCCATACAAGAGATGTTGAAACAACAGCTTACAGAAAGGAGAGAAAGGGGAAAAAATTATTGGTAACAAACCCTTTTTAATTATTTGTTTAATTTCGATCAATCTACTTATTTTTCTTTTTTTTTTGAAATTAATTCATTTTCTCTCTCACATCATCATCAATATTGATCTCTCTCTCCTCTAGATCTCCTCAACTCACTAATTGAGTGTTTCTTAATATTTTGGGGGAGGGGAGTTTTCGTTAGATTAGAACGAAACACGTCCAACTATCTCTAATTACATGAAAAACCTTGAAACTAAAAGTTAAGCTACGAGACGTGGGAGGCGCCATATAACATATTTAAGGTATTTTTTTAAAAAAAAATCAAAACTTTGATTAGACTTTGATGATCTAAATGAGTGTACGAGAGAGAGAGAATAGGTGTTGGTGGGTATGGAAGGGAGATGATTATAGGGAAGGGGACCTTTTAGATTTGATGAAGAGTAGAGAAAATTCAAGTTTGGTGTAGGAGAGAGAAAAGGAGAGAAAGAGAAGACAAAGAGGTAAGGGGAGGAGAGAGAAATTAGGGGCATTTTAATTAATTTTGATTCACTTAGGGACAAGAAACATGCATTGAGGGCTATGTTGCCCTTTGCCTTTAGGGATGCAATTAGGGGAAGGCCAAGTGACCTTCCACCAAATTTTATACAAAACCCTCAACTTTCAACAGGGAAAACCCTATCTTTTTCTTCTTCTTTCTCTCTCTCTCTCTATTTTTTTTTCTTTTCAAACTATCATGTGTTTAATTTAGAGCAATATATATGAAAATTGCAAGTTATAACATTTCTAGTTTTACCACTTTGATAAAGCTTTGTAAATCAAAAGGAATATATATATATATATATATCAAACTTATGCACATATGACCAATAAATAGAAATGTGTATGTTTAAATGTTTGGTTGGTGGGGTAAATAAAAGCTTGTGTAATTAGGTAAACTCAAGATGAAAAAAAAAAGAAAAAAGATCACACCCTTGTCATATATATATATATATATATACATAGTAAAAATTGAACAATGAAAAGAAAGATCCAAACTTCTAAAGTCAAGTGTTCATGCTCCATACTAGTTGAAGCTATATGCTAAAAAAAAGCAAAATTTACATATATTTATTTCGTGGTGAAATTAACTGTTAACTATATTTATACGTAAATTAAAGTTTATTTTATTTGATATTTTCACACTTATTTATTGGACTCTCTCAAATTCTATTATGTTCTTAATCTAGTTTGAGACGATGTTCAGATACATAATTACTGAGATATTCATCTCCTTCATTGATTTCTTGTTAAAAGAAAGGCTGTTTGATCCCAAACCCTATTCCCAAATATTCCAAATGAAATTATGAGCGAGTCAAATAATCCTAAAAGATTATATACGTACATATATGTATATGTAAGAAAACAATACAGAAAAGAGTGTGTGTGGTCTAGAAATCACCTAAGAGATTAACCCATAATTAAATGAAAACTTAGTTATAAAAGAAGTCTTCAAATGGTAAGAAAGACGGCTCTGACAGATCCAAAAGAAAGGTACAAAAAAATTAGGATGTCGCATAGCTGTAACATGAAAATTCAGCAGTTGTTGTATTGTATCTTTGTGTTTTTTTTTTTCAAAACAATTTTCATATTTAAAACCATCAATTTCTGGTGATCTTAAATTAATAAACTACTCCTTATCCTCTCTTTCTCTCTCTCTCTAAACCTATACAAGATCTTCAAAATTCAGCCTAAGATTCATCGAAGGTTCAACTTTTACACACCCCCATCTCATAAGAAATTAATCAAACCTTTTCTTCTTATACAACTAATTTTAATTTTCTATGTAATATGTTAACAAAGAAAATGTAATTCTGATGTCTATTTGTTTCTCTCTTTGTTCTTTGAGTTATCTTAATTTCCTGTTTTTAATCCTTTTCATTTGTAGGAAGA

**>CsLBD19**

AATTAAACTATACTTTTAACTATGATTATTCTATATCAAAGAGGGCATATATGAATAATAAAGTTGTCACCTTGTGCTTCCCTACTCCAAACCCATTAATACTCTAATAATAATAATGATAATAAATCTCTATAGAATAAAAACAATAATTTAAAAGTGAAATTATGGTGCAAAGTAAAAAAAAAAAAAAATGGAGAAGAAAAGTAGAGTTCAGAGAAAAATAATAAATCCACTATAATAAAAAAAAGAATAATAAATGAACACAAGAATTTTCAATATATATATATATATAAAGATACGATCTTATAAAAAGTAAAGTGCTAAAAAATGATCCAAAATATAATAGGAAAGTTTTCAATAGCGGAAATGAAAATAGAATGTCAAAAAAGTATTAAAAGGGAATAAAACATATTTTAAATAATAAAGGCAAAATAGAATAAATGAAAAAAAAAAGTTTAAATATTAGCAAATTGAAAATTAAAATAAAAAAAACCCCAGGCAGCAATAACCTATAGTGTGTTTAACATAATTGCAAAGTTTTTAGATGAATAATTTGAAGATGACCTAATACACTGACAGCTATCTGTTAACAGCTTCATTTACGTCAACAAATGTATTTATAAAGAATCGAATGAATTACGATTGGACTAAAATTATGTTTGGTTTTGTTTTTTTTTTTTTTATAAATATTTTAAAGCGAAAGATTTGATTTATTAATTGAAAAGAGTTGAAATATTTAAAGTGATTATTGTTTGGGTTTGAAGTTGAAGCTGAAATAAAGAAGTGAGTTAAGTTGTGGTTTGAGGAATTGATATATAAGAGGACAGGTAAGCATTGAGTGAGAACCCAATAAAATAATTGTGAGAGAGAAAGAATAAGTAAAGAAAAGTAAGTAAGAAGTGGAATTTATTTAATTCACCTTGGAAGTAGAAACTCGTAAAGTGAAGAAGACACGTGAGTAACATGTGCCAGATACCACGGCTGCGAAACCAATCCTCAATCCTTCGGTTCCCTCTTCCCTCTTCCCTCTCCACTTCATCCTTCATCCTCTTCTTTACCTCTTCTTCCAAACTCCTTGTTTTTTTTTTTTTTTAATATATAATGTAAGTTTCTTAATCTTTTTATAATATATTAATGAATAAAACAAAACTGTTTATTTATAATTCAGCGATATTATATTGATGGTTTAACTTATTTAAAAAGTGTTAAAATATAATAAATTTTAAATTGTTATTGGTGACATTTGACTTTTAAGTTGATAAGAAAATTGGGGTGTGCACTTTGTATTTTTCAAAACAAAAAATATGATAGCAACAATAAATTAGGTCTTTTTCTTTTTTTTTTGTTCTCATAGAAAAGTCACATCAGTTTAAATTATTTAATATAATTAATTTAGAATAATAAAAAAAAAGGAAAAGAATAAAAGAAATGAAAATGGAAAGAAAAAGAAAAATGTATTAAAATAATAAAAAAAAGAATCTAAAAAGAAGAAGAATATGGGAAAAGCAAAAAAGAAGAAAGAAAGAAAGAAAAGAAGAGTTCGCGGAAACACGGAGCCGCTCTTGCCCAGAGTTTGGAAGGAAGATATTATTATTCTCTCCCCCCCTTCCTAAACGCTACAACATATGGGCCCCACTCATATCTGACAACTGGTCCCCACTTTCACCCCAAGAAAGTCCACCGTCAACACTCTCACATCAAACTTAACCACAGTTACTTTCTTAACCCTAACCCTCCCCGATGTCCCAATGCCCCCAATCTCTCATTTCCTCTCTTCTTTTTAACCCTCTCTTCTCCCATTTCCCACACTCCCCTCCGCTCTTCCCTTCACTCTTCTTCTCCGTTTTCTTCTCTCTCATTCTTTTTTTTTCTATTTTTCTTATTACCCATTAACATTCCTCTCCTCCTCATTCCTTCTATCTTAAGAAACCTTTTCTTCTTCCTTCTTCCAACCACATAAATTAACACACACCTCTCAAATATTATTACCAATTTCAACC

**>CsLBD20**

TAGAAAAAATGATGATTTAATTTAGAATAGTGAATCGAAAAGCAGAACATAATTGGTAATTACTAGAGAAATTTCGTCCCAACACGATTTACTCACCCATGTTAATTATTGTTACTTTTTCTTCTTCTTAACGTGGGGGACTAATGATAATTTACCATGCATATAATAATTAGGATTAGGAACAGTAATTTTAATGTCTATTTATAACCATCCACTAATTGATTAATTATATATGAAGAACCAGTCAATACTTAAATTGTAGCTACTTTGAAGAAAATGATGTAGCAAAGTGGACTGAATTAAAATGATCAATTATGATAATATAATTTATAAAGTATATTGGAGATTACACTATTACTGCCCAAGAAACCAGTCCAAAGCCATTCTACCAAGTTAGAAAATCTTTGAATTATTTTTTTTCAAATTTATTATTAGAAAATACATATATGTGGGTATGAAAAGTAATATATTATCATAAATTAGGAACGATTATTTTATTTTATTTTAATAGTATTATAATTATATTTATGATTACATGAAAAACATTACTCAACCAAATCAATTTTTATATATATAATTTGAGCAATTATAAAAACAAATGTGATTTGTCCATCAAATCATCCAAAGAGAATAATTTGTTAACTTAAAGTTTATTGCTTTAGCTTACTACTTTAATGAAATAAAGTAAGAAGTCAAGTAAATTAGAAGCAATATCTTTCTTTTTTATTTTTTTTTATTTTTACTTTTATTTTTTCTCCAAAGAAAGGTAATAAATTATGAAGTCTTTATTAAAAGTAAAAGGGTTTTATTTTTTTAAAAAAAGTGCTTTTCAAAGGTCAATTGTCCCTCTCTATAGGGAATTAGGCTCTTAGAGCTAAAAATCAATCAAATATATTAATTCCAATGCAATCGAGTGGATTTATAAATTTAAGTTTACTTCATGGAAAATAATATGTAATAATAATTTATTTACTCCCAACTTTCCATTTTCTCTACTTTTGCCTCAAACATTAAACATAGAAAATGGTGAATATAAAAATATACTCAGTAAGGGATCAACTTCCCTCTAAATAATTTTAAACCTTTTGAGGGTCAAGATAAAAATATTTTAGGTTAAAATACTAATTTGATTTTAGTATTTTAGAGATGGATTAATTTACGATTTGGAAATCGGTGCAAGGATTCACAGTGGCATAATCTGGATCAATACTTTTCATAAAACTTGAATTTAGTTTTTTAAAATTAATATTTATTAGAAGTTAGATAAATTATAGTAATATTTATTTCATTACAAAATAGATTACAAATTTCTTTTATTTTTTAAAAAATCAACAAGAACTTAAATGAGAAAAAAAAACATATAATTGGAAAACTTTGACAACCTCTCATTTTAATATGATACACATACTATTGTTTTAAATATATATATATTTCTTTTGGAAAGAGTACTCTTATATATTCTTGAAAATTTGTATTTGGTTAATCACTCACTGATATATATANNNNNNNNNNNNNNNGATATATATATTTAACTTTTAAGAAGAAACATGAAGAAAGATTGATTTGTGTTTGTATTAAAGGAATGAAATTAAAAATTAAAGAGTAAGACATTTAATAATGGCCCTTTTCTAACTAAGAAAAACAAAAGTAATTCATAAACAAATCCTTCAATAATTTTATTTCAAATTTCTAAAAAAATGACGCAAGCAATACGTAGGAGACAAAAGTCTTCCTCTCCCCCACTAAATTCCTCTTCTATAAATATCACCTTCTCACCCTCTTTCATTTCCCAAACACACAAAAACTCACCAAAAAAGAGAGAGAAAATATTCTCTCTCCTCTCGCCCCCCACATTCTCTCTCTAGAAAATTTTAATTAAATACATATATATATATATATTTTTTTAATTTATGGCTTACGTGGCCAAATGTACTAATAATTCTCCTCCAACTCCCATCAGTGTTAATAATAATCCAAATAATCCTTTATCTTTCTCCCCA

**>CsLBD21**

ACCCATCTCTCCTTTTCTCTTTCTCTCTTGATTTTGACTCAAAAGGGCTTGCCAAATTGTATTTTGCTTAAAATGGCTCTTTTTCCGATGACAACAAAAACTTATATTCTTGTGCGGGTGCAATGAATTTTGAAAGCTTTTACCTATATATGATTCCATCAAACACAATCAACATTATCGTCTCCCTCCTACAAAATCTGAAAACCAAAATTTTAAAATTAAAACTTCAATAATAATATGTTTTCAAATACGTGTTTAATTGGTAAGAGATTTAGGATTTTACCTTTAATTTTGATTGACTAAAAAATAGTGTTTTTAATCGTCTTAAACCCTGTATTTACGGTTGTTTACGAATATTCTCACATTCACTTATTATTAAATTTAAAAATGTTACACAATTTAATTACATTCTTTTAAAATCATATTTTCTTTATTCAAATTTGTTACTTAAGAGTTTTTAGTATAAAAAAAAATTAAAATGAATGACACCAGACAAAAGAAATGATTTAAACCTTAAATCTTATATATTCGTTCAATTTTTTTTAAAAAAATGTTGTTCGGATTTTTTTTTAAGGCTTAATAGTTGGTCTATATATATTTAGTTTAAAGTTACGTATGATGTTGAATTGGAAAATCATAATTATTGGATCAAAGTGAGATGGGATGAGAATATTTGTCCCTATATCCTTTACTTCAACCACAACTCCAACCACTGACCTAATCTCTATTTTTTTACATTGTACCCTTAAATTTTTAATAACAATAATTTTGGACCCTTTGAATTTATAGAAATGTTAAAATTGACGGAACTCTAAACTTACTGTAATTGTAAAAATGGAATAATTATCAATAAAATATATACAATTATGTAATGATTGAGGTTCAATTATAAAATTTGAATGAGATTAAGGGTTTAATTTTTAATTTTGAAATTTTAAGGGTATAGTTGCAACTATAGATGGTTTTTGTAATTTTTTCAAAGATATATGTATATAATATGTATCTTTTGAGTTTTTAAAATGTAATGGTTCATATGCAGGTGTTATAAGGAAAAATAATAGGGTTTTCTATAGACAACTTTATACTCCTTTTCCCTTTTATGTAATGTACATATTTTTACTCATCAAATGTTAAATTGTTAGAGTTTATTGTGGTTGAGATGTCTGATAAAGAAAACACAAATGAAATAGTTAACTTAATTAATGAAAAGGTTAAAATTTTAGCTCTTCTAGAGTCTTTATCTCAATAATGGCTTGGCCCCCAAAGGGGGATTTGAGTTTCTTAATGCATTAGGTAGGTTCATGTTGTGAAGCCAAAGTGAGTGCAACTAAAAATGGTATATTGTACCTATGTTAACATCTATAAAGTATATGGTTTGAATCTCTGTGATTTCTAGTTTTCTATTACATTAAAATAACTAATTTTGGTATATTGTATTTAGTTTTGTTACGTTGAAATTATTTCACAATATCAAACATACAATTGATTTCTATAAAAATAATTAAACTCATGTTTAGGAGTAAATTCCAACCCGAAACTATTTTAGTCATTTCAAAACTAAGAAACATGTCTTTTAACCTAAAAACAAACAACACTTCATGAAGTTAGGGTTATGAAGATAAGAATGATGTCCAAGTAGGTTTTTGAGAGACTCATAATAATTCCCATGAAGAATCTTTCGAAAAATAGGCTCTTTTAAGGCACAAATTGTGAAAAAAAAAGAACCCTTACCCCAATGTGAGACTGACCTTGTAGCCCATAAAGGAGACCCCAAAAAATAAAGCACATACACATACACACAACTTTTTGCTAAAAAAGTTATTATGCCTTTGGAATAGAACAAAAAAAAAAAAAAAAACCCTACCAAAAAAAATAAATAAAAACATACATACAAAATGCAAATGATGGGTTATATAAAGACCAACATCTCTTCTTCTTAATTTTATCAAACTTGTTCCTTTTTTTCTTGTACTTTAAAGTCTACCATAATAATTGAGACA

**>CsLBD22**

TAATTTTACCATTTAACATATACCAAAAAATTAAAACCCTAGATTATTAAAAATGATTATCTTAATTGGCATCCTAGTCATCCAAAAGTCCACCTCACATTTTGTTAGAGAGTTAACAGAACCTAGTACGTAATACGGACCAAAATATGTTTAATTTTTTAAGTTTAGGTATTAAAATAAATACTTTTAAAAGTTAAAGGACCAAAATAAAACAACACATACACATTTTAAAAGACTAATATATAATTTAAACCTTAATTATATCATCTAAAAAAGATCATAGTGGTAAAAAAGCGAGATTAACTCAACTGGTCAAAATATTACTTGCATCTTTAAAAGTTGGAGATTCAAATTCTCGGTCCACGTATTTAATATCTATATATATAACCATTTTTTTCAATACCTATGTATATAACCATTTTTTTCTTGAGAACTCCATAGAAAGAGAACATCTCTCATTCTAGATATTACCACTTGGAGAGAGAGAGAGTAGAGAGCATGTTTCATTCAAAGAGTCACAAACAAATTACCATTTTGTATTGAAAAATCAATACTTTCTAATGTTTGAATTATTAAAAAACAAGATCAAGTGAGATTTCACTAGAAGCTACTATCCACTAATTTTCAAACAAATTACTTCAAAACGCAATTAACCATAAATAATAATAATAAACAAAAGAAAATAATCACATGTTCAAAACTTACCTGCCTTGATGACCACAAGGCTCTTCTCCATTCAAACAAATGCTTCTATTCTTAGTTAACATATAGACAAAAATAATATCCGTCCTATATTTTTTACGTATATAGTTCTTATATTTTAAGAGCGTATAAAATTAAAGACTAAAAAGTCAGCATGAGCTTAGCTCAACTACCAACTGTGTATTTATGTGGACAAGAGGTCTTACATTCAAATCCTCACCTCAACATTTATTATAATACCTTCACAAAAGAAAAAAAAAATTAAAGACTAAAATTAAAAGTATTAGATTCAAAATATATGAATAAAAAAATATTTTTTAAATAAAAATAAATATAAATATTTTGATTATCCTTTAATCCTTCAATTTTAAGATTTATATTAATTCAATTAGTTGTTGAACTGATACCAACTTTATTTTGTTTTGGATAATTTTTGAAATTTTAGTTTGAAATTTTAGTTTGAAATTTTCAGGACAATTATCCATAAAAGTTAATTTGAAATATTGTAGGTGAATTAGTTTTTAAAATTAATGGTAATATACAAACATATACTCCTTTTAGTACAAAAACTTTTAAGTAGTTCTAGAATTAAATCGTGAAAGTAGACACTATATAATAGTGTACCCCTTGTAGCTCACATATTATAGACGAAACCTATATTAAGGAACATATCAAAAAGATAATGAACTCCATGAAGTCAGTGCGTATATATGTAGTTGAGCTCGTAACATACTTAAAAGGGTGTGTAGAGCAAGTTTACATGAGATGAACAAAATACACCTCAAGATGAATAGCTAGGCAAATTTGTTCGTAAAGGTTATTTTATGTCAAAATATGGATAATTATCACTAAAAATTTTGTTGTATTAAAAAAAAAAGTTAGATATTAAAATATTTTAAATATGTATTTAGTAGTTAATACAAAAATTAAAGATGGTAGGGTTTGAAGAATTAGATGAGATGCATACTAATTGAGTAATTTTTTTTAATTAAAAATGGTGGAAACAAATATTTGGACAAATAATTAATAAGAAAATAAACCCTCAATTATTAACTAATTATGATTACTATTTTCTCTGAGGCAATTAATTGATATTATAATAATTAATATATTTTAATAATCACTCACACTTTGGGCTCACACACGTTGGTCACCCATTATAAATATACACTCTTTAACCACTTTCTTCTTCAACAATATTGACTCTTTCTCAAAGTTTCAGTTCTAAAAAAAAACTCTCTCTCTCCGCCGTCTCTTTCTCCCTCTAAGTTTTCTGTTCCCTGGAAGCAAAAAGAAAT

**>CsLBD23**

ATTCAAAAATAGTAAATTTTAGATGTTAGATATGCAATTTGTTTATATAGCTGGTCACTTAAAAATACAACAAATGTATGCATGATAAAATATGAAATTAAATAAGATTACTTAAAGATGAGAGAATCGTCACTTAGTCATAGAGATTGAGAATTTACAAACGAAAGAAAAGTTAATAGTTATTATTAGTATAAAATGATAAAAAACCACAAGTCTCTTTCTGGAAAAGAAAAGTGTAATTATTATAAAATATATATATATATATATATATATATATATATATATATATATATATATATATCTTGAAAGGGTATATTGTCATTTTCTTTGTGAACATCTCTAAAAACTTGTTTGAGTAATTTTGTGAAGAAAGGTTCTTAATGAGTTGGATGAAAAAGAGGTGAACTTCATGAAAACCAATGCCCCAACGCATTAGAAAATAAAATCTTTTTGGAATATTATAAATTCCTAACGTGTAGAACAAATTTAAATAATTATCTAAAAATACATAAATTTATATCATTATTATCCAACATATGAACCATAAATTGATGGCTAATAACTAAGTGTATAACCATTTTTAAAAATGACACAGAAAAAATTTATCAACTTCTATTAACGTTAACATTGTAAATATTTTGATTCATTGGGAGAGACGCAACCTTTCTTTCCAAAGGTTGAGGAGGTTGAATAGCTTATTCTATATATTGTACTAACAAAAACTACACAAAACTAAACTATGTTTTAATTAAATACACAAAACTTAATTATATAGATCGTGCCCTTAAGGCATATTCAATTTAACAATAAAGTTTAACTCATTTTGGAAAAGAAGAATTAAGTGTTTTATTTGATAATTAAATTCAGAAAATATTATAAAAAACTGAAATATTATACAAAAAAAAATATTAAATTTTTTTGACAAACTTACTGGAATAAAATCAATCAAAAACAAAATTTATTAAAAAAATCAAATGTAAATATTTTGTCCACTTTTTTCGTCCATTACTTTCAAAGCATCTATTTTAGTTCGGTTATATTTCATTTTGGTTAATGTGCATCTTCATTTTGTTTATTTGAATCAAAATTATTATCACGATATTTTCTATAAAACATTTTTCTAGATATAGTCACAAAATTAGATTAAATAGCTTTGGATAGTGTGCAAATATTAGAAGGAAAGTGAACATTTTAGAACTATATAAACAAAAATTAAATACTTAAAACCAAAATTCTAAGAACCAATGAATGAAATTAGTAATGAAAGAAGATAATTATGAAAGGTTTTGACCCACAAAACTGTGCTTAGAAGTAACATTTAAATTTTTGTGTTGTACAAAAATAAAAAAGAAAGAACCACTCTCTTTCGTGTATACATTGCGGCCTCTCTACGGTGATCATGCCCAAAATCCAATTTATAAGTTCTCTATTTCTTGATTTTGACAACAAACTAACATTTTCTCTTTTTAATAATTATATATTTTCTGGTGATAATAAAATTAAATTAGGTTTGAGACTTTGAGGTAATTCAAAGAAAATGAAAACACTTTTTATTTTTATTTATTTTTAATTATTTGATCTCCCTAATAAATAAGCAAACTCTTACTCCTCTCACATGCAATTAATTGCCAAAGTCAAAACACTCACACGTGAGCCCATTAATATTACTTTTGATTTTCTTTTTTAGTTAAGAGTTTAAAACCTCCAAACCAATAATAATAATACATAGTAATTACGATTGGAGTTGAGCTATATATGTGTGTGTATATATATATATATATGTATACATTGTATTATAATTTTCTTTTGCTCTCTCTAGATCTTAGGTGGAATAATCCTCGTACATAACCAATAGCTATTAGAGATGACCCAAATCCTCATATCACCATTCATTACTACAAACTAGTTATTTTCTCTCTTTCTTTCTTATTTCTTGGACCTTTTTCTCTTTCTACTTTTCTGGCTAATTTGATCTCAAGTGAAGATTAGAGCATGCTTGG

**>CsLBD24**

TACACCTAATACATCCCTTGATACACCCAATACNNNNNNNNNNNNNNNNNNNNNNNNNNNNNNNNNNNNNNNNNNNNNNNNNNNNNNNNNNNNNNNNNNNNNNNNNNNNNNNNNNNNNNNNNNNNNNNNNNNNNNNNNNNNNNNNNNNNNNNNNNNNNNNNNNNNNNNNNNNNNNNNNNNNNNNNNNNNNNNNNNNNNNNNNNNNNNNNNNNNNNNNNNNNNNNNNNNNNNNNNNNNNNNNNNNNNNNNNNNNNNNNNNNNNNNNNNNNNNNNNNNNNNNNNNNNNNNNNNNNNNNNNNNNNNNNNNNNNNNNNNNNNNNNNNNNNNNNNNNNNNNNNNNNNNNNNNNNNNNNNNNNNNNNNNNNNNNNNNNNNNNNNNNNNNNNNNNNNNNNNNNNNNNNNNNNNNNNNNNNNNNNNNNNNNNNNNNNNNNNNNNNNNNNNNNNNNNNNNNNNNNNNNNNNNNNNNNNNNNNNNNNNNNNNNNNNNNNNNNNNNNNNNNGTAGGGCTTTAAAAAGTGACAATAAATAAATAATGAGTGGAGTATTAAAAAGAGGAATAAAAATAAGTTATTGAAAAATAGTTTAAGAATAATAACAAATTTGAAGTAAATAGTATTTTAAAAAGTAAATTTTTTATCATGTATCTTGGGCTGTAATTGTTGTTATCTCAAAATGCTATCTATATAGTTTTCCAATAAAATAAATACAAAATTAAGTAACTAAAATAGAAATTTAACTAAAAATAAATCTTAGAATTCAATCATATCATATTAAATGTACTTTTTTTTTTCTTTTATCTTTGAAATGCATATTTGAGATACCAAAAATGGTAAAAGTGAGACAACAAACCAAAAATAAATAAGACTTTTACAAAAATAGCAAAAAAAATTAGGATAATTATAAAGTCTTCACAATATTTTTTAAATTTGAAAAAGTAACAAATTTAAGAGTGATGACCATTTGAAGCCCTATGATTAATAACTTATAATTAGATATTACTAATTTTGTTATATTTGCAATATGCAAAAAAATTGATGTCATGAGTTGTTTTTTCTCTAAATGTTTTTGTTATTTGATGCAAATTTTCAATAAATAATACATAAAAAAGTCCATAAATATTATATTCATGTTCTGTGAATTCATGTAATTATTATTCTTTAAAAAAATACTCTATAGATTAATACATAACAAAACTATCTATTAAACAAGTCATTATCAACTCCAGTCCCTCTACGCTCAATAAATCTTAAATTTGGTCTCTATTGTTAATTTATTGTAACTTTTTTACACTTTCTTTTTTGAATATATATATAGATATATCAGACAAACTATATAAACTAAAGAAACTACGATCAAACATTCTCATCAACAATAATGGAGATGTTAACAAATTTGCTCATGAAAGTTAATATTGAAAATATTTGAAAGTATTTTTTTTTGTTATTTAGTGGATGAAAATTAATTATACTTTTAAAAGTTCTGACATTAAAATTAGAACATAACAAACGACATAAAATTTCTATAGTATCATTGCTCTAATTACTAATATTGATATTAATATTGATATATATATATATATATAAAAAAGAAAGTGTAAAAAAGTTACAATAAATTAACAATAGAGACCAAATTTAAGATTTATTGAGCGTAGAGGGACTGGAGTTGATAATGACTTGTTTAATAGATAGTTTTGTTATGTATTAATCTATAGAGTATTTTTTTAAAGAATAATAATTACATGAATTCACAGAACATGAATATAATATTTAAAAGAGTTTTGGACATATACTAAATTCCATTTGTGTGGTCACCATAAAAAAATAAAAATTAAAACAAATGGCACAAAAATATTATTTTAAACATAAATAAATGTAAAATTAAAATAATTGACAAACGAACATGTGAAAAAGTTGATCTATAAAAGACAAAGAAGAGTAGTTTTATTCAATATTCAATATTCTCCCCCTTTTTCATTGATTGCTCCATACGACAGCAGCACT

**>CsLBD25**

GAAAGAAGGATCAAAAAGCTAGGTAAACCCTAATTCTAACTTTTTCATACTTAATTAATTTTATGATATGTATGTATATATATTATATATATTATGCTCAACAAGAACTTTGTAAAGATCTCCTTGATGGTTTTATTTTTTCCCTTTTGAGATTTTTTCCTTGGAGTCACTTAATTAAATTATAATTACTTCATTGAAATTATTTTGATAATTTGTACTTGTCCTGCCTGTTTACCTAAAAAAACTATTAAATTGTAATTAAGATGAAACACAGATCCTGCAGAATATGTGTTTATTAAATACTACGATTTATAATGTTTTTTCTTTTTTCTTTTTTCTTTTTTCTTTTTTCTTTTTTTATAAAAAAACTAAGAATTCATAATGTTTTAGTACAAAGGAAGGTGTGGGATTTTTAATGTTTCAGCTGAAACAGTAATGTGTGGTATGTTTTGTACAGTGAAGAGAAATAAAAACTAGACAATATAATTTAAATTCCACAAACAAAAAAAAGAAAAGAAAAGAAAAGAAAAGAAAGAAAGAAAAGAGTAGGGTGTGTCTAAGAACAATTTAAAGTGTATTGTATTTGTGATGTAATATTTAATAAATTATGTACTACTAAATGTAGCACAGTAGGAATAGACCTATCTATTTACTATTGTAATATTATAAATATACAGCAAAAGAAAATGAAATTAATTTACATTTTCTATAAAGTTTTATCTCAAGATTAGTTGACAAATAAGAAGATTAATTTCATGCATCTTATAAAAATTGTGATATATCGTAAGTATCTAATTGTTATCTGTAAATGTGTAACATGATGAAGATATATAAGGCAAAAACACTTTTAAGAAACAGAGAAGAAAATCAATAAGGGAAATCCACTCATTCGAGTTAGAAAGCACTTTTTCTTCTATCTTGTATTGATATACCTCTATCATCAATATTCTTCTATCACAAATCTATGAGTGATAGGACATTATTAAATATATACTTTTATAAGGGATAAAAATTTATCAACGTTTATTATCGATAAAATATGTAATTTTATTATATTTTATAAGTATTCTTGTCATTCATAATATTTTTCCATTATTGATGTTTTTTTCTTCTCTCTATAAATTCTTCTAGATAGAAAAAAGATTGAAACTATTTTTTAAAAAAAACTTAGGTGCGTCGGGAACAATTTAATAGATCATTTTACTATATTTTGTATGTAAATAATTTTTTTATATTATCTCACGATTCCTTATATATAAATACAAATTAAGTAAAAGACAATTATAAAGCAATTAAATTAAATATAGATTGACACCAAGTCCCATTAAAAACCTAATTTAATTCTTTGAAAAGTAATCTTTATTAGTTTAGAAAACCCAATTTAACAATGAGATTATTGGTGTCTCATTCCCCAACCAATTTGATATGGAATTGTACAGTGTAAAAAGGGTTAATGCAAAAGCAAACCCCATTTGTGTACTTCAGTAATTTTAAAAAGGATTTGGTTTAAAAAAGTGGAGAAGGGGATGAATGAACCCTTCCTTATTTGCATACATTTCTACCCAAAATATTATTCATATGCAACAAAAGATAAAGCTTTTTAGATTTATGTTAGCTACTTTAGGTACAGAAGTTATTCCATCGTTAAAAAAAGTTATATTATAATTCACTATTGAAAGCCCTTACCAATTAAAAAAAAAAAAATCATATACAAACTATACTGATAACTACATTATTTTTAGGTGTTTTTTTTTTAAAAAAATAAAATTAACAAACTATTTATACTCGATCAAAATAAAATTTATTAAAGTTGGTTTTTATGTGAACGGTAAATACATATCTGGTCAAATATTACATTTTTTACAATTTTCATAATTTTTTTTAAATGCTTAATTCTATTACAAGAACAAAAGAATAAAATGATATTGGTGGATATTCTCCCATATAATATATATTGTTTGGTTTTGTTGATATAGAAGGAAAAAAGAAAGAAAAAGAAT

**>CsLBD26**

ACTTTTTATCAAATTATTGAATAATATTTGATAGAAATCTCATGTACAACAAAACACAATTAAACATTATATTTACCTACATTTGTAAAACTTTGGACAAACGAAGACTAAAACTCACTCCATTTCATTTTAACATAAAGTGTAAATATTTTTCTCACTTGGTCATTTTTAACAATTTTTTTATTATTTTAAATGACAAAACTACATAAAATATTTGAAAATATAACAAAGTATGATCATAATCTATTTACGATATATTACAATAGACTACTATAATATATGTGTCTATGATATAGTAGTCCCTCGTGACTATAATATTTTGTAATTTAACTTGTGTATTTGAAACAAAGTATAATTTTATGGGTCGTAGGTTCTATCTCAAAACAAACAATTGACAATGGAGGGTAGACCACAATGTAAGATAGTGCATTTTGGGCTCACCAATCTTGAACCAAATACATGTGTTGGGCTTAATGGACTCTGATAACAATATTTGATAGTACTATTTTATCTCGAAACCAATTCTCAAAGTACTTGTTTGGGCTTAATGGACTTTGATGTTCTATAGTTCCATTTCAAAACCAATTGAATTATAAGTTAATTGTAATGGATGACCATTTGATAAATAATAATTAAAGATATAACAACATTTTAAAAAAATTGTAAATATAGCAAAACTATCACTAATAGACTCGTATGATCTATCGGTGATAGACCAATTTTTAAAACATGTGATAGACTCTATCATTGATAGAATTTGACAAATTTTGCTATATTTGCAAAAATTTTAAAATGTTGCTATATACTTAATTATTTTGAATTTAATTGCTAAATTTGCAACTATTTCTTGATTATAAGAAGAATATCTCTTTCTCAACTTGAGATTTTTAACATAGCAACGTATTTCGTATAATTTAACTAAAATTATATAATATTACAAAATAATAATTGAAAAATTATCAAAAAAATATGTACCTCGTATACTTAACTAATTGGATATAAGAAGAGTAGTTCTTTTTCGACGTGAGATTCTTAACATCGTCACAACATATTTCCAATAATTTAACTAAAATCATATAATATTACAAAATAATAATTAAAATGATAAAAAAACATATATTTCATATACTAACCAATGATCTTTTATATTCAATTTAATAATTAATTTATAAATATAACAACTAATTTATTTTAAATCATTATTCAAACTCCAATTCAATTTTTGAATGAAAGGGCAAAGACGTATCAAAAACATAAAATATATATAGTTTTACTTTTCTTGAATCTTCATTCTATCATAGTTTACATAAAATTTATAAAAAGCTTTTAACAATTCAAAATCTTATCTTGTTTTATAAGTTAACCTAAGATTCAAATCCATAGATTTCTGAAATCGTGTATGTTCTATATGTTTGATCCTATTAATGGGAATTCTCTCCCTTAATAAAAAAAAAAATCAATTTTACTGCATGTTTTTGTAGGGATTTAAAACAATTATAATTATTATTTCTTTTATAATTTTCGAAATCACTGTTTTTAATATTTCAAAATGAATACTTAAATACGTGTTTCTTGGTTATTTTACATATATATTAAAATAATTTTAATCATTTCAAAATCACTTTCAAACACCATTTCAAAATCACTCTCCAACACCGTTAAAGAAAAAAAAAATATTGGTTTAACAATAAACTTAGACAAAAATTCAACAATATAATCGAATCAAAACCGCACAAAAGTAGTGAAAAGGTATTTAGAAATTTGAAAACTTGCCACATAGTAGATCTGAATCAGCCATCCTTTTGAATCGGAAAAACTTTAAATACTAACTTTAGAATTTCTTCGTTAGATCTAAACCTAAATTAATCTGATTTTCTTTTCACATTTGCCATTTTTCTCCACCACACACTCACTCACTCATGATCAGCTCAAAACTTTCCCCTTACGAAATAACAGCCTTAATTTTCATAAGTTGGCTTGTTACAGGTTGGCTTCTTACT

**>CsLBD27**

TTTAATTCAATATCAGAAGATCCAAATCGTGAGTGTAATTAACAATTCAAATCAGCCTTCCACTTTCTTCTTGCATATTCTGAAAGGCCATCAAAGGTGTGTTGTTTCACATTTCCTTCCATTTCCCCATATCCAATGTTTGTTTTGATGGTCGTTGCTTACTTTTTCTTAGACACATTACTCATCCTACCTTTCATTTCTGTCAAGGTCAACCACTTAGGTAAACTCGTCTTTTTCTTGGTAATGTTTTATAACACAATTAAATAAGGGTAGTTGCTTCATAATAGATTATTTGCAGTGTTGATAGACTCTTACTAGTGATATAATCTATAACTATTAGACTTCTACGAACAATATAATCTATGATCGATAGACTCACTCATAGATAGTTTCTATCCCTTGTCTAGAGTTGAGCCCTGGGATTTGACGGTAGACTTAAAAAGCAACCTACAAACACTTTACGTCCCATCATTCTGAATAATGCTTGCATCCTTTGTATTATCACGACTACTAGCACAAAGTTAACTGATGCTTACTCTCCAAATATGGTCATTGTTGGATCTAAATTTTTCTATATATGTAACATCTTCTTGCCTTGGATGGACTATGTTTAGTAATACTTTGAATACTATTTGCAGACTGTCCTATTAAAATGGAAAAAAAACGAGTGTGATTTGTCAAGTAAACTCTTTAGCTTTTGGTTCATCCAAAAGGGTACAAATTCCGAAGTCATATTTATTGGACAAAAATGAAAAAGTCATTGCTCATCACACCTGTGGAAGTTTCTGTATGGACCTATATGCAAGTTTGCTGACTTTTTCTCAAATGGGCTCCAATAGAAAAGGCCCAATTAAAGCCCATAGAAAAGCCCAATTTCACTGTCTCTTTCTCTCTCTTAATTATTTAGGGTCCTACTAAAATGTTGAGTAGTACAAATGAAATCCAAACAAAGGGACCCTACTTTAATGATCGAATGACAAATTAGTGGAAGAGAAGAAAGCACCATAAAGAAGTGTTACAGTAAAACAAACAAACATCAAAATATCAAAACTTAATCAAACAGAAAGATTATTATTATTTATTATTATTGATGTTGAATAATTATACTGGGCCGGCCCAATTCACAAAAACTGACCAATCAAGATGATTCATATAATTTCTTTGTGTTTTGGTTATTGCCTAAACCTGCTATGCACTATCTTTACTTCACTCTCTCTTTCTTTTGGTTTTTTCTCAATTCTTCCAACAATCCATTTCTCTCTATCTTGTTTGAAGAAAACGACGACCATTTTTTTGACAAGTGGGTTCTGGGAATTCCATTATCATCCTTGTCTCTTTCCCCACCCCAATATTTTCTTTGATGTGACTCTTTTCATCCTTTCATCTTTTCATCATACTTCACTTTCTTTTCCCTACAAATATTTTGTACCAATCTCTAACACATAGAATGAAAGTCGTTTGGAAATTATCTTGTTAAAACCCACCTCTCACTACTACCACTACTAAGCACGAAAACACTCTATCAAAGTAATTTCTACAACTCACTTCCTTCTACTCTCAATTACGAGAATTCAAAAAATGAACTTAATTGAAGTTAGCACACTCAAGCTTAAACCAATTACATTGACTTGACTTATTTTATGGTGTTTAGGAGCTCCTTATTTCTTTAAATTTATACTTCTAATATTTTTTAAAAATGAATTTCCTTTGCCCCGTGAAACTAATTTGTTCTTCTCCCTTTTATAAAAATGATTAAAGGGAGATGAAGGAGAAATTTTGAGCTTCATAAAAGATAGAAAAAAGGAGAGGTTTTTATGATAGATGTACAAAAGAGAACATAGAGTTTTTCTTTTCTTTTTTTGCAGATTCCCAAACTATCAATTTCACACCACAAAGCTGAAAACCAACTTTCTACTAAAAGAAATAAAATAAAATAAAAATTGGTTTTTATTTTTACTTTCTTCATACAGATCATCAATATCTTACTTTATTTATCAAAT

**>CsLBD28**

TATTAAAAAGATATATATGGTAATTTAGTGTGTTTTTATTTGTTGTTTATGGGAAGAAAAGGCCAAAAGGGTGTTAAGTCATTTTCTTTGCATACGCAAATGAGTGACAAATGGTTGAGTCTTTTGGTGAATGATCTCTCTTGTGGACACACACCAAGAATCTCAACTACATTATCAAAATCTATTATTATCAAAATATTCAAAAGAACCATTATATTTGTTGTGGGATTTTGATTTTGACTAGAAACTACTACACACCAACCAAGTGAAAAACATTTCTTTCTTTTACAATCTCACTTAATTACTTTATTTTGTATTTATTTTATTTCTCAAGTTCCAAATTCTGACTTTTCTCTTTTAGAACATATGTTTCACTATGCTGCTTTCCAGTTTTTCTTGGACTTGAGGACCTACTCATAGATTTTAAGCCTTCCATAAATTAATCAACCACCAACATATGTATAATATAACGAAGTTTTTTAATCATTAAAATGGTCGAATAGGCTAAAAATTAAATTGGACACAAATTTGAAAATTAAGTATGTATTTAGATCTATGTCTTATGTATGTTGTTTAAGGGTTTATTAAATTCAAAATAGAAAGTTTGGAAAGTTTTTGTCTTCTAGTTTTTTTTCCCTATATATAGTTGAGTTACACTACAAAAATGCTCATTTAATTTCAAAATGTTTACTAATCATAGAAAACCATATATTTGCATTGTGTGCTTAGTTTAATTTATTAATTAATGATGTTTGGAACTATTAATTGAGAGATTTAATTTCATTCAATTCAATTCAATTCATTAGTATTATTAATTTCGCAGTCAGTTTTAACTCCAAAGTGTTTTTTTTTTTCCTGGAGTTTTAGAAAAAGTTGAAAGTTTGGTGAAACTGTCTATTAATTGATATAACTTCATTGCATCATATTCATTCCCATTTACTTTTCTTTTTAAAGAAAAAAGATCCCATTTACTTAACAAGAATTTTGGTCTCCCCACTAAGTATTCAATTAACAACGTTTTTGTTTGGTTTATATATATATATTTCATGAACTGAGTGTTAGATTTGGAAACAAAACAAAAAAAAAGTTACTAACTAATTATAGTCAGCATTCAATTCAATGAACATATTAAAAAGTTCGAATAGTATATATATATTATTAGTGTCGATGATGAAATCCAAATGGCTAGTGAAAAAGATTTAGTCCAACATTTACTTGACAATAGTAAATTGATTCTACATGCATGGGTGTGATGCTATTCACTTGAACTCTAATACTGAAACCAAATGGTAGAAAAAAATGTAAAAGGTTACAGTTTTAAGATTAAGAATAAGCTTATTTATTTTAGAGGTATAATAATATATTTGAGAGGTTTTGATCTTGGTTGAGGTCGAACACGTTGGCTTGGACTTCTGGACCCATCTAATCATACTATATTAATTTTTATTTTTCTGACGATATCATTTTGCCTATTATTATTATTATCGATTATGCTATTTGATCCAAGTTTACACCATCAAACAAATTGGGGGATGTATTTGGTTTAGAGAGATTAGAAAGCAAAATGTGTTCTCACATGGATGAAAAGTCAAAATGCATTTAAGAAGCCACACGTGAGGCTGCAGTCATAAGATATTATATACACACACACATATATATATATTAGCCTCTATTTTCCACTGCCTTTCCCCATTAATATTCATAAATACGATTAATTAATATTTATAAAACAAAATTCCCATATTCAATCTTTCTCAAGTTCCCATATTATAAATAAGAATTTCACCAGTCCCATTCATATTTTTTCACTCTTCTTTCTTAACATACAATACAACCCTAGTATTATTATTTATATATATATATATATCTTGCCTTTTAAGTACACTCCCTTTCTTTCCCAAGTTTAACTTCTTCGCATTCTTCTTCTTCATCTAAAAATCCCTTCTCTCTTCTACTTCTTCTTCTTTCGTTCAATTGGGGACAGAAAAGTGAATGCTTGA

**>CsLBD29**

AATTGTTAGTTATGTGTTTTTAAAAATGGGACTCCATTCATTGTTACTAATGTGTTGTAATTCCAATGCAAAATTCCTAACAGCAATTTTATTCCTTTACATTTGACTATTACGTGGGATTCACCCTACTTTTTTTTTTACTAACCTTTCATCCTTTTCATTTTTTTTATACTTTAGTTAATTAACAATATATAATTATAGAGTTTGTAAACACAATCAAATAAAGAATTTCAAAGGATGGTTGATATAATATGAATGCAAACTCATTACTATAATGACATGTTTTATTGTTGTTGTTGTTGTTTTTTAGAAAAGTTAACGAAATTTAATTTGGTATTAGCATTAATATATAACTTTAATTTTTTTAAAAAAAGTAAAGTAGTGAAATTTGTTATTAAAAGAAAATAATAAATAATGATAATGAAGAAAGAAAGAAAGAGAGAAAGAGAGAAAGGAAGACGTTACCTTATTATACAACAAGTTTGAACGGCGCGTGGGGCCCCTACTCTCGATCTGAGGTCATCAAATTACCCGGCCGTTACATTCCTGCTCACCGACTCAAACGTGGCATCCCCAAATTGGTGCACACTGTTCCCTGGACCCCACATTTGAACGCCGAACTTCTTATTTTTGACCTTTTTCTTCATTCCCCTCTCTCATCACACCCACACATTTACATCATTTTCTTCTCTCTCAATTTATCCCATCACATTTTCAATACTTTAATTCAATTTTCTAAAATTACTCAATTTTGAGCTTCATTAAACAATTCTACTTATTCATCAATAACATCTTTTAATTTTGATTAATTAACATGTCATATTTGTTACGTTCCTAGCTTGGTTATGCTTTCAATAGTTTAAAGTTAAAGATTATCATACTTATTAAAGAATAAAATAACTTTAATCAGTGTATAGTGCAATTTCATTTGATCATTTTTTGAAAGAGAAAAATATTCGAACCTCCTATTTTATCTTTAACGAAAACAATGGTATATTACGGTCTGTTTCTTCAGGAATTTTTGTAGAAAAAATAGCACCATTTTTCATACAATGTGTTGTTTTTTTGGAAAGTTAAGAGCAGAGCCAATTCTGTCTCACTCTCGCACTCATTCCTTCAACTATTGAGTTTTTGAGTGGCTTGGGTTTCAAAAGGGCTTCATCATTATAAATCAAAGAAGAAGGTGATTTTGGATATGAGTCTCATATAGCTTTTCTTACCCACTTTCCTCTTTTGGCTTTTTATAGTAAATGAAAAGCCATAAATGAAAAATATATATAAATAAAAGAAGCTGAGTCATTATTCAATTTGAACCCATTAGCAAAAAAAAAAAAGAAAAGAAAAGAAGAAAGAAAAGGTTGACGTCTTTTGCTCTCCACGCAGCTTAAAACTCGCAGTGTGGCAAAATTTAGACAGTCTGTTGGACACGTCTCCAAACCTACCAATTTCTTTATATATAATATTTACTTTGTTAATAAGAAAACAAAAATACCCCCTCTCTAAATAATGTGTTACAATTATAATTAGAATTATTAGAAGAGAGTGTTGTATAGGTAGAAGATTAAAAAAAACAACAACAAAAACTCACGAGGGATTTCAAGTGAGAGAATTTAAAAAGAAAGAAAGAAAGAAAGAGAGAGAGCACTAAAAATATAAAAAGAAAAAGAGAGTAATATTTAGCAATAGGGCTTATCGCATGTTTGCTTCACGCAAGCAGCTAAATGTAGGTGTTAAGGCTTTGCTCTCTCGCGTGGCTTTAAAGTCTTTCTTTCTTTCTTTCTTTCTTTCTTTCTTTCTTTCTTATTTTTTTATTTTCCTCTTTTCAAATAAAGTCATTTCTTCTCTTCTTTCTCCTCATTAAATTAAAATCCTCCCCCCAAAAAAATACATTTCTTTCACTTCCTCAATTTTCCACCAAACCCTAAAACCCCCTCTTCTCCCTTCAAAATCCATATCTACTTTTATAAAAATCAAAATTCTTTTGTTATCTATCTCCTAGC

**>CsLBD30**

GATTTTCTTTTACACTATAGCTTTATTTATTAATGGGTTATATGCATGACTTTTGGGGTTTGCCTTCCATTTGACTCCAAATCAAAATAAAATCTCTTTTATGACATTCATATCATGAATCCCTTCAATATTTCAATGAATCATAAATATTTTAATCAATTGAATATTTATTCTTCTAATGATTTTTTGTTAGTCTTAAAGGAACTATACAATAAGAAATGAACAATAATGTCGGTGTAAGAGCTAGGGAGGTTCCATTTTCAACGTCACATTTGTTAAACTAAAATAATAACAATAATACTAAAAGAGAAATACATCTTCATCAACCTTAGCATTTTAGTATTTATTTTGATGGACCAAAATTGTAACCTCCAAACTGGTGAAATTAGATGTCATCAACCTATGTTGTCTTCTTTGGCCATTAATGACATTTCTTTTCCCTTTTAAGTCAAATATTTTAGTCCACAAATCCATTTCTTGGAATATAAAAAAAGCACTTCAGCCTTTTCTTCTATTCTTCTTCTTCTTCTTTTATCAGTACAACCTCTAGTAGGTAGCCCCAATTAATTAATAAGAAAACACAACAAAGACAATATGATAATAATGGGTGGAAGCTAGCTAGCTCATGTCCATTTTATTTATTAAAATGTATATAAGCCCTAAATGTTCATTTCAAATTTGATTGAGTTTGATGAAACTTATCAATCAACTAAAAATATATATGTCTTAGACATATTCAAATTGGTTAAAGACATTTTTGATTAAATTTTCATTCTTATGCAGTATTCTCACCTCTTAAAAAACAACATTTTCTTCTTATCATATTTATCATAGGCATGAATAAGTAACCATTATTGTTGCCAAACACAATAATATTGCATCTGTGTAAGTAAAGTAGGTTAAATATTACTAATTCTCAACTAAAAAATGTGCAGTGAAGAAGAAAATGAAAGTAAGTAAAAAAGGACAAAGTACAACAACTATGATTAAACTGTGGTAGTAGTTGCAATGACACCCTCAAACTTTTAGTTGTAAGAACGAGACTCTTAAAATTTTTCTAATATTGAGTTTAAATAACCCTTCGTAGAAATACAATCTTAAACAATAAAAATTGAACCGTTTAATGTATACTTTTTGTATCCAATTTCAACACTCAATTCTTTTATTTCCAAAGTTTGACGATATAATTTTAACTATTTGTACTTTAGGGAAGATTTCTACGTTTTAGGTTAACATACAAATTAAAAATGAAACCTACAAGGACTTGATCATCAAACCTACCAATGACCTCAAAGCTGAAACTAATATACTTCAACACGACTTCCCTTTCATCATGTGTTTAGAAATTAAATTAGCTAGTACACAAAATGGAACAAAATGACTATAATCAAATATAAATGGAAGAGAAAATAACATAACATTTGGTATAAACTTGTTGGGTATATGATACTGACTTTGTTAGGGATGTAAGTAACTAGGTAAGTATGAATGTGAAGGAAGTGTATAAAGAGTTAAGATATATTAAGATGAATCATCGTTTTAAGAGACAGAAAGGGATAGATAATTGAATGATTTTGAGAGTAGATCAGCAGATACTATGATGAAGGCTAGGTTTAAAATTTCTGTGTTGCAATGGAACATAAAAAATGGAAAAGACAAGAGGAGAGGAGGAAAAGGGAAAAGAGAGAAGAGATGTGATGGGATATATCTAAAAAAAAAAAAGAGTAGATGTGAATGTAGACGTGGCAGTCATTGAAAGGGGTTAAGAAGTTTGCTTGTGTCACCACTCGGTGTTTATATGTTTGCTTCACGCAAGCAGCTGCCCCTCAACTTCCCCTCCTCTTTAAGACTCAAACCAAAATCACACACTAAAACACACAAAACTTTCACACAATTCATCCAAATTTTCATCAAACTTTCAAATGTAAAGGGAAAAACCCCATTTGCCCTTTCATATGACCCACACCCAAACATCAATTGCCCTCCATCAACATACCCTC

**>CsLBD31**

CTAAAAGAACAAATTTCCTTTATTAATTTTAGTATTCAATAGCTTTTATCATTATCTTCTTCCTCAAAGTTCATTATTGTAAGCATCAATTCTTCCGTTGAGTTCAACCCGTCTCTCTAACGAGTGTGCATTTTAAGATAAGTGTTGTGGTAACTTCATAAAGCAACCAATTTATACCATTTCTACAAAAGTCGGACTAAATTTAGTTTCAAAACAATGATGTTCTACAACACAAAGAATATCAACCGATAATACATAGGTGGGAGAGTAATTACAATATATATAAGCAAAATGGAATTATTTATCATTTGTTGATATAGTACAAAATAATAATAACAAAAACAATAAGTTTCAAGTCATTTTTAAAGACGACTAGAGCTCCATTTGGCCAATAAGAAGATAAGATTATTAAATACATAAAATTGAAAGAGTGGAGAGAAAATGGACCATATAGCTTTTATATCATAAAATATTGAAAATAATGTAGAAAAAAAAATGAAAGAAAATCGAATCCAAATGATGTAACTTTGCTTAGCTTAAAAAACAAATAAAAGAAAAACAAAGACAATTAACAAAAAATAGTATTAACATCAATTAGATCACATAGCATACTGATACAGTATAGGATCTAATTATAAGAGAAAAGTCCAAAATTAGCACGTGTGATTCTTTCGTCATTTTGGATTCCAATCTTGTCATTCGTATCTTTTCCTTTTGTATAAATATAATATAATTCATTATTGTTTTGTCCAAGAAATGTATGTAAAAGTTCCGCCCAATTTCATATTTTATATGTCTATATTTAACATTTAATTAACCACCTAATTAGCTTCAACTTCAACCAAACTTTACTTCCTTCCACACTCCCTACCCTATTACATTATAAATTACAAAAACAATAGTTAACATATTAGACAATTTACTTTGAAATCAAATAAGTAGACTTTTAAAAGTTACACACACACATATAACTTTCTAATACATCATTATTTAATTATTATCTTTCGTTCATTCTTTTTTTTTTCTCTAAATTCTTGTTTTCTAAGAACCCTGTCAATAAGGTAAGTCATAAGTAAAACAATTTTAGATATATATCCATTATTTGAAGAGCTAAGATTGTTCCATAAGTAAGTATACTTCTAAAAGTAAAATAATAACAAAATTTAAAAATATATATTCAAACGACTAAGTACAAATTGTTTGTCCTAATTAAATGATCAAATATTTTTATGAAAAATAATGATAATAAGCGCTCTTAAATAAATCTCCAAACAGTAACTCACCCATCTAAATATCTATTGAAGAGACAACTGAAGTGATCAACACACACCCTTGGATTTTTTTTTTTTTTAAAAAGACACCAATCAAAATTAAAACATTCATATGAAAAATATAAATAATATTACTTATCAAATACCATGGGATTGGATCCAGTTGCTTAATGACATCAAATCAACAAGTTTTAAATTGATATTTAAAAGAAAAAAGAGAAGAAGCTAGAGGATTAATTTAATTATGATATATAATTCATAGCATGTGGTCTAGTATTAAAATGTCCCAAATTACAAATAATAATAATAACAAAAAATGCAATAAGTGGGTATTGTTGAATAACTTTACACAAATATAATTAAAGAAAAGAGAGCAAGAAAAAGATGTGAAAGAAAAATGGAGAGGAGACAAAATATCATAATATAGTTGTGGGGGCAAAAGAAAGATCTTCTCTCAACAAAATAATAATAATAAACAAAAAAAAAAAATTATTTTGGTGATATGGGGAAAATCTAAAGGGTTCTTCTTTTGTAGGTCCCTCTAAAGATTTGCCATCACTTCTCTATATAACATCTTCCATCTTCTTCCTCATCGCTCCCTACTTTTCCTTTCTCTTTTCTTCTACTTCTCAAGAAAACCTCTGACTCGTATTTCCAAAACCCAATTTTAGCTCAAAATTGGATAGTTATTAAAACTATCTCAATTTGCATTCTCTTAATTAAAGAGAA

**>CsLBD32**

GAGAATTTTAACACCGACCTTGCATATATTAAGATAATACATGTCAATTACTATAGATGAATGAAATAACAACGATCATATTTTAATTTTACAAATATATCGACAAACATTTTTCAAAACAAAGATAATTATCAATTTTATTTATATTAATAATTTGGTTGATTTTAGTTTCAAATTATATTATTAATGTATTTCTCTATCCATTCAAATTAGTGTGATAGTCTTGTTTTTTGTTTTAAATATTGTATGAGTATTTTCAGATTATTAATCATGTCGAATCTTTCGACTTACCGATATATATGATAATTTAGGTTTTCAGCCTAACATTAATTTGACACGGTAATAATAGAATAGAAATACGTAAAATACGTTTAAAAGGGCTTTCGTTAGTACCAAGGCATCTAAAAGTAGAAACAAAATGTGGAAAAAATGTATGGGAGGAGAAGAGTCGTTAGATATGCACAATAACATTTCACTATTTATTTCTTGATTTTAACTTAGTATAATCACATGCTACCCATTTTGAGACTCAATTCCATGGCCTTTTTTGCATCTCTTTTTGGCTGTCTATCTATATATTCAATCGTCAAATGCATGAACTTATCATTTTTATTCATTTATTCAACCTGTAATTTTGTATATGATTTTAAAAATAAATAAAGTAAATAGTGAATATGTTAGGGAAAAAAAAACAAGCAGTGTGCATTAAAAATTGAAAGGGAAAGTTAATGTATGAAACTAATTTGGTTCAACAATTGTATTTCTTTGTGGGGAGGAGGTTTTGCAATAGTGTAATTTAGATGTTTAGAATTTTATATTAATTTGGATGTTAGCTTCATGGATGAACACAATTAGTTGCAAAGTTCAATATGGTTAAGTAAGTTTGAGGCCAAAAGTTTCTAACCTCATTACCTCATCTCATGTCGTATTAAAAAGAGAAAAATAAATGTAATGTAATAATAGCATACCATATAATATTTAAATGATTGTATTTTTCAAAATGGTTTCCATGATAACAATTAAATCTATTTATGACATATCATATGTCTATCATGATATAGATAGTATAGATAGTTGTTTACCACGATCAATCGTAAATAGATCGTGATATTTTATTATATTTTTGTAAATAGCTCTATTTTGATTTAATTTTGAATGTTTTAAAATAGCTTTATTCATCTATTTTATTATATTTTTGTAAATAGCTCTATTATGATGGATGAAAGTATATTAAGTTATATATCATCTAATACTACATAGTTTTGCATTTTCATAAATGTCATGTCATTATATTATGCAAACCGTGGAGAAAAACAAATGAACCTATCAGTTTTAATGGGAACCTCGATTTTAAAATTTAATACCTAGAATCATAAGTTTATGAGTTTAGTATCCATTTAGTCCATAAACTTAAAATAATTTTACATTGGTATACAAATATAATAAAATATCATAATTTATCTATGATAAGTTAAAGTAGATCACTATTATTTGTATTTATCTATATCTCAAATTAACGTAGAAAATTTGGCGACACTATTTTTATATATTTAAAAAATATAGATAAAAAAACTACAATTTTACAAATAAGAAAAGTAAATTAAAAAAAAAAAAAAAGTGCATTCACATCCACACCGTCACATGGTTCCCCATTGGTTAGTCAAAACCAGCCCTAATATCCATTGAATTTCAATCAAATCTTGAAATGTCCATACTACCCTTGCCCACTTTTGGTTCTCTACTTTCTTAAATGTGTACACAAAGAAACTTTAACTTTTCAAAAGAAAAGAATTAGTCTAATAATTTCAATTTGATTGAAATCATCATCACATGCCCTCGTTTCCCTATATATAAACTCACTAATGGGGTTCTCTCTTTTTCCATCTTTTGGATTCCCTCTTCAACAACTCTCCAAATCCAAACCCATTACTCTTAATCATTTCCCTTGCCTTCTCTTCTTCTTTTAGTATTCAAGAACACAATATTATTGTGTATTAAGGA

**>CsLBD33**

TACCCATTTTTTGTTTTTTCAAAGCAGTAGATTATAGATCTTATAGTTACTAGCACACTTGTAAGCTGAAGATCATATCTAATATGTTATGATAAGTGGAGAGCATTTTTTCAAAGCAATGGGTATGGTAAAAGGTGGTGGTATTAGGAGTAGAAACATAGCATTGTCTCAGTGGTGGGAGCAGCCACCCACCAATGCATGTAATGGCACTTTTTATTAACTGATAAAATGTGAAGAAAGGGTCACCTTCACAAAAGGTTTGGTAGTAATTAGTTTAGTGGGACTCTCTCTCTCTCTCTCTCTTTTCATTTTAGTTAAAAAAAGTGTTCGAATAAAATATATGAAACCAAACTACCAAGTTAATAGAATGTATATTTTCGTTTCATGTTGCAATTCATTTTGAAATGTTCTTTTTAAGTTCACTAACTTTTAACGACATAAAAGAAAGTATATAACTTTAATCAACTCCTAATAAGTTCTCTAATTTGTGTTGAAACTGTTTGTATCATTGATTAGATTATTTGAAATGAAAATTGGAAGTGTAGAATTAGTTTGTGTTGTTGAAAAATTAAAAGACTTTTGTGGTCTTAGTTTATGACTTGAAGTCCAACAAAATTGGAATATCTAGAAAGAGATAAGGATGGTCTCTAATTCACAAAAGTCATATAATTGGAAAATTAGGCATTTGATTACTGCATTAAATAAGAAGAAAAAAAGAAAAAGAATTCTTTCCCACAATTATTCCCAAGGAGAGAACATAATTTTCAACTATGACACCAAACCTTCATACCAACATCAACAATGATAATCATGAGGGTATTAAATCATAATTTCAAAAAGAGGAACCACAAATTCAAAATCACATGTAATTACAAATTTTGTTTAAAATGTAAACATCTAATATGTTGATTTGAAGCATATGTTTTATATTTTTTTTTTACTGTCAACATGTTTTATGGCTTAAGTTATGTTTGTTTTGGAAGAATTATAGCAAAATAAATGTGTATTTTAAACAAATTGGCTTATGTGCAAACTAGTTTCTTTCTTTCTACCAAATTGAGCATGTTCTCAATTAGCTTTTAGAAGCAAAATATAGAATTTGTTATCTCACGAGTCACATTACTCTATGGAAGGAATGTCAAAATAAAACACATGTATTTTCATCTCAATATCCATATTGGAGCTTCCCACCTTTATATTACTTTTAAGAAAAAATTATTTAATCAACTAAATAAATCANNNNNNNNNNNNNNNNNNNNNNNNNNNNNNNNNNNNNNNNNNNNNNNNNNNNNNNNNNNNNNNNNNNNNNNNNNNNNNNNNNNNNNNNNNNNNNNNNNNNNNNNNNNNNNNNNNNNNNNNNNNNNNNNNNNNNNNNNNNNNNNNNNNNNNNNNTATAAATATTTTAATTTATTTTATTATTTTAAAAAATGTTCCAATTCAAAAACGAGAAAGTGAGAAGTTCCTCTCGCCCATATTTTTTATATATATATTAAATTTTGAATTATTTGCATTTAAAACGGAGAAATTGACCTTTTATTAGACAAAAGAAATACCCTTTTCTAGACTAAGTGTTTTGTGTAAACTAAAATAAGCTTCACACACATAATTACATTTAGACCCATTTTTCATCTAAATTCTCATTTAGTCAAAGTTTAAGTCTCATTTCAATTTATTTCATAAACTTAAAAAAAATGTTACAATTTAATATTTAAAATTAGAAAAGTAATTATTTATTTGTAAGATATGAATTAATAAGATAATAGTAGAAAAGAAAAGAAAAGAAAAAGGTGCAAAATCAGAAACCTAACTAATTAGAATTAATGAGGCAGCCCCCATTCAAAATTAAAATCATAATTTTCAAACTTTAACTCATTTCATGTTCATACCCTTTTCTCTTTTCTCTATTTATATCTCTGTTTTTCCATTTCCAAACGAAACCAAACCAAACCCTCCATTTCTTCTCCTTCTTCTTCTCCGTTTCTTTACTCCGATCATCCCAAA

**>CsLBD34**

TTGTAATATTGTTGTAGGATTTTTTTTCTTCCAATTTTTACTATTATCTTTTTTAATTTAAATAGTTTACATATTAAACACGTACTACGATAATTTAGAATTACAACAACCAACTCATTATCCTATTATAATTCAACTACTATAATTTAGAATTACAACAACCAACTCATTGTCCTAAACATCTTTGGATGCACGATCCTTTCTAACCAATTAAACATGATCCTATTTAGTTATAACAATATATTACTTCAAAATATCAAAGTCAGATTGTCGGAGAAGAAAAAAATTAATTAGAAAAGTCAATAAATTCCACTAGTAATATATAGTGGATAAGGTGTATTGTTTATTAGGGAGTAATGTAAAGTGTCCAATTAAGAGTTGGGTATGTGGCCTTGTAAAGGCAATGGAAGAATCCCCCTTGAAGAGTTAATACACACAAAAAATAGTACAACATGGTCCACAACCTAAGATCTACAACATTTACAAATTTGGATATTTAAAGCATTTTTAAACAATAGGACTCTCTTTGGTCCCTTAGAGTTGTCACCCACAATAAAAAGTTGAACTCTTTATTAGTTGGAATCTTTTTTGAAACTCTTTTATCCCTTCTCCTCTCTTTCTTTTTTAACATTTTAGGTAAAGAGGGGAATTTGAGTTATGTTTTTCTAGTAGATCCCCATTTGGTATAGAACCAAATTATTTGAGAAGATCACTAGTGTTTTTTAATCCATCCTTCAAACCTTATTCCAAGGTACTCACATTGTGACCAACCTTCTCAATTAACTTTTCCTTTTTATGATTTTTTTTTCCCCTTTAAATGTTGTATATCTTACAACTAATTAAAGTAGGTTAAAGTTATAATTAAGTATAGTAAGGTAAAAATTTATTTAGACTTGTAGTAGTTCATTGGGCAGCACTTCAAAAACATATAACAAATTGAAAAAAAGAAAAGAAAAAAAAATGGACCTCCCCACTTTAAAATAAAATTTTATAAGCACTCTTTATGGGGAAAAAAGAGGGTATTAAAAATATATTAATTTAAGAGCTACATATCTATAAGCAATTTGGTATTGACCTAATTTGTTGGGAGGAAAAATAGTTGGCAATTTCATGCACTTACTGTAAAACCCCAATAAACAAATAGAAAAAATATTTATTTTATGAATCATATAACGTATTATGTAAATATAAGAAGATGGTCCAAAATAGTTTTAAAAAAAATATATATAATTTGGTAATTTTTGTAAAAATAGATTGGGTTGAATTACAAAATTTGAATATAGTTTTTTAAAAATGAGGAATCTGATAGATAGGAAAGAAAATTTAGAGTTGACTAATTTAGAGAGTTTGAATAAAAAGAATGAAAAGTGGAAGAAATTGATTTATAAAATTGAAGGTAAAGATAAAGAGGAAGGAAAAAGAAAAATAGAAAAGATATTTAGGCGGTAGAGAGAAAAAAAGAAAGAAAAACATTATTTAGGCATAGAAGAAATGGAGTCCCAAGGCCAACACGAGAGGCCACATAACATGGAGGACCTACCTTCTCTTCTCCCTCAATATAATTATATGAATATATGAATATATAATTATATATTTAAAATAAAATATTAAGAAAAGATATTTTATAAGAGAGAGGAAGAAACGCATTAACAAAAGAAGCAAAAGCAATATCCATAAAATAAAATAAGATAAAAAGAAAAAGAGGAAATCCAACGGTGGAGGAGCGGCCATCCGCGGAGACACGGAGCCGCTAGCACCCAACTCTTTGGAATCTCAACACAAAAGAAAATTTTAAAAAAGTGTTTTTCTTTATACCCACGCGCTCTAACCTCGCGTCACACACTCCCAACTAACCCAACCCTTAACCACAGTTAAAACTTCCCCCCTTACCCCCTTCTTTCTTTCTTATATATCCCTCTTCATTTCTTTCCTTACTCCCCCGATTCCTCCCACCTTCCTTCTTCTTCTTCCATGCCACACACCATTCCCATTTCTCAACCA

**>CsLBD35**

AGTTTTGTCCATGACTTCACAAGGACATTTTTGGATTTTAATGCATGTTATTTAAGCTATGCTTATGAATACCAAGTTTAACAGTTTAAGTTTTAGTTATAGACTATTCCTGAACCAATGAAAGTTCAATCGCCTCATACCTATTTTCTTCCTTGTGAAGACAGCTTGGAAAGCTAGGGGAAGCATTTTAAGATAGACAAACTCATTTACATGCATTCATTCACTTTCAAATTCCCGTGCTTGCCCAATGTAGAGAAGATCGCATGGCCATTAAATTTATGTTCTTTATCCCTTTATTTTATTACATATTGTTTAACTTTGTAAATGAAACGAGTTTACTGTGAACATCTTTGCGGTTATTGGATATAATGATAATGGTTGCTACTTTGCAGTTGTACGCATGGTGTGCCCGTACGGTTTCTACATTGACTGCACGCTCACATGTGGAAGATCGATTCGGAGTTGCCCAGCTCTCTGGGAGTAATGCTACTGTTATGTCAACACTATTGTCTTGCCTGCTTGCTGTTGAAGTGCTGATGGGGAAGAAGACTAATCTACAATCTTCACACAATCTATTCGGTCCAGCTGGTATTAAATGGGCAACATCGAGTATTAGAAGAGTAGATGCTTCAGTTGGTAAAAAGAAAAACGGGCCGCTGCACTCGAAGGCATATGCAATTGCAGACGTATTGAGGGTCTCGATTTACCTCATTGTTACCGCATTCCATAATGAGATGGTGAACAGTGCCAAGTCTGGTGTTCTTGAGAAAGATTGGATCACGGATGAAAAACCCCCTTTTGGTACTCGCGAGTTGCTTCTGCAGAAATTGCATATTTTCTTGGATTTTCAAGCTTAGATATAACCGGGTTTCGACCCGTTTAGTGAATTATACCTAGCCACCTGTTAGGCAGTTTTGTCCTTTCTAAAGAGTTATGAATATGAAGATGATGTTAAGACTGATGGAAATTCACAAACTAAGTTTGCAAGTTTGTAATACGCTTTACATCTCTTGTCTCCGCTTCTTCACTCCAAAATTGAGAAATTATCGCGGCCTCATCATTTTGACTCTTGGTAGAAACACAATCCTCGATATTGCTTTTATATTTTTGGGTTTTAATCAAACAGCTTATAAGCCTTTTCTTTAAGTCTCTACTTATTAATACGATCATTTACTGCGGTTTCATGGCCAAACCCTAATCTCCATTTCAAATGCTATATTATCCAATCCTTCCATATCACTTGGGGTTATTAGTCCAATTGAATTGGCTTCAACTGCCTGTAGATGTAAACGTGTATATGATTCTTAATAACTTGTCATTGATATGATCATTTGGATTACAAGGATCAAGATTTGAAAATTATGTACACATTGTTAATCGTAGTTGTCGTCAATTGGTTGACATAAAGGTTGAGTATCTTGGCACCAACAAATCGCTTCAATAATCTCTCACATATATCGTTTCCAATTGTGAGTTCTTTTGGTCTAGAGCTATGAAAAAATTTGTCTACTGTATTGTCAAGAAATTTCACAACCTAACCCAACCAAAAACTTATTGGAGGGCAAATCTCGTGTTAACAATACTTTGGAGTTGGGTCGTCCGAGTATGAGCTTGCCAACCCAAACTCAGTGACATTTTTTTCCTCTCTCAAATTCTTAACAAAAGAGTTACTTTTCTCTTTCCTATAAATTTGTTTTCACTTAATAAAAGTTTTTGTTCTCTAAATCTCCATTCCTCCAAATCTAAGTTTTTGTTTCTTATCTTTAAAAAGAAATTATTTCATAATTATCTGTATCTTTTATAAAAAAAATATTTCATAATTATCTACCATTTAAAACGTTTGATCTTCAAATTTTAAATAACATCAACTTAAATCTATTTATCTTAATAAATGAATGCTAACGCCAATAAAAATAATAAACAAAGAGATTATTATTATATAAACAGCATTTGATTCTGATATGATATTATTGTGATTATAGAAACTAATTAAAGACGGTA

**>CsLBD36**

GTTAGGGCCTCTCCCTACACCTCAGTTTGTGCATCAGAAGAGGCTTTAACTCCTAACGTCATATCTTCCGACGATGCAAAACGACGTTAGGAGATCCAAAATTTCTTGTAGTGAACTTATATAACTCGAAGTTTTAGTCAATTCAAAAAAAATATATATTTTTTTGAGAGAAAAACTATATACGGACAGGACAAAAGTTTAACCTAACTAGCTACTATATATTCTACTAAGATTTTTTTGACATTCATAAAAGTTGGGCGTGGGTAAAGAAAAGACAATTCTTTAGAAAATGTGGTAAAGTTATGACATTTGGGAAAACCAATTAATTATTAATGCACGAGTTACATAATATAACAAGATAAAGGAAAATGGTAGAAAAAAAAAAACACAATTATGGCCATCTTCTCTATTTGATGTAACTTGCCTCCTATATAACTATCAATGAGATGGACATCACCAAAAATTTCTAATATATTTCCCATTACAAAACAAATATTTTCCCACTATCCTTTACATTTTTATTTTATTTAATTAATCACTACGTGTTTTCTCCATTTAATCACACTGTTCATTAATCAAATTTTTGTGTGTGTATGTATAGTCCATGATTTGATATTTGAATGTCTATACTTTTATGGATTCATGCATTAGCAATATAAGATATGATGATGTATATAATATTTGAAAAAAAACTCCTCTTCTCTCTCCCACTAAGCATACTAGAAAAGGTAATCTTAATTAATTAATTCTATTAATACATTATAAATATATATACTTGAAGGATAAGGCAAGTTAATTAGATACGTAGCTTAAGAATCAAGCAATTAATTCCATAATACATAATACATTCATTAATCAACATATGCAAGTTACCCATATAATTAGCAATGAGTTTTTGTGTTGTCATTATACGGCCATGTGAGCTTGACTAATTATAACATCAAACATAAAGTGTGGTTGAAATTTTTGAATTTGTCAACTATTACTACAAATGATCACATGGAAATGACAACAAATTATGGCCCCAACTAATTAAGCACTTTAAATCTCTTTCTTTTTTTCTTATCTATATAGTTAGTTTACGTTCTTATCTACAACCCAATATGAAAGTCAACTATTTCAAACATATATATATATATGTGTAAATTCTTCTTCCATTCAAGTAGTTAAAAATATTATAATTAAATCCAAAAAAACACACCATCTATGTATTAAACCTGAGTAAATAAAGTAATCTCCAAAATTCATGATTTAGAGCTCTTCCGCAAGAACTTATATACTTTAAAAAAAGATAGATAACTAAAAACATAACTTGCGATGGACGAAACTACATGGTGGAAGGAAGATAATTGTTTTTGTAAGGAAGAAATGATCAGACAAGGAGGCTATTCATGGGTGTGAACCACTGTTTAGTTATACAAACTTTGTACTATACATATATATTAGTCATTATTGTTAGGTGTTATTTATGTAGCAACATTGGAAAAACAGAAAAATGATGGAGAATGAAGACAGAGAAGAATGAGATTTTTATTGGATAAACAAGAAAAGTAATAATTAGAGTTGAAAAGGAAAAGGTGAAATAATTAATTGTGATATAATTTGAATGAAATTAAAAGGGGGGGTTGATTTGGTTGGTAAGTAAATAATGAAAAATATTCCAATAATGTAGAGGCAAAATCTTGGTAGTATTCTTGGATATATGGTGGGTCCCTTCAAAAGATTTTTGATATTCACTTTCTACTCTATTTAATTCAATTCATGCCACCTGATCCTTCCTCTCTCTTATAACTCATTTTTTCTTGAATTTGCCTTTCAATTTCCCCCCCAATATTTAAACAAAATTCACTTCTTTCTTTGAGCTGTTTCAGTTTAATTCTTCATCTGGGTTTTATTCACACAAAATAATATATTATTACAGTTGATAATTAGCAAGCTAAGATCAATTTCTGAAGATTAATTATATAGTAACAATATTTTAGTACTGATCATCAAAATAAA

**>CsLBD37**

ATGGCAGCCTCTTCTTCTTCTTCTACTCCTCCATGTGGAGCTTGCAAATTTCTTAGAAGAAAGTGTGTCAATGGTTGCATCTTTGCTCCTTATTTCCGCCACGAGCTTGGCGCCTCTCACTTCTCCGCCATCCACAAAGTTTTTGGTGCCAGCAACGCTTCCAAGCTTCTCGCTCACCTCCCTGTCTCCCACCGTTGTGAAGCGGCCGTGACCATCTCATATGAAGCTCAAGCGAGACTTCAAGATCCGATTTATGGTTGCGTTTCTCATATTATCGCTCTCCAACAACAGGTTTTTTAATAATAAAATTTACCATATAAACGAACTCATATCATCCAACTTCAAGTTTTAGATTCCATGATAATAAACTTTATCATAATTTTTAAAGAAAAGCAAAACGATCATACTTATTTGATCTCCTCACCTTAAACTTTTGAGTTTATGTTAATTTATTCGAAACCCTAATTTTCGAAATCGAGTTTGATGAGTATTTGAATGGGTGTGGAAATTGTAGGTTATGAATCTAAGGGCTCAAGTGGCTTATTTAAGAGAACAAGCAGCACAAATTCTTCTCAACAATTATACCACAAGAAACCCTACTGAATTACTTTATGAAAAATCTCCATTTTGTTATTCCGATGACCCATTCTTAGATCTTCAAAGCTGGCTTCTGGCTGAGAGCTGCAGCGCCACCGCACCGGAGCTTGAACCTGCGGCGGTGCCGTACTTTGAGATGAGTGTCGTCGATTCAAAACTCGCCGGAGATTTCTGTTGCTAAGGATTATTGTGGATCGTGCGATCTCTTCCCCCCTCGTTTTCATATCAACGGCAAATAATAATTCGAAGTATGGGTGTCGTTTTTAAGATAATTTGAATTTGAATTTGAATTTTATTTTTATTTTCTGAATTTTTCTTTTTCTTTCTCTTTTTTGTTCTGTTTGTGTTTATGTGTGATAATCATGTGTAAAATTAAATTAAATAATATTTTAAAAAACAAACCCTAAATGTTGATATGTATATATCAATTGGGAACCCTAAGATATTGCCCTTTTTTAATGGACTTATATTAGCCATTAAATAAAGATCTACAAATTTTCTTGAAGAGGAAAAATTATTTTCTGTTATAATTTAGTATTCTGATGTTTATATTTAGTGATATACTTTTTTTGCATTTAATAGATCATAAGGACAAGTTCTGATTTATTTATCGTTTCTATGTTTTCAAAAAGAAAAAAAACGTTTGGAAGAAAAACAATTCAAACCTGTTGAGTATATTCAATTTTAAAAACGATTTCAAAAACCAATAACCCCTTCAAATATTCATTCGTTTAAAATTTTGTATTTATTTTATTTTTAAATTTTATTCGAGTCTAATAACCTAAACATTTAAATTTTAAATTGGAACCAAACGTGTGTTTAAGAAATTTTTAAATGATGAATCAAATGAAAGTTGAGTATTTTATCTAGTTTATATAATCAAACTTAGTACAAAATTTCTTTTTTTTTTTGAAATTGACGCCTTCAATTTTCATATTCTTCCTTGTACCAAATAAAATTCTTGCTTCTTCACGTGTGTTTAATTTTTGTTTGACAGGGAACTGATTGAATTATGTTTCTTAAAATTGGTTTTTAGATTTTAGATTTTGTGATAACTTACATACCATTTTTGAAGAACATTTTTCTATTTTCAATATATGAAAATTGTGAATTTAAAATTTGAAAATGCAAAATTATATTAAATAGAGATAACAACACTTTAACTTTTAAGTAAAATATGTATTGTGTAATGCATTATAAATTATATAATATTACATAATAATAACAATTATACACTTCTTTTACAGAAAACATATTCATAAATAAACTAGAATAACTTTTTTTAATCTGTTGCCCTTGTTTTAATGTTTTTCTGTCAATTAATGGTTCCTCGTTTTTATTTTAATCTTTAAACTTATCTTTTCATTTTTGTAAGAAACGATAGGATGATGCATTAGTTATTGC

**>CsLBD38**

AAGAAAACGGAGAAGATTGAGGTAAGGCCGATCCCTACGGAGGGGGAAGGAAGCTTTTCGGTTTTTCTTTGTTTTTCAAATTGTAAAAAATATCCTTCGGAACAATTCTTATTCCGTTCCGTCCAATATTAATATTTCATAAATATTATTAAACTATTAATAAAAACAATATGAGTATTATTATTTTTATTAATCAAATTCAAAATCAATTTGTTTTAGTGTAATAAAACTAAGGGATTTGTTTGATTACAATAAAAATAAGAGATTTGAAGGAAACACTTGTGATAATGTAATTGGATTTCTACCAAATGAGAAAAAAACTCATTTCTTTTTTAACATTCCAAATTCTCAATTTCTGAAACTGGGAGTTCATCTAACATGAAGACTATTGCCCTCTTCTACATCAACCAATAGCTATATGAATTTCTTGTCATTCAACTCATGTGTATGAAACTTGGACAACACATCGTTTGCCATTTCATTTAGTCATTACTAGTATACGTCTAGTGATGAGCAATTAAGGGAAGAGCTTCGGTCGGTTGCTAGTATCACGAATGGTGGAAGTCAGGGTTGAAAACCCTTGAATTTATCTAAAAAAGTGTATTTCAAATTTTTTTGATCATTTGCATGCGACACAACAACACGAACACTACAACTTTTTTAAAAAACTAGGACAAACACGTTGTTCCATTTGAAAACTTTGATTTGAGGGTGTATATTGAGGTATGACCATTTGGACATGGAGGCTTAGGAGGTTTGTTTTCCAGGAAAAGAAAAGGAAGATAATAATAAGCCTAAAACGTGAGATGAATGGTCTTTGTGGACTTTTAAAATGCTAGCTTTAGTATATTTTTTCTTCCCCAACGTGCAGGTTCCATGCCCTAAAATTGTCCAATAGGTGTAAGTGTACTAGTTGTTTGGACGTGTCCAAAATTAAATAATAAAAATAAAATAATTGTCACACTACGGACACATGTCCAATACAACACTTGTCATCTTAAAAGCGTTAGTGTTTCTTATGTGTTGAGAGGTGAAATATAAAGACTTTCAAGATTTTTAAGGCCAATTAAAATAGGGTTTCGAAGATCCAAAAGATCAAAATCAACGTACAATCAAAATTCAATAGTTTAAAATTTAAAAATATTAGGTATGAAAATCAAGAATTGAAAGATAAAAGAAAACAATTTGCTAGAAAAGTAAATGAATATTCTTGCTATATTTTCAAGGCAAATGCAATATTAAAAGATGCTTTATACTCACAAGCTCTGTCACATCCTCAATGGTATTTTTCCCCTAAAACCAATGGTCAAGCAAACAAGTCAAGTCCATTGGGCTGGAGTGGGCAATGTCTCAAGTATTTGGGCTTATGCTTAGCTTGACGAAAGAAAACCCACTTTTGCAGCTCTCTCTCTCTCTCTTGATTATATTAACAATTTATCATATATTGTGAAACTTGAATGTTTGTTTAAAAAATATAGTACACTTAGTCAAACTTTCATATACAAAGCCATAATTTTTTTTAATAATTTGGAAATTAATTCCCAAGTGAATTTAGGAATAAAATGGTAAAGAATTTTAGTAAAATTGAAATATTGTTAATGGAAAATATGAGAAAAAGGTAATTGGAAGAAGGGAGAAATAAAATAAAAAAAAAAAAATAGAGAGAGAAAAAGAGTAATAAAGAAAAGTTGGAAAGAATCAGTTAGAAGAAATCCCCCATTTTGCAGAATTATCGTTGGTGATTGTAACTAAAAATAGTTAGAATGGTTTAGCATTTCACATCCATCCATTCCCTCAAGAACTAAAAATTAAAGAGAAAAAACTGGAAAAAACAAAGAACACATAGAAACCAAATCCCTCAAAATTTAAAATTATTCCCCCAAAAAATAAGCAAAAGCAAAGAACAAAATCCGCTTTCTCCCATCCCCGCGGGAGCCGCACCCGCTCTCTCAATACCTTCCATCCTCAACAACAACAAACACTACTCCTTAACCCCTCAAA

**>CsLBD38**

TAAAGTTTTCCCCACTCCAGGAGGACCGCTAAGCAGTATTCCACCTGATTAATAAACATTTAACCCTTGAAGCATCCTAATGAAACAGCTCCAGTTACTTTGTGTTTCCATTTAAAGCAGGAAATTACTCATACATGGCTTAATCTACATTAGGACCCAAAAAAATGCTCATTTGTAGTACTAACAAACCATTAATAAGAAAAAATTCTTACGATAAAAGTACATTCTGACCTGGTATCTTTACACCCCTTCTCCTGTACATCTCTCCTTGTGTGAAAAACTTCACAATTTCCTCCAGCTCGAGCCTTATTTTCCCAAGCCCAGCGACATCCTCAAACTTCACATTCACCCCTTTCTCCAGATACTGAGGCAGCCTCTTGCCATGAGCCCGACGAACTCGAGCACCAGACTTCATAAATTGCGTAGCCATCTTCAAATAAGGATTTTGTTCACCTTTTCCTTGCTCAATATCATCATCCTCACCTTCTGTAACTTCCAGATCCGTCTCTAACTCCAGCATTTTCTTCCTCTCCTCTGCTTCAGCCTCCTCAATCTTTAATCGATCCTCATAATCCTTCTTCTGTCTCCTATAACTTAACACCACGGTCCTGTAAAAAATAACAAAAAATACCAAACCAAGGGCAAATGCCACATTTGGACTATTGGCTAATTCTTCCCATATCATTCCCATATTTTCGCTAATTTTTCTAGCCTCACGTAATGATTCCACCTGCTTCTTCCTCCTTGTCTCTCTCTTAATCCTCCTCTCCTCCTGTTTCTTCTGCATCTTCATAGCCTTTTCAATCTTCTCATTTTCCTGCCTCATTTTCGTCAATTCAGTAGTAGTTTCCATCTTTACCTCATCCCTCAGACGCCTAAGCTGTGCAACTCTCTTGGACTCCTTCTTAGGTTGACCAAAATAAAACATAAAAAACGGTACTCTGGCCAAGAACCCTAAATACGGTGTCGGCAATTCAGGAGGCTTTATAGGAGGTGTATATGCATTAACACAAACAGAATCGATCCCCAACTCATTCCAAAGCACCCAAAACCTTCGATTACTCTCCACAGACGGCAACACCGTCCTCAACACTCTGGAATCCTCCAAAATCACCATTACTAATTCCGACCTCAGTCTCAAAAACCCATTAGGATTTTTAATCACATGTTTAACTTTTCCCTCCCTTTTCAAATCGAGAAGTTCGGTATAGGGAATCCGGTTTGAGATAGCTGGAAGTCCCTGGGACCAGGAAAGCAGCTCCTGAGGCGAAAGAGATTCAGCTTTTTTGGCGGAAGATCTCTTGGGTCCACGTCTTCGTTCCTTCACTTCTTTGGAAGCGGCGGCAGCGAGGGCGCTGGACATAGGAAGAGAGGTTGAAAGTATAGACAGAGTTACGGAGATTTGCAGGAAATTCAGCTTGTTCTTCTTCTTAGAATCGTTGTGTTCGTCGGTTGGAGAATCGGACGGTGTTGAAATTTGAGATGATATTGAGGGATGTGGACGACGCCATGTTCGTGGCTTAAGGGTCCCGAGCCTGGCACTGGAAAATGGGGAAGAAAGAGTGAGAAACCGTTCGCAGGCCATCAATTGCGTTCCAGCCGGGGAAATCGATGGAAGGAGAATTCACATGGTCGGAAGAGAAGAGCGGGAAATCATCTCCCTTTCGAGCCTCGGTATTTCAGTTTCTTGATGATAAGTTGTTTCGAATAAGTCTCTTCGGAAATTAAAAAAATGGGATAATTTTGAAGGGTATAAAGAGTTTTGGGATGGATTTTAGGACACATTAACTAGTAAATTTCTATATTGTCCGTCAATTAAAAAAACCCTAAAAATAAACTTTTTATTCTTCGTTTCCTTCCTCATTTTTGCGTTCTCCTTCTTTCTCACTCTTTTCTACATAGCAAAATGCTGCGACTTCAACTTTCTCCTTTCTCAGTTGTCGTCGTTGTCTCCTTCATCGTCGCCGATTGTTCAAGCCTCCATAGTGGTCAGTCCATGTA
